# Carbene-catalyzed enantioselective oxidative coupling of enals and di(hetero)arylmethanes†
†Electronic supplementary information (ESI) available. CCDC 1843274 and 1843275. For ESI and crystallographic data in CIF or other electronic format see DOI: 10.1039/c8sc03480j


**DOI:** 10.1039/c8sc03480j

**Published:** 2018-09-18

**Authors:** Qiao Chen, Tingshun Zhu, Pankaj Kumar Majhi, Chengli Mou, Huifang Chai, Jingjie Zhang, Shitian Zhuo, Yonggui Robin Chi

**Affiliations:** a Guiyang College of Traditional Chinese Medicine , Guizhou , P. R. China; b Division of Chemistry & Biological Chemistry , School of Physical & Mathematical Sciences , Nanyang Technological University , Singapore 637371 , Singapore . Email: robinchi@ntu.edu.sg

## Abstract

Di(hetero)arylmethane is a unique structural motif for natural and synthetic functional molecules. To date, it remains challenging to functionalize the diaryl methyl sp3 carbon–hydrogen bond directly in an enantioselective manner. This is likely due to the relatively inert nature of the carbon–hydrogen bond and the difficult enantiofacial discrimination of two sterically similar aryl substituents. Here we disclose an N-heterocyclic carbene-catalyzed direct oxidative coupling of enals and di(hetero)arylmethanes. Our method allows for highly enantioselective transformation of diaryl methanes and quick access to benzimidazole fused lactams.

## Abstract 2

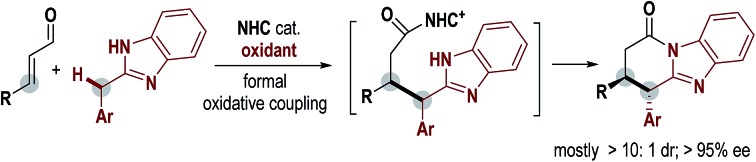
An N-heterocyclic carbene-catalyzed direct oxidative coupling of enals and di(hetero)arylmethanes allows for quick access to optical pure benzimidazole-fused lactams.

## Introduction

**Fig. 1:**
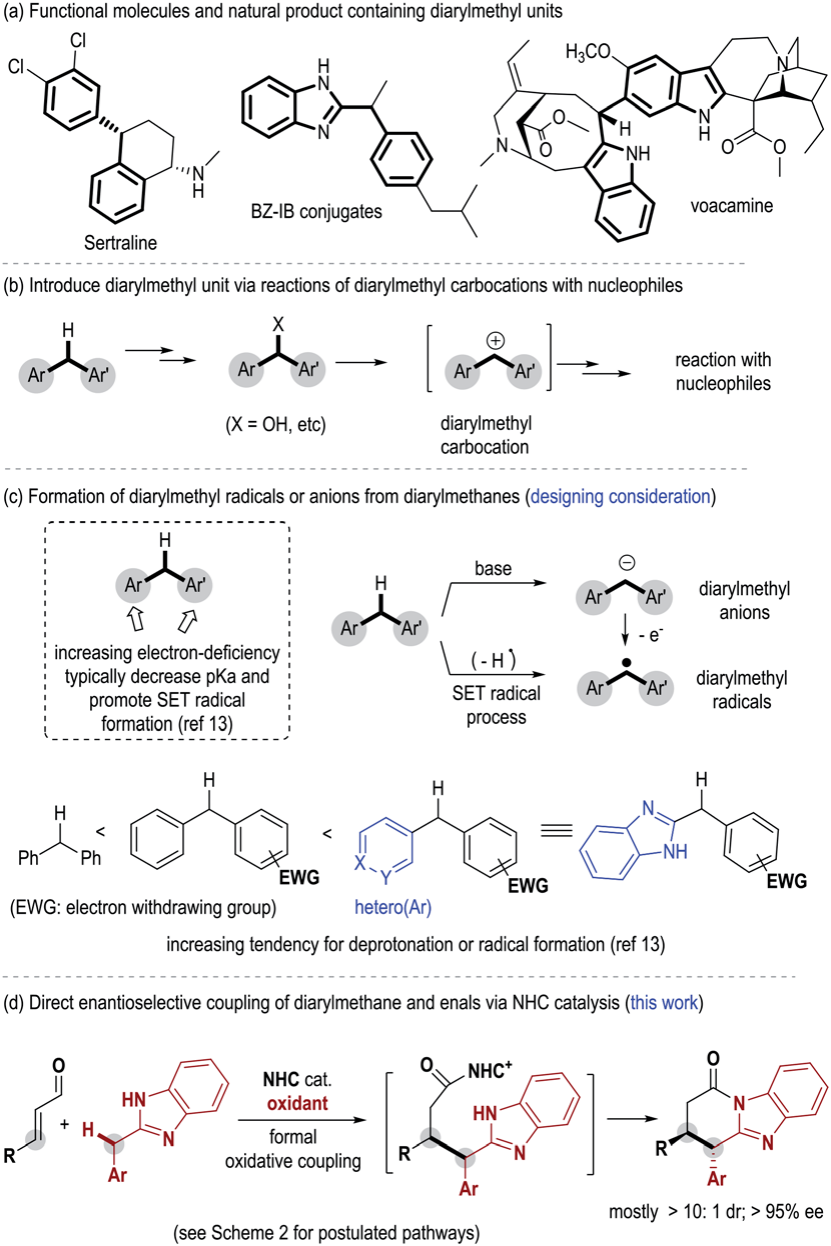
Diarylmethyl compounds and their enantioselective synthesis.

Diarylmethyl units are widely present in functional molecules and natural products. Examples of such molecules include the antidepressant drug Sertraline,^1^ Bz–IB conjugates with anti-inflammatory activities^2^ and the natural product voacamine^3^ with cannabinoid CB1 receptor antagonistic activity (Fig. 1a). Notably, the diaryl methyl sp3 carbon in these bioactive molecules is often present as a chiral center.^4^ To date, the most well-studied approach for the incorporation of diaryl methane unit relies on reactions between diarylmethyl carbocations and nucleophilic substrates^5^ (Fig. 1b). Diarylmethyl carbocation intermediates are typically generated *in situ* from their precursors such as diarylmethanols.^6^ In contrast, as a potentially more straightforward approach, direct modification of diarylmethanes is much less studied (Fig. 1c). This is in part due to the weak acidity of the methyl C–H and the difficult enantiofacial discrimination of two sterically similar aryl substituents. Deprotonation of such a benzylic C–H bond typically requires the use of a strong base such as ^*n*^BuLi or Na.^7^ As an alternative choice, oxidative transformations of diarylmethanes *via* benzylic organometallic species^8^ or benzylic radicals^9^ can be realized under milder conditions. However, in all these approaches, direct enantioselective modification of diarylmethane remains challenging. Limited examples in this direction include oxidative or photoredox transformations *via o*-quinone methides (*o*-QM)^10^ or *o*-quinodimethanes(*o*-QDM)^11^ as the key intermediates, and recent Rh-mediated asymmetric C–H arylation of diarylmethane.^12^

Herein we disclose an N-heterocyclic carbene (abbreviated as NHC or carbene) organic catalyst-mediated formal oxidative coupling of enal β-carbon and diarylmethanes (Fig. 1d). Our initial designing consideration is to generate a diarylmethyl radical from a diarylmethane substrate as a key intermediate under oxidative conditions (Fig. 1c). It is known that the tendency for deprotonation and radical formation of benzylic carbons can be well-tuned by altering the electronic properties of the aryl substituents^13^ (Fig. 1c). We therefore eventually introduce heterocyclic benzimidazole as one of the aryl units of the diarylmethane substrate in order to achieve suitable reactivity. The selection of benzimidazole units also provides two additional benefits: benzimidazole is a structural analogue of purine and a potential pharmacophore;^14^ the NH group in benzimidazole can facilitate the turnover of the NHC catalyst *via* the formation of a lactam product at the end of the catalytic cycle.^15^ Mechanistic studies suggest that some of our carbene-catalyzed reactions likely go through single-electron-transfer (SET) processes and radical intermediates in the key steps, although an electron pair pathway cannot be completely ruled out.

## Results and discussion

Key results in searching for suitable conditions by using cinnamaldehyde (**1a**) and diarylmethane **2** as model substrates are summarized in Table 1. We firstly used achiral triazolium salt **A**^16^ as the NHC pre-catalyst, DABCO as the base, and 3,3′,5,5′-tetra-*tert*-butyl-4,4′-diphenoquinone (DQ) as the oxidant.^17^ With 2-benzyl benzimidazole **2a** as the substrate, the proposed lactam product was not observed in an appreciable amount under various conditions. We then introduced an electron-withdrawing nitro group into the phenyl ring of diarylmethane (using **2b** as the substrate), and obtained the corresponding lactam product in 22% yield (entry 2). The use of amino indanol-derived triazolium carbene pre-catalyst **B**^18^ led to the lactam product in 36% yield with excellent ee (95% ee, entry 3). Several chiral NHC catalysts evaluated here did not provide better results in terms of both yields and ee values (entries 4–6). We then decided to use pre-catalyst **B** for further condition optimization (entries 7–12). The switch of the base from DABCO to DMAP significantly improved the yield from 36% (entry 3) to 60% (entry 7) with a slight increase of ee (from 95% to 98% ee) as well. Further optimization by using 1.5 equivalents of **1a** and DQ and performing the reaction at 40 °C gave a better result of 84% yield and 98% ee (entry 8). Several oxidants (phenazine,^19^ hexachloroethane^20^ and 4-nitro pyridine *N*-oxide^21^) previously used in oxidation and single-electron-transfer (SET) radical reactions could also mediate the reaction with low to moderate yields and excellent ee values (entries 9–11). Finally, the reaction outcome with **B** as the catalyst could be further improved by lowering the reaction concentration (entry 12).

**Table 1:** Screening of reaction conditions^*a*^

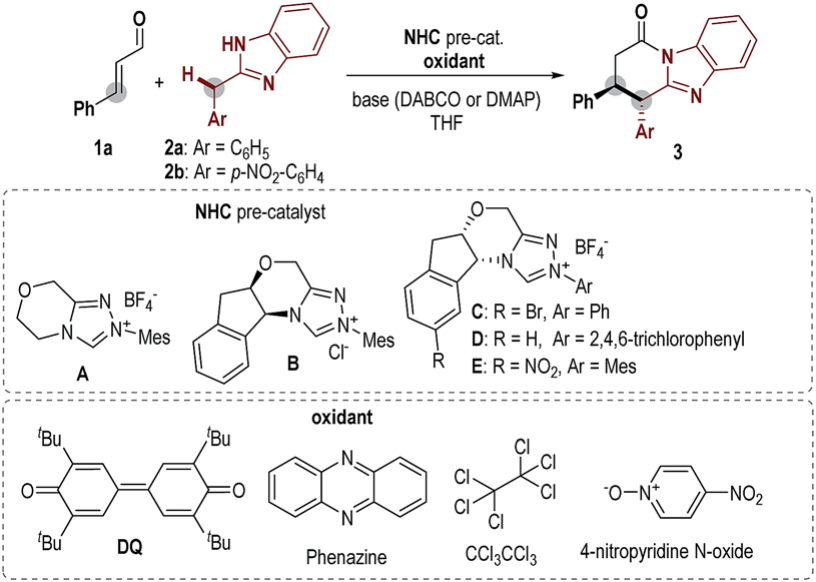					
Entry	**2**	NHC, base	Oxidant	Yield^*b*^/%	ee^*c*^/%
1	**2a**	**A**, DABCO	DQ	Trace	—
2	**2b**	**A**, DABCO	DQ	22	—
3	**2b**	**B**, DABCO	DQ	36	95
4	**2b**	**C**, DABCO	DQ	27	–73
5	**2b**	**D**, DABCO	DQ	18	–77
6	**2b**	**E**, DABCO	DQ	25	–95
7	**2b**	**B**, DMAP	DQ	60	98
8^*d*^	**2b**	**B**, DMAP	DQ	84	98
9^*d*^	**2b**	**B**, DMAP	Phenazine	46	96
10^*d*^	**2b**	**B**, DMAP	CCl_3_CCl_3_	7	nd
11^*d*^	**2b**	**B**, DMAP	4-Nitropyridine *N*-oxide	40	98
12^*d*^ ^,^^*e*^	**2b**	**B**, DMAP	DQ	92	98

^*a*^Reaction conditions: **1a** (0.06 mmol, 1.2 equiv.), **2** (0.05 mmol, 1.0 equiv.), NHC (20 mol%), base (0.5 equiv.) and oxidant (1.2 equiv.) in 0.5 mL THF at rt.

^*b*^Determined by ^1^H NMR, with 1,3,5-trimethoxybenzene as an internal standard.

^*c*^Determined by chiral-phase HPLC analysis.

^*d*^1.5 equiv. of **1a** and oxidant were used; reaction temperature was 40 °C.

^*e*^In 1 mL THF. DABCO = 1,4-diazabicyclo[2.2.2]octane; DMAP = 4-dimethylaminopyridine.

With an acceptable condition in hand (Table 1, entry 12), we evaluated the scope of enals by using **2b** as a model diarylmethane substrate (Table 2). Placing halogens, carboxylic esters, nitro groups, methyl, and methoxyl units as substituents at the *para*-position of the enal β-phenyl ring was well tolerated (**3a–3h**). The absolute configuration of **3c** was unambiguously confirmed by single-crystal X-ray diffraction analysis (for details, see the ESI†). The use of enals with different substitution patterns on the phenyl ring all gave products with excellent yield, dr, and ee values (**3i–3k**). Heterocyclic, naphthalene, and alkene substituents were all tolerated in β-substitution of enals (**3l–3o**). Enal with a β-carboxylate substituent gave product **3p** in moderate yield with excellent dr and good ee. The use of enals with a β-alkyl substituent led to products with low yields (around 10% yields as estimated *via* NMR).

**Table 2:** Examples of enal substrates^*a*^

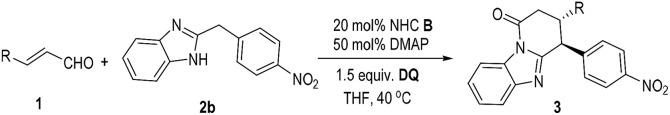
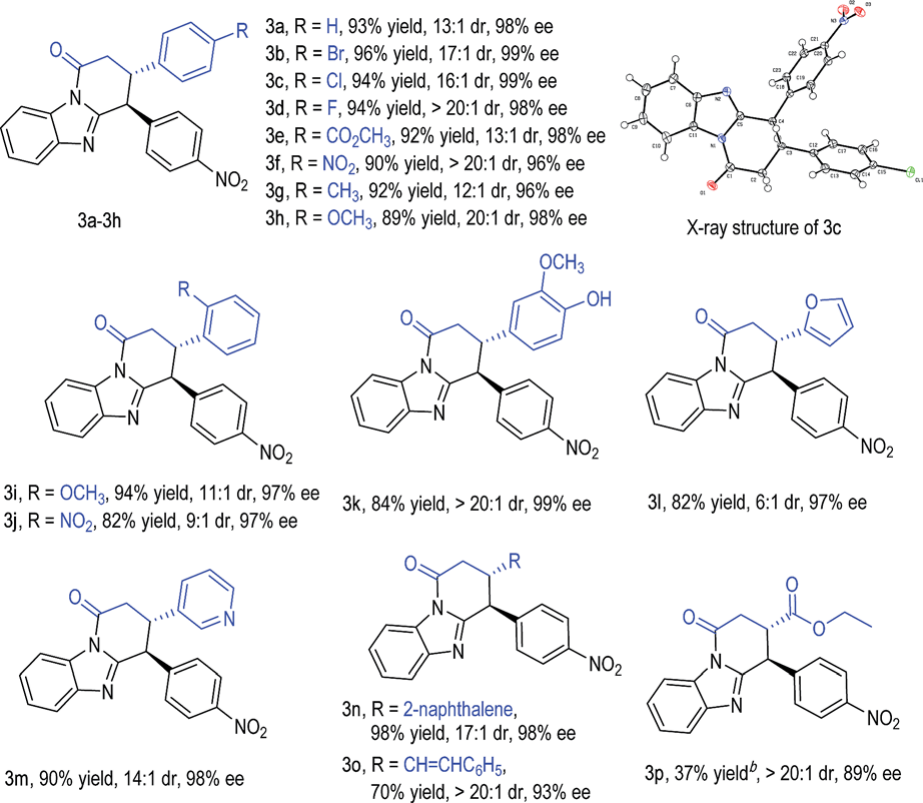

^*a*^Unless otherwise noted, all reactions were run on a 0.2 mmol scale under the standard conditions (Table 1, entry 12). Yield refers to isolated yield.

^*b*^The reaction temperature was 80 °C.

We next studied the scope of diarylmethanes by using cinnamaldehyde **1a** as the model enal substrate (Table 3). The nitro substituent in **2b** can be replaced with other electron-withdrawing units such as CN, CO_2_CH_3_, SO_2_CH_3_, CF_3_, and Br (**4b–4f**). Placing nitro groups at the *ortho*- or *meta*-position of the benzyl group of substrate **2** led to some decrease in reaction yields without affecting enantioselectivities (**4g–4h**). We also investigated the effect of substituents on the benzimidazole framework, and found that various substituents and substation patterns were well tolerated (**4i–4o**). When unsymmetrical benzimidazoles were used, the corresponding lactam products were obtained as a mixture of two regio-isomers (**4k–4o**, see the ESI† for details). This regio-isomer issue can be circumvented *via* one additional step in a one-pot operation: the lactam ring opened in acid alcohol in quantitative yield with ee and dr retained (as exemplified in **2k** to **5**, Table 3, one-pot procedure). Substrate bearing simple imidazolidine can also be used, giving product **4p** in 69% yield, 10 : 1 dr, and 98% ee.

**Table 3:** Examples of di(hetero)arylmethane substrates^*a*^

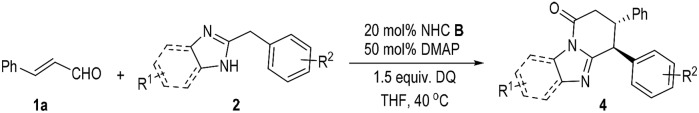
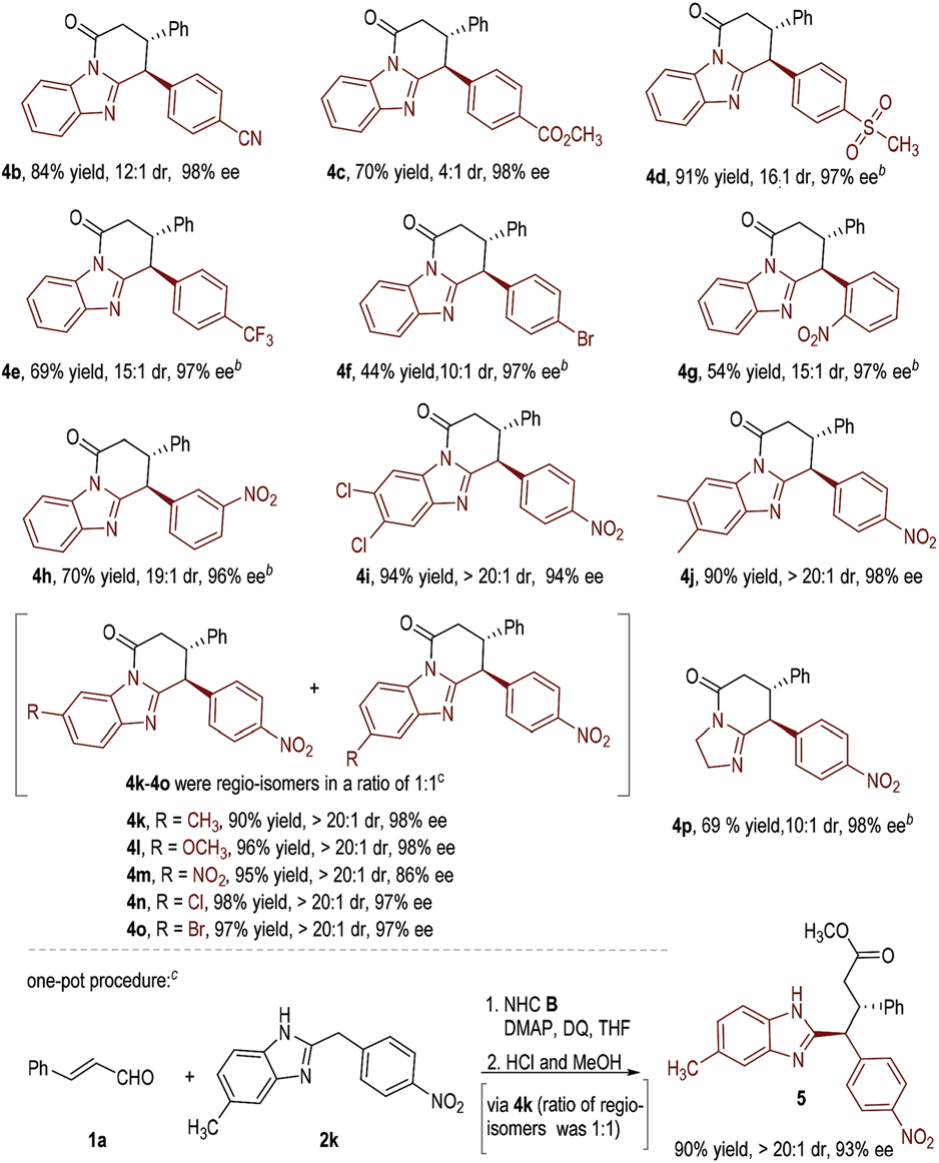

^*a*^Unless otherwise noted, all reactions were run on a 0.2 mmol scale under the standard conditions. Yield refers to isolated yield.

^*b*^The reaction temperature was 80 °C.

^*c*^The regio-isomer issues can be circumvented *via* one additional step in a one-pot operation.

**Scheme 1:**
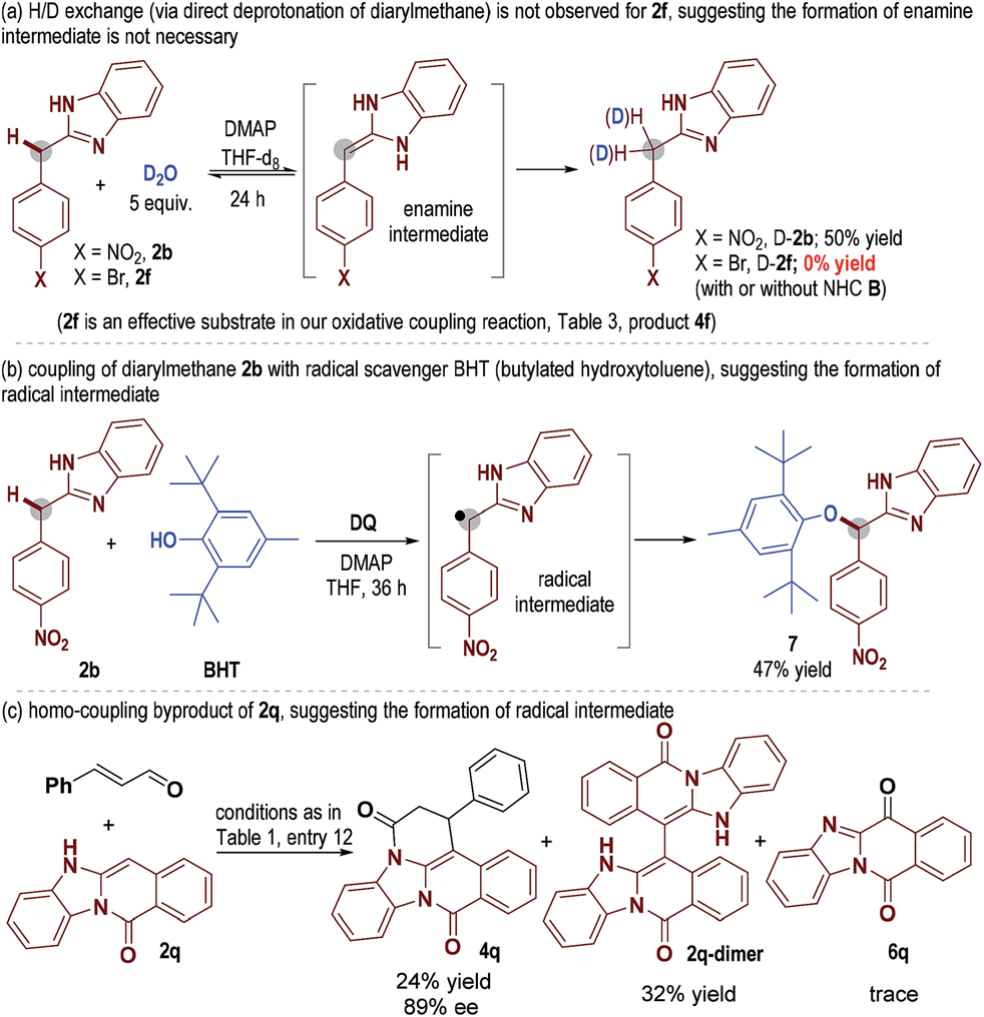
Control test.

**Scheme 2:**
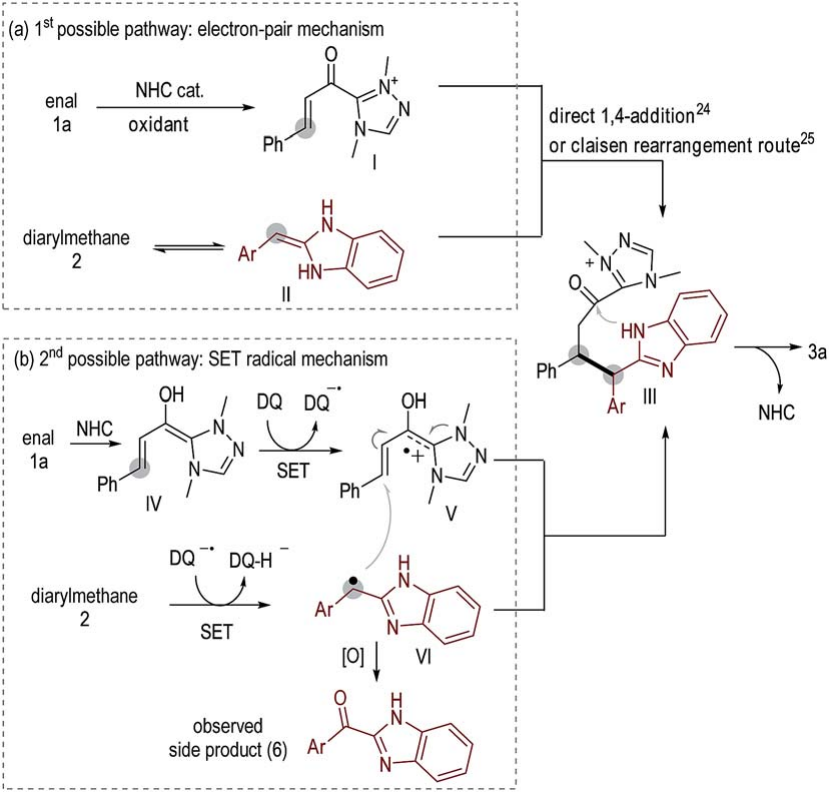
Possible reaction pathways.

To understand the reaction pathways, we performed several control experiments. We performed H/D exchange experiments for diarylmethane substrates **2b** and **2f** (Scheme 1a). With **2b** as the substrate, deuterated adduct D-**2b** was observed in 50% yield (NMR yield, see the ESI† for details). However, when **2f** (an effective substrate in our oxidative coupling reaction, Table 3, product **4f**) was used, no H/D exchange was observed. This result suggested that the formation of an enamine intermediate was not necessary in our catalytic coupling reaction. When **2b** was mixed with the radical scavenger BHT (butylated hydroxytoluene) in the presence of a DQ oxidant, adduct **7** could be obtained in 47% yield (Scheme 2b), suggesting the existence of benzylic radical intermediates. It is also worth noting that ketones **6** derived from **2***via* oxidation of the benzylic carbon were observed as the main side products in nearly all examples. **2q-dimer** generated from homo-coupling of benzylic carbon was also observed when **2q** was used as a substrate (Scheme 1c). This evidence suggested the presence of benzylic radical intermediates (see the ESI† for details of other mechanism study).

Two possible reaction pathways are illustrated in Scheme 2. In the 1st possible pathway (Scheme 2a), oxidation of **1a** under NHC catalysis leads to α,β-unsaturated azolium ester intermediate **I**.^22^ Isomerization of **2** leads to an enamine intermediate **II**. Nucleophilic addition of **II** to **I***via* an electron-pair pathway^23^ (direct 1,4-addition^24^ or Claisen rearrangement^25^ route) leads to **III**. Cyclization of **III** leads to product **3a** with regeneration of the NHC catalyst. In the 2nd possible pathway (Scheme 2b), NHC-bound radical cation intermediate **V** is formed *via* a SET oxidative process^21^,^26^ starting from enal **1a**. Under oxidative conditions, diarylmethane **2** is converted to a benzylic radical intermediate **VI**.^9^ Coupling of radical intermediates **V** and **VI***via* a SET process^27^ leads to intermediate **III**, which is subsequently converted to product **3a**. Ketones **6** derived from **2***via* oxidation of the benzylic carbon, as well as the homo-coupling generated **2**-dimer (Scheme 1c), were observed as the main side products in nearly all examples, suggesting the presence of radical intermediate **VI**.^9^

**Scheme 3:**
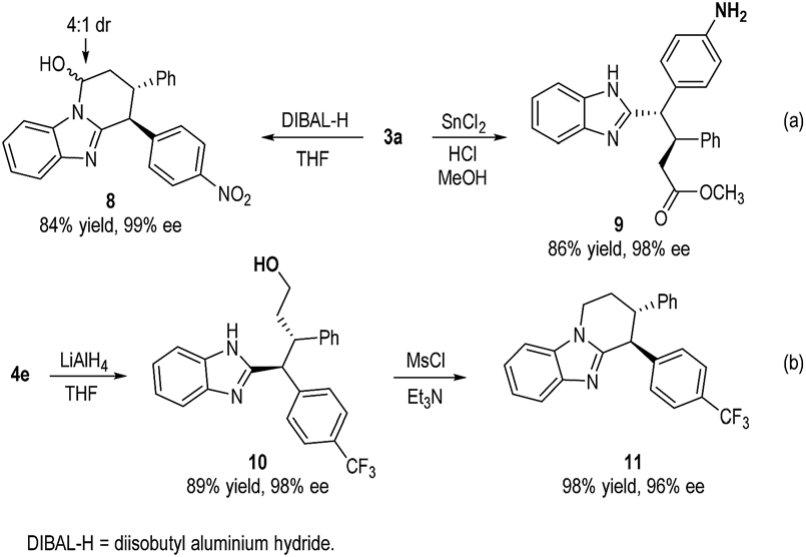
Product Transformation.

The oxidative coupling products from our catalytic reactions can undergo further transformations under simple conditions (Scheme 3). For example, adduct **3a** was reduced by DIBAL-H to *N*,*O*-acetal **8** in 84% yield. Nitro group reduction with simultaneously ring opening of lactam can also transform **3a** to ester **9** in 86% yield. Reduction of adduct **4e** with LiAlH_4_ efficiently gave alcohol **10** (89% yield), which was further cyclized under MsCl to afford benzimidazole fused piperidine **11** in 98% yield. In all cases, the ee and dr of the molecules were completely retained during the transformations.

## Conclusions

In summary, we have developed an NHC-catalyzed highly enantioselective oxidative coupling of di(hetero)arylmethanes and enals to give benzimidazole fused lactams. Ongoing studies include the development of effective methods for enantioselective oxidative coupling of more challenging inactivated carbon–hydrogen bonds, application of this method for assembly or modification of pesticides and active components of Chinese medicines, and bioactivity evaluations of relevant molecules.

## Conflicts of interest

There are no conflicts of interest to declare

## Supplementary Material

Supplementary information

Crystal structure data
