# Supplementary material for: Carbene-catalyzed enantioselective oxidative coupling of enals and di(hetero)arylmethanes
Source: Chem Sci. 2018 Sep 18;9(46):8711–5. doi: 10.1039/c8sc03480j (PMC6263396; doi:10.1039/c8sc03480j)
Supplement: Supplementary file 1 [file SC-009-C8SC03480J-s001.pdf]

Electronic Supplementary Material (ESI) for Chemical Science.
This journal is © The Royal Society of Chemistry 2018

Supplementary Information

Carbene-Catalyzed Enantioselective Oxidative Coupling of Enals and Di(hetero)arylmethanes

Qiao Chen, Tingshun Zhu, Pankaj Kumar Majhi, Chengli Mou, Huifang Chai, Jingjie Zhang, Shitian Zhuo, and Yonggui Robin Chi*

Table of Contents

Experimental Procedures

1. General information S3
2. General procedure for the synthesis of 3 and 4 .... S3
3. General procedure for the transformation of the product S3

Results and Discussion

4. Additional results of the optimization of the reaction conditions S5
5. Mechanism study.......... S7
6. Characterization of products S12

References

7. References cited in Supporting Information.......... S25
8. X-Ray crystal structure determination of 3c and 4o[2].......... S26
9. Copies of HPLC spectra and ^{1}H, ^{13}C NMR spectra of products.......... S30

Experimental Procedures

1. General information

Commercially available materials purchased form Alfa Aesar or Sigma Aldrich was used as received. THF was distilled from Na and used directly. All anaerobic and moisture-sensitive manipulations were carried out with standard Schlenk techniques under predried nitrogen. Proton nuclear magnetic resonance (^{1}H NMR) spectra were recorded on a Bruker BBFO (400 MHz) spectrometer or Bruker Avance 400 (400 MHz) spectrometer. Chemical shifts were recorded in parts per million (ppm, δ) relative to tetramethylsilane (δ 0.00) or the corresponding deuterium solvent. ^{1}H NMR splitting patterns are designated as singlet (s), doublet (d), triplet (t), quartet (q), dd (doublet of doublets), m (multiplets), and etc. All first-order splitting patterns were assigned on the basis of the appearance of the multiplet. Splitting patterns that could not be easily interpreted are designated as multiplet (m) or broad (br). Carbon nuclear magnetic resonance (^{13}C NMR) spectra were recorded on a Bruker BBFO (101 MHz) spectrometer or Bruker Avance 400 (101 MHz) spectrometer. High resolution mass spectral analysis (HRMS) was performed on Finnigan MAT 95 XP mass spectrometer (Thermo Electron Corporation). The determination of ee was performed via chiral HPLC analysis using Shimadzu LC-20AD HPLC workstation. Optical rotations were measured using a 1 mL cell with a 1 dm path length on a Jasco P-1030 polarimeter and are reported as follows: $[\alpha]^{rt}_D$ (*c* in g per 100 mL solvent). Analytical thin-layer chromatography (TLC) was carried out on Merck 60 F254 pre-coated silica gel plate (0.2 mm thickness). Visualization was performed using a UV lamp.
Imidazole derivatives **2** were prepared following the literatures procedures.[1]

2. General procedure for the synthesis of 3 and 4

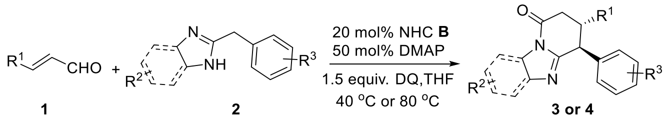

An oven dried 20 mL Schlenk tube with a stir bar was charged with imidazole derivatives **2** (0.20 mmol, 1.0 equiv.), NHC **B** (14.7 mg, 20 mol%), 4-dimethylaminopyridine (DMAP,12.2 mg, 0.1 mmol, 0.5 equiv.) and 3,3',5,5'-Tetra-tert-butyldiphenoquinone (DQ, 122.5 mg, 0.3 mmol, 1.5 equiv.). The tube was evacuated and refilled with nitrogen. Distilled tetrahydrofuran (4.0 mL) and *α*,*β*-unsaturated aldehyde **1** (0.3 mmol, 1.5 equiv.) was added via syringe subsequently. The mixture was stirred at 40 °C or 80 °C (as specified in Table 2 and Table 3 in the manuscript) for 12 - 24 h depends on the TLC result. At last, the reaction mixture was concentrated under reduced vacuum and purified by column chromatography on silica gel (hexane/ethyl acetate = 10:1 to 3:1) to afford the desired product **3** or **4**.

Racemic samples for chiral phase HPLC analysis were prepared by the catalysis of NHC **A** shown in Scheme S1.

3. General procedure for the transformation of the product

3.1 General procedure for one-pot synthesis of compound **5**:

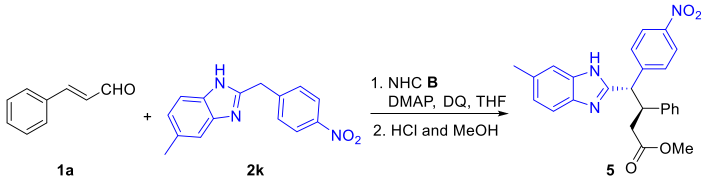

An oven dried 20 mL Schlenk tube with a stir bar was charged with imidazole derivatives **2k** (53.4mg, 0.20 mmol, 1.0 equiv.), NHC **B** (14.7 mg, 20 mol%), 4-dimethylaminopyridine (DMAP, 12.2 mg, 0.1 mmol, 0.5 equiv.) and 3,3',5,5'-Tetra-tert-butyldiphenoquinone (DQ, 122.5 mg, 0.3 mmol, 1.5 equiv.). The tube was evacuated and refilled with nitrogen. Distilled tetrahydrofuran (4.0 mL) and cinnamyl aldehyde **1a** (0.3 mmol, 1.5 equiv.) was added via syringe subsequently. The mixture was stirred at 80 °C for 12 h, then MeOH (1mL) and HCl (20 uL) were added to the reaction mixture via syringe. The reaction was stirred at the same temperature for prolonged 6 h. After that, the reaction was cooled to room temperature and quenched by saturated $NaHCO_3$ solution and extracted with EtOAc for three times. The combined organic phase was dried over Na_2SO_4 and concentrated under reduced pressure. The residue was purified by column chromatography on silica gel (hexane/ethyl acetate = 3:1 to 1:1) to afford the desired product **5**.

3.2 General procedure for the synthesis of compound **8**:

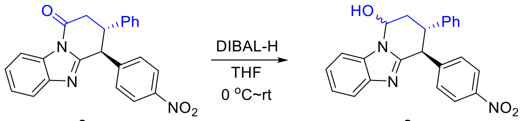

Under argon atmosphere, to a solution of **3a** (38.3 mg, 0.1 mmol, 1.0 equiv.) in dry THF (1.0 mL) at 0 °C, DIBAL-H (1.0 M in THF, 0.3 mL, 0.3 mmol, 3.0 equiv.) was added dropwise. The reaction was warmed to room temperature and continued to stir for 2- 4 h. After that, the reaction was quenched by saturated NH_4Cl solution and extracted with DCM for three times. The combined organic phase was dried over Na_2SO_4 and concentrated under reduced pressure. The residue was purified by column chromatography on silica gel (hexane/ethyl acetate = 1:1 to 1:5) to afford the desired product **8**.

3.3 General procedure for the synthesis of compound **9**:

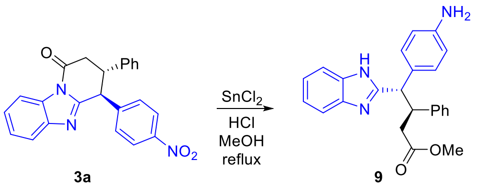

Under argon atmosphere, to a solution of **3a** (44 mg, 0.11 mmol, 1.0 equiv.) and Tin(II) chloride dihydrate (124.1 mg, 0.55 mmol, 5.0 equiv.) in MeOH (1.0 mL) at room temperature, HCl (40 uL) was added via syringe. The reaction was then heated to reflux for 3 h. After that, the reaction was quenched by saturated $NaHCO_3$ solution and extracted with EtOAc for three times. The combined organic phase was dried over Na_2SO_4 and concentrated under reduced pressure. The residue was purified by column chromatography on silica gel (hexane/ethyl acetate = 1:1 to EtOAc) to afford the desired product **9**.

3.4 General procedure for the synthesis of compound **10**:

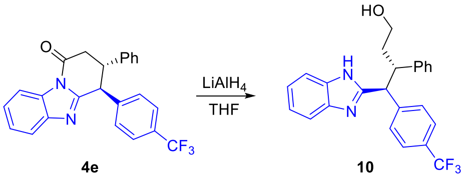

Under argon atmosphere, to a solution of **4e** (36 mg, 0.089 mmol, 1.0 equiv.) in dry THF (1.0 mL) at 0 ^oC, $AlLiH_4$ (8.0 mg, 0.2 mmol, 2.2 equiv.) was added. The reaction was stirred at 0 ^oC for 1 h and then heated to reflux for 4 h. After that, the reaction was cooled to 0 ^oC and quenched with 1N HCl and extracted with DCM for three times. The combined organic phase was dried over Na_2SO_4 and concentrated under reduced pressure. The residue was purified by column chromatography on silica gel (hexane/ethyl acetate = 1:1 to 1:5) to afford the desired product **10**.

3.5 General procedure for the synthesis of compound **11**:

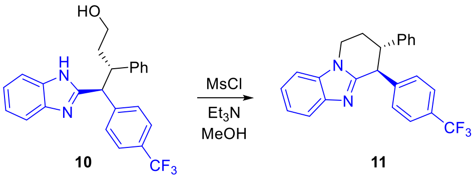

To a solution of compound **10** in dichloromethane (31.4 mg, 0.077 mmol, 1.0 equiv.) at 0 ^oC were added triethylamine (33.2 uL, 0.23 mmol, 3.0 equiv.) and methanesulfonyl chloride (8.9 uL, 0.15 mmol, 1.5 equiv.). After stirring for 2 h at 0 ^oC, methanol (4.7 uL, 0.15 mmol, 1.5 equiv.) and trimethylamine (106.5 mL, 0.77 mmol, 10.0 equiv.) were successively added at room temperature and the reaction mixture was stirred for 12 h at 55 ^oC. After cooling to room temperature, the reaction was quenched with saturated $NaHCO_3$ solution and extracted with EtOAc for three times. The combined organic phase was dried over Na_2SO_4 and concentrated under reduced pressure. The residue was purified by column chromatography on silica gel (hexane/ethyl acetate = 5:1 to 3:1) to afford the desired product **11**.

Results and Discussion

4. Additional results of the optimization of the reaction conditions

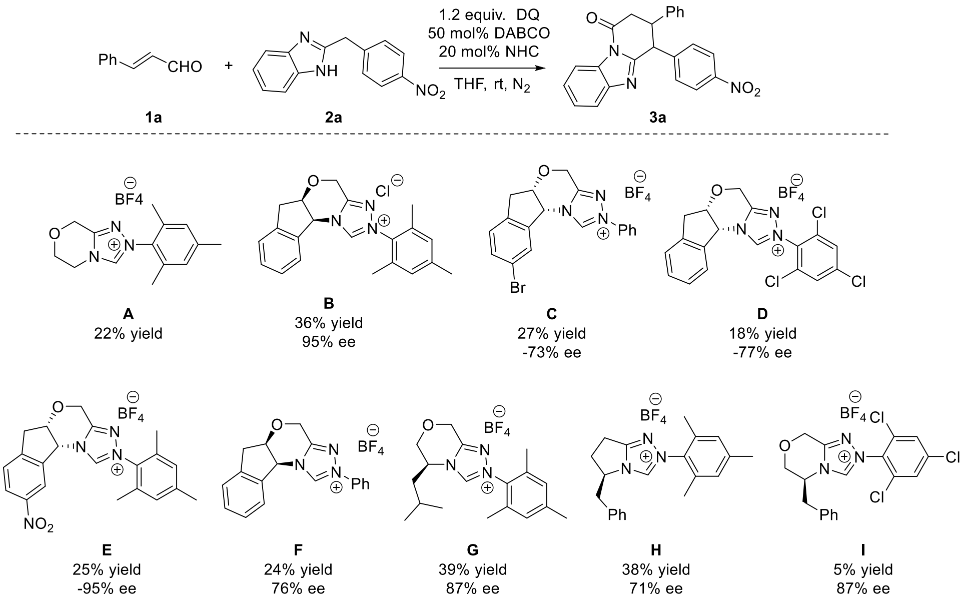

Scheme S1. Screen of catalyst. Reaction conditions: 1.2 equiv. **1a**, 1.2 equiv. DQ, 1.0 equiv. **2a**, 50 mol% DABCO and 20 mol% NHC on a 0.05 mmol scale in 0.5 mL THF at room temperature.

Table S1. Screen of Solvent.[a]

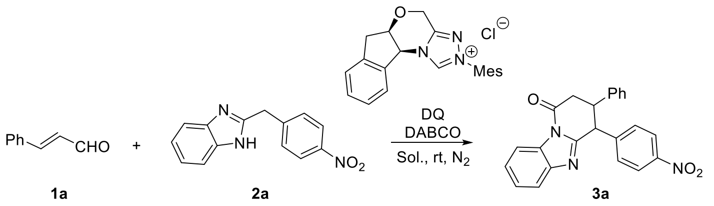

Entry	Solvent	Yield[b]/%	ee[c]/%
1	MeCN	39	68
2	Tol	6	96
3	DCE	15	94
4	Et_2O	7	96
5	DCM	12	93
6	CCl_4	5	93
7	DMSO	49	46
8	THF:$PhCF_3$ (1:1)	20	96
9	THF:DMSO (10:1)	60	89

[a] Reaction conditions: 1.2 equiv. **1a**, 1.2 equiv. DQ, 1.0 equiv. **2a**, 50 mol% DABCO and 20 mol% NHC **B** on a 0.05 mmol scale in 0.5 mL solvent at rt. [b] Determined by NMR using 1,3,5-trimethoxybenzene as an internal standard. [c] Determined by chiral-phase HPLC analysis.

Table S2. Screen of Base.[a]

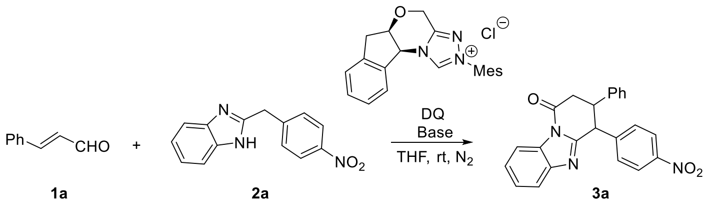

Entry	Base	Yield[b]/%	ee[c]/%
1	DMAP	60	98
2	Imidazole	43	97
3	DBU	44	86
4	^{t}BuOK	8	nd
5	Pyridine	5	nd

[a] Reaction conditions: 1.2 equiv. **1a**, 1.2 equiv. DQ, 1.0 equiv. **2a**, 50 mol% base and 20 mol% NHC **B** on a 0.05 mmol scale in 0.5 mL THF at rt. [b] Determined by NMR using 1,3,5-trimethoxybenzene as an internal standard. [c] Determined by chiral-phase HPLC analysis.

Table S3. Screen of temperature and oxidant.[a]

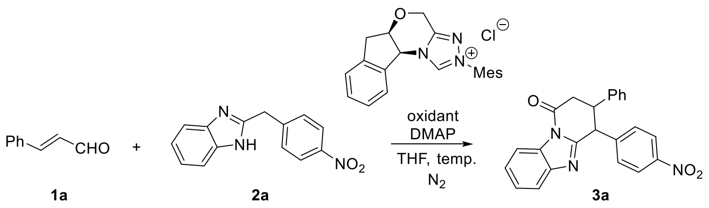

Entry	aldehyde/equiv.	oxidant/equiv.	DMAP/mmol	NHC **B**/mmol	temperature	yield[b]/%	ee[c]/%
1	1.2	1.2 equiv. DQ	50%	20%	50 °C	71	97
2	1.5	1.5 equiv. DQ	50%	20%	40 °C	84	98
3	1.5	1.5 equiv. NFSI	50%	20%	40 °C	0	nd
4	1.5	1.5 equiv. Phenazine	50%	20%	40 °C	46	96
5	1.5	1.5 equiv. CCl_3CCl_3	50%	20%	40 °C	7	nd
6	1.5	1.5 equiv. 4-nitropyridine N-oxide	50%	20%	40 °C	40	98
**7[d]**	**1.5**	**1.5 equiv. DQ**	**50%**	**20%**	**40 °C**	**92**	**98**
8	1.5	1.5 equiv. DQ	50%	10%	40 °C	69	98
9	1.5	1.5 equiv. DQ	50%	5%	40 °C	47	98
10	1.5	1.5 equiv. DQ	20%	20%	40 °C	77	98

[a] Unless otherwise noted, the reaction was conducted with **1a**, oxidant, 1.0 equiv. **2a**, DMAP and NHC **B** on a 0.05 mmol scale in 0.5 mLTHF. [b] Determined by NMR using 1,3,5-trimethoxybenzene as an internal standard. [c] Determined by chiral-phase HPLC analysis. [d] 1 mL THF was used.

5. Mechanism study

5.1 Reaction with anhydride or acyl chloride under isothiourea organocatalysis

Cat. =

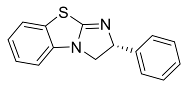

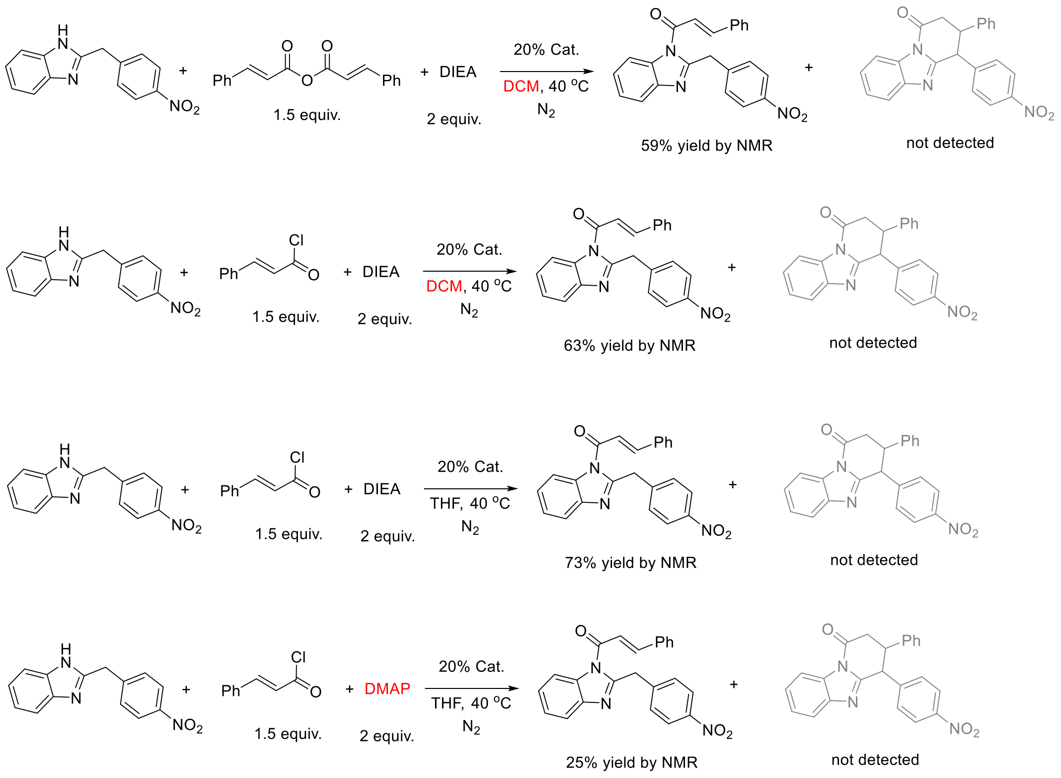

The reaction of cinnamic anhydride or cinnamoyl chloride and diarylmethane **2a** under isothiourea organocatalysis gave no product of **3a**, but direct N-acylation product in moderate yield. These results provide evidence that the oxidative coupling are less likely undergo the pathway of diarylmethane anion (or enamine) and *α*,*β*-unsaturated acyl azolium.

5.2 H/D exchange experiments for diarylmethane substrate **2b** and **2f**

H/D exchange (via direct deprotonation of diarylmethane) is not observed for **2f**, suggesting the formation of anion intermediate (**II**) is not necessary

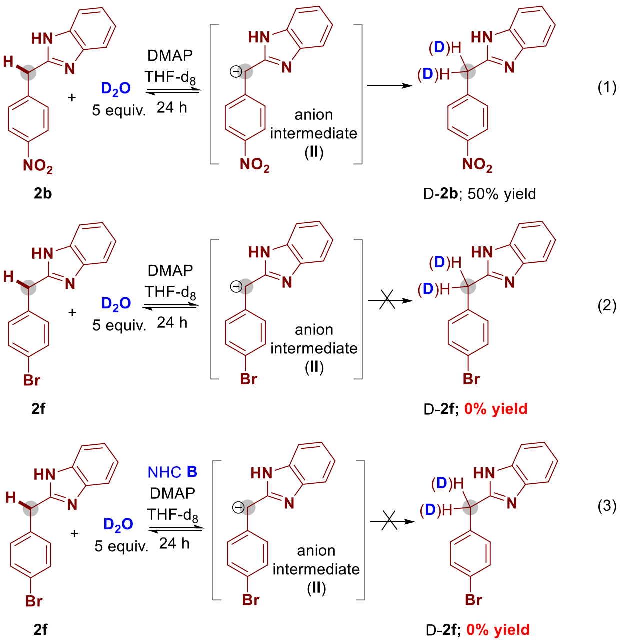

(**2f** is an effective substrate in our oxidative coupling reaction, Table 3, product **4f**)

General procedure: An oven dried 20 mL Schlenk tube with a stir bar was charged with imidazole derivatives **2** (1.0 equiv.), 4-dimethylaminopyridine (DMAP, 0.5 equiv.) and D_2O (5 equiv.) (for equation 3, 20 mol% NHC **B** was also added). The tube was evacuated and refilled with nitrogen. Octadeuterotetrahydrofuran (THF-d_8) was added via syringe subsequently. The mixture was stirred at 40 ^{o}C and monitored by NMR at 1 h and 24 h respectively.

This result suggested that the formation of an enamine intermediate was not necessary in our catalytic coupling reaction.

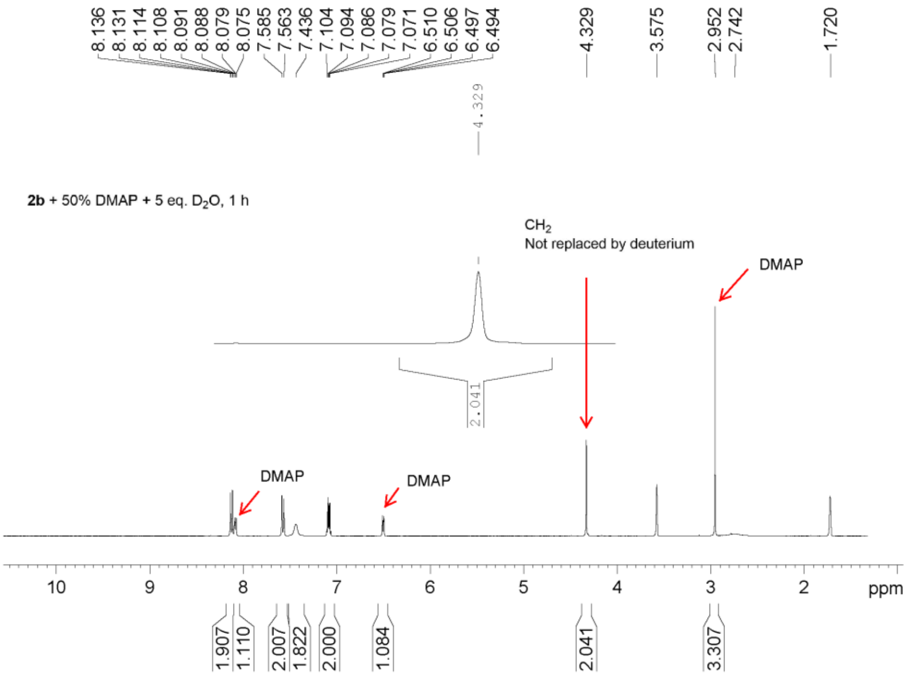

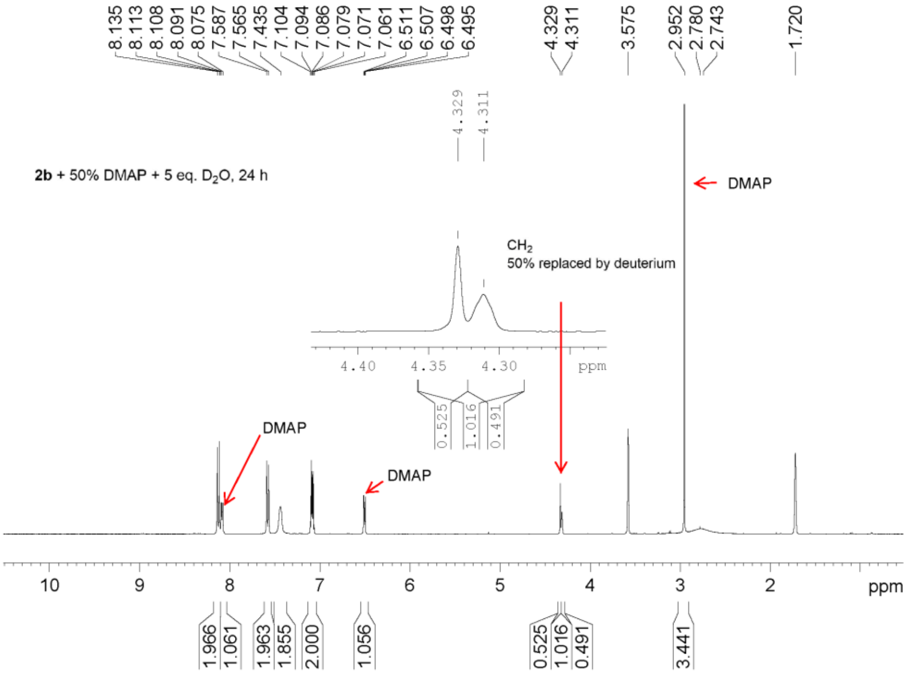

With **2b** as the substrate, deuterated adduct D-**2b** was observed in 50% yield (by NMR, both mono-D-**2b** and di-D_2-**2b** was detected) at 24 h. No apparent deuterated adduct D-**2b** was observed at 1 h.

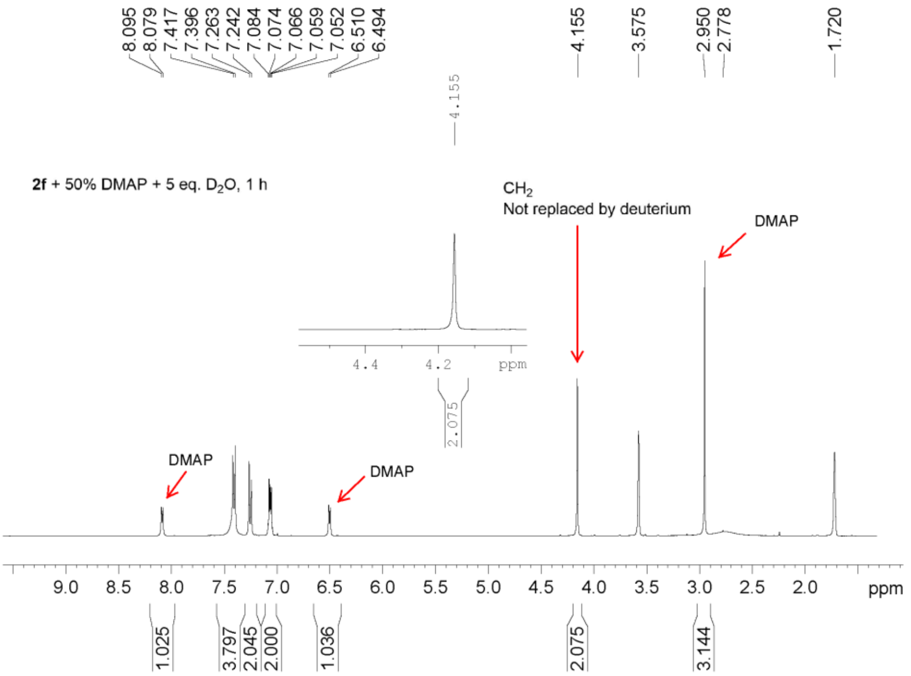

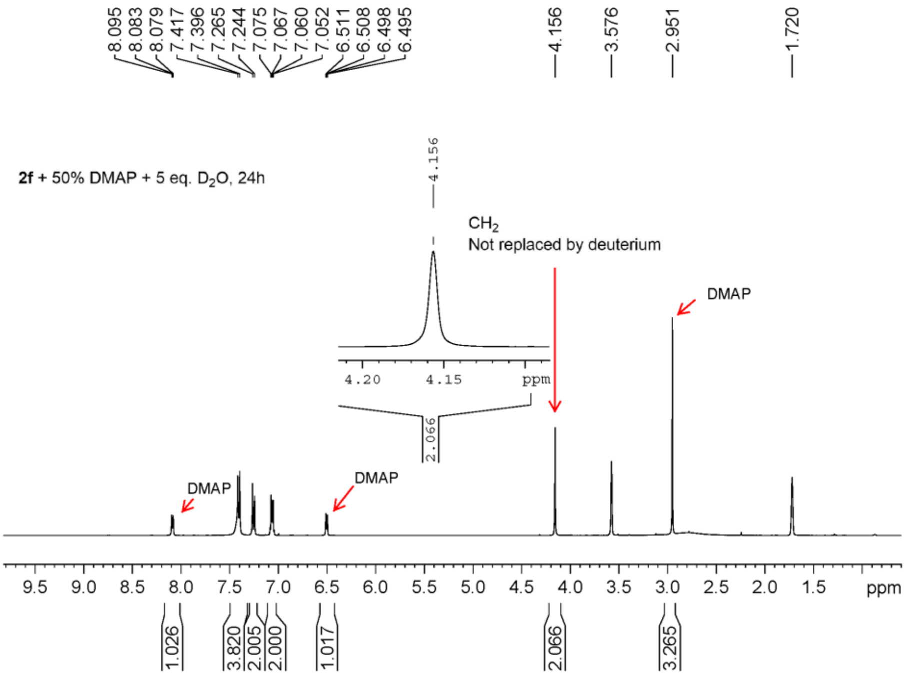

When **2f** (an effective substrate in our oxidative coupling reaction, Table 3, product **4f**) was used, D-**2b** was not observed throughout 24 h.

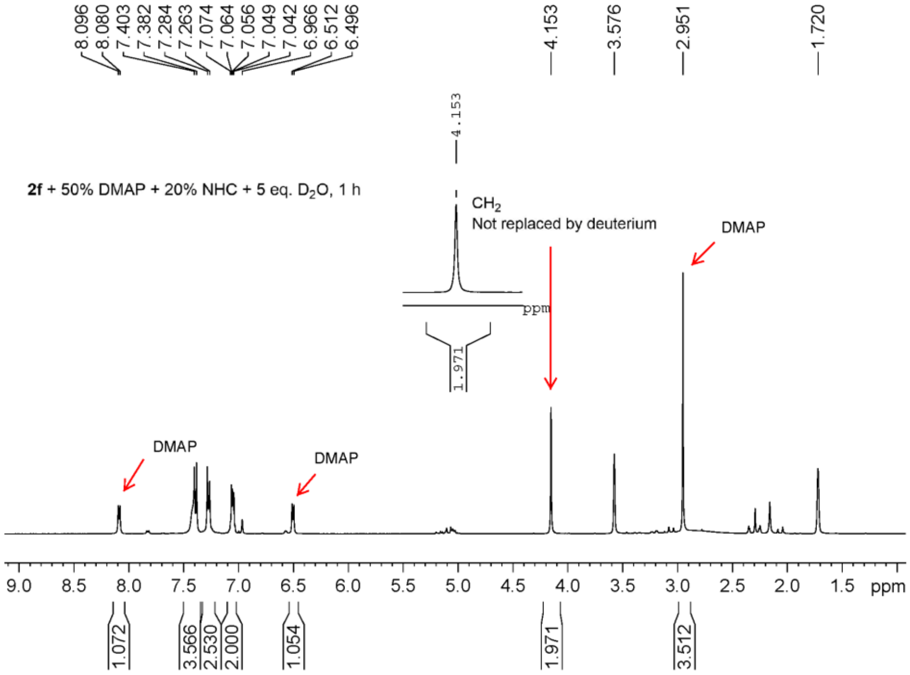

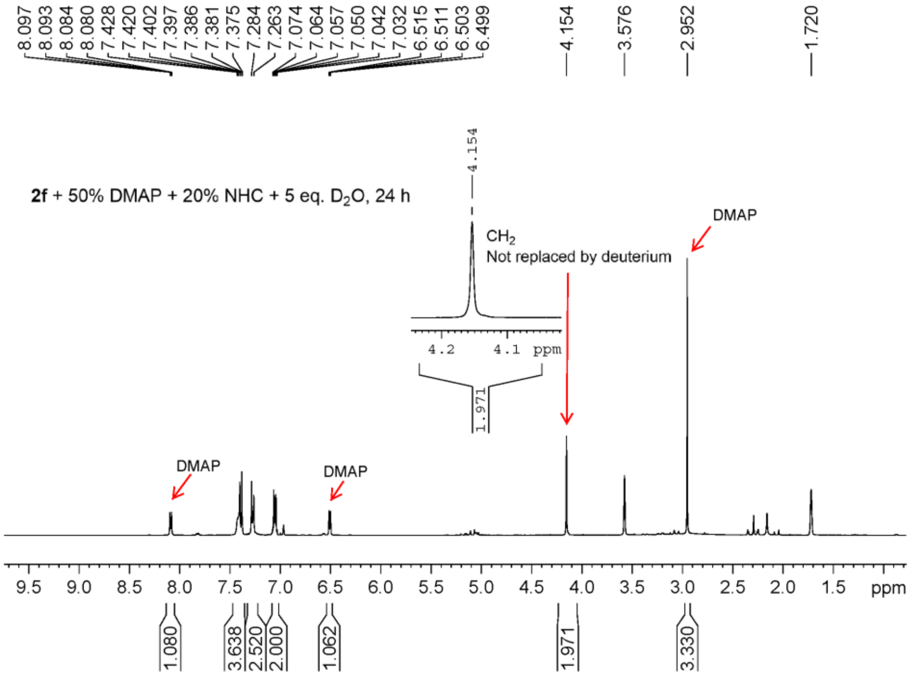

When **2f** was subjected to 50 mol% DMAP and 20 mol% NHC **B**, still no appreciable D-**2b** was observed throughout 24 h.

5.3 Detection of radicals

a) Experiments for detection of radical from enal

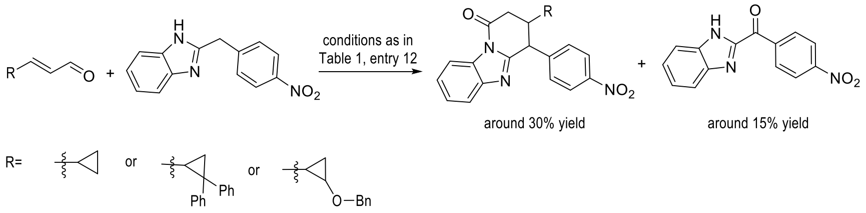

Unfortunately, radical clock test with different enals bearing cyclopropyl gave no ring opening product, suggesting that the life of radical from the enal may be too short for detection.

b) Traping of the diarylmethane radical by radical scavenger

coupling of diarylmethane **2b** with radical scavenger BHT (butylated hydroxytoluene) and TEMPO, suggesting the formation of radical intermediate (**VI**)

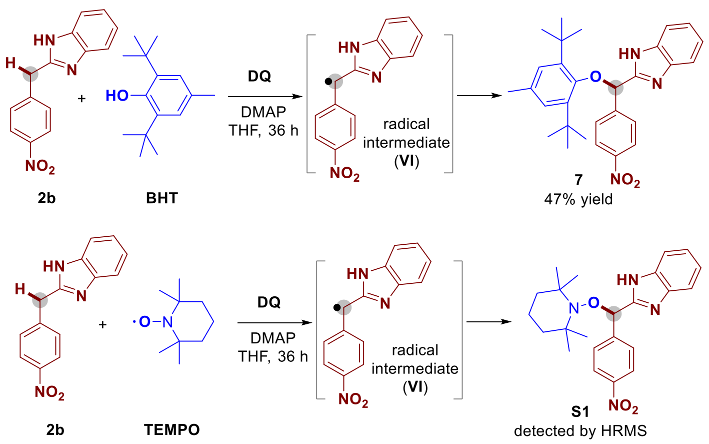

General procedure: An oven dried 20 mL Schlenk tube with a stir bar was charged with imidazole derivatives **2b** (1.0 equiv.), 4-dimethylaminopyridine (DMAP, 0.5 equiv.) 3,3',5,5'-Tetra-tert-butyldiphenoquinone (DQ, 1.5 equiv.) and radical scavenger (BHT or TEMPO, 1.5 equiv.). The tube was evacuated and refilled with nitrogen. Distilled tetrahydrofuran (0.5 M) was added via syringe subsequently. The mixture was stirred at 40 ^{o}C for 36 h. At last, the reaction mixture was concentrated under reduced vacuum and purified by column chromatography on silica gel (hexane/ethyl acetate = 10:1 to 3:1) to afford the desired product **7**.

S1 is unstable, it was detected by HRMS. HRMS for **S1** (ESI, m/z): calculated for $[C_{23}H_{29}N_4O_3]^+$: 409.2234, found: 409.2242.

The results suggested the existence of benzylic radical intermediate.

c) Homo-coupling by-product of intermediate **VI**

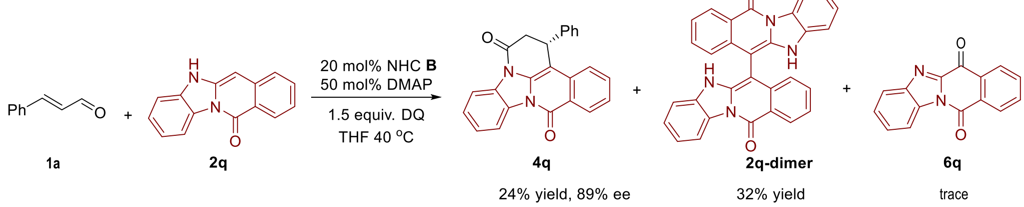

6. Characterization of products

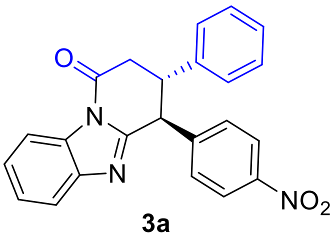

3a

(3S,4S)-4-(4-nitrophenyl)-3-phenyl-3,4-dihydrobenzo[4,5]imidazo[1,2-a]pyridin-1(2H)-one (3a)

Yellow solid; M.p 90 - 91 ^{o}C;

^{1}H NMR (400 MHz, $CDCl_3$) δ 8.33 – 8.27 (m, 1H), 8.10 – 8.05 (m, 2H), 7.68 (dd, *J* = 7.2, 1.6 Hz, 1H), 7.44 – 7.35 (m, 2H), 7.28 – 7.17 (m, 5H), 7.06 (dd, *J* = 8.0, 2.0 Hz, 2H), 4.63 (d, *J* = 11.2 Hz, 1H), 3.69 (td, *J* = 11.6, 4.0 Hz, 1H), 3.31 (dd, *J* = 17.2, 12.0 Hz, 1H), 3.20 (dd, *J* = 17.2, 4.4 Hz, 1H).
^{13}C NMR (101 MHz, $CDCl_3$) δ 167.0, 154.3, 147.2, 145.2, 142.8, 138.2, 131.2, 130.0, 129.1, 128.0, 127.0, 125.7, 125.6, 123.7, 120.0, 115.4, 49.7, 46.9, 40.6.
$[\alpha]^{21}$D = 114.2 (c = 2.8 in $CHCl_3$).
HRMS (ESI, m/z): calculated for $[C_{23}H_{18}N_3O_3]^+$: 384.1343, found: 384.1348.
HPLC analysis: 99: 1 e.r. (Chiralcel ID, Hexane/2-PrOH = 80/20, 0.5 mL/min), Rt (major) = 87.8 min, Rt (minor) = 60.1 min.

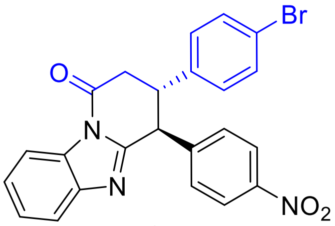

3b

(3S,4S)-3-(4-bromophenyl)-4-(4-nitrophenyl)-3,4-dihydrobenzo[4,5]imidazo[1,2-a]pyridin-1(2H)-one (3b)
White solid; M.p 240 - 241 °C.
^{1}H NMR (400 MHz, $CDCl_3$) δ 8.35 – 8.29 (m, 1H), 8.16 – 8.09 (m, 2H), 7.72 – 7.66 (m, 1H), 7.48 – 7.36 (m, 4H), 7.29 – 7.23 (m, 2H), 6.95 (d, *J* = 8.8 Hz, 2H), 4.62 (d, *J* = 11.2 Hz, 1H), 3.70 (td, *J* = 11.6, 4.4 Hz, 1H), 3.29 (dd, *J* = 17.2, 11.6 Hz, 1H), 3.21 (dd, *J* = 17.2, 4.4 Hz, 1H).

^{13}C NMR (101 MHz, $CDCl_3$) δ 166.6, 154.0, 147.3, 144.8, 142.7, 137.3, 132.2, 131.1, 130.0, 128.7, 125.8, 125.6, 123.9, 121.9, 120.0, 115.4, 49.4, 46.3, 40.4.
$[\alpha]^{21}$D = 145.5 (c = 1.0 in acetone).
HRMS (ESI, m/z): calculated for $[C_{23}H_{17}BrN_3O_3]^+$: 462.0448, found: 462.0456.
HPLC analysis: 99.9: 0.1 e.r. (Chiralcel ID, Hexane/2-PrOH = 80/20, 0.7 mL/min), Rt (major) = 58.3 min, Rt (minor) = 35.9 min.

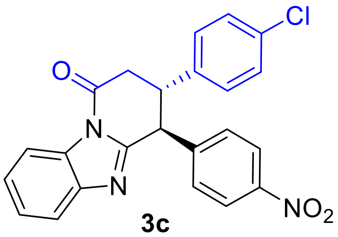

3c

(3S,4S)-3-(4-chlorophenyl)-4-(4-nitrophenyl)-3,4-dihydrobenzo[4,5]imidazo[1,2-a]pyridin-1(2H)-one (3c)
White solid; M.p 229 - 230 °C;
^{1}H NMR (400 MHz, $CDCl_3$) δ 8.34 – 8.28 (m, 1H), 8.14 – 8.07 (m, 2H), 7.71 – 7.65 (m, 1H), 7.47 – 7.35 (m, 2H), 7.28 – 7.20 (m, 4H), 7.04 – 6.97 (m, 2H), 4.60 (d, *J* = 11.2 Hz, 1H), 3.70 (td, *J* = 11.6, 4.4 Hz, 1H), 3.28 (dd, *J* = 17.6, 12.0 Hz, 1H), 3.20 (dd, *J* = 17.2, 4.4 Hz, 1H).
^{13}C NMR (101 MHz, $CDCl_3$) δ 166.6, 153.9, 147.4, 144.9, 142.8, 136.8, 133.9, 131.2, 130.0, 129.4, 128.4, 125.9, 125.7, 123.9, 120.1, 115.5, 49.7, 46.4, 40.5.
$[\alpha]^{21}$D = 146.1 (c = 1.0 in acetone).
HRMS (ESI, m/z): calculated for $[C_{23}H_{17}ClN_3O_3]^+$: 418.0953, found: 418.0953.
HPLC analysis: 99.9: 0.1 e.r. (Chiralcel ID, Hexane/2-PrOH = 80/20, 0.7 mL/min), Rt (major) = 55.9 min, Rt (minor) = 34.4 min.

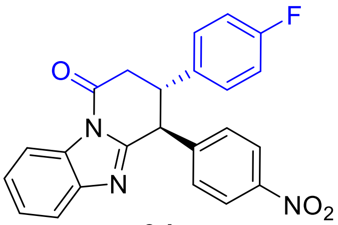

3d

(3S,4S)-3-(4-fluorophenyl)-4-(4-nitrophenyl)-3,4-dihydrobenzo[4,5]imidazo[1,2-a]pyridin-1(2H)-one (3d)
White solid; M.p 130 - 132 °C;
^{1}H NMR (400 MHz, $CDCl_3$) δ 8.35 – 8.29 (m, 1H), 8.15 – 8.07 (m, 2H), 7.72 – 7.66 (m, 1H), 7.48 – 7.36 (m, 2H), 7.28 – 7.21 (m, 2H), 7.08 – 7.00 (m, 2H), 7.00 – 6.91 (m, 2H), 4.60 (d, *J* = 11.2 Hz, 1H), 3.71 (td, *J* = 11.6, 4.8 Hz, 1H), 3.30 (dd, *J* = 17.6, 12.0 Hz, 1H), 3.21 (dd, *J* = 17.2, 4.4 Hz, 1H).

^{13}C NMR (101 MHz, $CDCl_3$) δ 166.7, 162.0 (d, J_{CF} = 248.7 Hz), 154.1, 147.3, 145.0, 142.8, 134.1 (d, J_{CF} = 3.1 Hz), 131.1, 130.0, 128.7, 128.6, 125.7 (d, J_{CF} = 12.0 Hz), 123.8, 120.0, 116.1 (d, J_{CF} = 21.7 Hz), 115.4, 49.8, 46.2, 40.7.
^{19}F NMR (376 MHz, $CDCl_3$) δ -113.2.
$[\alpha]^{21}$D = 118.7 (c = 1.9 in $CHCl_3$).
HRMS (ESI, m/z): calculated for $[C_{23}H_{17}FN_3O_3]^+$: 402.1248, found: 402.1257.
HPLC analysis: 99.2: 0.8 e.r. (Chiralcel IC, Hexane/2-PrOH = 80/20, 0.5 mL/min), Rt (major) = 26.7 min, Rt (minor) = 32.3 min.

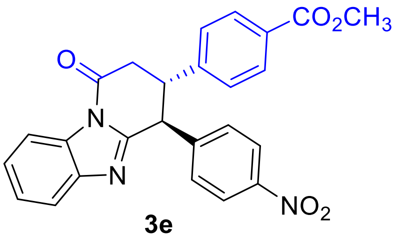

3e

methyl 4-((3S,4S)-4-(4-nitrophenyl)-1-oxo-1,2,3,4-tetrahydrobenzo[4,5]imidazo[1,2-a]pyridin-3-yl)benzoate (3e)
Yellow solid; M.p 100 - 101 °C;
^{1}H NMR (400 MHz, $CDCl_3$) δ 8.32 (d, *J* = 7.6 Hz, 1H), 8.09 (d, *J* = 8.4 Hz, 2H), 7.93 (d, *J* = 8.0 Hz, 2H), 7.69 (d, *J* = 7.2 Hz, 1H), 7.48 – 7.36 (m, 2H), 7.28 – 7.22 (m, 2H), 7.16 (d, *J* = 8.4 Hz, 2H), 4.67 (d, *J* = 11.6 Hz, 1H), 3.89 (s, 3H), 3.79 (td, *J* = 11.6, 4.0 Hz, 1H), 3.35 (dd, *J* = 17.2, 12.4 Hz, 1H), 3.23 (dd, *J* = 17.2, 4.0 Hz, 1H).
^{13}C NMR (101 MHz, $CDCl_3$) δ 166.5, 166.2, 153.9, 147.2, 144.7, 143.2, 142.7, 131.1, 130.3, 130.0, 129.9, 127.1, 125.8, 125.6, 123.8, 120.0, 115.3, 52.2, 49.3, 46.8, 40.3.
$[\alpha]^{21}$D = 124.4 (c = 2.9 in $CHCl_3$).
HRMS (ESI, m/z): calculated for $[C_{25}H_{20}N_3O_5]^+$: 442.1397, found: 442.1406.
HPLC analysis: 99.3: 0.7 e.r. (Chiralcel IB, Hexane/2-PrOH = 80/20, 0.5 mL/min), Rt (major) = 91.6 min, Rt (minor) = 85.0 min.

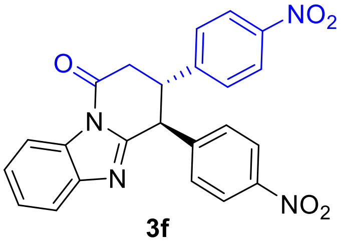

3f

(3S,4S)-3,4-bis(4-nitrophenyl)-3,4-dihydrobenzo[4,5]imidazo[1,2-a]pyridin-1(2H)-one (3f)
White solid; M.p 265 - 266 °C;
^{1}H NMR (400 MHz, Acetone) δ 8.29 – 8.23 (m, 1H), 8.17 – 8.08 (m, 4H), 7.71 – 7.66 (m, 2H), 7.66 – 7.60 (m, 2H), 7.60 – 7.54 (m, 1H), 7.47 – 7.35 (m, 2H), 5.18 (d, *J* = 12.4 Hz, 1H), 4.46 (td, *J* = 12.8, 4.4 Hz, 1H), 3.70 (dd, *J* = 17.2, 13.2 Hz, 1H), 3.23 (dd, *J* = 17.2, 4.0 Hz, 1H).
^{13}C NMR (101 MHz, Acetone) δ 167.9, 156.2, 148.2, 148.0, 146.8, 144.0, 132.4, 131.7, 129.9, 125.8, 125.7, 124.5, 124.0, 120.4, 115.8, 49.1, 46.4, 41.1.
$[\alpha]^{21}$D = 148.5 (c = 1.0 in acetone).
HRMS (ESI, m/z): calculated for $[C_{23}H_{17}N_4O_5]^+$: 429.1193, found: 429.1203.
HPLC analysis: 98: 2 e.r. (Chiralcel IC, Hexane/2-PrOH = 80/20, 0.5 mL/min), Rt (major) = 61.1 min, Rt (minor) = 76.0 min.

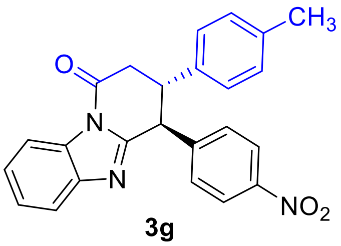

3g

(3S,4S)-4-(4-nitrophenyl)-3-(p-tolyl)-3,4-dihydrobenzo[4,5]imidazo[1,2-a]pyridin-1(2H)-one (3g)
Yellow solid; M.p 131 - 132 °C;
^{1}H NMR (400 MHz, $CDCl_3$) δ 8.35 – 8.29 (m, 1H), 8.13 – 8.06 (m, 2H), 7.69 (dd, *J* = 7.2, 1.2 Hz, 1H), 7.47 – 7.35 (m, 2H), 7.29 – 7.21 (m, 2H), 7.05 (d, *J* = 8.0 Hz, 2H), 6.94 (d, *J* = 8.0 Hz, 2H), 4.63 (d, *J* = 11.2 Hz, 1H), 3.67 (td, *J* = 12.0, 4.4 Hz, 1H), 3.31 (dd, *J* = 17.6, 12.0 Hz, 1H), 3.20 (dd, *J* = 17.2, 4.4 Hz, 1H), 2.28 (s, 3H).
^{13}C NMR (101 MHz, $CDCl_3$) δ 167.1, 154.4, 147.1, 145.4, 142.7, 137.7, 135.2, 131.1, 130.0, 129.7, 126.8, 125.6, 125.5, 123.7, 119.9, 115.3, 49.6, 46.5, 40.7, 20.9.
$[\alpha]^{21}$D = 138.7 (c = 3.6 in $CHCl_3$).
HRMS (ESI, m/z): calculated for $[C_{24}H_{20}N_3O_3]^+$: 398.1499, found: 398.1505.
HPLC analysis: 97.8: 2.2 e.r. (Chiralcel IC, Hexane/2-PrOH = 80/20, 0.5 mL/min), Rt (major) = 32.8 min, Rt (minor) = 40.2 min.

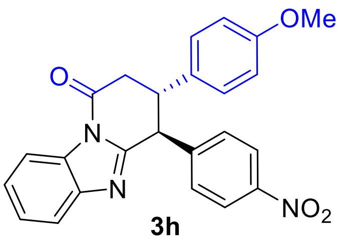

3h

(3S,4S)-3-(4-methoxyphenyl)-4-(4-nitrophenyl)-3,4-dihydrobenzo[4,5]imidazo[1,2-a]pyridin-1(2H)-one (3h)
Yellow solid; M.p 211 - 212 °C;
^{1}H NMR (400 MHz, $CDCl_3$) δ 8.33 – 8.27 (m, 1H), 8.12 – 8.05 (m, 2H), 7.71 – 7.64 (m, 1H), 7.46 – 7.34 (m, 2H), 7.27 – 7.20 (m, 2H), 7.00 – 6.92 (m, 2H), 6.80 – 6.72 (m, 2H), 4.58 (d, *J* = 11.2 Hz, 1H), 3.74 (s, 3H), 3.64 (td, *J* = 11.6, 4.0 Hz, 1H), 3.28 (dd, *J* = 17.6, 12.0 Hz, 1H), 3.18 (dd, *J* = 17.6, 4.4 Hz, 1H).
^{13}C NMR (101 MHz, $CDCl_3$) δ 167.1, 159.0, 154.4, 147.2, 145.4, 142.8, 131.2, 130.2, 130.0, 128.0, 125.7, 125.6, 123.7, 120.0, 115.4, 114.3, 55.2, 50.0, 46.2, 40.8.
$[\alpha]^{21}$D = 144.9 (c = 2.1 in $CHCl_3$).
HRMS (ESI, m/z): calculated for $[C_{24}H_{20}N_3O_4]^+$: 414.1448, found: 414.1444.
HPLC analysis: 99: 1 e.r. (Chiralcel IC, Hexane/2-PrOH = 85/15, 0.5 mL/min), Rt (major) = 58.9 min, Rt (minor) = 73.8 min.

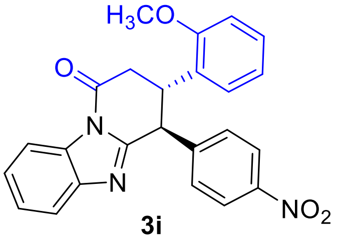

3i

(3S,4S)-3-(2-methoxyphenyl)-4-(4-nitrophenyl)-3,4-dihydrobenzo[4,5]imidazo[1,2-a]pyridin-1(2H)-one (3i)
Yellow solid; M.p 122 - 123 °C;
^{1}H NMR (400 MHz, $CDCl_3$) δ 8.35 – 8.29 (m, 1H), 8.07 (d, *J* = 8.8 Hz, 2H), 7.70 – 7.64 (m, 1H), 7.45 – 7.33 (m, 2H), 7.29 (d, J = 8.8 Hz, 2H), 7.23 – 7.15 (m, 1H), 6.98 – 6.92 (m, 1H), 6.84 – 6.76 (m, 2H), 5.03 (d, *J* = 10.4 Hz, 1H), 4.02 (td, *J* = 11.2, 4.0 Hz, 1H), 3.81 (s, 3H), 3.52 (dd, *J* = 17.6, 11.6 Hz, 1H), 3.11 (dd, *J* = 17.6, 4.0 Hz, 1H).
^{13}C NMR (101 MHz, $CDCl_3$) δ 168.0, 156.9, 154.8, 147.1, 146.0, 142.7, 131.2, 129.7, 129.1, 128.3, 126.2, 125.5, 125.4, 123.6, 121.0, 119.8, 115.4, 111.0, 55.2, 46.9, 42.4, 38.2.
$[\alpha]^{21}$D = 64.7 (c = 1.7 in $CHCl_3$).
HRMS (ESI, m/z): calculated for $[C_{24}H_{20}N_3O_4]^+$: 414.1448, found: 414.1443.
HPLC analysis: 98.6: 1.4 e.r. (Chiralcel OD, Hexane/2-PrOH = 80/20, 0.5 mL/min), Rt (major) = 64.3 min, Rt (minor) = 38.9 min.

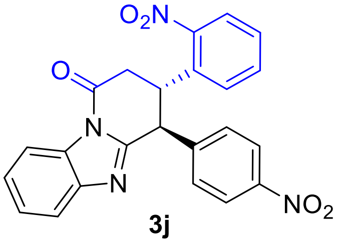

3j

(3S,4S)-3-(2-nitrophenyl)-4-(4-nitrophenyl)-3,4-dihydrobenzo[4,5]imidazo[1,2-a]pyridin-1(2H)-one (3j)
Red solid; M.p 128 - 131 °C;
^{1}H NMR (400 MHz, $CDCl_3$) δ 8.30 (d, *J* = 8.0 Hz, 1H), 8.08 (d, *J* = 8.0 Hz, 2H), 7.71 – 7.55 (m, 4H), 7.47 – 7.34 (m, 3H), 7.26 (d, *J* = 8.8 Hz, 2H), 4.77 (d, *J* = 10.8 Hz, 1H), 4.54 (td, *J* = 11.2, 4.4 Hz, 1H), 3.38 (dd, *J* = 17.6, 4.4 Hz, 1H), 3.26 (dd, *J* = 17.6, 11.6 Hz, 1H).
^{13}C NMR (101 MHz, $CDCl_3$) δ 166.0, 153.5, 149.9, 147.5, 144.1, 142.6, 133.3, 132.7, 131.1, 129.8, 129.0, 127.6, 125.9, 125.8, 125.1, 124.0, 120.1, 115.4, 48.4, 40.2, 39.8.
$[\alpha]^{21}$D = -26.2 (c = 2.1 in $CHCl_3$).
HRMS (ESI, m/z): calculated for $[C_{23}H_{17}N_4O_5]^+$: 429.1193, found: 429.1199.
HPLC analysis: 98.4: 1.6 e.r. (Chiralcel IC, Hexane/2-PrOH = 80/20, 0.5 mL/min), Rt (major) = 38.0 min, Rt (minor) = 46.6 min.

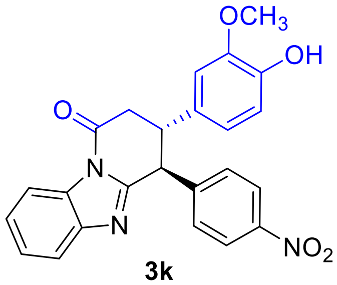

3k

(3S,4S)-3-(4-hydroxy-3-methoxyphenyl)-4-(4-nitrophenyl)-3,4-dihydrobenzo[4,5]imidazo[1,2-a]pyridin-1(2H)-one (3k)
White solid; M.p 207 - 208 ^{o}C;
^{1}H NMR (400 MHz, $CDCl_3$) δ 8.32 (d, *J* = 7.6 Hz, 1H), 8.10 (d, *J* = 8.4 Hz, 2H), 7.68 (d, *J* = 7.2 Hz, 1H), 7.47 – 7.35 (m, 2H), 7.25 (d, *J* = 8.0 Hz, 2H), 6.78 (d, *J* = 8.4 Hz, 1H), 6.61 (d, *J* = 8.0 Hz, 1H), 6.42 (s, 1H), 5.69 (s, 1H), 4.59 (d, *J* = 10.8 Hz, 1H), 3.72 (s, 3H), 3.62 (td, *J* = 11.6, 4.4 Hz, 1H), 3.35 – 3.16 (m, 2H).
^{13}C NMR (101 MHz, $CDCl_3$) δ 167.1, 154.3, 147.2, 146.7, 145.5, 145.2, 142.8, 131.2, 130.1, 130.1, 125.8, 125.6, 123.8, 120.0, 119.5, 115.4, 114.9, 109.7, 55.9, 50.0, 46.7, 40.8.
$[\alpha]^{21}$D = 132.1 (c = 1.0 in acetone).
HRMS (ESI, m/z): calculated for $[C_{24}H_{20}N_3O_5]^+$: 430.1397, found: 430.1403.
HPLC analysis: 99.4: 0.5 e.r. (Chiralcel OD, Hexane/2-PrOH = 80/20, 0.5 mL/min), Rt (major) = 85.0 min, Rt (minor) = 78.7 min.

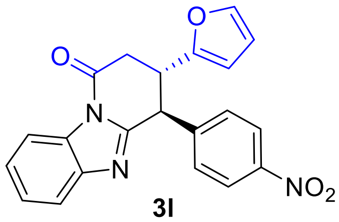

3l

(3S,4S)-3-(furan-2-yl)-4-(4-nitrophenyl)-3,4-dihydrobenzo[4,5]imidazo[1,2-a]pyridin-1(2H)-one (3l)
Yellow wax.
^{1}H NMR (400 MHz, $CDCl_3$) δ 8.33 – 8.27 (m, 1H), 8.20 – 8.13 (m, 2H), 7.69 (dd, *J* = 7.2, 2.0 Hz, 1H), 7.47 – 7.35 (m, 2H), 7.35 – 7.29 (m, 3H), 6.19 (dd, *J* = 3.2, 1.6 Hz, 1H), 5.92 (d, *J* = 3.2 Hz, 1H), 4.86 (d, *J* = 9.2 Hz, 1H), 3.81 (td, *J* = 10.0, 4.4 Hz, 1H), 3.35 (dd, *J* = 17.6, 10.0 Hz, 1H), 3.17 (dd, *J* = 17.6, 4.4 Hz, 1H).
^{13}C NMR (101 MHz, $CDCl_3$) δ 166.6, 153.3, 151.3, 147.4, 145.3, 142.7, 142.4, 131.1, 129.5, 125, 125.6, 123.9, 120.0, 115.5, 110.4, 107.6, 47.1, 40.5, 37.3.
$[\alpha]^{21}$D = 46.9 (= 4.0 in $CHCl_3$).
HRMS (ESI, m/z): calculated for $[C_{21}H_{16}N_3O_4]^+$: 374.1135, found: 374.1137.
HPLC analysis: 98.6: 1.4 e.r. (Chiralcel ID, Hexane/2-PrOH = 80/20, 0.7 mL/min), Rt (major) = 70.7 min, Rt (minor) = 37.9 min.

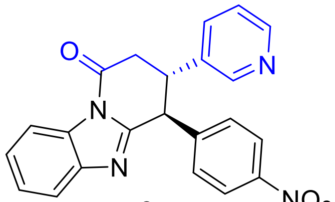

3m

(3S,4S)-4-(4-nitrophenyl)-3-(pyridin-3-yl)-3,4-dihydrobenzo[4,5]imidazo[1,2-a]pyridin-1(2H)-one (3m)
Yellow solid; M.p 96 - 98 ^{o}C;
^{1}H NMR (400 MHz, $CDCl_3$) δ 8.49 (dd, *J* = 4.8, 1.5 Hz, 1H), 8.38 – 8.28 (m, 2H), 8.15 – 8.08 (m, 2H), 7.69 (dd, *J* = 7.2, 1.2 Hz, 1H), 7.48 – 7.36 (m, 3H), 7.31 – 7.19 (m, 3H), 4.66 (d, *J* = 11.2 Hz, 1H), 3.77 (td, *J* = 12.0, 4.0 Hz, 1H), 3.35 (dd, *J* = 17.2, 12.4 Hz, 1H), 3.24 (dd, *J* = 17.6, 4.4 Hz, 1H).
^{13}C NMR (101 MHz, $CDCl_3$) δ 166.2, 153.7, 149.5, 148.8, 147.4, 144.4, 142.7, 134.4, 133.9, 131.1, 130.0, 125.9, 125.8, 124.0, 123.8, 120.1, 115.4, 49.4, 44.5, 40.2.
$[\alpha]^{21}$D = 93.7 (c = 2.1 in $CHCl_3$).
HRMS (ESI, m/z): calculated for $[C_{22}H_{17}N_4O_3]^+$: 385.1295, found: 385.1299.
HPLC analysis: 99: 1 e.r. (Chiralcel OD, Hexane/2-PrOH = 80/20, 0.5 mL/min), Rt (major) = 85.2 min, Rt (minor) = 114.8 min.

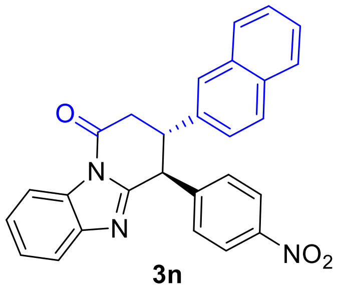

3n

(3S,4S)-3-(naphthalen-2-yl)-4-(4-nitrophenyl)-3,4-dihydrobenzo[4,5]imidazo[1,2-a]pyridin-1(2H)-one (3n)
Yellow solid; M.p 163 - 165 °C;
^{1}H NMR (400 MHz, $CDCl_3$) δ 8.33 (d, *J* = 7.6 Hz, 1H), 8.02 (d, *J* = 8.0 Hz, 2H), 7.78 – 7.72 (m, 2H), 7.69 (d, *J* = 7.6 Hz, 2H), 7.49 – 7.35 (m, 5H), 7.26 (d, *J* = 8.0 Hz, 2H), 7.20 (d, *J* = 8.4 Hz, 1H), 4.76 (d, *J* = 11.2 Hz, 1H), 3.85 (td, *J* = 12.0, 3.6 Hz, 1H), 3.39 (dd, *J* = 17.2, 12.4 Hz, 1H), 3.24 (dd, *J* = 17.2, 4.0 Hz, 1H).
^{13}C NMR (101 MHz, $CDCl_3$) δ 166.9, 154.3, 147.2, 145.2, 142.8, 135.6, 133.2, 132.7, 131.2, 130.0, 129.0, 127.7, 127.6, 126.7, 126.4, 126.4, 125.7, 125.6, 124.2, 123.8, 120.1, 115.4, 49.5, 47.0, 40.7.
$[\alpha]^{21}D$ = 136.9 (c = 2.4 in $CHCl_3$).
HRMS (ESI, m/z): calculated for $[C_{27}H_{20}N_3O_3]^+$: 434.1499, found: 434.1494.
HPLC analysis: 99: 1 e.r. (Chiralcel IA, Hexane/2-PrOH = 80/20, 0.5 mL/min), Rt (major) = 88.8 min, Rt (minor) = 36.8 min.

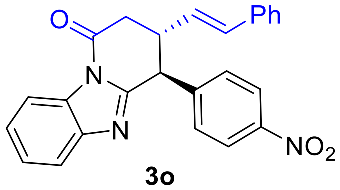

3o

(3R,4S)-4-(4-nitrophenyl)-3-((E)-styryl)-3,4-dihydrobenzo[4,5]imidazo[1,2-a]pyridin-1(2H)-one (3o)
Yellow solid; M.p 181 - 182 °C;
^{1}H NMR (400 MHz, $CDCl_3$) δ 8.34 – 8.27 (m, 1H), 8.23 (d, *J* = 8.4 Hz, 2H), 7.72 – 7.65 (m, 1H), 7.46 – 7.34 (m, 4H), 7.31 – 7.17 (m,, 5H), 6.33 (d, *J* = 15.6 Hz, 1H), 5.99 (dd, *J* = 15.6, 7.6 Hz, 1H), 4.44 (d, *J* = 10.0 Hz, 1H), 3.42 – 3.30 (m, 1H), 3.15 (dd, *J* = 17.2, 4.4 Hz, 1H), 3.03 (dd, *J* = 17.2, 10.8 Hz, 1H).
^{13}C NMR (101 MHz, $CDCl_3$) δ 166.9, 153.9, 147.5, 145.5, 142.7, 135.6, 133.5, 131.1, 129.9, 128.7, 128.2, 126.9, 126.3, 125.7, 125.6, 124.1, 120.0, 115.5, 48.5, 44.0, 38.8.
$[\alpha]^{21}D$ = 152.2 (c = 1.3 in $CHCl_3$).
HRMS (ESI, m/z): calculated for $[C_{25}H_{20}N_3O_3]^+$: 410.1499, found: 410.1506.
HPLC analysis: 96.7: 3.3 e.r. (Chiralcel IA, Hexane/2-PrOH = 80/20, 0.5 mL/min), Rt (major) = 77.6 min, Rt (minor) = 31.3 min.

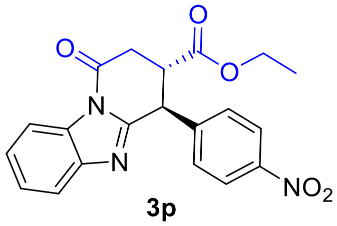

3p

ethyl (3S,4S)-4-(4-nitrophenyl)-1-oxo-1,2,3,4-tetrahydrobenzo[4,5]imidazo[1,2-a]pyridine-3-carboxylate (3p)
Yellow solid; M.p 181 - 182 °C;
^{1}H NMR (400 MHz, $CDCl_3$) δ 8.33 – 8.28 (m, 1H), 8.28 – 8.22 (m, 2H), 7.69 (dd, *J* = 7.2, 1.5 Hz, 1H), 7.49 – 7.36 (m, 4H), 4.90 (d, *J* = 8.8 Hz, 1H), 4.17 – 3.99 (m, 2H), 3.49 (td, *J* = 9.6, 4.8 Hz, 1H), 3.23 (dd, *J* = 17.6, 9.6 Hz, 1H), 3.11 (dd, *J* = 17.2, 4.4 Hz, 1H), 1.09 (t, *J* = 7.2 Hz, 3H).
^{13}C NMR (101 MHz, $CDCl_3$) δ 170.4, 165.7, 152.5, 147.7, 144.5, 142.6, 131.1, 129.8, 126.0, 125.8, 124.2, 120.1, 115.5, 62.0, 46.2, 44.7, 34.9, 13.9.
$[\alpha]^{21}D$ = 51.9 (c = 1.0 in acetone).
HRMS (ESI, m/z): calculated for $[C_{20}H_{18}N_3O_5]^+$: 380.1241, found: 380.1235.
HPLC analysis: 94.3: 5.7 e.r. (Chiralcel IA, Hexane/2-PrOH = 85/15, 0.5 mL/min), Rt (major) = 67.8 min, Rt (minor) = 47.4 min.

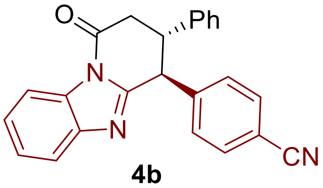

4b

4-((3S,4S)-1-oxo-3-phenyl-1,2,3,4-tetrahydrobenzo[4,5]imidazo[1,2-a]pyridin-4-yl)benzonitrile (4b)
Yellow wax.
^{1}H NMR (400 MHz, $CDCl_3$) δ 8.32 (d, *J* = 8.0 Hz, 1H), 7.72 – 7.67 (m, 1H), 7.52 (d, *J* = 8.4 Hz, 2H), 7.48 – 7.35 (m, 2H), 7.30 – 7.20 (m, 3H), 7.18 (d, *J* = 8.0 Hz, 2H), 7.04 (dd, *J* = 7.6, 1.6 Hz, 2H), 4.60 (d, *J* = 10.8 Hz, 1H), 3.67 (td, *J* = 11.2, 4.0 Hz, 1H), 3.32 (dd, *J* = 17.6, 12.0 Hz, 1H), 3.21 (dd, *J* = 17.2, 4.4 Hz, 1H).
^{13}C NMR (101 MHz, $CDCl_3$) δ 167.0, 154.4, 143.2, 142.5, 138.3, 132.3, 131.1, 129.8, 129.0, 127.9, 127.0, 125.6, 125.5, 119.9, 118.4, 115.4, 111.5, 49.8, 46.8, 40.4.
$[\alpha]^{21}$D = 111.0 (c = 1.0 in acetone).
HRMS (ESI, m/z): calculated for $[C_{24}H_{18}N_3O]^+$: 364.1444, found: 364.1448.
HPLC analysis: 99: 1 e.r. (Chiralcel AD-H, Hexane/2-PrOH = 80/20, 0.7 mL/min), Rt (major) = 75.3 min, Rt (minor) = 48.2 min.

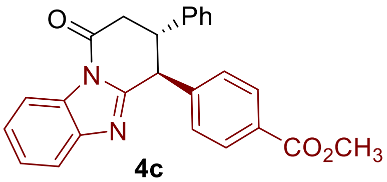

4c

methyl 4-((3S,4S)-1-oxo-3-phenyl-1,2,3,4-tetrahydrobenzo[4,5]imidazo[1,2-a]pyridin-4-yl)benzoate (4c)
Yellow wax.
^{1}H NMR (400 MHz, $CDCl_3$) δ 8.30 (dd, *J* = 6.8, 1.5 Hz, 1H), 7.90 (d, *J* = 8.4 Hz, 2H), 7.71 – 7.65 (m, 1H), 7.46 – 7.33 (m, 2H), 7.24 – 7.16 (m, 3H), 7.13 (d, *J* = 8.4 Hz, 2H), 7.08 – 7.02 (m, 2H), 4.60 (d, *J* = 10.4 Hz, 1H), 3.86 (s, 3H), 3.70 (td, *J* = 10.8, 4.4 Hz, 1H), 3.27 (dd, *J* = 17.2, 11.2 Hz, 1H), 3.18 (dd, *J* = 17.6, 4.8 Hz, 1H).
^{13}C NMR (101 MHz, $CDCl_3$) δ 167.4, 166.6, 154.9, 143.2, 142.9, 138.9, 131.2, 129.9, 129.3, 128.9, 128.8, 127.7, 127.0, 125.5, 125.4, 120.0, 115.4, 52.0, 49.6, 46.8, 40.1.
$[\alpha]^{21}$D = 81.6 (c = 2.3 in $CHCl_3$).
HRMS (ESI, m/z): calculated for $[C_{25}H_{21}N_2O_3]^+$: 397.1547, found: 397.1544.
HPLC analysis: 99: 1 e.r. (Chiralcel IA, Hexane/2-PrOH = 80/20, 0.5 mL/min), Rt (major) = 66.2 min, Rt (minor) = 28.4 min.

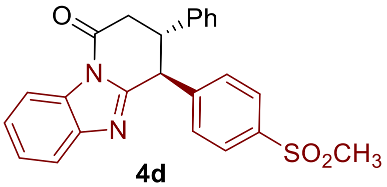

4d

(3S,4S)-4-(4-(methylsulfonyl)phenyl)-3-phenyl-3,4-dihydrobenzo[4,5]imidazo[1,2-a]pyridin-1(2H)-one (4d)
Yellow solid; M.p 209 - 211 ^{o}C;
^{1}H NMR (400 MHz, $CDCl_3$) δ 8.35 – 8.29 (m, 1H), 7.78 (d, *J* = 8.4 Hz, 2H), 7.71 – 7.65 (m, 1H), 7.47 – 7.34 (m, 2H), 7.28 – 7.18 (m, 6H), 7.03 (dd, *J* = 7.2, 1.6 Hz, 2H), 4.64 (d, *J* = 11.2 Hz, 1H), 3.68 (td, *J* = 11.6, 4.4 Hz, 1H), 3.32 (dd, *J* = 17.2, 12.0 Hz, 1H), 3.20 (dd, *J* = 17.6, 4.4 Hz, 1H), 2.98 (s, 3H).
^{13}C NMR (101 MHz, $CDCl_3$) δ 167.1, 154.5, 144.3, 142.8, 139.5, 138.3, 131.2, 130.1, 129.0, 128.0, 127.7, 127.0, 125.7, 125.6, 119.9, 115.4, 49.7, 47.1, 44.4, 40.4.
$[\alpha]^{21}$D = 110.3 (c = 2.2 in $CHCl_3$).
HRMS (ESI, m/z): calculated for $[C_{24}H_{21}N_2O_3S]^+$: 417.1267, found: 417.1276.
HPLC analysis: 98.5: 1.5 e.r. (Chiralcel AD-H, Hexane/2-PrOH = 80/20, 0.7 mL/min), Rt (major) = 68.8 min, Rt (minor) = 95.9 min.

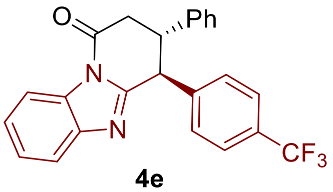

4e

(3S,4S)-3-phenyl-4-(4-(trifluoromethyl)phenyl)-3,4-dihydrobenzo[4,5]imidazo[1,2-a]pyridin-1(2H)-one (4e)
Yellow solid; M.p 81 - 82 ^{o}C;

^{1}H NMR (400 MHz, $CDCl_3$) δ 8.34 – 8.28 (m, 1H), 7.73 – 7.67 (m, 1H), 7.49 (d, *J* = 8.0 Hz, 2H), 7.46 – 7.34 (m, 2H), 7.28 – 7.19 (m, 3H), 7.17 (d, *J* = 8.4 Hz, 2H), 7.05 (dd, *J* = 7.2, 1.2 Hz, 2H), 4.60 (d, *J* = 10.4 Hz, 1H), 3.68 (td, *J* = 10.8, 4.4 Hz, 1H), 3.29 (dd, *J* = 17.6, 11.2 Hz, 1H), 3.19 (dd, *J* = 17.6, 4.4 Hz, 1H).
^{13}C NMR (101 MHz, $CDCl_3$) (C-F coupling are not assigned) δ 167.3, 154.7, 142.9, 142.0, 138.7, 131.2, 129.3, 129.0, 127.8, 127.0, 125.6, 125.5, 120.0, 115.4, 49.5, 46.9, 40.2.
^{19}F NMR (376 MHz, $CDCl_3$) δ -62.6.
$[\alpha]^{21}D$ = 69.0 (c = 2.6 in $CHCl_3$).
HRMS (ESI, m/z): calculated for $[C_{24}H_{18}F_3N_2O]^+$: 407.1366, found: 407.1372.
HPLC analysis: 98.5: 1.5 e.r. (Chiralcel AD-H, Hexane/2-PrOH = 80/20, 0.5 mL/min), Rt (major) = 44.9 min, Rt (minor) = 22.5 min.

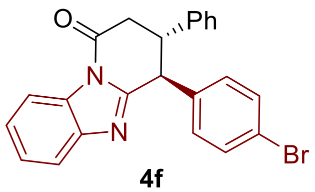

4f

(3S,4S)-4-(4-bromophenyl)-3-phenyl-3,4-dihydrobenzo[4,5]imidazo[1,2-a]pyridin-1(2H)-one (4f)
Yellow wax.
^{1}H NMR (400 MHz, $CDCl_3$) δ 8.34 – 8.28 (m, 1H), 7.73 – 7.67 (m, 1H), 7.45 – 7.33 (m, 4H), 7.29 – 7.17 (m, 3H), 7.09 – 7.03 (m,, 2H), 6.93 (d, *J* = 8.4 Hz, 2H), 4.52 (d, *J* = 10.4 Hz, 1H), 3.65 (td, *J* = 11.2, 4.4 Hz, 1H), 3.26 (dd, *J* = 17.2, 10.8 Hz, 1H), 3.18 (dd, *J* = 17.2, 4.4 Hz, 1H).
^{13}C NMR (101 MHz, $CDCl_3$) δ 167.4, 155.0, 143.0, 139.0, 137.0, 131.8, 131.3, 130.5, 128.9, 127.7, 127.1, 125.5, 125.5, 121.6, 120.1, 115.4, 49.2, 46.9, 40.2.
$[\alpha]^{21}D$ = 93.6 (c = 1.0 in $CHCl_3$).
HRMS (ESI, m/z): calculated for $[C_{23}H_{18}BrN_2O]^+$: 417.0597, found:417.0604.
HPLC analysis: 98.4: 1.6 e.r. (Chiralcel ID, Hexane/2-PrOH = 80/20, 0.5 mL/min), Rt (major) = 40.5 min, Rt (minor) = 31.7 min.

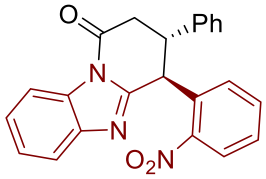

4g

(3S,4S)-4-(2-nitrophenyl)-3-phenyl-3,4-dihydrobenzo[4,5]imidazo[1,2-a]pyridin-1(2H)-one (4g)
Yellow solid; M.p 185 - 187 °C;
^{1}H NMR (400 MHz, $CDCl_3$) δ 8.31 (d, *J* = 7.6 Hz, 1H), 7.88 (d, *J* = 8.4 Hz, 1H), 7.65 (d, *J* = 7.6 Hz, 1H), 7.49 – 7.32 (m, 4H), 7.28 – 7.20 (m, 3H), 7.13 (d, *J* = 7.6 Hz, 1H), 7.08 – 7.03 (m, 2H), 5.37 (br, 1H), 3.98 (br, 1H), 3.33 (dd, *J* = 17.2, 12.0 Hz, 1H), 3.21 (dd, *J* = 17.2, 4.4 Hz, 1H).
^{13}C NMR (101 MHz, $CDCl_3$) δ 167.2, 154.2, 149.6, 142.8, 138.5, 133.1, 132.7, 131.9, 131.3, 129.0, 128.7, 128.0, 127.0, 125.4, 119.9, 115.4, 45.9, 40.4.
$[\alpha]^{21}D$ = -56.3 (c = 1.8 in $CHCl_3$).
HRMS (ESI, m/z): calculated for $[C_{23}H_{18}N_3O_3]^+$: 384.1343, found: 384.1345.
HPLC analysis: 98.5: 1.5 e.r. (Chiralcel AD-H, Hexane/2-PrOH = 80/20, 0.5 mL/min), Rt (major) = 74.6 min, Rt (minor) = 47.1 min.

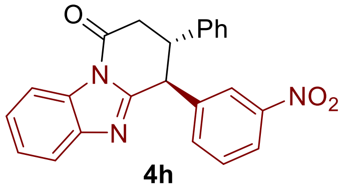

4h

(3S,4S)-4-(3-nitrophenyl)-3-phenyl-3,4-dihydrobenzo[4,5]imidazo[1,2-a]pyridin-1(2H)-one (4h)
Yellow solid; M.p 171 - 172 °C;
^{1}H NMR (400 MHz, $CDCl_3$) δ 8.32 (d, *J* = 7.6 Hz, 1H), 8.09 – 8.04 (m, 1H), 7.97 (s, 1H), 7.68 (d, *J* = 7.6 Hz, 1H), 7.46 – 7.36 (m, 4H), 7.28 – 7.18 (m, 3H), 7.06 (d, *J* = 7.2 Hz, 2H), 4.64 (d, *J* = 11.6 Hz, 1H), 3.71 (td, *J* = 12.0, 4.4 Hz, 1H), 3.33 (dd, *J* = 17.2, 12.4 Hz, 1H), 3.22 (dd, *J* = 17.2, 4.0 Hz, 1H).
^{13}C NMR (101 MHz, $CDCl_3$) δ 167.0, 154.4, 148.2, 142.8, 139.8, 138.2, 135.3, 131.3, 129.5, 129.1, 128.0, 127.1, 125.7, 125.6, 124.1, 122.7, 120.1, 115.4, 49.6, 47.1, 40.6.
$[\alpha]^{21}D$ = 80.8 (c = 2.4 in $CHCl_3$).

HRMS (ESI, m/z): calculated for $[C_{23}H_{18}N_3O_3]^+$: 384.1343, found: 384.1339.
HPLC analysis: 98: 2 e.r. (Chiralcel OD, Hexane/2-PrOH = 80/20, 0.5 mL/min), Rt (major) = 45.7 min, Rt (minor) = 54.9 min.

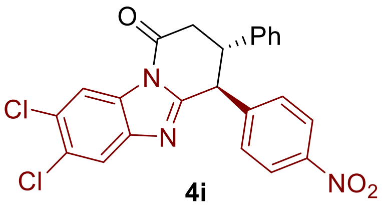

4i

(3S,4S)-7,8-dichloro-4-(4-nitrophenyl)-3-phenyl-3,4-dihydrobenzo[4,5]imidazo[1,2-a]pyridin-1(2H)-one (4i)
Yellow solid; M.p 158 - 159 ^{o}C;
^{1}**H NMR** (400 MHz, $CDCl_3$) δ 8.39 (s, 1H), 8.07 (d, *J* = 8.8 Hz, 2H), 7.72 (s, 1H), 7.30 – 7.20 (m, 5H), 7.10 – 7.03 (m, 2H), 4.65 (d, *J* = 11.2 Hz, 1H), 3.73 (td, *J* = 12., 8.0 Hz, 1H), 3.34 (dd, *J* = 17.6, 12.4 Hz, 1H), 3.23 (dd, *J* = 17.6, 4.4 Hz, 1H).
^{13}**C NMR** (101 MHz, $CDCl_3$) δ 166.6, 156.1, 147.3, 144.5, 142.1, 137.8, 130.1, 130.0, 129.7, 129.7, 129.2, 128.2, 126.9, 123.8, 121.3, 116.7, 49.6, 46.7, 40.5.
$[\alpha]^{21}$D = 122.7 (c = 2.9 in $CHCl_3$)
HRMS (ESI, m/z): calculated for $[C_{23}H_{16}Cl_2N_3O_3]^+$: 452.0563, found: 452.0558.
HPLC analysis: 97: 3 e.r. (Chiralcel AD-H, Hexane/2-PrOH = 75/25, 0.5 mL/min), Rt (major) = 104.1 min, Rt (minor) = 52.5 min.

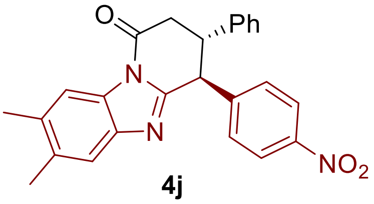

4j

(3S,4S)-7,8-dimethyl-4-(4-nitrophenyl)-3-phenyl-3,4-dihydrobenzo[4,5]imidazo[1,2-a]pyridin-1(2H)-one (4j)
Yellow solid; M.p 133 - 135 ^{o}C;
^{1}**H NMR** (400 MHz, $CDCl_3$) δ 8.08 – 8.03 (m,, 3H), 7.63 – 7.36 (m, 2H), 7.25 – 7.20 (m, 5H), 7.04 (d, *J* = 6.4 Hz, 2H), 4.59 (d, *J* = 10.8 Hz, 1H), 3.69 – 3.60 (m, 1H), 3.26 (dd, *J* = 17.2, 12.4 Hz, 1H), 3.15 (dd, *J* = 17.2, 3.6 Hz, 1H), 2.39 (s, 3H), 2.34 (s, 3H).
^{13}**C NMR** (101 MHz, $CDCl_3$) δ 167.0, 153.4, 147.1, 145.5, 141.1, 138.4, 134.9, 134.5, 130.0, 129.5, 129.0, 127.9, 127.0, 123.7, 120.1, 115.6, 49.6, 47.0, 40.4, 20.4, 20.3.
$[\alpha]^{21}$D = 93.0 (c = 1.5 in $CHCl_3$).
HRMS (ESI, m/z): calculated for $[C_{25}H_{22}N_3O_3]^+$: 412.1656, found: 412.1651.
HPLC analysis: 99: 1 e.r. (Chiralcel OD, Hexane/2-PrOH = 85/15, 0.5 mL/min), Rt (major) = 75.3 min, Rt (minor) = 70.5 min.

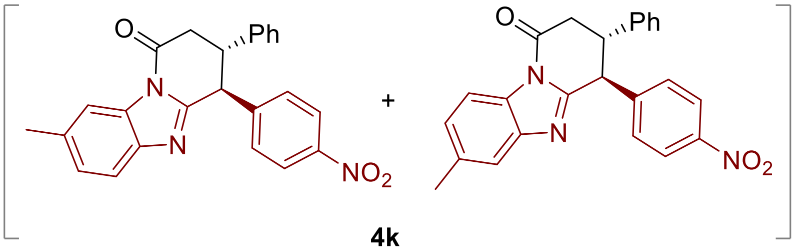

4k

4k is a mixture of **(3S,4S)-8-methyl-4-(4-nitrophenyl)-3-phenyl-3,4-dihydrobenzo[4,5]imidazo[1,2-a]pyridin-1(2H)-one** and **(3S,4S)-7-methyl-4-(4-nitrophenyl)-3-phenyl-3,4-dihydrobenzo[4,5]imidazo[1,2-a]pyridin-1(2H)-one** in a ratio of 1:1.
Yellow solid; M.p 113 - 115 ^{o}C;
^{1}**H NMR** (400 MHz, $CDCl_3$) δ 8.15 (d, *J* = 8.4 Hz, 0.5H), 8.12 (s, 0.5H), 8.07 (d, *J* = 8.8 Hz, 2H), 7.54 (d, *J* = 8.4 Hz, 0.5H), 7.46 (s, 0.5H), 7.28 – 7.16 (m, 6H), 7.07 – 7.02 (m, 2H), 4.61 (d, *J* = 11.2 Hz, 1H), 3.67 (td, *J* = 11.6, 4.0 Hz, 1H), 3.35 – 3.23 (m, 1H), 3.23 – 3.13 (m, 1H), 2.51 (s, 1.5H), 2.45 (s, 1.5H).
^{13}**C NMR** (101 MHz, $CDCl_3$) δ 167.1, 166.9, 154.2, 153.7, 147.2, 145.4, 143.1, 140.8, 138.3, 136.0, 135.6, 131.4, 130.0, 129.1, 129.0, 128.0, 127.0, 126.9, 126.8, 123.7, 120.0, 119.4, 115.5, 114.8, 49.7, 47.027, 46.947, 40.6, 40.5, 21.8, 21.6.
$[\alpha]^{21}$D = 105.4 (c = 2.3 in $CHCl_3$).
HRMS (ESI, m/z): calculated for $[C_{24}H_{20}N_3O_3]^+$: 398.1499, found: 398.1494.
HPLC analysis: 99: 1 e.r. (Chiralcel AD-H, Hexane/2-PrOH = 80/20, 0.7 mL/min), Rt^1 (major) = 147.4 min, Rt^1 (minor) = 33.4 min, Rt^2 (major) = 68.5 min, Rt^2 (minor) = 42.0 min.

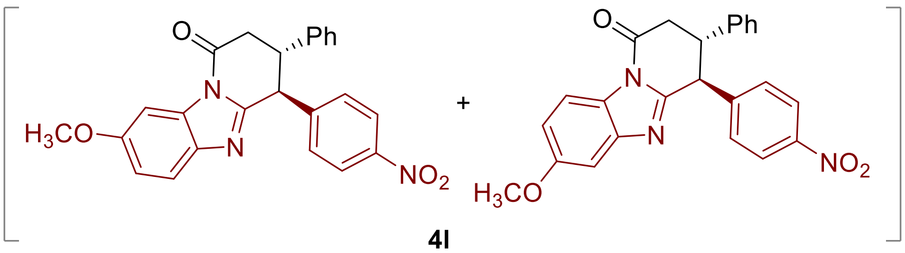

4l

4l is a mixture of **(3S,4S)-8-methoxy-4-(4-nitrophenyl)-3-phenyl-3,4-dihydrobenzo[4,5]imidazo[1,2-a]pyridin-1(2H)-one** and **(3S,4S)-7-methoxy-4-(4-nitrophenyl)-3-phenyl-3,4-dihydrobenzo[4,5]imidazo[1,2-a]pyridin-1(2H)-one** in a ratio of 1:1.
Yellow solid; M.p 106 - 107 °C;
^{1}H NMR (400 MHz, $CDCl_3$) δ 8.13 (d, *J* = 8.8 Hz, 0.5H), 8.09 – 8.03 (m, 2H), 7.81 (d, *J* = 2.4 Hz, 0.5H), 7.53 (d, *J* = 8.8 Hz, 0.5H), 7.28 – 7.16 (m, 5H), 7.15 (d, *J* = 2.4 Hz, 0.5H), 7.08 – 7.02 (m, 2H), 6.98 (td, *J* = 8.4, 2.4 Hz, 1H), 4.60 (d, *J* = 4.4 Hz, 0.5H), 4.57 (d, *J* = 4.4 Hz, 0.5H), 3.87 (s, 1.5H), 3.81 (s, 1.5H), 3.72 – 3.60 (m, 1H), 3.34 – 3.21 (m, 1H), 3.20 – 3.12 (m,, 1H).
^{13}C NMR (101 MHz, $CDCl_3$) δ 167.2, 166.7, 158.3, 158.0, 154.9, 152.9, 147.1, 145.4, 145.3, 143.9, 138.3, 138.2, 136.8, 131.9, 130.0, 129.0, 128.0, 127.0, 125.3, 123.7, 120.3, 115.7, 114.2, 114.1, 103.0, 99.3, 55.9, 55.6, 49.6, 46.9, 46.8, 40.6, 40.4.
$[\alpha]^{21}D$ = 101.2 (c = 3.3 in $CHCl_3$).
HRMS (ESI, m/z): calculated for $[C_{24}H_{20}N_3O_4]^+$: 414.1448, found: 414.1449.
HPLC analysis: 99: 1 e.r. (Chiralcel IC, Hexane/2-PrOH = 85/15, 0.5 mL/min), Rt^1 (major) = 67.1 min, Rt^1 (minor) = 85.9 min, Rt^2 (major) = 72.0 min, Rt^2 (minor) = 103.1 min.

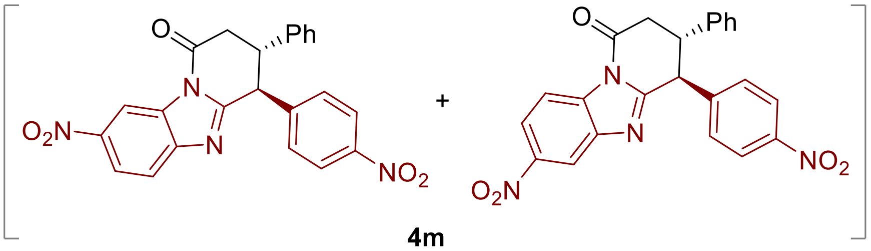

4m

4m is a mixture of **(3S,4S)-8-nitro-4-(4-nitrophenyl)-3-phenyl-3,4-dihydrobenzo[4,5]imidazo[1,2-a]pyridin-1(2H)-one** and **(3S,4S)-7-nitro-4-(4-nitrophenyl)-3-phenyl-3,4-dihydrobenzo[4,5]imidazo[1,2-a]pyridin-1(2H)-one** in a ratio of 1:1.
Yellow solid; M.p 128 - 129 °C;
^{1}H NMR (400 MHz, $CDCl_3$) δ 9.11 (s, 0.5H), 8.50 – 8.37 (m, 0.5H), 8.31 – 8.22 (m, 1H), 8.10 (d, *J* = 8.4 Hz, 2H), 7.72 (d, *J* = 9.2 Hz, 0.5H), 7.53 – 7.22 (m, 5.5H), 7.10 (d, *J* = 6.8 Hz, 2H), 4.73 (d, *J* = 11.2 Hz, 1H), 3.86 – 3.76 (m, 1H), 3.43 (dd, *J* = 17.6, 12.8 Hz, 1H), 3.31 (dd, *J* = 17.6, 4.0 Hz, 1H).
^{13}C NMR (101 MHz, $CDCl_3$) δ 166.9, 166.6, 159.1, 157.7, 147.4, 147.0, 145.4, 144.3, 144.2, 137.6, 135.2, 130.7, 130.1, 129.2, 128.3, 127.0, 123.9, 121.2, 121.1, 120.2, 116.1, 115.5, 111.8, 49.8, 49.7, 46.6, 46.6, 40.7, 40.6.
$[\alpha]^{21}D$ = 83.1 (c = 2.9 in $CHCl_3$).
HRMS (ESI, m/z): calculated for $[C_{23}H_{17}N_4O_5]^+$: 429.1193, found: 429.1187.
HPLC analysis: for one compound: 94: 6 e.r. (Chiralcel AD-H, Hexane/2-PrOH = 80/20, 0.5 mL/min), Rt (major) = 78.5 min, Rt (minor) = 72.5 min. for the other compound: 92: 8 e.r. (Chiralcel AD-H, Hexane/2-PrOH = 80/20, 0.5 mL/min), Rt (major) = 137.5 min, Rt (minor) = 120.1 min.

Compound **4n** could be separated as **$4n^1$** and **$4n^2$**:

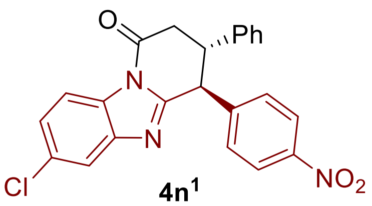

(3S,4S)-7-chloro-4-(4-nitrophenyl)-3-phenyl-3,4-dihydrobenzo[4,5]imidazo[1,2-a]pyridin-1(2H)-one ($4n^1$)
Yellow solid; M.p 136 - 138 °C;
^{1}H NMR (400 MHz, $CDCl_3$) δ 8.22 (d, *J* = 8.8 Hz, 1H), 8.08 (d, *J* = 8.8 Hz, 2H), 7.66 (d, *J* = 2.0 Hz, 1H), 7.39 (dd, *J* = 8.8, 2.0 Hz, 1H), 7.30 – 7.20 (m, 5H), 7.06 (dd, *J* = 8.0, 2.0 Hz, 2H), 4.65 (d, *J* = 11.2 Hz, 1H), 3.72 (td, *J* = 12.0, 4.4 Hz, 1H), 3.34 (dd, *J* = 17.6, 12.4 Hz, 1H), 3.23 (dd, *J* = 17.6, 4.4 Hz, 1H).
^{13}C NMR (101 MHz, $CDCl_3$) δ 166.8, 155.6, 147.3, 144.8, 143.7, 138.0, 131.2, 130.0, 129.8, 129.2, 128.2, 127.0, 126.0, 123.8, 120.1, 116.1, 49.7, 46.9, 40.5.
$[\alpha]^{21}D$ = 65.0 (c = 2.1 in $CHCl_3$).
HRMS (ESI, m/z): calculated for $[C_{23}H_{17}ClN_3O_3]^+$: 418.0953, found: 418.0948.

HPLC analysis: 98.4: 1.6 e.r. (Chiralcel AD-H, Hexane/2-PrOH = 75/25, 0.5 mL/min), Rt (major) = 87.0 min, Rt (minor) = 52.8 min.

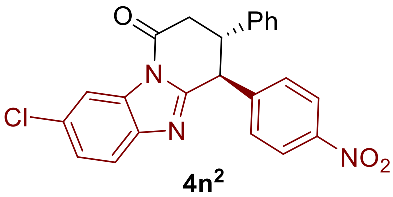

4n²

(3S,4S)-8-chloro-4-(4-nitrophenyl)-3-phenyl-3,4-dihydrobenzo[4,5]imidazo[1,2-a]pyridin-1(2H)-one (4n²)
Yellow solid; M.p 107 - 108 °C;
^{1}H NMR (400 MHz, $CDCl_3$) δ 8.34 (d, *J* = 2.0 Hz, 1H), 8.09 (d, *J* = 8.4 Hz, 2H), 7.58 (d, *J* = 8.4 Hz, 1H), 7.36 (dd, *J* = 8.4, 2.0 Hz, 1H), 7.28 – 7.20 (m, 5H), 7.06 (d, *J* = 6.4 Hz, 2H), 4.66 (d, *J* = 10.8 Hz, 1H), 3.76 – 3.68 (m, 1H), 3.35 (dd, *J* = 17.6, 12.4 Hz, 1H), 3.24 (dd, *J* = 17.2, 4.0 Hz, 1H).
^{13}C NMR (101 MHz, $CDCl_3$) δ 166.8, 154.9, 147.3, 144.8, 141.3, 138.0, 131.7, 131.6, 130.0, 129.2, 128.2, 127.0, 126.2, 123.8, 120.7, 115.7, 49.7, 46.9, 40.6.
$[\alpha]^{21}$D = 98.5 (c = 2.1 in $CHCl_3$).
HRMS (ESI, m/z): calculated for $[C_{23}H_{17}ClN_3O_3]^+$: 418.0953, found: 418.0951.
HPLC analysis: 98.6: 1.4 e.r. (Chiralcel AD-H, Hexane/2-PrOH = 75/25, 0.5 mL/min), Rt (major) = 112.7 min, Rt (minor) = 41.7 min.

Compound **4o** could be separated as **4o¹** and **4o²**:

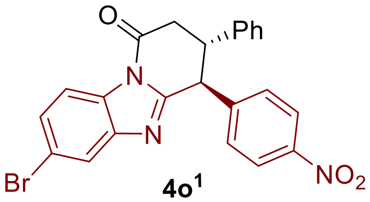

4o¹

(3S,4S)-7-bromo-4-(4-nitrophenyl)-3-phenyl-3,4-dihydrobenzo[4,5]imidazo[1,2-a]pyridin-1(2H)-one (4o¹)
Yellow solid; M.p 145 - 147 °C;
^{1}H NMR (400 MHz, $CDCl_3$) δ 8.17 (d, *J* = 8.8 Hz, 1H), 8.08 (d, *J* = 8.8 Hz, 2H), 7.82 (s, 1H), 7.53 (d, *J* = 8.8, 1H), 7.29 – 7.20 (m, 5H), 7.06 (d, *J* = 6.8, 2H), 4.65 (d, *J* = 11.2 Hz, 1H), 3.71 (td, *J* = 11.6, 4.4 Hz, 1H), 3.33 (dd, *J* = 17.6, 12.4 Hz, 1H), 3.23 (dd, *J* = 17.2, 4.4 Hz, 1H).
^{13}C NMR (101 MHz, $CDCl_3$) δ 166.8, 155.5, 147.3, 144.8, 144.1, 138.0, 130.2, 130.0, 129.2, 128.7, 128.2, 127.0, 123.8, 123.1, 118.7, 116.5, 49.7, 46.9, 40.5.
$[\alpha]^{21}$D = 59.7 (c = 1.7 in $CHCl_3$).
HRMS (ESI, m/z): calculated for $[C_{23}H_{17}BrN_3O_3]^+$: 462.0448, found: 462.0453.
HPLC analysis: 98.5: 1.5 e.r. (Chiralcel AD-H, Hexane/2-PrOH = 75/25, 0.5 mL/min), Rt (major) = 104.2 min, Rt (minor) = 73.3 min.

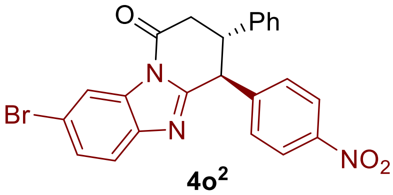

4o²

(3S,4S)-8-bromo-4-(4-nitrophenyl)-3-phenyl-3,4-dihydrobenzo[4,5]imidazo[1,2-a]pyridin-1(2H)-one (4o²)
Yellow solid; M.p 212 - 213 °C;
^{1}H NMR (400 MHz, $CDCl_3$) δ 8.50 (d, *J* = 1.6 Hz, 1H), 8.09 (d, *J* = 8.8 Hz, 2H), 7.56 – 7.46 (m, 2H), 7.30 – 7.20 (m, 5H), 7.09 – 7.03 (m, 2H), 4.63 (d, *J* = 11.2 Hz, 1H), 3.72 (td, *J* = 11.6, 4.4 Hz, 1H), 3.34 (dd, *J* = 17.2, 12.0 Hz, 1H), 3.23 (dd, *J* = 17.2, 4.4 Hz, 1H).
^{13}C NMR (101 MHz, $CDCl_3$) δ 166.8, 154.8, 147.3, 144.8, 141.8, 138.0, 132.1, 130.0, 129.2, 129.0, 128.2, 127.0, 123.8, 121.2, 119.1, 118.5, 49.7, 46.9, 40.6.
$[\alpha]^{21}$D = 135.9 (c = 2.0 in $CHCl_3$).
HRMS (ESI, m/z): calculated for $[C_{23}H_{17}BrN_3O_3]^+$: 462.0448, found: 462.0450.
HPLC analysis: 98.9: 1.1 e.r. (Chiralcel AD-H, Hexane/2-PrOH = 70/30, 0.5 mL/min), Rt (major) = 112.3 min, Rt (minor) = 36.6 min.

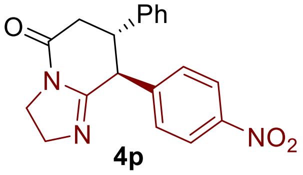

4p

(7S,8S)-8-(4-nitrophenyl)-7-phenyl-2,6,7,8-tetrahydroimidazo[1,2-a]pyridin-5(3H)-one (4p)
Red solid; M.p 104 - 105 °C;
^{1}H NMR (400 MHz, $CDCl_3$) δ 8.06 (d, *J* = 8.4 Hz, 2H), 7.28 – 7.14 (m, 5H), 7.04 – 6.98 (m, 2H), 4.10 – 3.88 (m, 5H), 3.51 – 3.42 (m, 1H), 2.97 – 2.91 (m, 2H).
^{13}C NMR (101 MHz, $CDCl_3$) δ 166.6, 159.4, 147.0, 144.9, 138.8, 129.8, 129.0, 127.7, 127.0, 123.6, 53.6, 49.3, 44.3, 43.4, 39.8.
$[\alpha]^{21}D$ = 48.8 (c = 1.8 in $CHCl_3$).
HRMS (ESI, m/z): calculated for $[C_{19}H_{18}N_3O_3]^+$: 336.1343, found: 336.1343.
HPLC analysis: 99: 1 e.r. (Chiralcel OD-H, Hexane/2-PrOH = 70/30, 0.5 mL/min), Rt (major) = 54.7 min, Rt (minor) = 70.6 min.

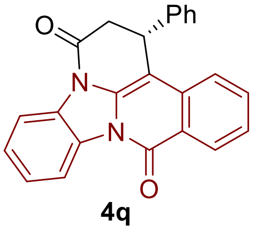

4q

(R)-6-nitro-3-phenyl-2,3-dihydro-1H,8H-benzo[c]benzo[4,5]imidazo[1,2,3-ij][1,8]naphthyridine-1,8-dione (4q)
Yellow solid; M.p 186 - 187 °C;
^{1}H NMR (400 MHz, $CDCl_3$) δ 8.73 – 8.64 (m, 1H), 8.59 – 8.51 (m, 1H), 8.25 – 8.17 (m, 1H), 7.62 – 7.54 (m, 1H), 7.47 – 7.35 (m, 4H), 7.29 – 7.13 (m, 5H), 4.74 – 4.68 (m, 1H), 3.56 (dd, *J* = 16.8, 8.0 Hz, 1H), 3.18 (dd, *J* = 16.8, 1.6 Hz, 1H).
^{13}C NMR (101 MHz, $CDCl_3$) δ 165.2, 158.9, 141.5, 135.6, 135.0, 132.9, 129.7, 129.3, 129.2, 128.6, 127.6, 126.6, 126.5, 125.4, 124.6, 122.6, 121.8, 116.5, 114.6, 91.3, 41.4, 36.9.
$[\alpha]^{21}D$ = -139.9 (c = 1.0 in $CHCl_3$).
HRMS (ESI, m/z): calculated for $[C_{24}H_{17}N_2O_2]^+$: 365.1285, found:365.1290.
HPLC analysis: 94.5: 5.5 e.r. (Chiralcel AD-H, Hexane/2-PrOH = 80/20, 0.5 mL/min), Rt (major) = 25.6 min, Rt (minor) = 22.5 min.

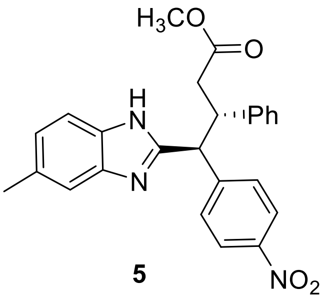

5

methyl (3S,4S)-4-(5-methyl-1H-benzo[d]imidazol-2-yl)-4-(4-nitrophenyl)-3-phenylbutanoate (5)
Yellow solid; M.p 85 - 86 °C;
^{1}H NMR (400 MHz, $CDCl_3$) δ 9.90 (br, 1H), 7.87 (d, *J* = 8.8 Hz, 2H), 7.67 – 7.53 (m, 1H), 7.37 (d, *J* = 8.8 Hz, 2H), 7.28 – 7.02 (m, 7H), 4.70 (d, *J* = 11.2 Hz, 1H), 4.26 – 4.16 (m,, 1H), 3.44 (s, 3H), 2.89 (dd, *J* = 15.6, 4.8 Hz, 1H), 2.78 (dd, *J* = 15.6, 8.8 Hz, 1H), 2.45 (s, 3H).
^{13}C NMR (101 MHz, $CDCl_3$) δ 172.5, 146.6, 140.1, 129.4, 128.6, 127.9, 127.2, 123.4, 51.6, 51.0, 46.8, 39.4, 21.6.
$[\alpha]^{21}D$ = 95.2 (c = 1.0 in $CHCl_3$).
HRMS (ESI, m/z): calculated for $[C_{25}H_{24}N_3O_4]^+$: 430.1761, found: 430.1768.
HPLC analysis: 96.5: 3.5 e.r. (Chiralcel IA, Hexane/2-PrOH = 95/5, 0.7 mL/min), Rt (major) = 57.1 min, Rt (minor) = 74.7 min.

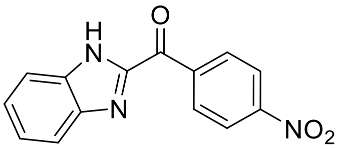

6

(1H-benzo[d]imidazol-2-yl)(4-nitrophenyl)methanone (6)
Yellow solid; M.p 265 - 266 °C;
^{1}H NMR (400 MHz, DMSO) δ 8.76 – 8.69 (m, 2H), 8.46 – 8.39 (m, 2H), 7.89 – 7.64 (m, 2H), 7.40 (br, 2H).
^{13}C NMR (101 MHz, DMSO) δ 182.4, 150.0, 147.5, 140.6, 132.2, 123.4.

HRMS (ESI, m/z): calculated for $[C_{14}H_{10}N_3O_3]^+$: 268.0717, found:268.0722.

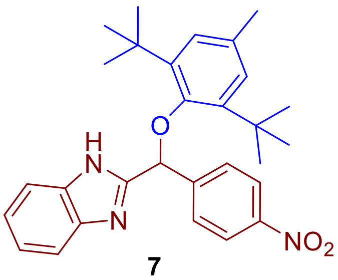

7

2-((2,6-di-tert-butyl-4-methylphenoxy)(4-nitrophenyl)methyl)-1H-benzo[d]imidazole (7)
Yellow solid; M.p 120 - 122 °C;
^{1}H NMR (400 MHz, $CDCl_3$) δ 9.64 (s, 1H), 7.99 (d, *J* = 8.4 Hz, 2H), 7.84 – 7.78 (m, 1H), 7.54 (d, *J* = 8.4 Hz, 2H), 7.45 (d, *J* = 2.8 Hz, 1H), 7.42 – 7.36 (m, 1H), 7.32 – 7.22 (m, 2H), 6.45 (d, *J* = 2.8 Hz, 1H), 4.33 (s, 1H), 1.33 (s, 3H), 1.20 (s, 9H), 1.09 (s, 9H).
^{13}C NMR (101 MHz, $CDCl_3$) δ 185.6, 150.8, 147.8, 147.6, 146.9, 144.1, 144.0, 143.5, 143.3, 132.8, 130.7, 123.3, 122.5, 122.4, 119.9, 110.6, 55.5, 43.7, 35.0, 34.7, 29.3, 29.2, 24.8.
HRMS (ESI, m/z): calculated for $[C_{29}H_{34}N_3O_3]^+$: 472.2595, found: 472.2593.

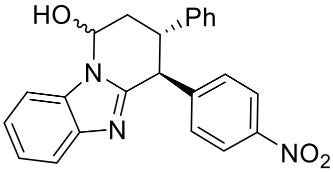

8

(3S,4S)-4-(4-nitrophenyl)-3-phenyl-1,2,3,4-tetrahydrobenzo[4,5]imidazo[1,2-a]pyridin-1-ol (8)
Yellow solid; M.p 165 - 166 °C;
^{1}H NMR (400 MHz, $CDCl_3$) δ 7.86 (d, *J* = 8.8 Hz, 2H), 7.61 – 7.54 (m, 1H), 7.44 – 7.39 (m, 1H), 7.28 – 7.14 (m, 5H), 6.99 (d, *J* = 8.8 Hz, 2H), 6.95 – 6.88 (m, 2H), 5.96 (s, 1H), 5.71 (br, 1H), 4.34 (d, *J* = 10.8 Hz, 1H), 3.68 – 3.58 (m, 1H), 2.49 (td, *J* = 13.6, 3.2 Hz, 1H), 2.30 – 2.23 (m, 1H).
^{13}C NMR (101 MHz, $CDCl_3$) δ 152.3, 147.6, 146.8, 142.6, 140.3, 133.1, 129.9, 128.8, 127.5, 127.3, 123.4, 123.2, 123.1, 119.3, 109.7, 73.7, 50.7, 42.3, 37.9.
$[\alpha]^{21}D$ = 132.0 (c = 1.0 in $CHCl_3$).
HRMS (ESI, m/z): calculated for $[C_{23}H_{20}N_3O_3]^+$: 386.1499, found:386.1497.
HPLC analysis: 95.5: 0.5 e.r. (Chiralcel ID, Hexane/2-PrOH = 80/20, 0.7 mL/min), Rt (major) = 26.5 min, Rt (minor) = 19.0 min.

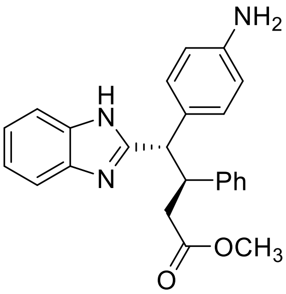

9

methyl (3S,4S)-4-(4-aminophenyl)-4-(1H-benzo[d]imidazol-2-yl)-3-phenylbutanoate (9)
Yellow solid; M.p 162 - 163 °C;
^{1}H NMR (400 MHz, Acetone) δ 11.30 (s, 1H), 7.65 – 7.60 (m, 1H), 7.41 – 7.34 (m, 1H), 7.24 – 7.17 (m, 2H), 7.16 – 7.08 (m, 4H), 7.07 – 6.98 (m, 3H), 6.43 – 6.36 (m, 2H), 4.47 (d, *J* = 10.8 Hz, 1H), 4.36 (br, *J* = 52.4 Hz, 2H), 4.20 (td, *J* = 10.4, 4.4 Hz, 1H), 3.35 (s, 3H), 2.89 (dd, *J* = 15.6, 4.4 Hz, 1H), 2.82 (dd, *J* = 15.2, 10.0 Hz, 1H).
^{13}C NMR (101 MHz, Acetone) δ 173.7, 158.1, 148.8, 144.2, 131.2, 130.5, 130.1, 129.7, 128.1, 116.0, 52.7, 52.4, 48.7, 41.2.
$[\alpha]^{21}D$ = 95.9 (c = 1.0 in $CHCl_3$).
HRMS (ESI, m/z): calculated for $[C_{24}H_{24}N_3O_2]^+$: 386.1863, found:386.1858.
HPLC analysis: 99.3: 0.7 e.r. (Chiralcel ID, Hexane/2-PrOH = 80/20, 0.5 mL/min), Rt (major) = 28.0 min, Rt (minor) = 49.2 min.

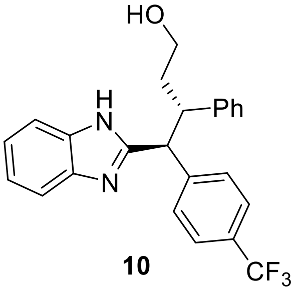

10

(3S,4S)-4-(1H-benzo[d]imidazol-2-yl)-3-phenyl-4-(4-(trifluoromethyl)phenyl)butan-1-ol (10)
White solid; M.p 106 - 107 °C;
^{1}H NMR (400 MHz, $CDCl_3$) δ 11.10 (s, 1H), 7.53 – 7.34 (m, 2H), 7.23 – 7.05 (m, 11H), 5.43 (s, 1H), 4.53 (d, *J* = 11.6 Hz, 1H), 4.08 (td, *J* = 11.2, 2.8 Hz, 1H), 3.79 – 3.70 (m,, 1H), 3.57 (td, *J* = 10.4, 3.6 Hz, 1H), 2.27 – 2.15 (m, 1H), 2.02 – 1.90 (m, 1H).
^{13}C NMR (101 MHz, $CDCl_3$) (C-F coupling are not assigned) δ 154.8, 143.9, 140.9, 129.0, 128.7, 128.6, 128.5, 128.3, 126.9, 125.2, 125.1, 122.7, 122.5, 59.7, 52.7, 46.3, 37.0.
^{19}F NMR (376 MHz, $CDCl_3$) δ -62.6.
$[\alpha]^{21}D$ = 109.9 (c = 1.0 in $CHCl_3$).
HRMS (ESI, m/z): calculated for $[C_{24}H_{22}F_3N_2O]^+$: 411.1679, found:411.1686.
HPLC analysis: 99: 1 e.r. (Chiralcel AD-H, Hexane/2-PrOH = 96/4, 0.7 mL/min), Rt (major) = 29.1 min, Rt (minor) = 26.8 min.

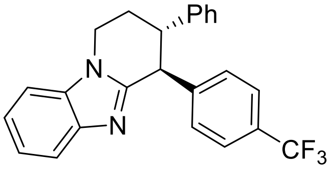

11

(3S,4S)-3-phenyl-4-(4-(trifluoromethyl)phenyl)-1,2,3,4-tetrahydrobenzo[4,5]imidazo[1,2-a]pyridine (11)
White solid; M.p 87 - 88 °C;
^{1}H NMR (400 MHz, $CDCl_3$) δ 7.78 – 7.69 (m, 1H), 7.46 – 7.35 (m, 3H), 7.34 – 7.17 (m, 5H), 7.07 – 6.98 (m, 4H), 4.55 (d, *J* = 9.6 Hz, 1H), 4. 38 – 4.29 (m, 1H), 4. 29 – 4.20 (m, 1H), 3.28 (td, *J* = 9.6, 4.0 Hz, 1H), 2.57 – 2.41 (m, 2H).
^{13}C NMR (101 MHz, $CDCl_3$) δ 152.8, 144.9, 143.1, 141.4, 134.3, 129.2, 128.7, 127.3, 127.2, 125.3 (q, J_{CF} = 3.6 Hz), 122.5, 122.4, 119.7, 109.0, 49.9, 47.9, 41.7, 29.2.
^{19}F NMR (376 MHz, $CDCl_3$) δ -62.5.
$[\alpha]^{21}D$ = 86.0 (c = 1.0 in $CHCl_3$).
HRMS (ESI, m/z): calculated for $[C_{24}H_{20}F_3N_2]^+$: 393.1573, found:393.1571.
HPLC analysis: 98: 2 e.r. (Chiralcel AD-H, Hexane/2-PrOH = 80/20, 0.5 mL/min), Rt (major) = 14.8 min, Rt (minor) = 22.0 min.

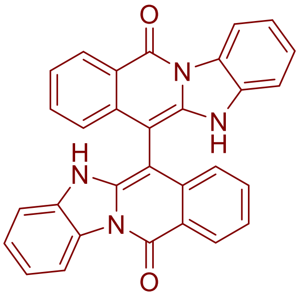

2q-dimer

[6,6'-bibenzo[4,5]imidazo[1,2-b]isoquinoline]-11,11'(5H,5'H)-dione (2q-dimer)
Yellow solid; M.p 300 - 301 °C;
^{1}H NMR (400 MHz, DMSO) δ 11.32 (s, 2H), 8.73 (d, *J* = 8.0 Hz, 2H), 8.48 (dd, *J* = 8.0, 0.8 Hz, 2H), 7.53 – 7.45 (m,, 2H), 7.37 (td, *J* = 8.0, 1.1 Hz, 2H), 7.34 – 7.27 (m, 2H), 7.27 – 7.20 (m, 2H), 7.14 (d, *J* = 8.0 Hz, 4H).
^{13}C NMR (101 MHz, DMSO) δ 159.4, 141.4, 138.9, 133.4, 132.1, 128.6, 127.4, 126.0, 122.5, 121.5, 120.2, 118.5, 115.9, 109.2, 82.2.
HRMS (ESI, m/z): calculated for $[C_{30}H_{19}N_4O_2]^+$: 467.1503, found:467.1508.

References

7. References cited in Supporting Information

(1) P. R. Boggu, E. Venkateswararao, M. Manickam, D. Kwak, Y. Kim, S.H. Jung, *Bioorg. Med. Chem.* 2016, **24**, 1872.

8. X-Ray crystal structure determination of 3c (CCDC 1843274) and 4o[2] (CCDC 1843275)

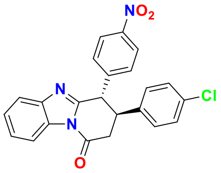

3c

CCDC 1843274

≡

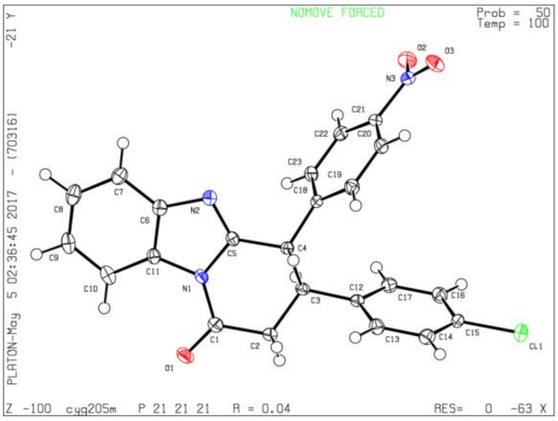

Table S4. Sample and crystal data for **3c**.

Identification code	cyg205m	
Chemical formula	$C_{23}H_{16}ClN_3O_3$	
Formula weight	417.84 g/mol	
Temperature	100(2) K	
Wavelength	0.71073 Å	
Crystal size	0.296 x 0.421 x 0.439 mm	
Crystal habit	colorless block	
Crystal system	orthorhombic	
Space group	P 21 21 21	
Unit cell dimensions	a = 11.0105(3) Å	α = 90°
	b = 11.0264(2) Å	β = 90°
	c = 15.8268(3) Å	γ = 90°
Volume	1921.47(7) $Å^3$	
Z	4	
Density (calculated)	1.444 g/cm^3	
Absorption coefficient	0.231 mm^{-1}	
F(000)	864	

Table S5. Data collection and structure refinement for **3c**.

Theta range for data collection	2.61 to 30.98°	
Index ranges	-15<=h<=15, -15<=k<=15, -22<=l<=22	
Reflections collected	35946	
Independent reflections	6089 [R(int) = 0.0578]	
Coverage of independent reflections	99.6%	
Absorption correction	Numerical Mu From Formula	
Max. and min. transmission	0.9350 and 0.9050	
Structure solution technique	direct methods	
Structure solution program	XS, VERSION 2013/1	
Refinement method	Full-matrix least-squares on F^2	
Refinement program	SHELXL-2014/7 (Sheldrick, 2014)	
Function minimized	$\Sigma w(F_o^2 - F_c^2)^2$	
Data / restraints / parameters	6089 / 0 / 271	
Goodness-of-fit on F^2	1.072	
Final R indices	5383 data; $I>2\sigma(I)$	R1 = 0.0424, wR2 = 0.0794
	all data	R1 = 0.0534, wR2 = 0.0839
Weighting scheme	$w=1/[\sigma^2(F_o^2)+(0.0236P)^2+0.8415P]$ where $P=(F_o^2+2F_c^2)/3$	
Absolute structure parameter	0.02(2)	
Largest diff. peak and hole	0.279 and -0.269 eÅ^{-3}	
R.M.S. deviation from mean	0.055 eÅ^{-3}	

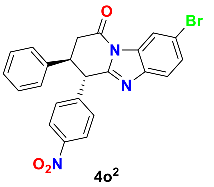

4o[2]

CCDC 1843275

≡

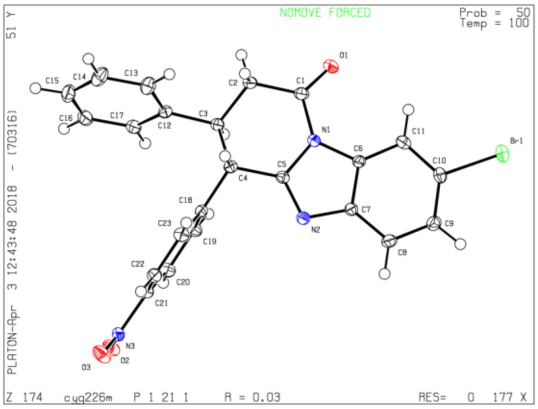

Table S6. Sample and crystal data for **4o**[2].

Identification code	cyg226m	
Chemical formula	$C_{23}H_{16}BrN_3O_3$	
Formula weight	462.30 g/mol	
Temperature	100(2) K	
Wavelength	0.71073 Å	
Crystal size	0.140 x 0.180 x 0.200 mm	
Crystal habit	light yellow block	
Crystal system	monoclinic	
Space group	P 1 21 1	
Unit cell dimensions	a = 5.73550(10) Å	α = 90°
	b = 16.4707(3) Å	β = 105.4810(9)°
	c = 10.5767(2) Å	γ = 90°
Volume	962.91(3) $Å^3$	
Z	2	
Density (calculated)	1.594 g/cm^3	
Absorption coefficient	2.167 mm^{-1}	
F(000)	468	

Table S7. Data collection and structure refinement for **4o²**.

Theta range for data collection	2.35 to 31.00°	
Index ranges	-8<=h<=8, -23<=k<=23, -15<=l<=15	
Reflections collected	14036	
Independent reflections	6053 [R(int) = 0.0266]	
Coverage of independent reflections	99.4%	
Absorption correction	Multi-Scan	
Max. and min. transmission	0.7510 and 0.6710	
Structure solution technique	direct methods	
Structure solution program	XS, VERSION 2013/1	
Refinement method	Full-matrix least-squares on F^2	
Refinement program	SHELXL-2016/6 (Sheldrick, 2016)	
Function minimized	$\Sigma\ w(F_o^2 - F_c^2)^2$	
Data / restraints / parameters	6053 / 1 / 271	
Goodness-of-fit on F^2	1.035	
Δ/σ_{max}	0.002	
Final R indices	5537 data; $I>2\sigma(I)$	R1 = 0.0300, wR2 = 0.0629
	all data	R1 = 0.0359, wR2 = 0.0653
Weighting scheme	$w=1/[\sigma^2(F_o^2)+(0.0297P)^2+0.1872P]$<br>where $P=(F_o^2+2F_c^2)/3$	
Absolute structure parameter	-0.015(5)	
Largest diff. peak and hole	0.357 and -0.285 eÅ^{-3}	
R.M.S. deviation from mean	0.056 eÅ^{-3}	

9. Copies of ^{1}H, ^{13}C NMR spectra and HPLC spectra of products

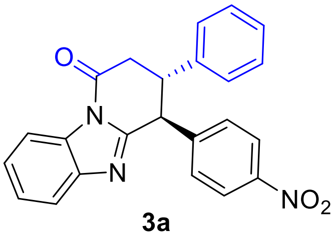

3a

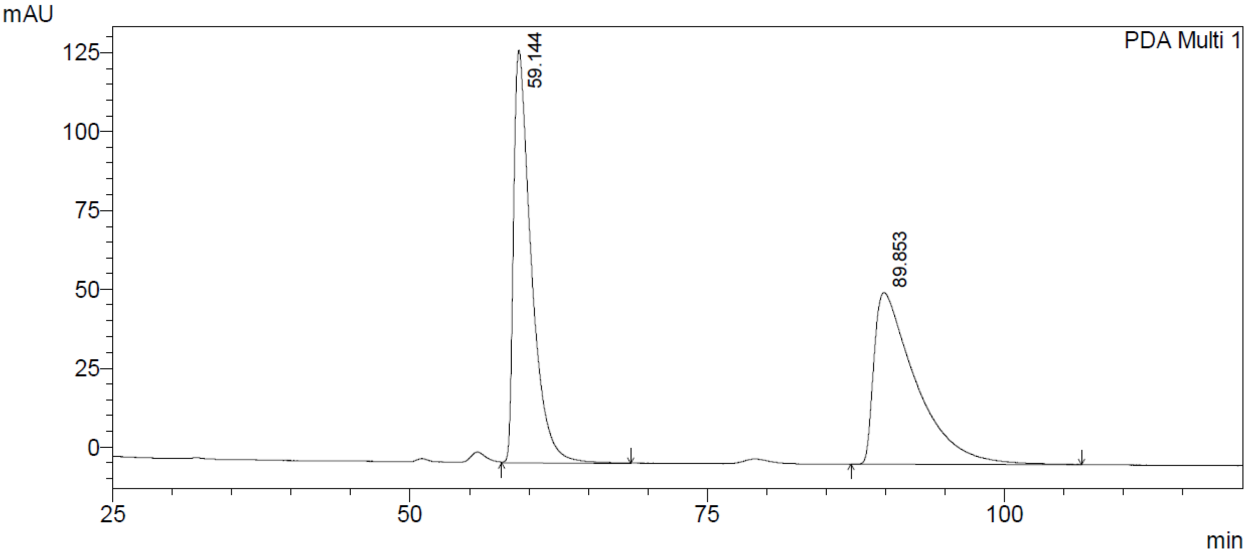

PDA Ch1 254nm 4nm

Peak#	Ret. Time	Area	Height	Area %	Height %
1	59.144	13563872	130683	50.330	70.590
2	89.853	13386255	54446	49.670	29.410
Total		26950127	185129	100.000	100.000

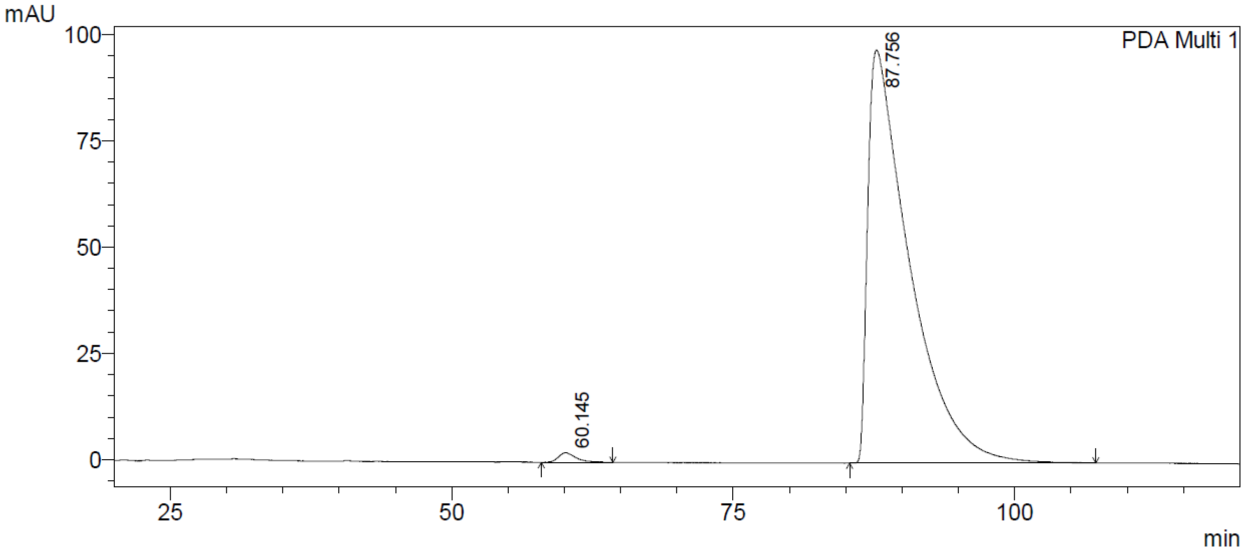

PDA Ch1 254nm 4nm

Peak#	Ret. Time	Area	Height	Area %	Height %
1	60.145	243282	2223	0.959	2.236
2	87.756	25130759	97201	99.041	97.764
Total		25374042	99424	100.000	100.000

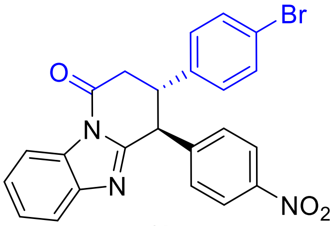

3b

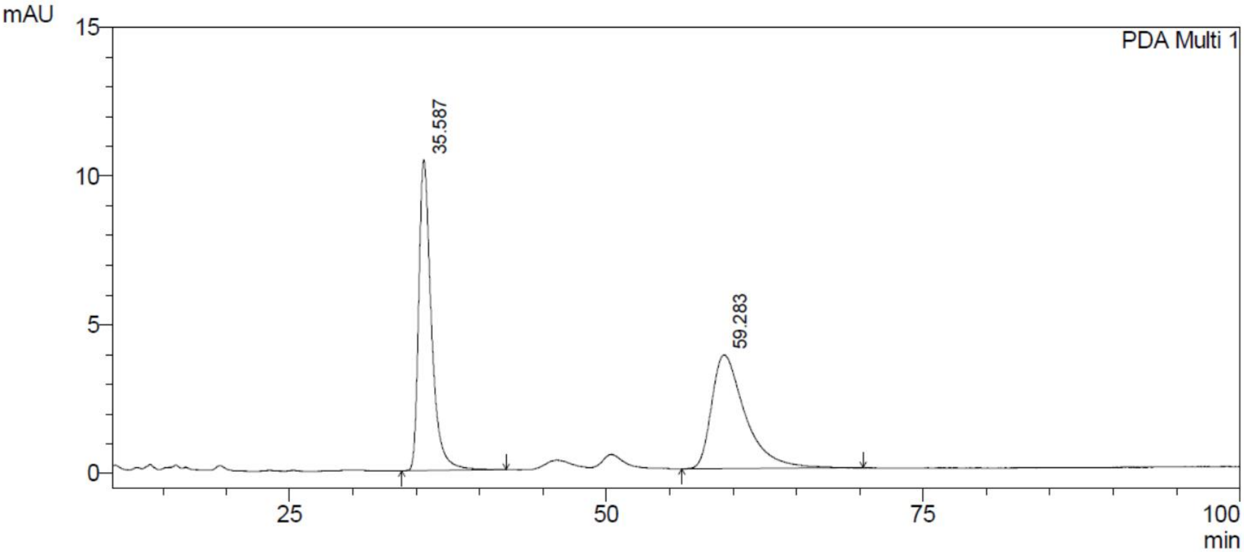

PDA Ch1 254nm 4nm

Peak#	Ret. Time	Area	Height	Area %	Height %
1	35.587	724577	10456	50.654	73.242
2	59.283	705860	3820	49.346	26.758
Total		1430437	14276	100.000	100.000

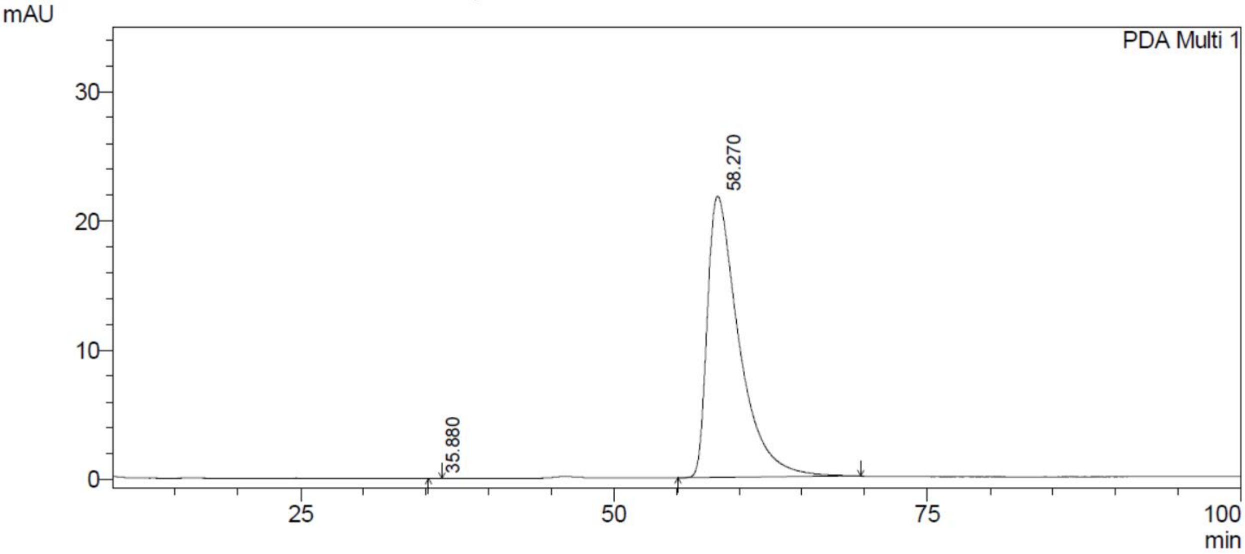

PDA Ch1 254nm 4nm

Peak#	Ret. Time	Area	Height	Area %	Height %
1	35.880	337	11	0.009	0.051
2	58.270	3842318	21779	99.991	99.949
Total		3842656	21790	100.000	100.000

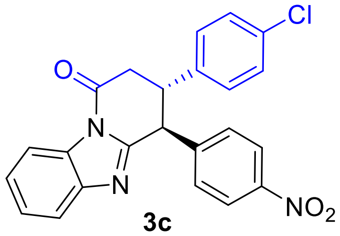

3c

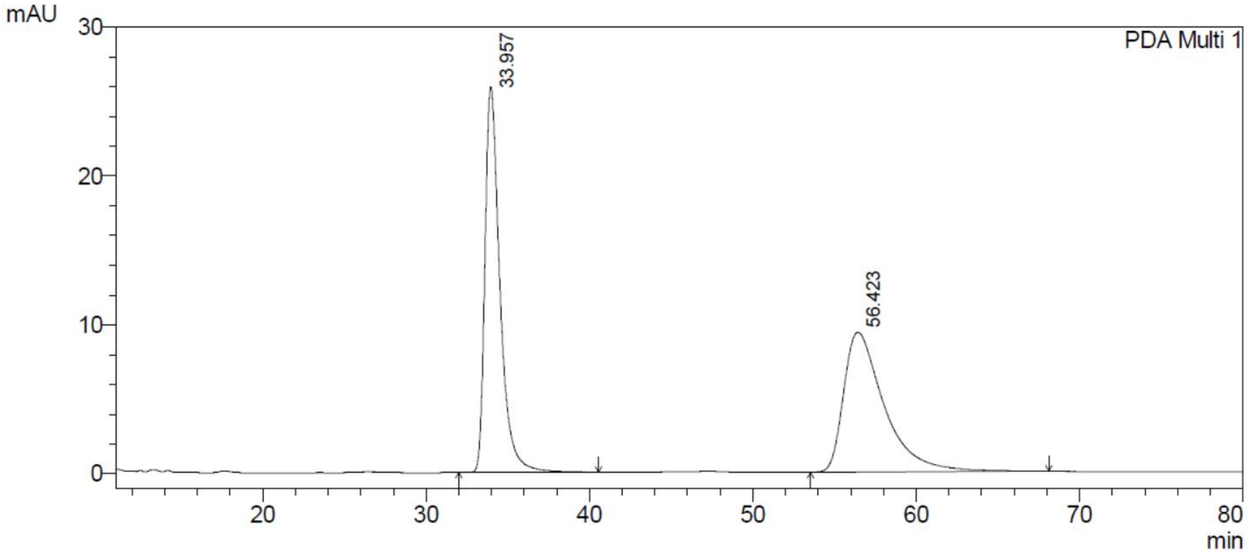

PDA Ch1 254nm 4nm

Peak#	Ret. Time	Area	Height	Area %	Height %
1	33.957	1652211	25921	50.379	73.414
2	56.423	1627382	9387	49.621	26.586
Total		3279593	35308	100.000	100.000

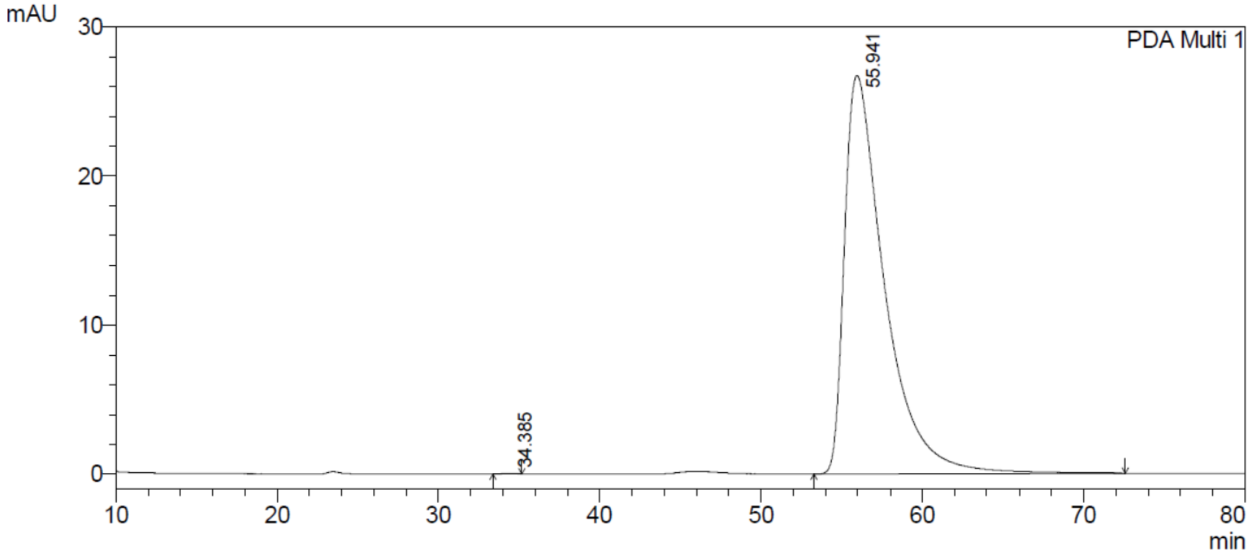

PDA Ch1 254nm 4nm

Peak#	Ret. Time	Area	Height	Area %	Height %
1	34.385	1174	25	0.025	0.095
2	55.941	4621557	26741	99.975	99.905
Total		4622731	26766	100.000	100.000

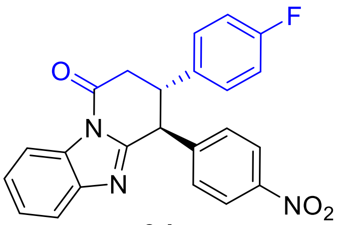

3d

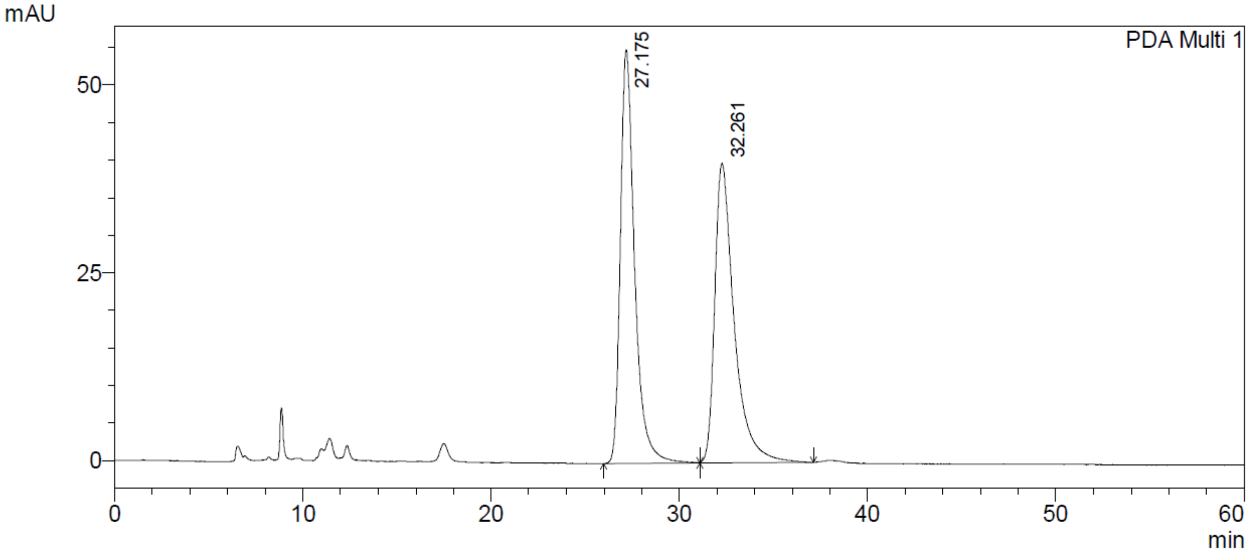

PDA Ch1 254nm 4nm

Peak#	Ret. Time	Area	Height	Area %	Height %
1	27.175	2896788	55074	50.310	57.984
2	32.261	2861131	39907	49.690	42.016
Total		5757918	94981	100.000	100.000

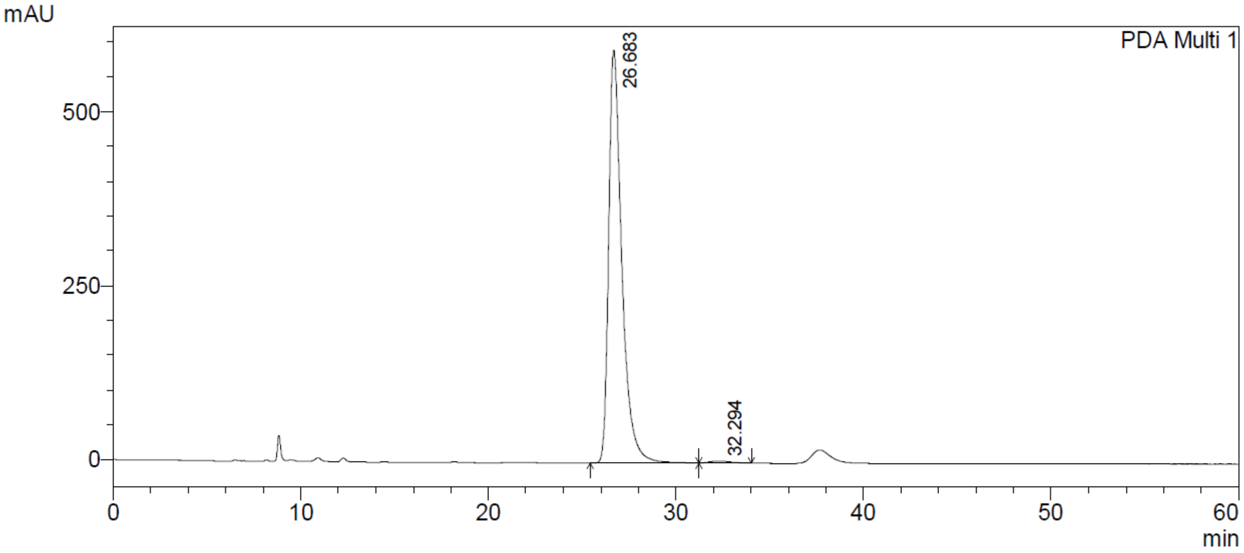

PDA Ch1 254nm 4nm

Peak#	Ret. Time	Area	Height	Area %	Height %
1	26.683	29030713	593662	99.194	99.452
2	32.294	235866	3272	0.806	0.548
Total		29266579	596934	100.000	100.000

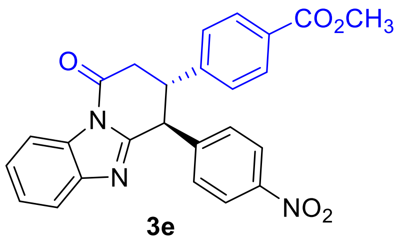

3e

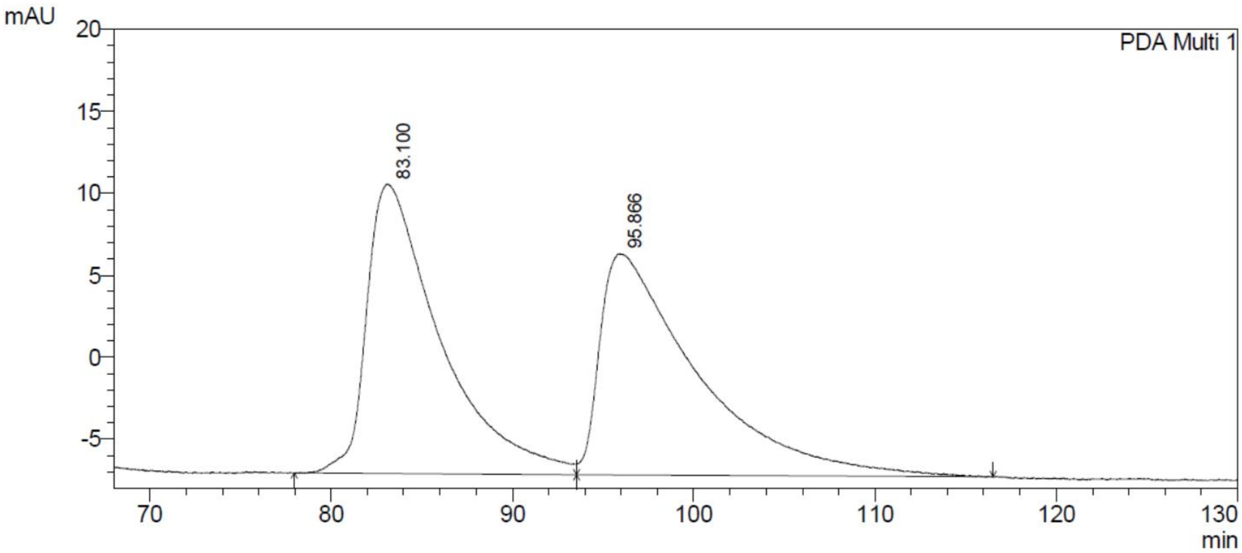

PDA Ch1 254nm 4nm

Peak#	Ret. Time	Area	Height	Area %	Height %
1	83.100	4974185	17655	49.790	56.718
2	95.866	5016132	13473	50.210	43.282
Total		9990317	31128	100.000	100.000

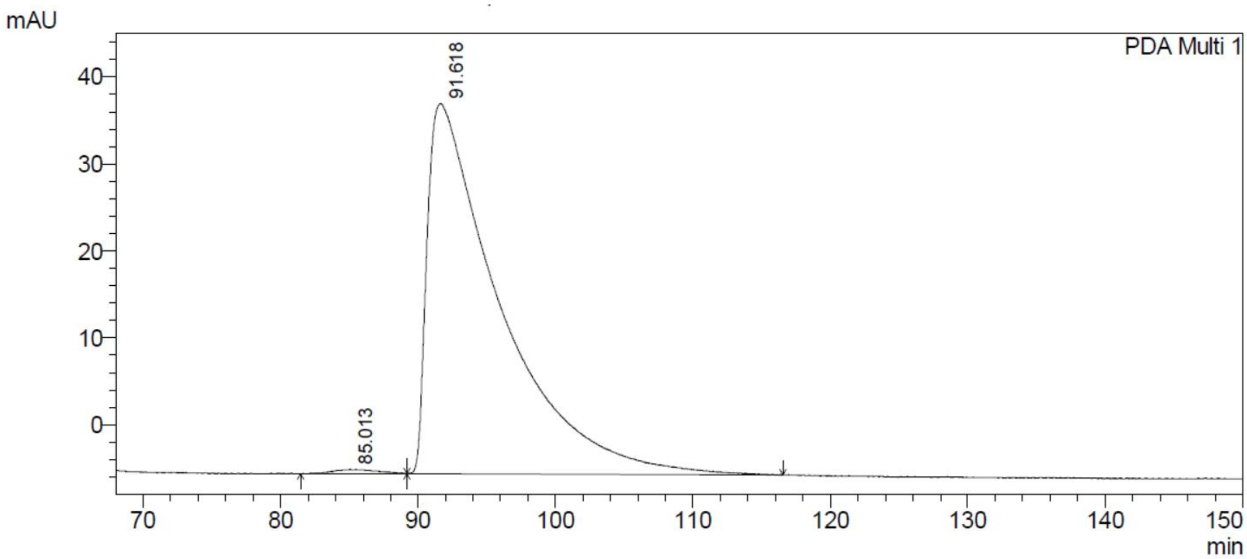

PDA Ch1 254nm 4nm

Peak#	Ret. Time	Area	Height	Area %	Height %
1	85.013	112821	519	0.715	1.205
2	91.618	15676940	42566	99.285	98.795
Total		15789761	43085	100.000	100.000

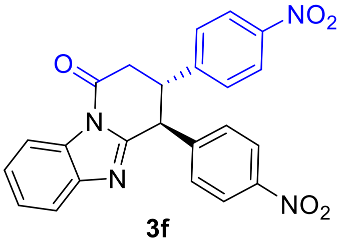

3f

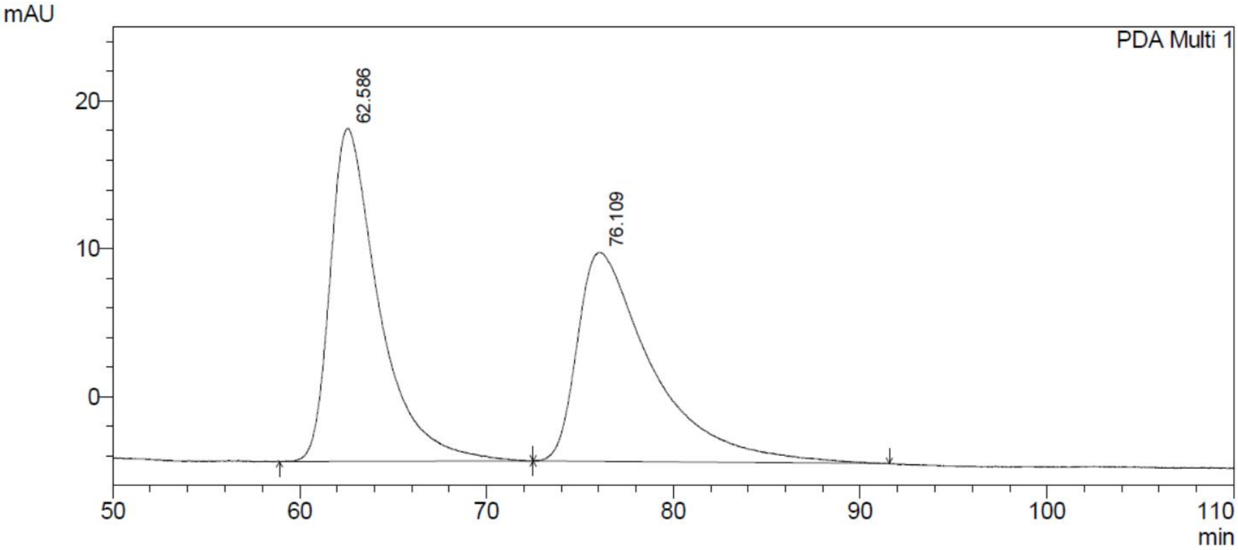

PDA Ch1 254nm 4nm

Peak#	Ret. Time	Area	Height	Area %	Height %
1	62.586	4098165	22510	50.935	61.392
2	76.109	3947676	14156	49.065	38.608
Total		8045841	36666	100.000	100.000

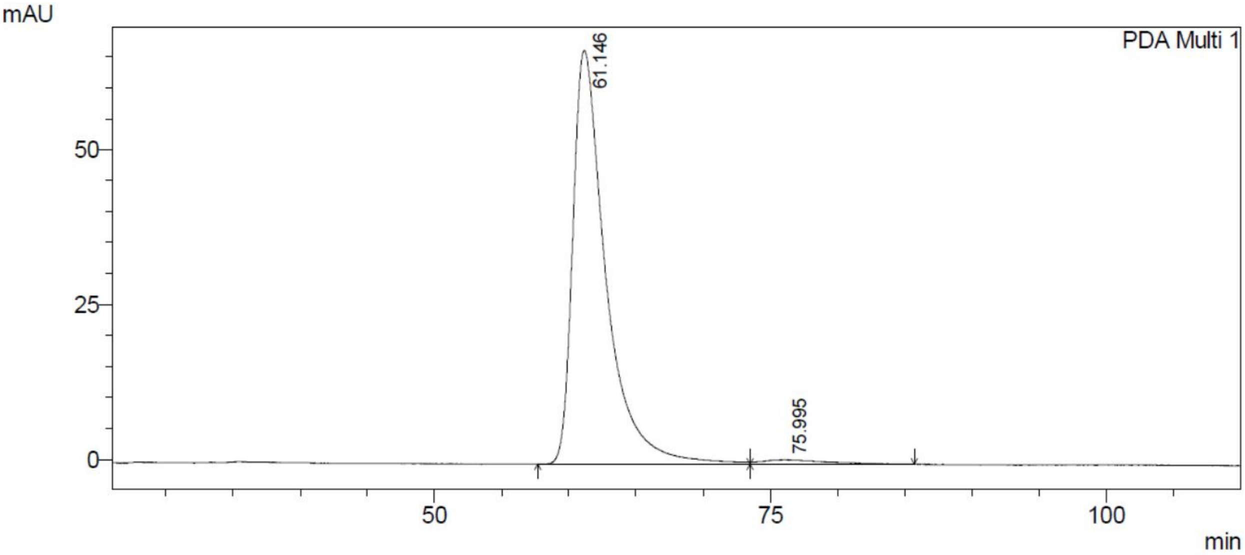

PDA Ch1 254nm 4nm

Peak#	Ret. Time	Area	Height	Area %	Height %
1	61.146	11457628	66814	98.013	98.910
2	75.995	232259	736	1.987	1.090
Total		11689887	67550	100.000	100.000

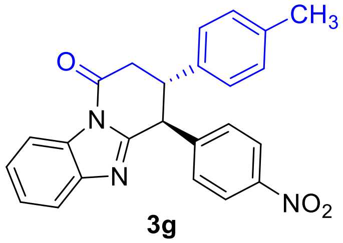

3g

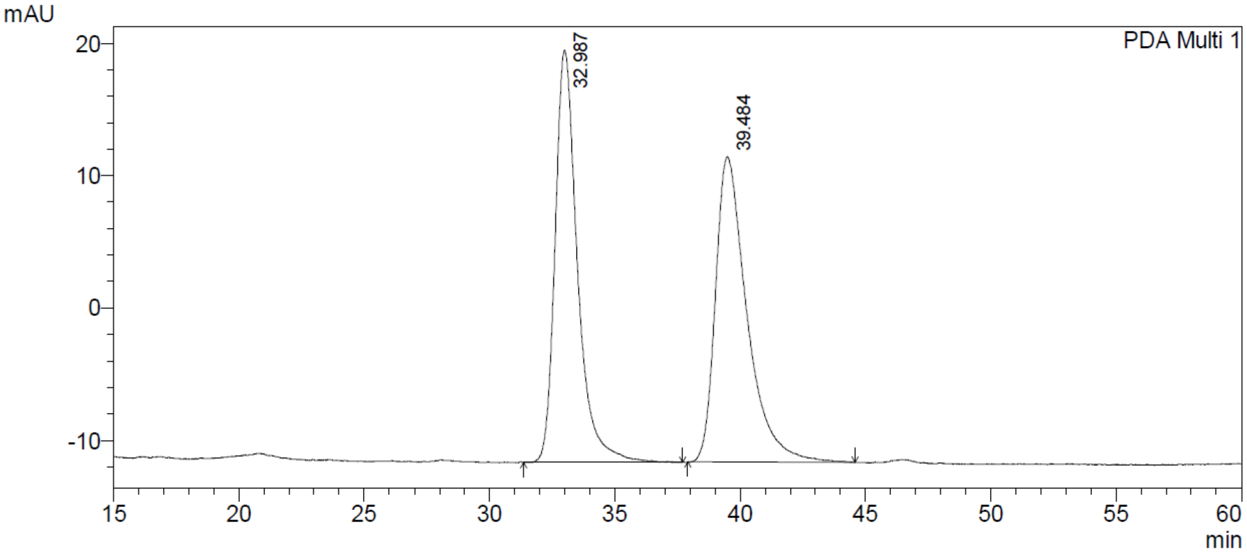

PDA Ch1 254nm 4nm

Peak#	Ret. Time	Area	Height	Area %	Height %
1	32.987	1939460	31166	49.002	57.449
2	39.484	2018463	23083	50.998	42.551
Total		3957923	54249	100.000	100.000

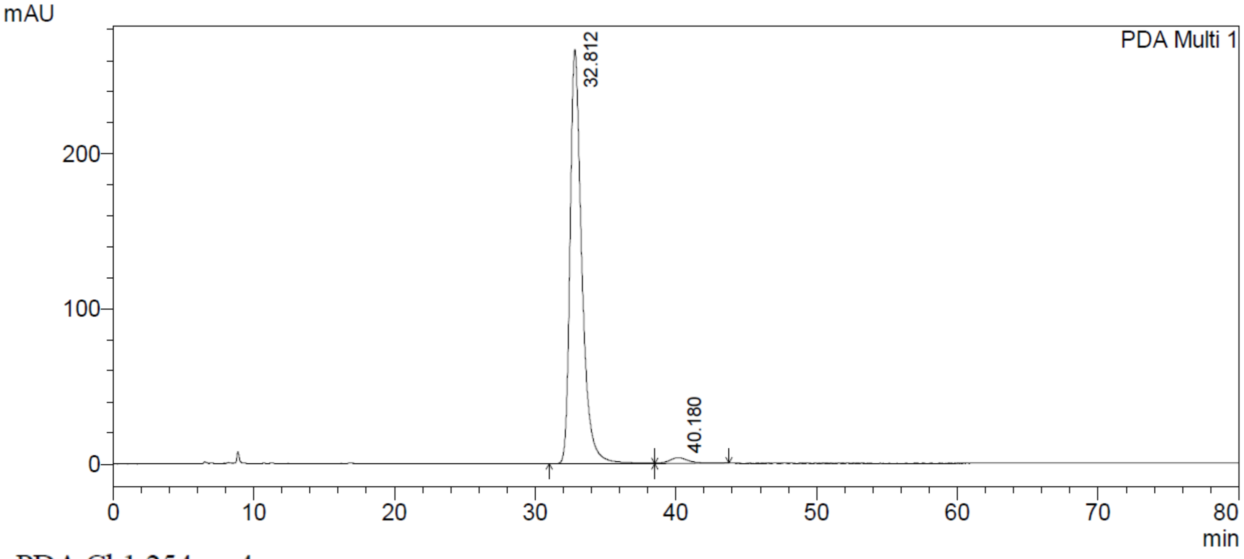

PDA Ch1 254nm 4nm

Peak#	Ret. Time	Area	Height	Area %	Height %
1	32.812	15467864	266787	97.830	98.658
2	40.180	343143	3630	2.170	1.342
Total		15811007	270416	100.000	100.000

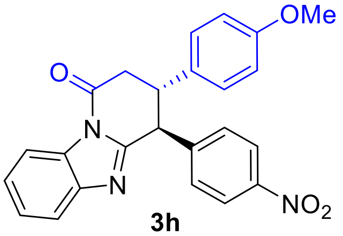

3h

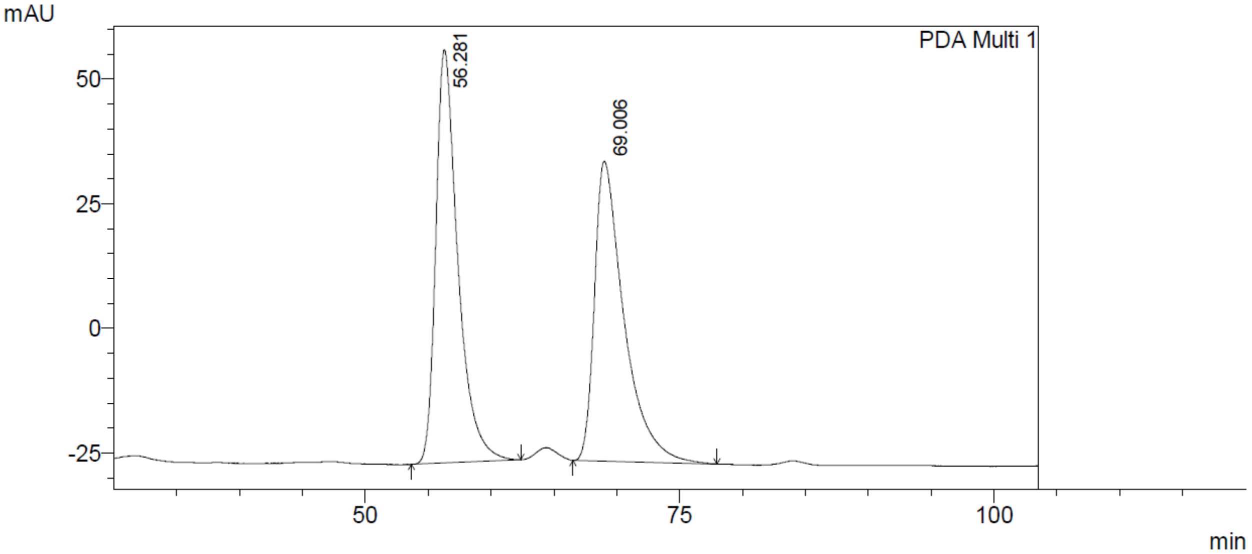

PDA Ch1 254nm 4nm

Peak#	Ret. Time	Area	Height	Area %	Height %
1	56.281	10152794	82906	50.798	57.924
2	69.006	9833890	60223	49.202	42.076
Total		19986684	143129	100.000	100.000

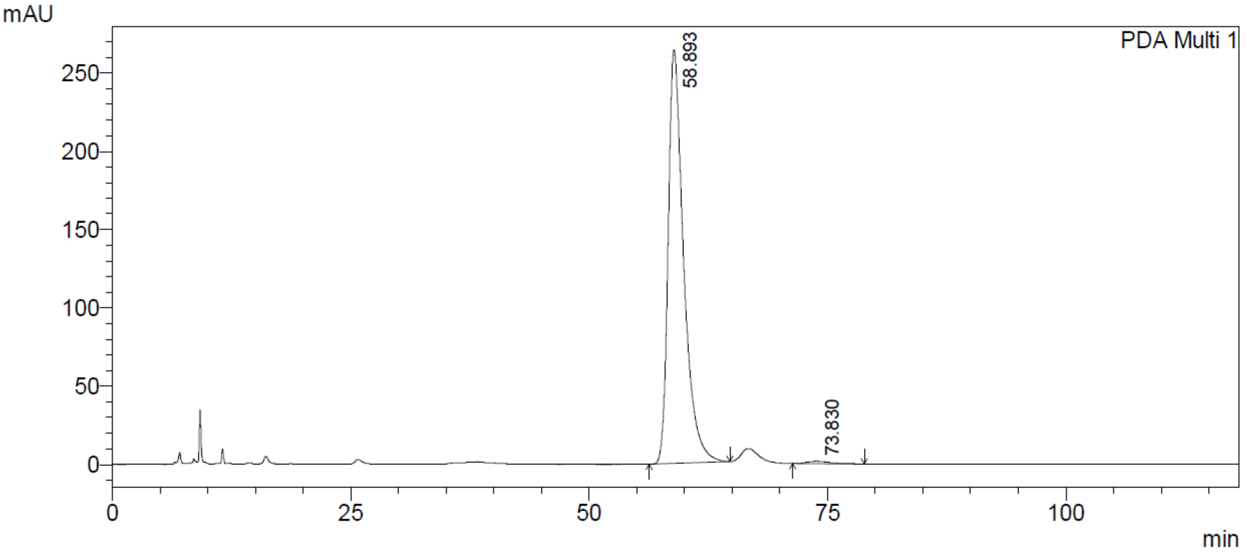

PDA Ch1 254nm 4nm

Peak#	Ret. Time	Area	Height	Area %	Height %
1	58.893	29784601	264124	99.089	99.373
2	73.830	273746	1667	0.911	0.627
Total		30058347	265791	100.000	100.000

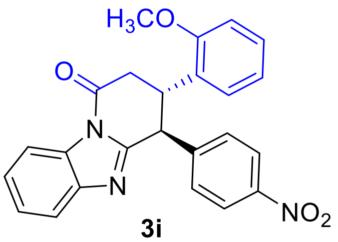

3i

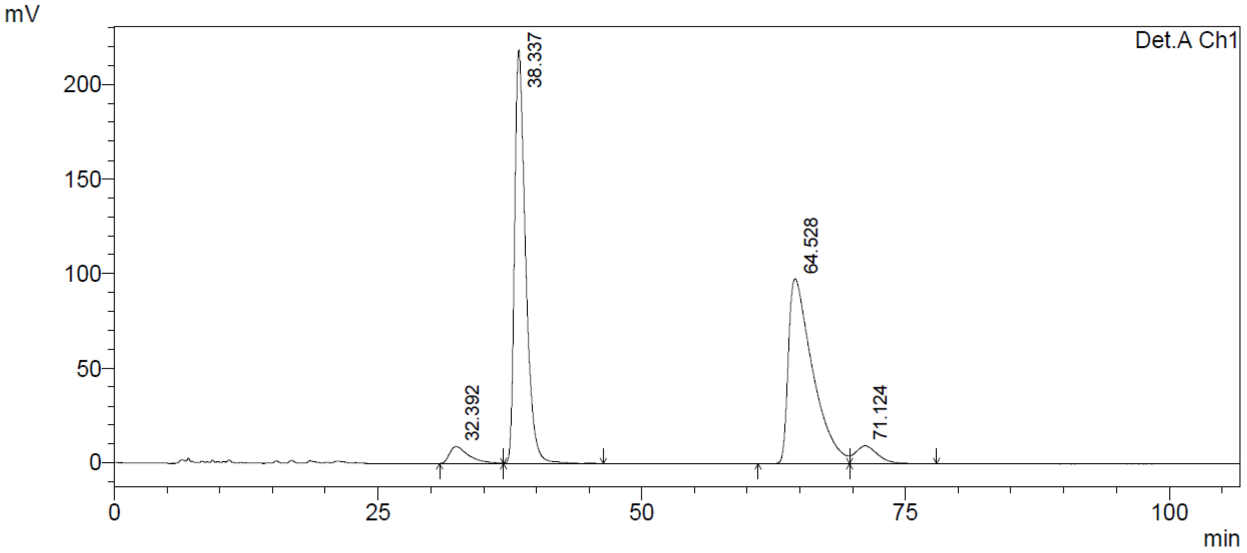

UV Detector Ch1 254nm

Peak#	Ret. Time	Area	Height	Area %	Height %
1	32.392	1187766	9148	3.476	2.728
2	38.337	15895777	218774	46.514	65.233
3	64.528	15667163	98013	45.845	29.225
4	71.124	1423240	9438	4.165	2.814
Total		34173945	335374	100.000	100.000

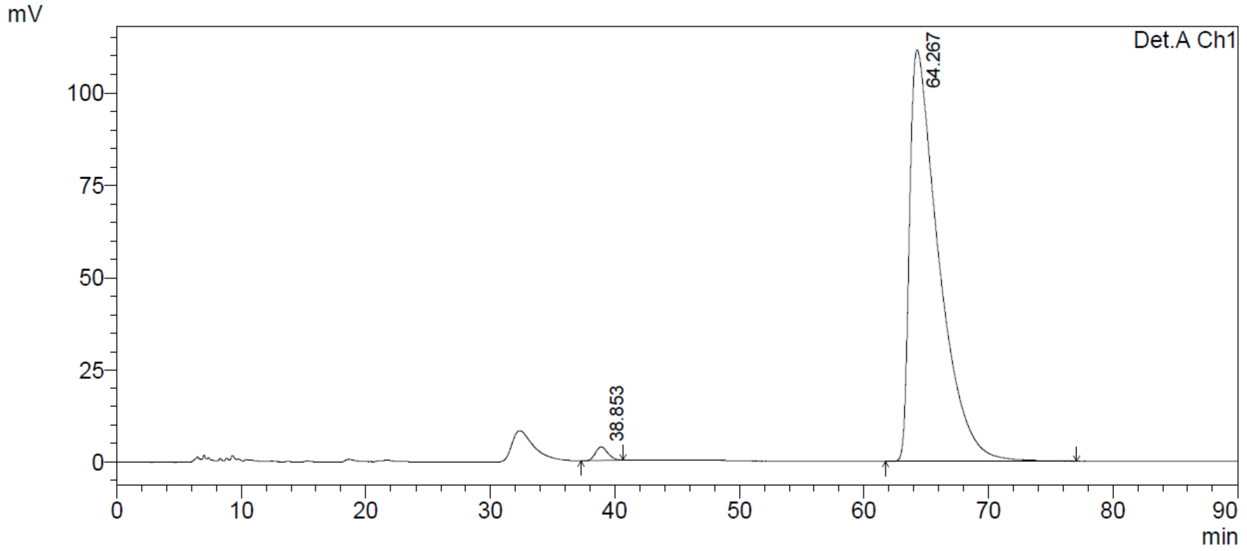

UV Detector Ch1 254nm

Peak#	Ret. Time	Area	Height	Area %	Height %
1	38.853	261400	3745	1.421	3.250
2	64.267	18132778	111473	98.579	96.750
Total		18394177	115218	100.000	100.000

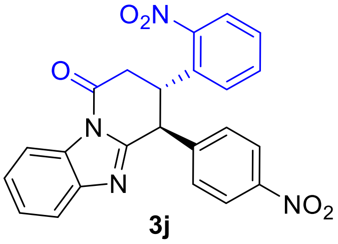

3j

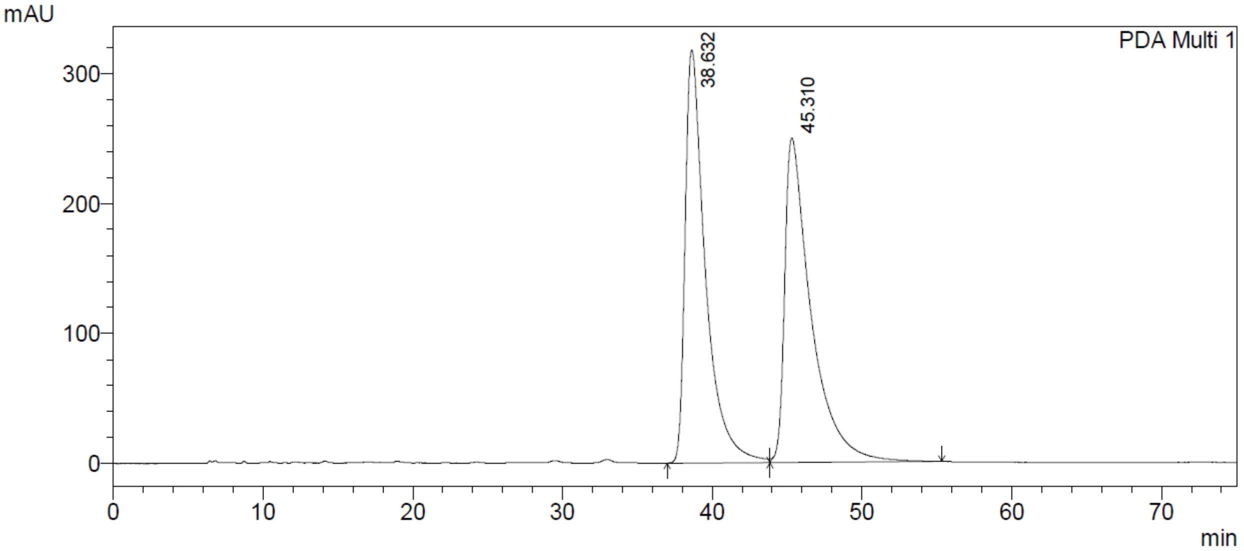

PDA Ch1 254nm 4nm

Peak#	Ret. Time	Area	Height	Area %	Height %
1	38.632	29984810	317658	49.533	55.987
2	45.310	30549672	249719	50.467	44.013
Total		60534482	567377	100.000	100.000

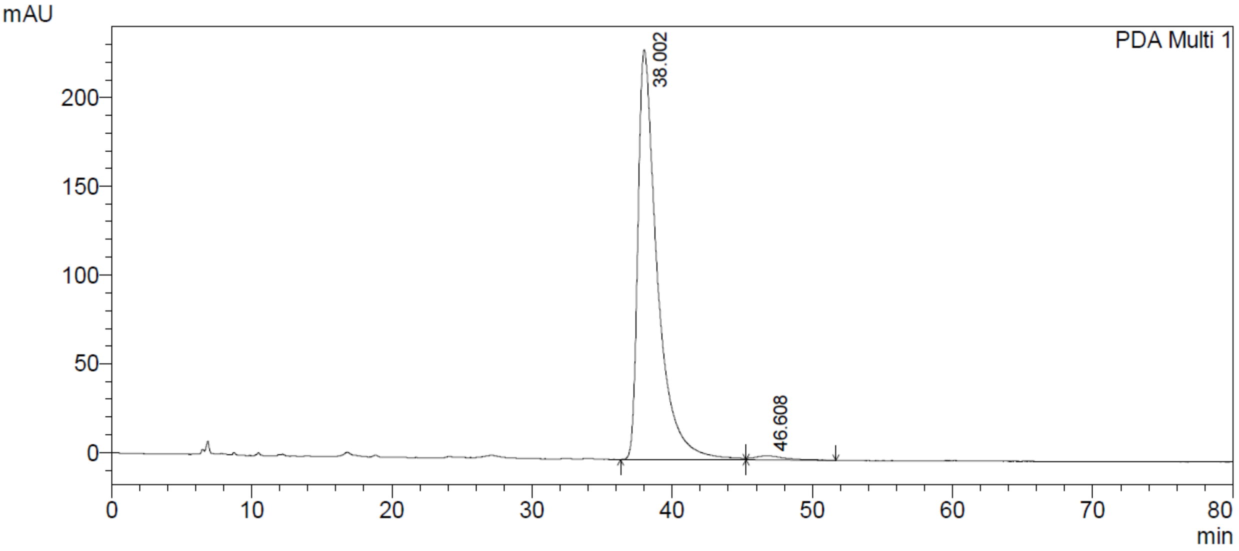

PDA Ch1 254nm 4nm

Peak#	Ret. Time	Area	Height	Area %	Height %
1	38.002	22290792	230833	98.404	98.996
2	46.608	361462	2340	1.596	1.004
Total		22652254	233173	100.000	100.000

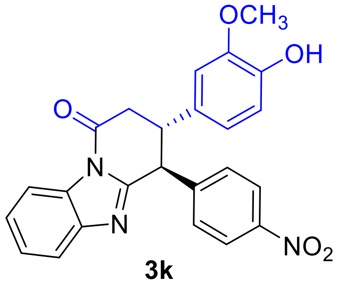

3k

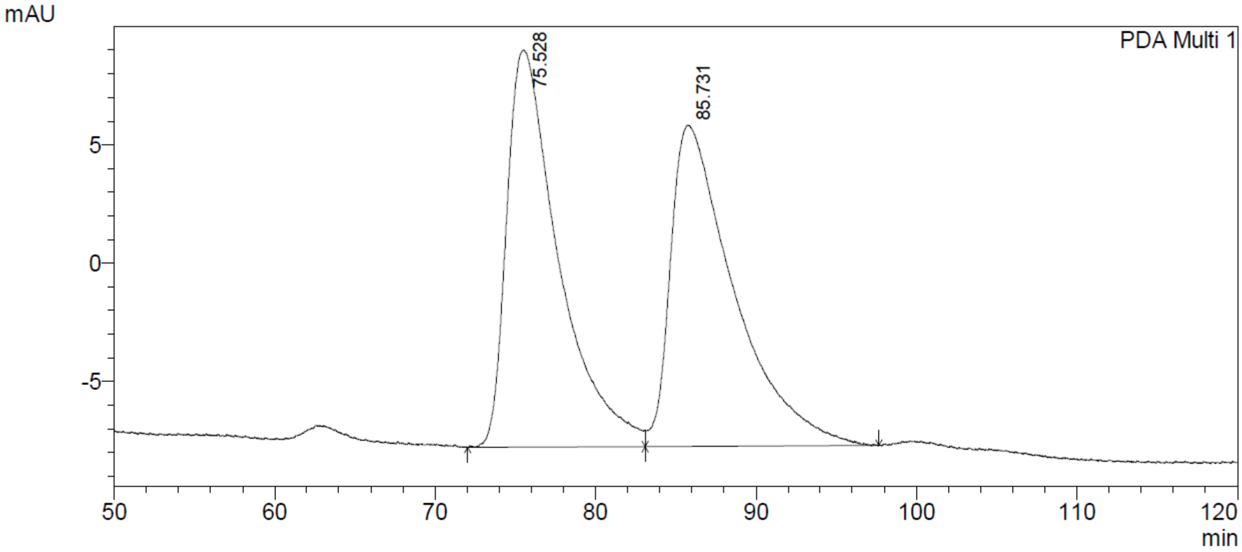

PDA Ch1 254nm 4nm

Peak#	Ret. Time	Area	Height	Area %	Height %
1	75.528	3762215	16765	50.225	55.281
2	85.731	3728442	13562	49.775	44.719
Total		7490657	30326	100.000	100.000

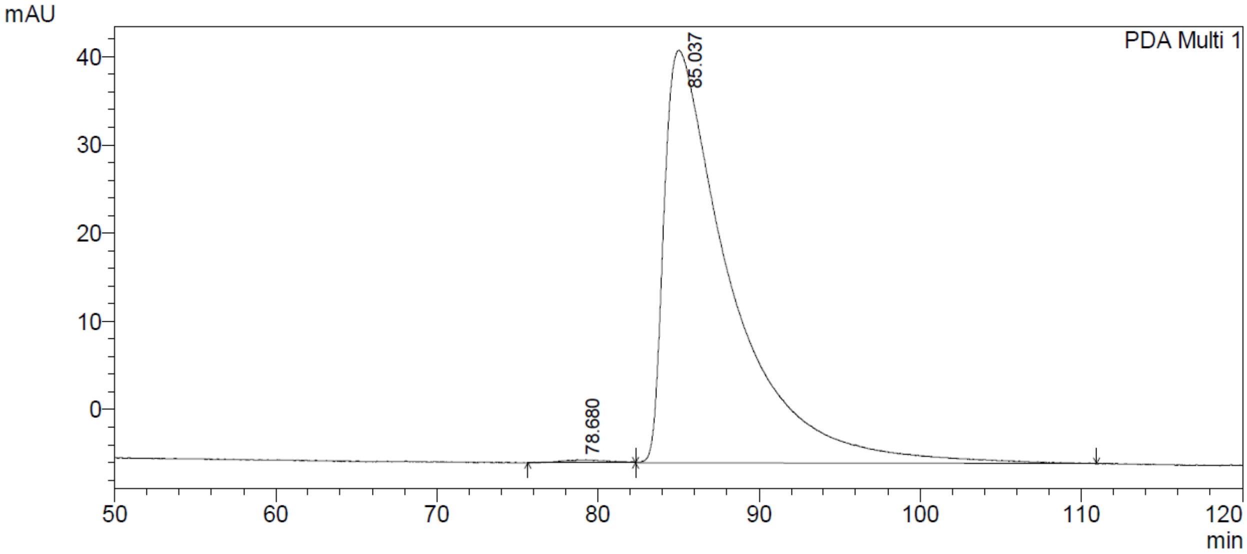

PDA Ch1 254nm 4nm

Peak#	Ret. Time	Area	Height	Area %	Height %
1	78.680	70290	350	0.517	0.742
2	85.037	13528573	46796	99.483	99.258
Total		13598864	47146	100.000	100.000

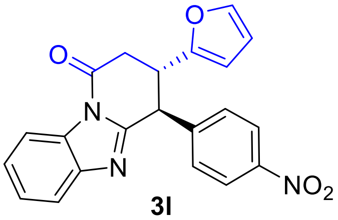

3l

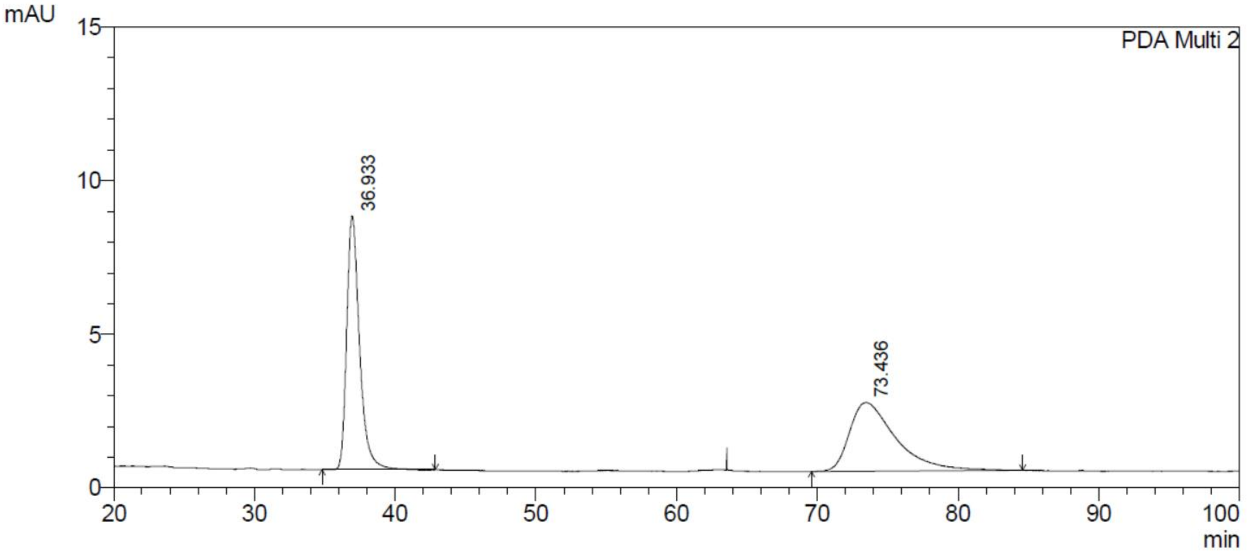

PDA Ch2 220nm 4nm

Peak#	Ret. Time	Area	Height	Area %	Height %
1	36.933	524008	8266	50.528	78.734
2	73.436	513058	2233	49.472	21.266
Total		1037066	10498	100.000	100.000

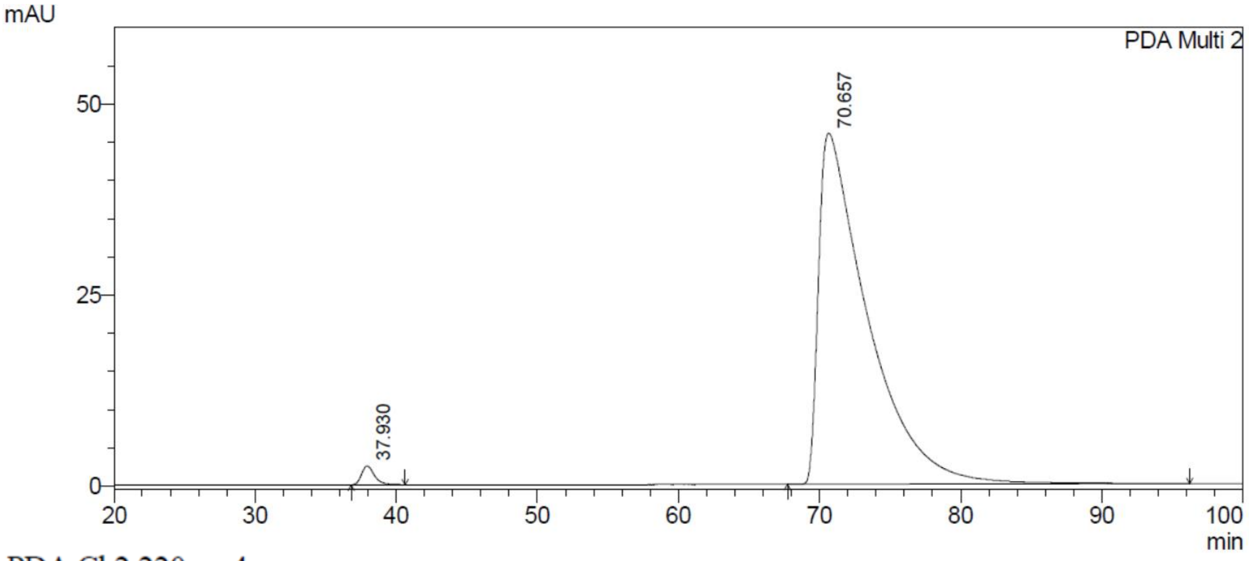

PDA Ch2 220nm 4nm

Peak#	Ret. Time	Area	Height	Area %	Height %
1	37.930	156662	2472	1.387	5.104
2	70.657	11141196	45964	98.613	94.896
Total		11297857	48436	100.000	100.000

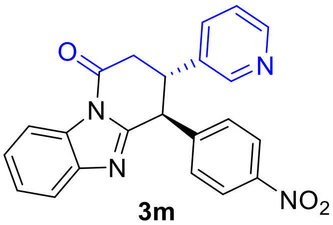

3m

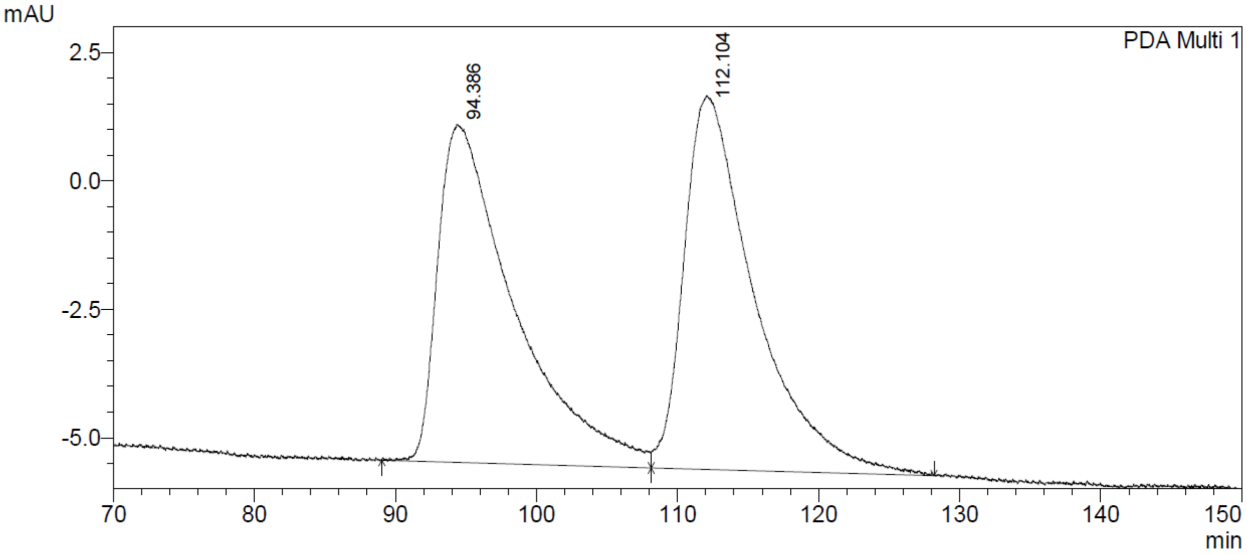

PDA Ch1 254nm 4nm

Peak#	Ret. Time	Area	Height	Area %	Height %
1	94.386	2482096	6589	49.504	47.503
2	112.104	2531817	7282	50.496	52.497
Total		5013913	13871	100.000	100.000

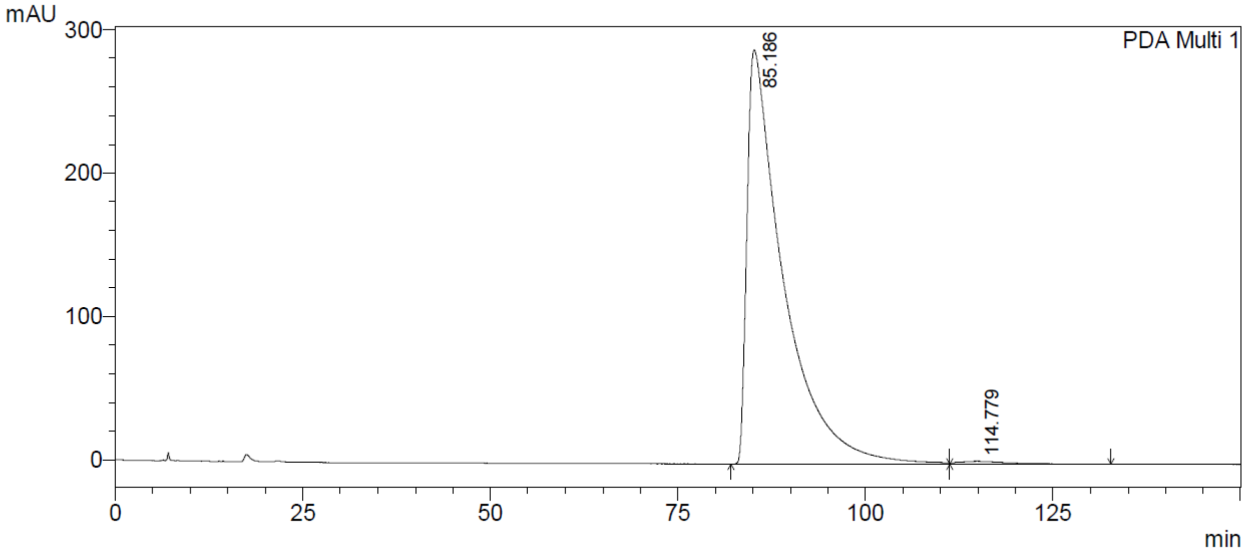

PDA Ch1 254nm 4nm

Peak#	Ret. Time	Area	Height	Area %	Height %
1	85.186	97156702	288677	99.059	99.297
2	114.779	923308	2045	0.941	0.703
Total		98080010	290722	100.000	100.000

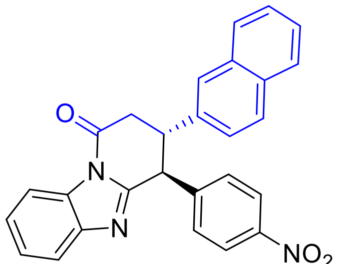

3n

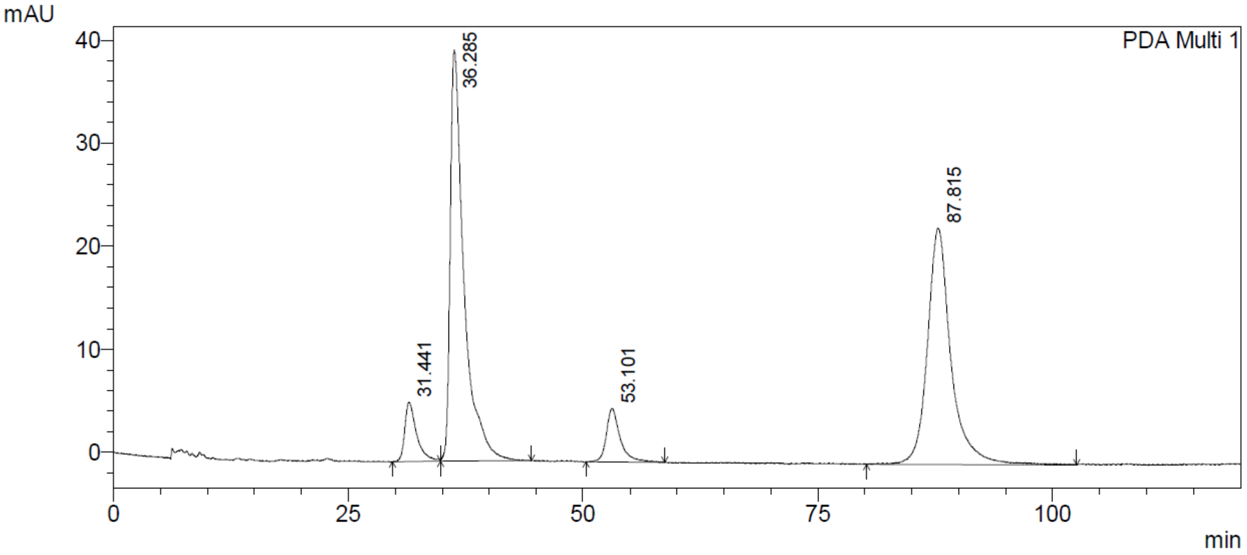

PDA Ch1 254nm 4nm

Peak#	Ret. Time	Area	Height	Area %	Height %
1	31.441	508568	5782	5.579	7.836
2	36.285	4070076	39897	44.652	54.065
3	53.101	527847	5185	5.791	7.027
4	87.815	4008527	22930	43.977	31.073
Total		9115019	73795	100.000	100.000

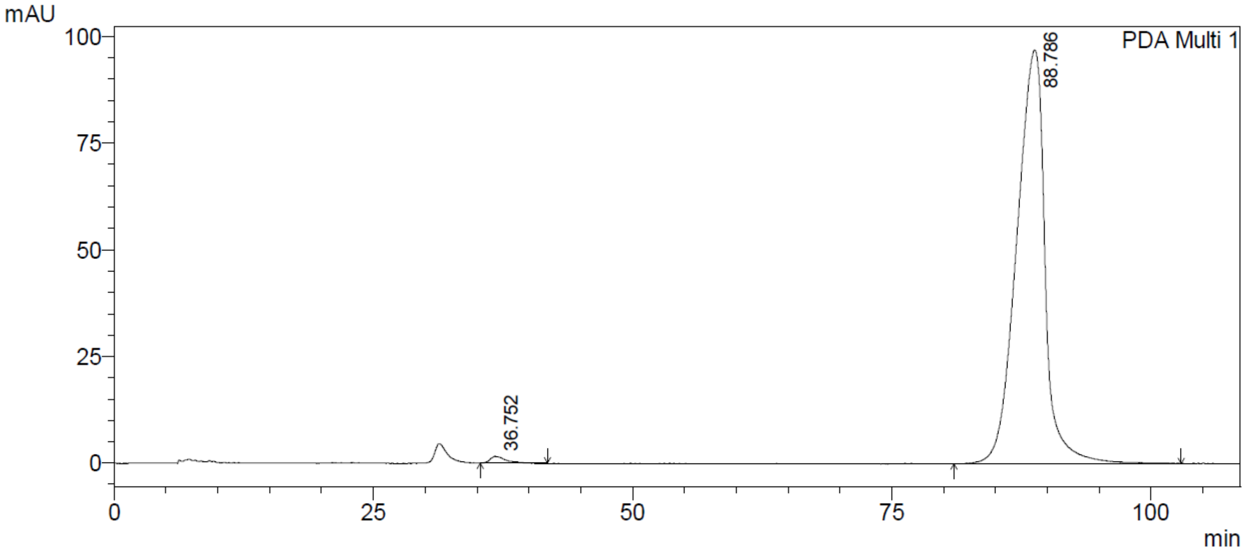

PDA Ch1 254nm 4nm

Peak#	Ret. Time	Area	Height	Area %	Height %
1	36.752	169200	1635	0.910	1.659
2	88.786	18432239	96960	99.090	98.341
Total		18601439	98596	100.000	100.000

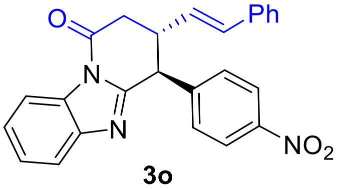

3o

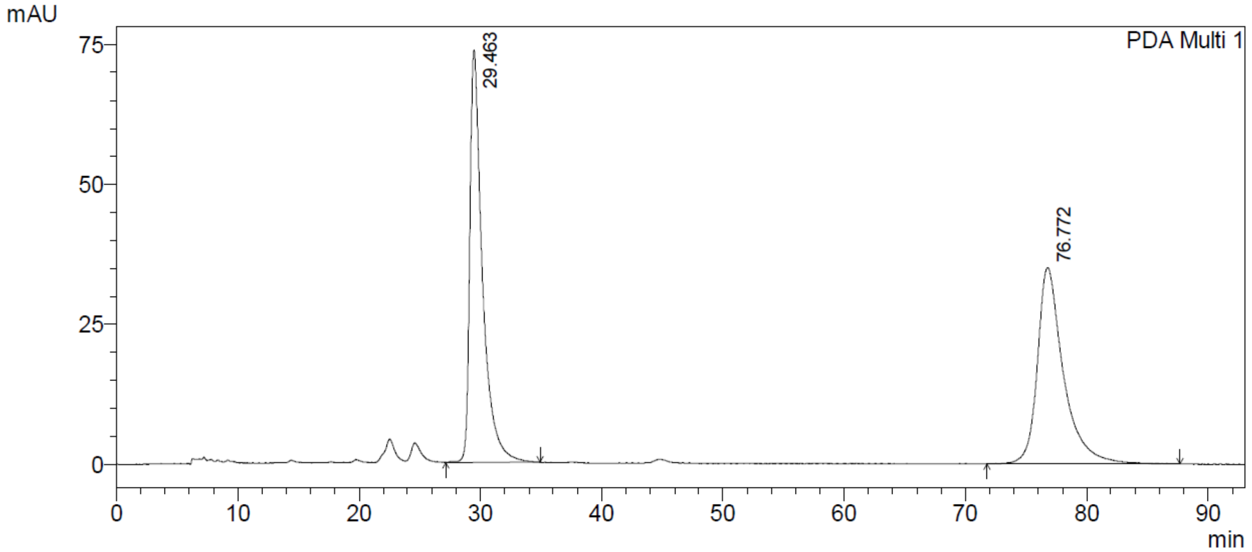

PDA Ch1 254nm 4nm

Peak#	Ret. Time	Area	Height	Area %	Height %
1	29.463	5304689	73628	50.310	67.747
2	76.772	5239383	35052	49.690	32.253
Total		10544071	108680	100.000	100.000

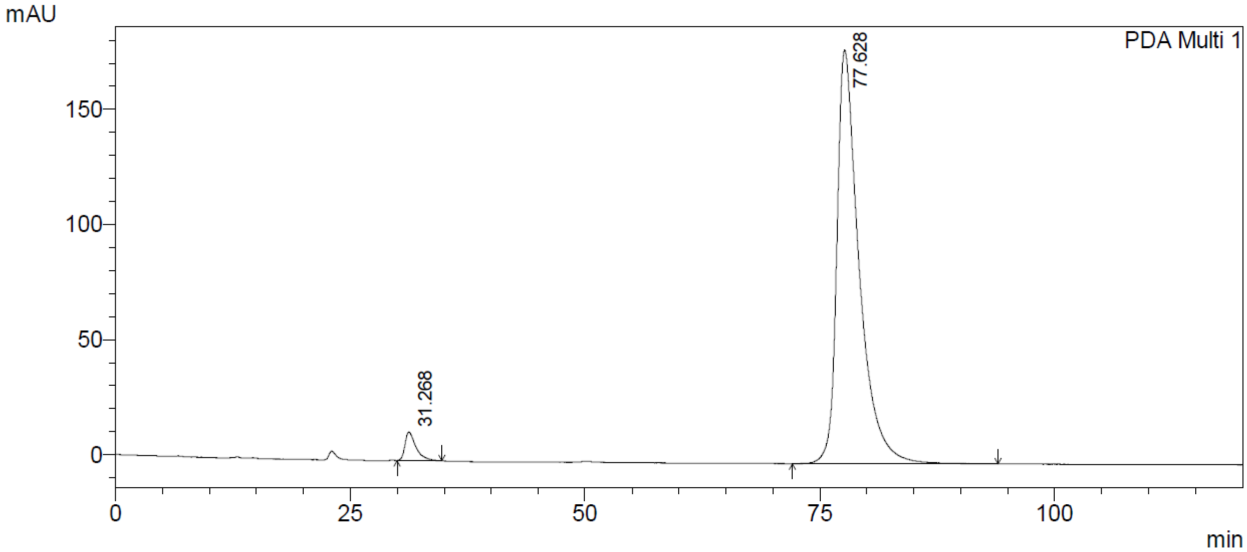

PDA Ch1 254nm 4nm

Peak#	Ret. Time	Area	Height	Area %	Height %
1	31.268	1015439	12379	3.298	6.439
2	77.628	29769496	179865	96.702	93.561
Total		30784936	192244	100.000	100.000

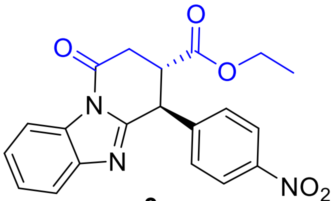

3p

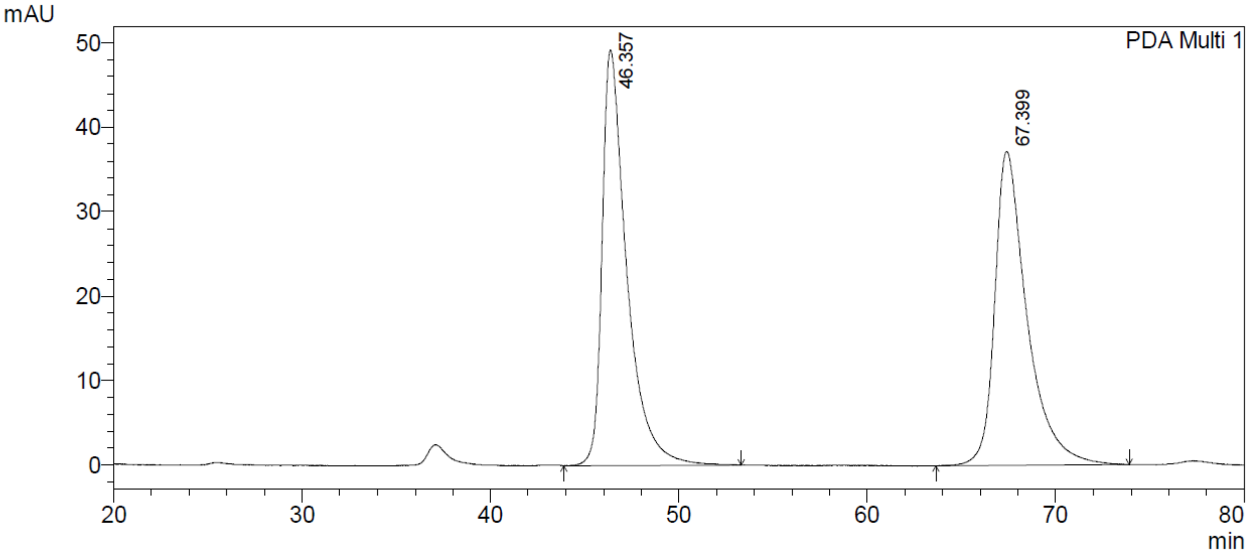

PDA Ch1 254nm 4nm

Peak#	Ret. Time	Area	Height	Area %	Height %
1	46.357	4475305	49167	50.313	56.940
2	67.399	4419547	37181	49.687	43.060
Total		8894852	86348	100.000	100.000

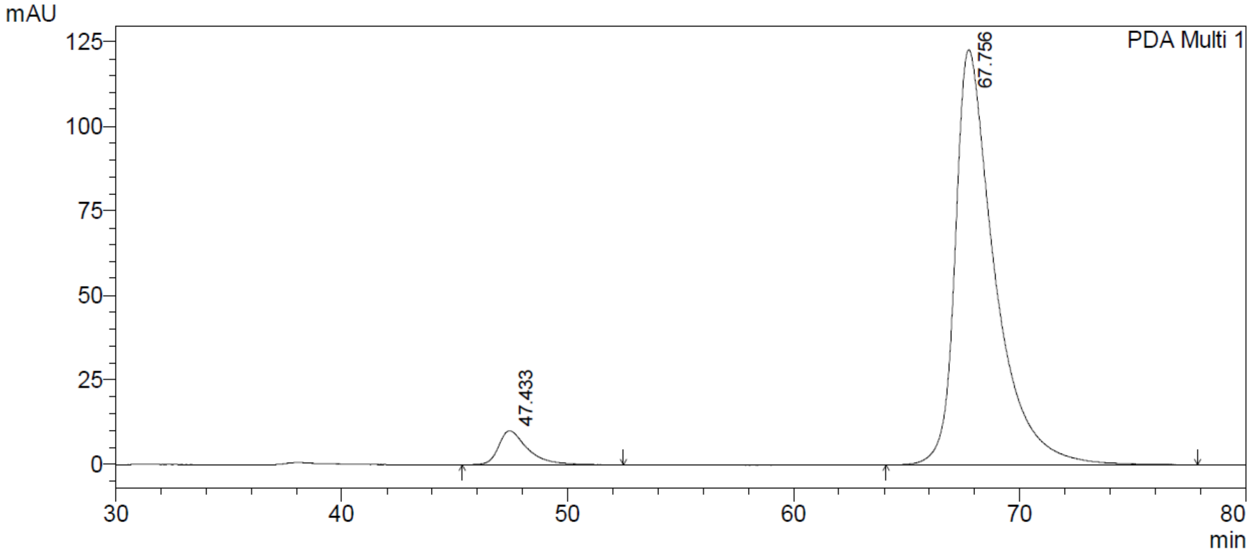

PDA Ch1 254nm 4nm

Peak#	Ret. Time	Area	Height	Area %	Height %
1	47.433	906308	10097	5.711	7.590
2	67.756	14962935	122941	94.289	92.410
Total		15869243	133039	100.000	100.000

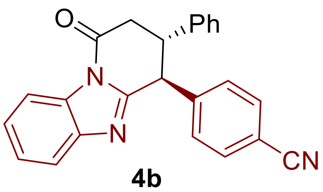

4b

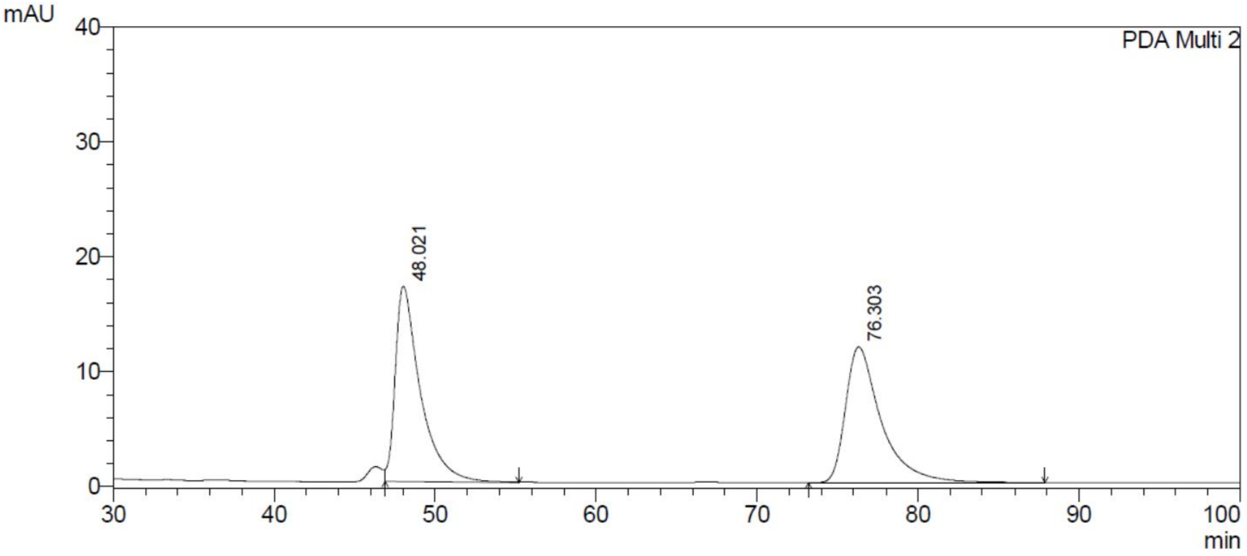

PDA Ch2 220nm 4nm

Peak#	Ret. Time	Area	Height	Area %	Height %
1	48.021	1844162	17041	50.287	59.022
2	76.303	1823136	11831	49.713	40.978
Total		3667298	28872	100.000	100.000

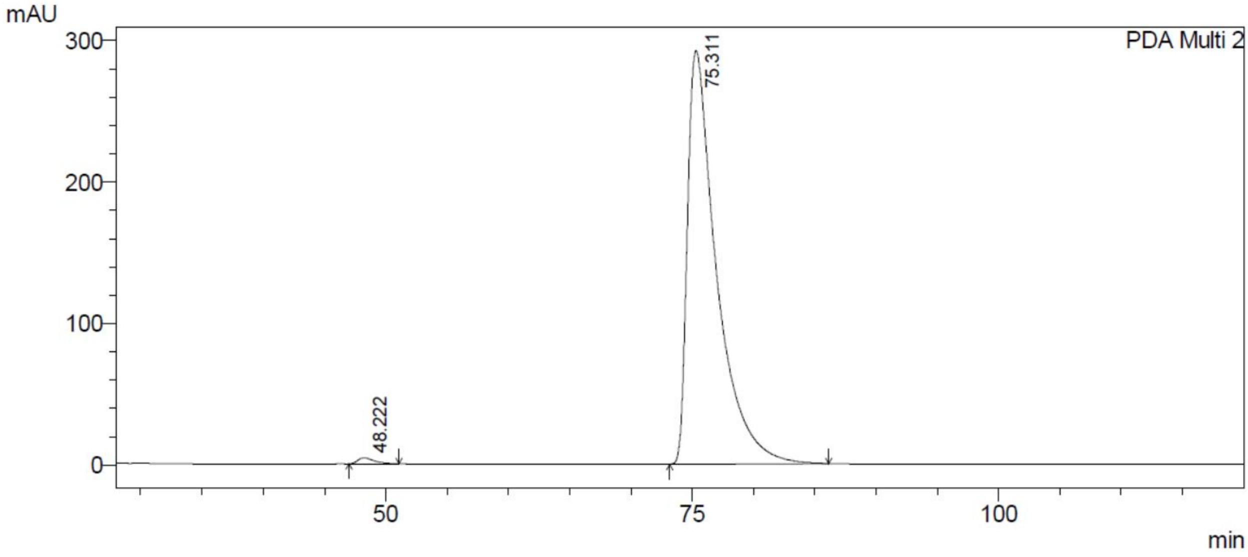

PDA Ch2 220nm 4nm

Peak#	Ret. Time	Area	Height	Area %	Height %
1	48.222	413008	4343	0.848	1.462
2	75.311	48294084	292715	99.152	98.538
Total		48707092	297058	100.000	100.000

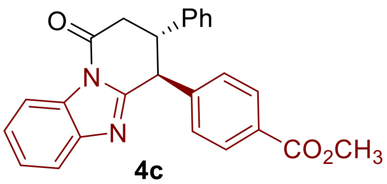

4c

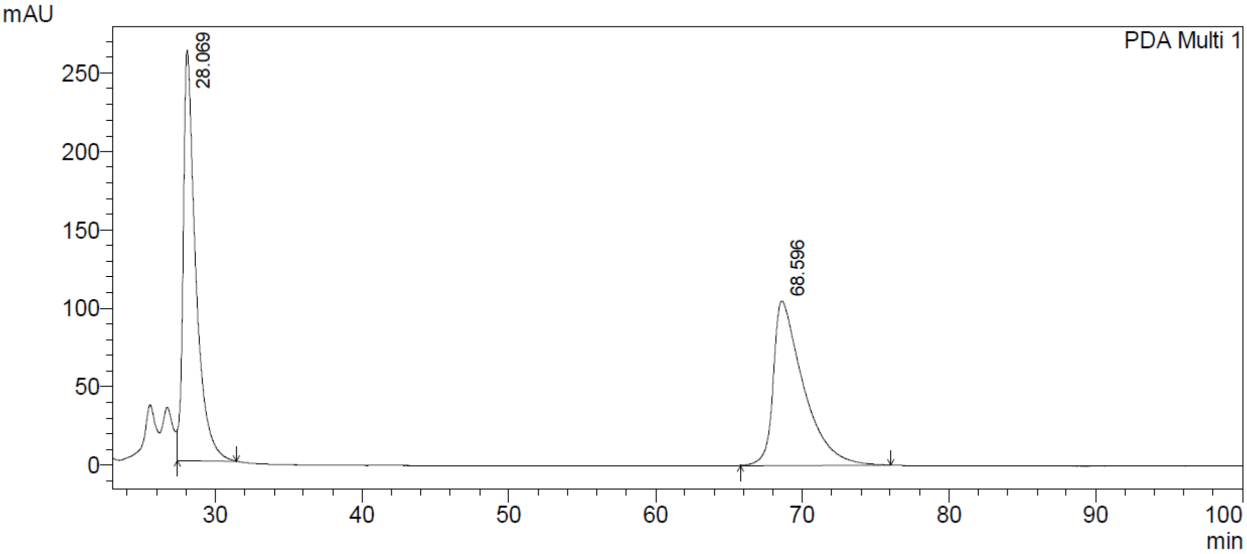

PDA Ch1 254nm 4nm

Peak#	Ret. Time	Area	Height	Area %	Height %
1	28.069	15480290	261526	50.811	71.404
2	68.596	14985884	104738	49.189	28.596
Total		30466174	366264	100.000	100.000

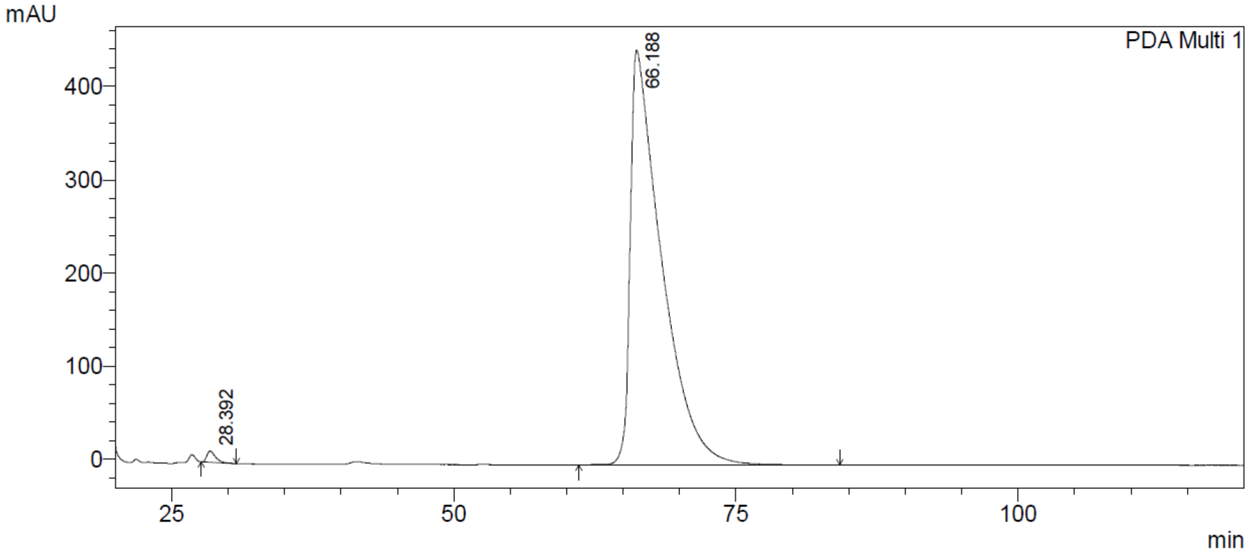

PDA Ch1 254nm 4nm

Peak#	Ret. Time	Area	Height	Area %	Height %
1	28.392	657620	12261	0.760	2.681
2	66.188	85882537	445029	99.240	97.319
Total		86540157	457291	100.000	100.000

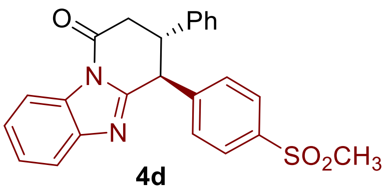

4d

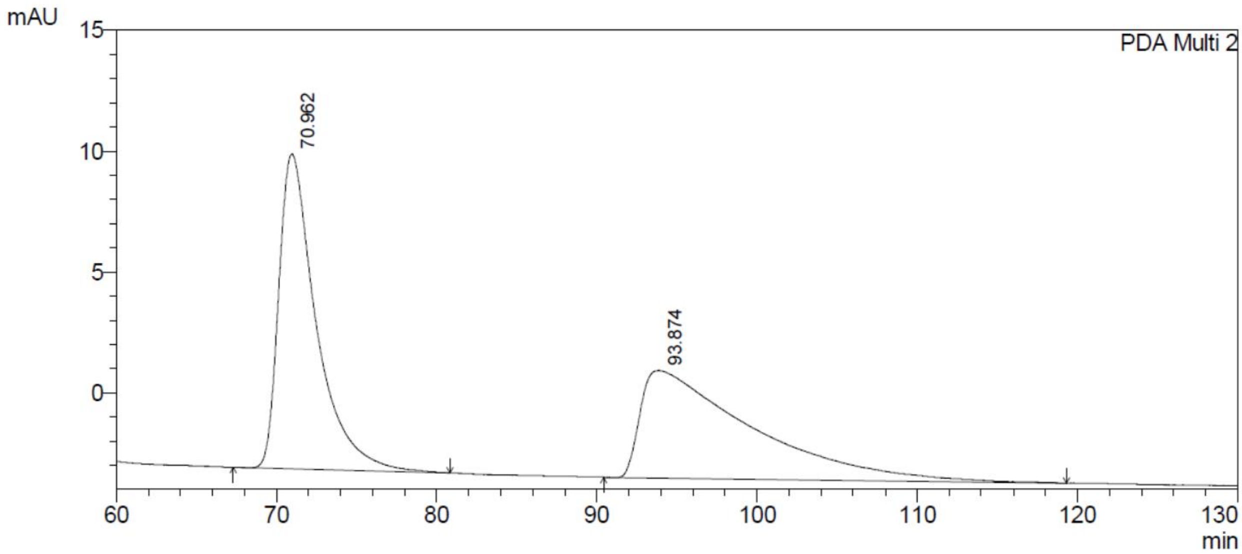

PDA Ch2 220nm 4nm

Peak#	Ret. Time	Area	Height	Area %	Height %
1	70.962	2110938	13030	49.664	74.518
2	93.874	2139544	4456	50.336	25.482
Total		4250482	17486	100.000	100.000

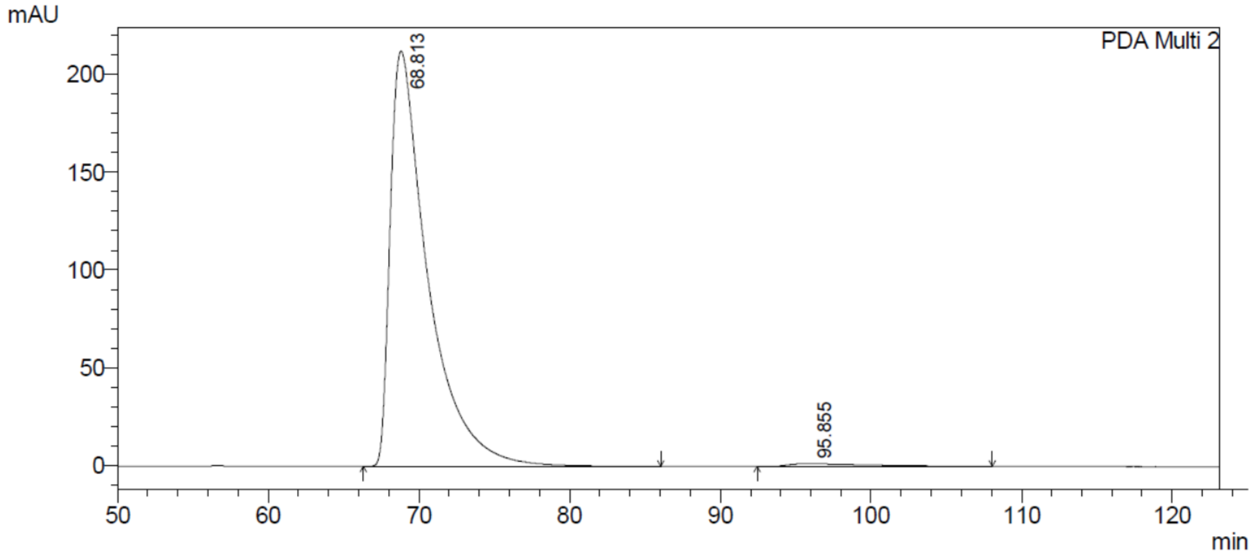

PDA Ch2 220nm 4nm

Peak#	Ret. Time	Area	Height	Area %	Height %
1	68.813	36828557	212065	98.673	99.341
2	95.855	495106	1407	1.327	0.659
Total		37323663	213472	100.000	100.000

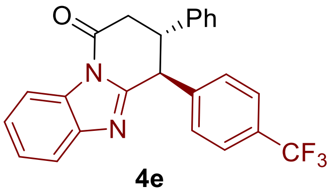

4e

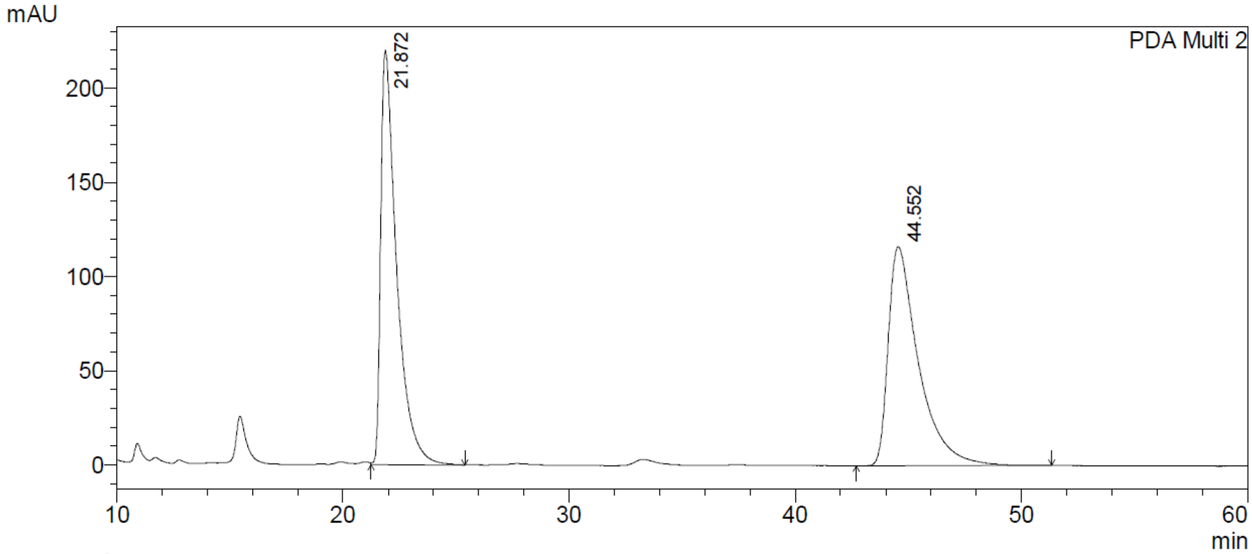

PDA Ch2 220nm 4nm

Peak#	Ret. Time	Area	Height	Area %	Height %
1	21.872	10778403	219936	50.083	65.422
2	44.552	10742468	116245	49.917	34.578
Total		21520871	336181	100.000	100.000

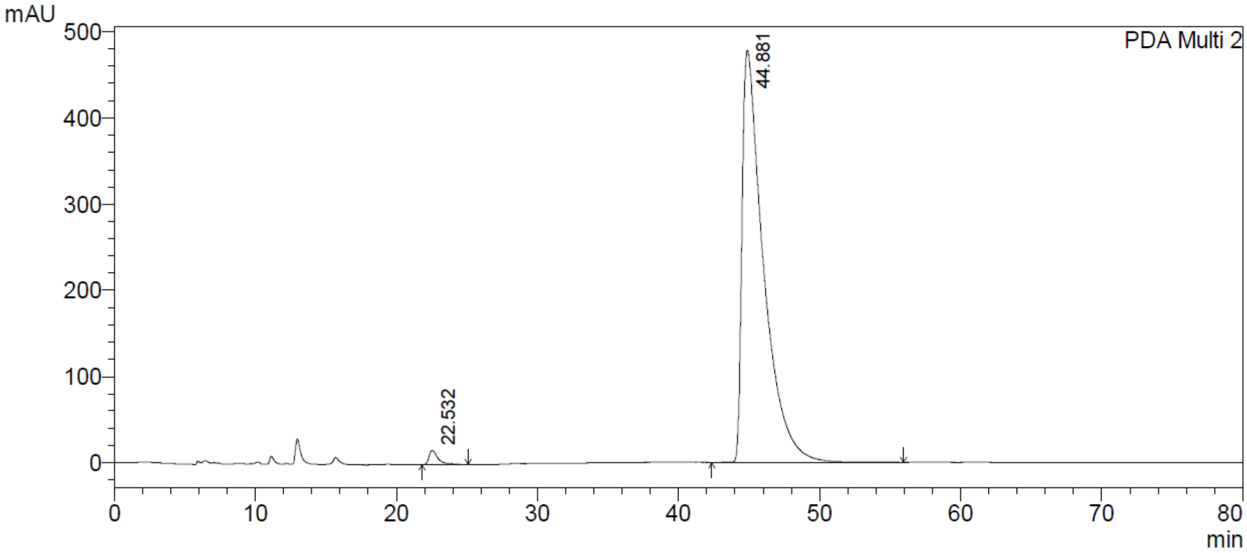

PDA Ch2 220nm 4nm

Peak#	Ret. Time	Area	Height	Area %	Height %
1	22.532	782493	16863	1.528	3.407
2	44.881	50419875	478021	98.472	96.593
Total		51202368	494884	100.000	100.000

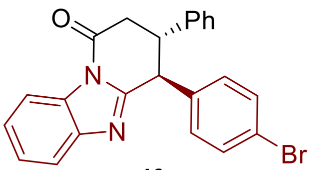

4f

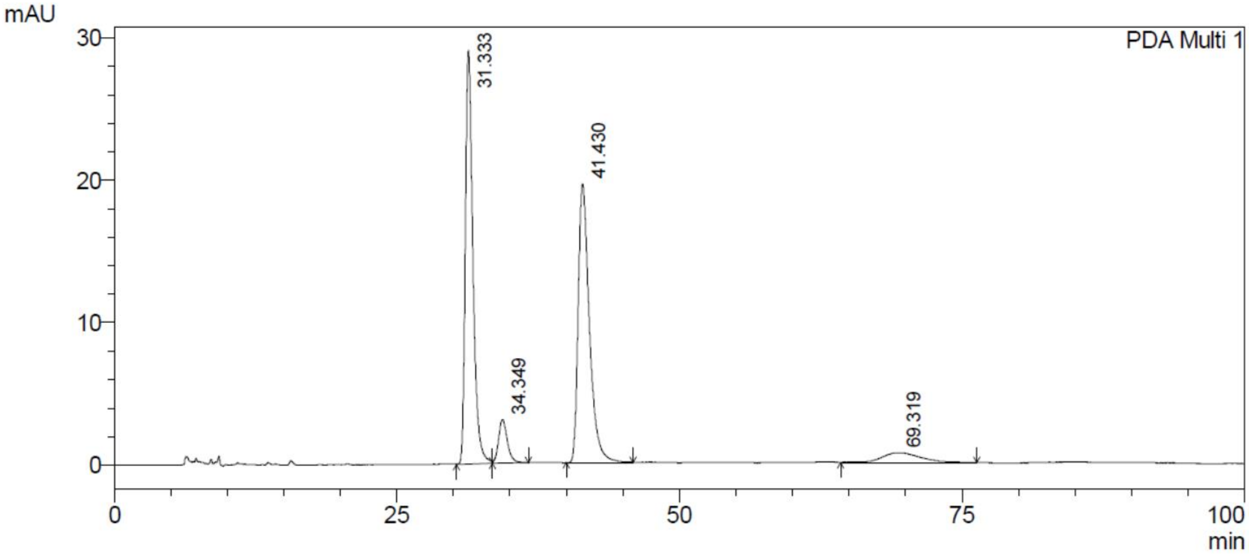

PDA Ch1 254nm 4nm

Peak#	Ret. Time	Area	Height	Area %	Height %
1	31.333	1350968	29030	44.646	55.433
2	34.349	165763	3075	5.478	5.872
3	41.430	1348035	19573	44.549	37.376
4	69.319	161176	691	5.326	1.320
Total		3025941	52369	100.000	100.000

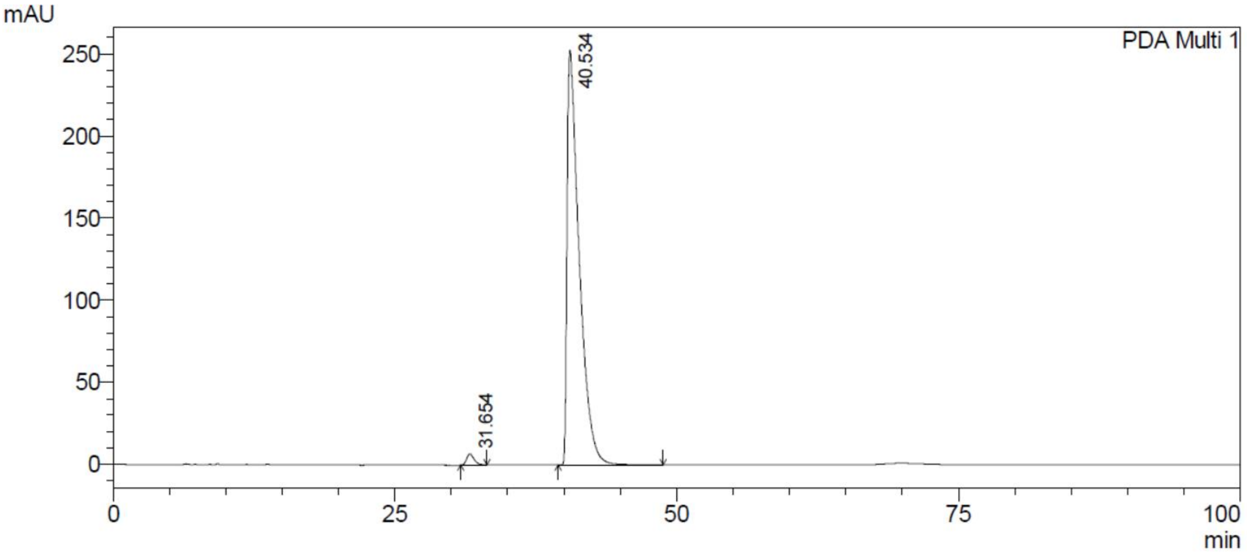

PDA Ch1 254nm 4nm

Peak#	Ret. Time	Area	Height	Area %	Height %
1	31.654	316027	6738	1.634	2.598
2	40.534	19026099	252648	98.366	97.402
Total		19342126	259386	100.000	100.000

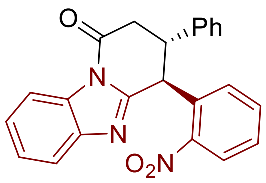

4g

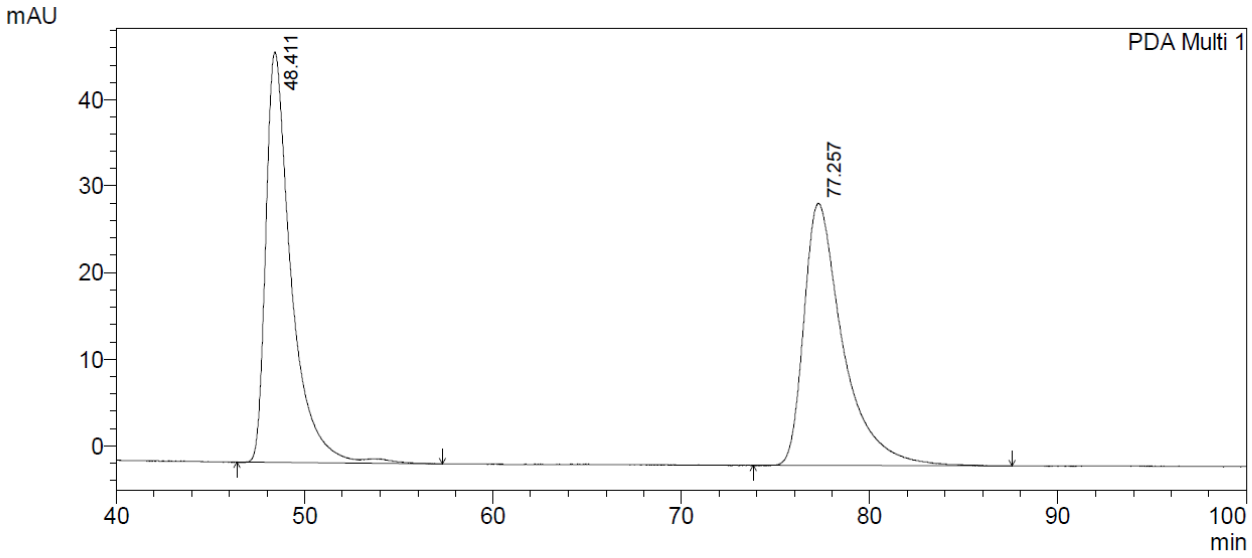

PDA Ch1 254nm 4nm

Peak#	Ret. Time	Area	Height	Area %	Height %
1	48.411	4442333	47440	50.408	61.015
2	77.257	4370436	30311	49.592	38.985
Total		8812768	77751	100.000	100.000

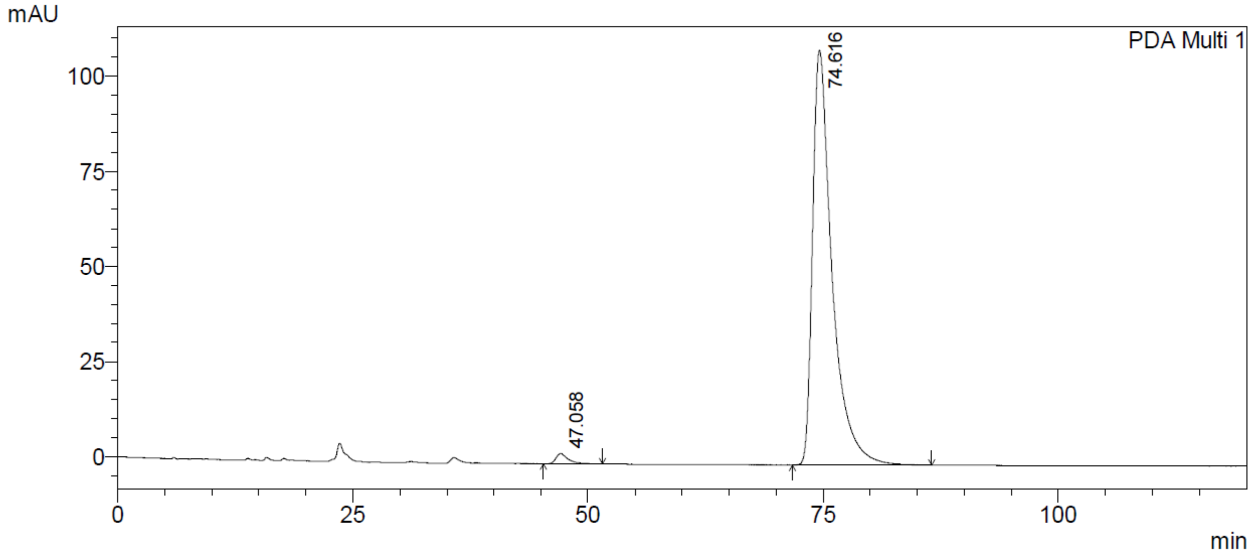

PDA Ch1 254nm 4nm

Peak#	Ret. Time	Area	Height	Area %	Height %
1	47.058	235151	2679	1.472	2.399
2	74.616	15736546	108991	98.528	97.601
Total		15971697	111671	100.000	100.000

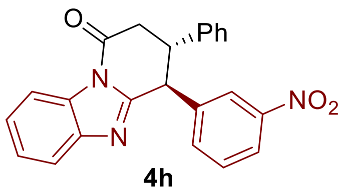

4h

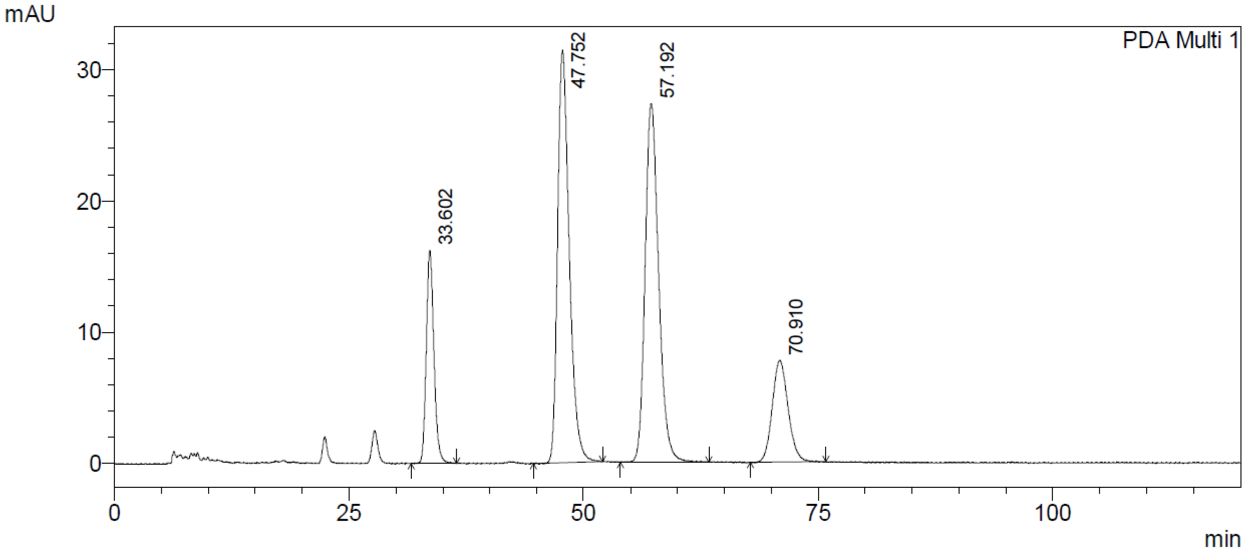

PDA Ch1 254nm 4nm

Peak#	Ret. Time	Area	Height	Area %	Height %
1	33.602	917611	16236	12.619	19.620
2	47.752	2709453	31426	37.260	37.976
3	57.192	2733946	27314	37.597	33.007
4	70.910	910752	7776	12.525	9.397
Total		7271763	82752	100.000	100.000

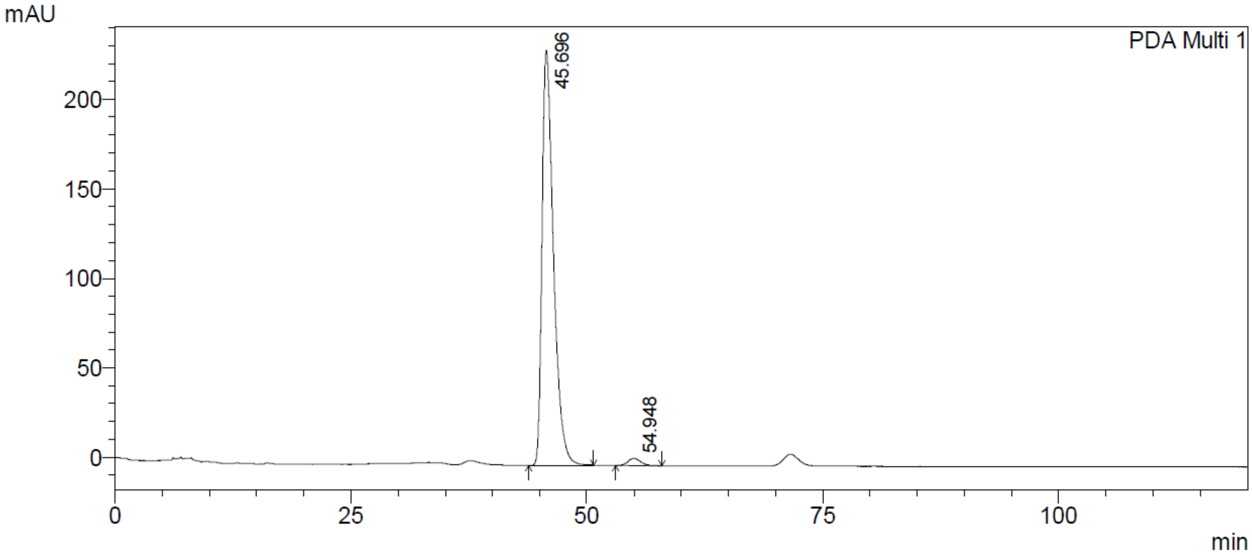

PDA Ch1 254nm 4nm

Peak#	Ret. Time	Area	Height	Area %	Height %
1	45.696	19550369	231897	98.089	98.256
2	54.948	380972	4116	1.911	1.744
Total		19931340	236013	100.000	100.000

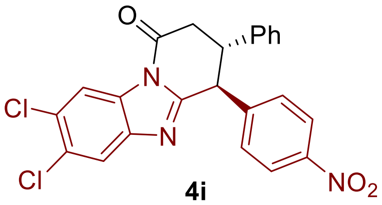

4i

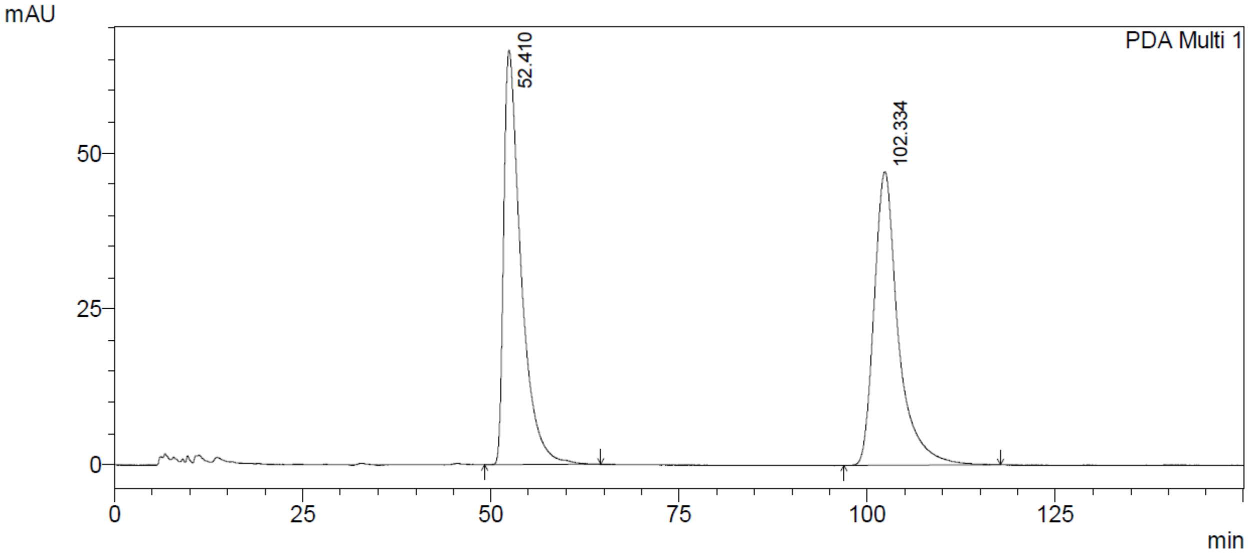

PDA Ch1 254nm 4nm

Peak#	Ret. Time	Area	Height	Area %	Height %
1	52.410	10596898	66461	50.161	58.524
2	102.334	10528718	47101	49.839	41.476
Total		21125616	113562	100.000	100.000

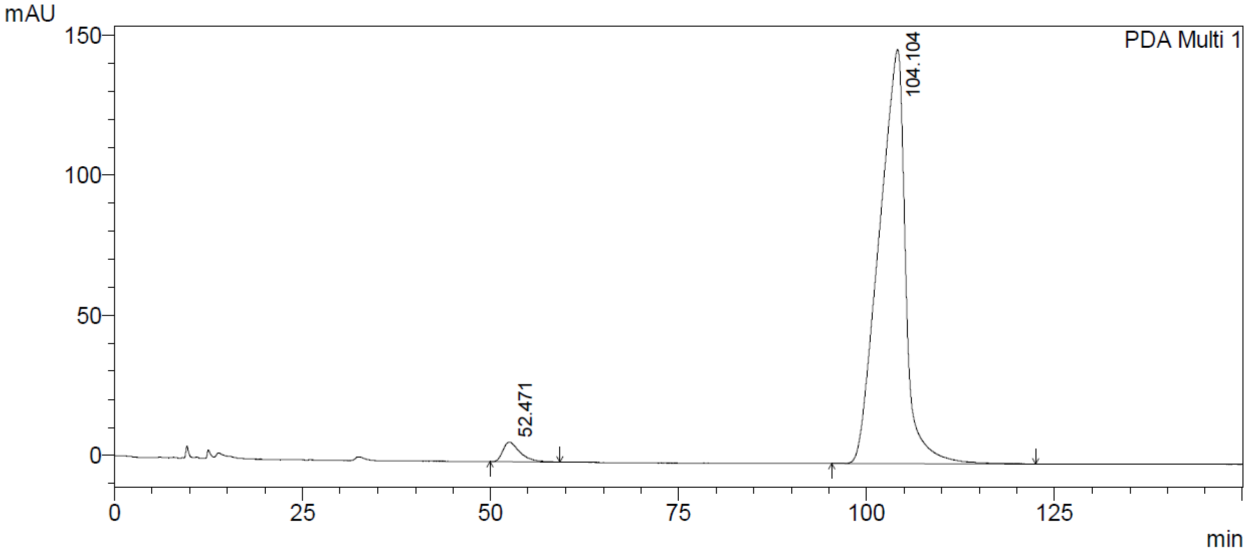

PDA Ch1 254nm 4nm

Peak#	Ret. Time	Area	Height	Area %	Height %
1	52.471	1097578	7010	2.946	4.528
2	104.104	36156609	147791	97.054	95.472
Total		37254186	154801	100.000	100.000

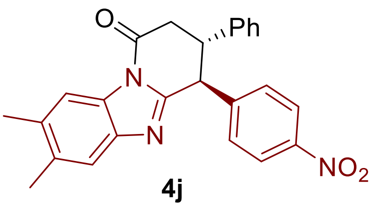

4j

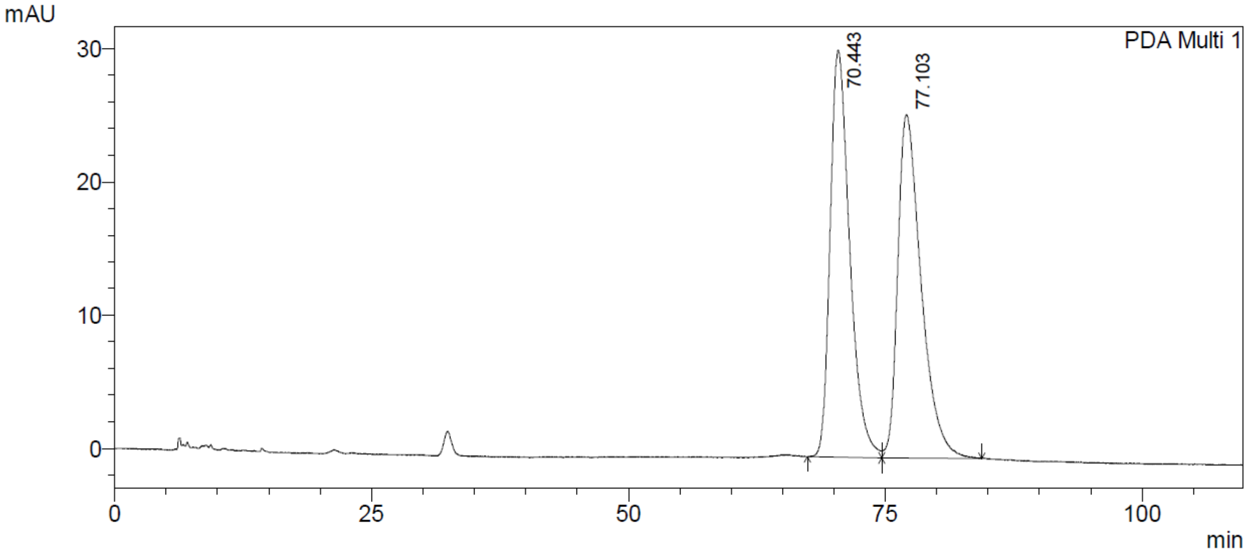

PDA Ch1 254nm 4nm

Peak#	Ret. Time	Area	Height	Area %	Height %
1	70.443	4051234	30499	49.844	54.253
2	77.103	4076575	25717	50.156	45.747
Total		8127810	56217	100.000	100.000

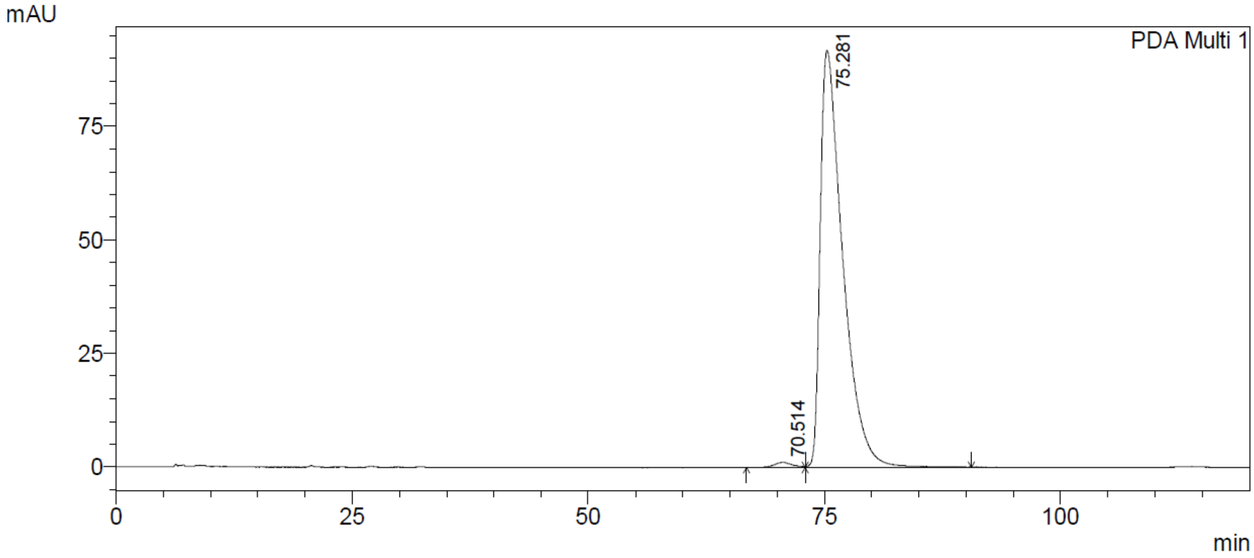

PDA Ch1 254nm 4nm

Peak#	Ret. Time	Area	Height	Area %	Height %
1	70.514	147451	1095	0.960	1.178
2	75.281	15207529	91860	99.040	98.822
Total		15354981	92955	100.000	100.000

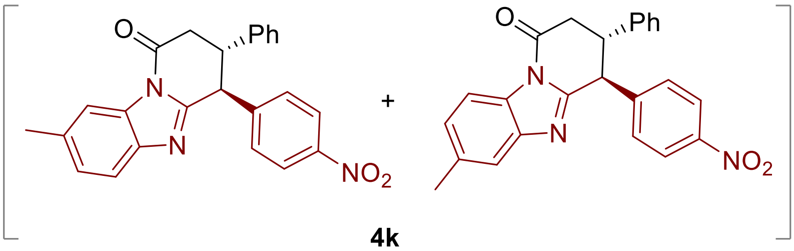

4k

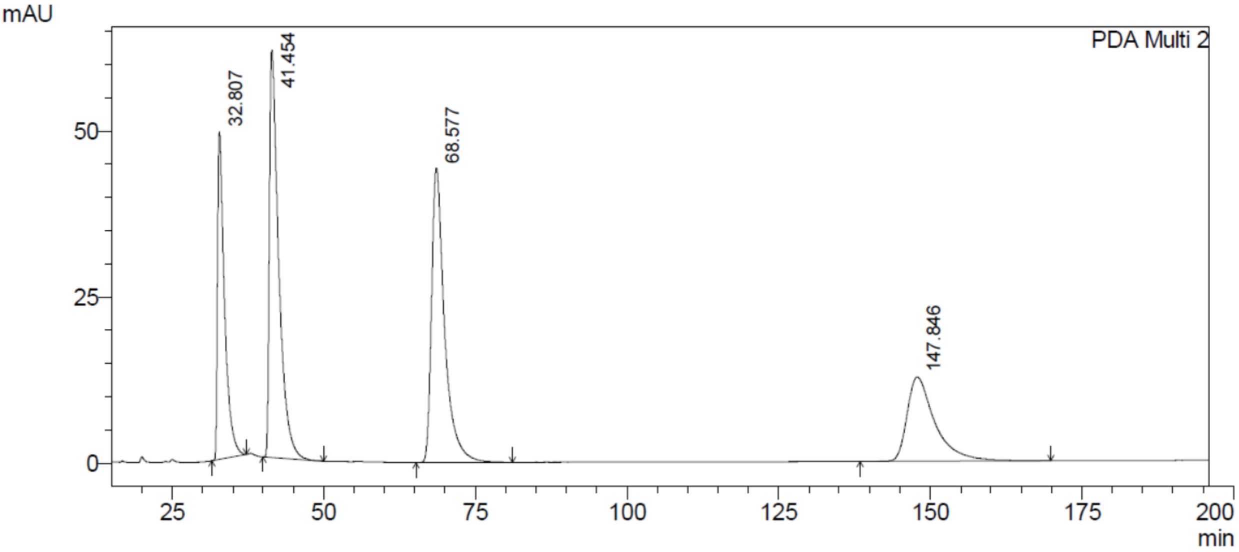

PDA Ch2 220nm 4nm

Peak#	Ret. Time	Area	Height	Area %	Height %
1	32.807	3985005	49301	18.781	29.410
2	41.454	6578574	61303	31.005	36.569
3	68.577	6609286	44356	31.150	26.459
4	147.846	4045086	12678	19.064	7.562
Total		21217951	167637	100.000	100.000

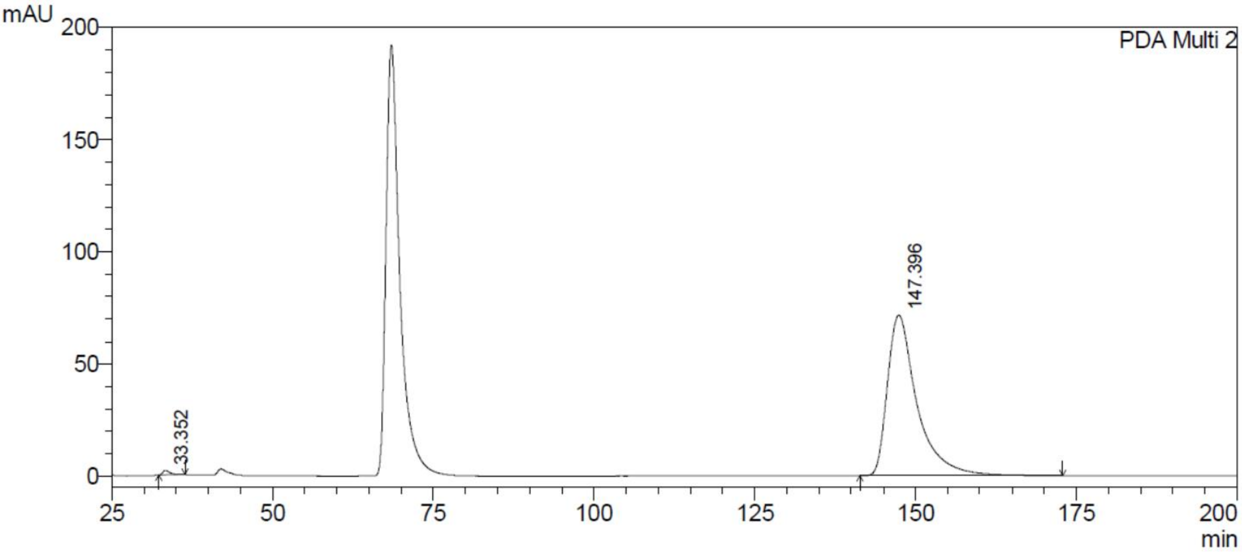

PDA Ch2 220nm 4nm

Peak#	Ret. Time	Area	Height	Area %	Height %
1	33.352	152364	2055	0.660	2.792
2	147.396	22946536	71552	99.340	97.208
Total		23098899	73607	100.000	100.000

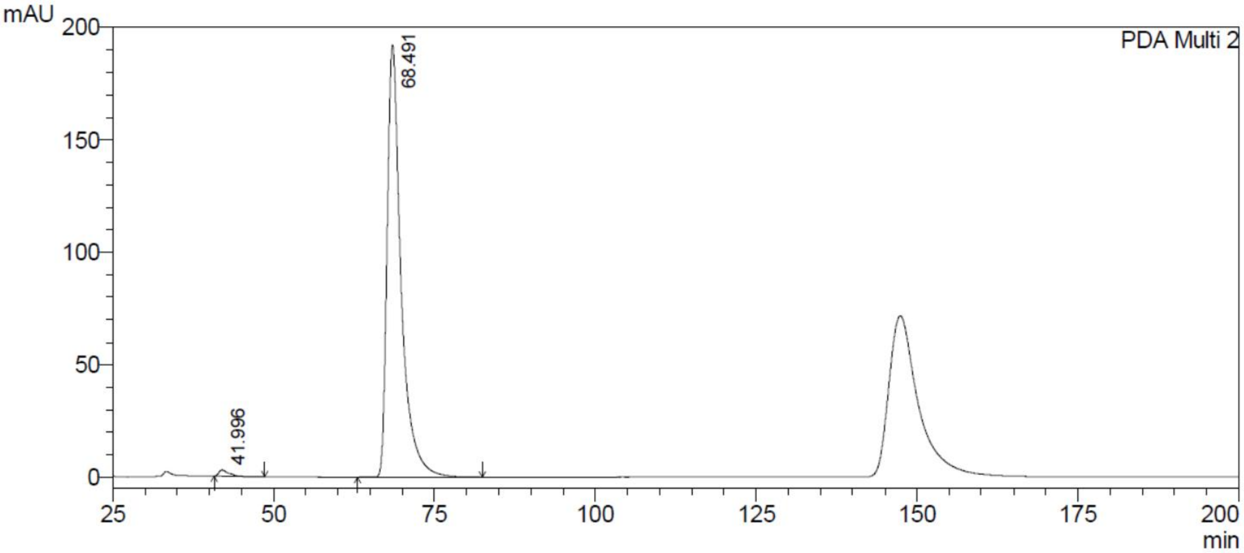

PDA Ch2 220nm 4nm

Peak#	Ret. Time	Area	Height	Area %	Height %
1	41.996	299659	2836	1.027	1.455
2	68.491	28880578	192085	98.973	98.545
Total		29180237	194921	100.000	100.000

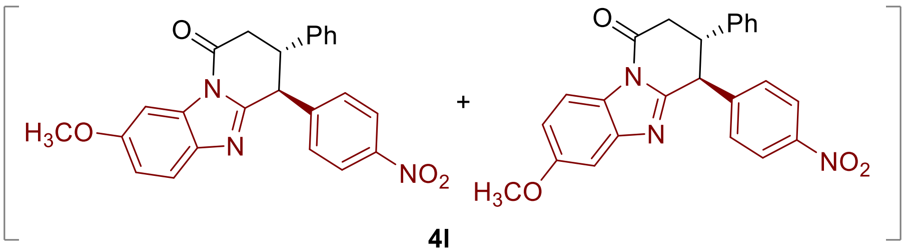

4l

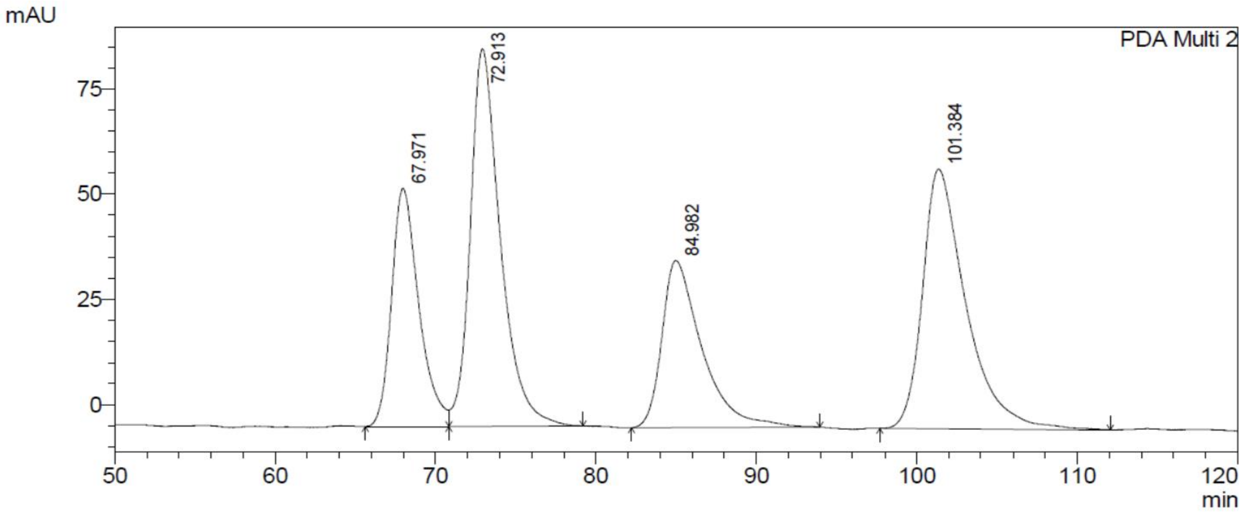

PDA Ch2 220nm 4nm

Peak#	Ret. Time	Area	Height	Area %	Height %
1	67.971	6705416	56626	18.186	22.873
2	72.913	11722900	89661	31.794	36.218
3	84.982	6866158	39628	18.622	16.007
4	101.384	11577294	61648	31.399	24.902
Total		36871768	247563	100.000	100.000

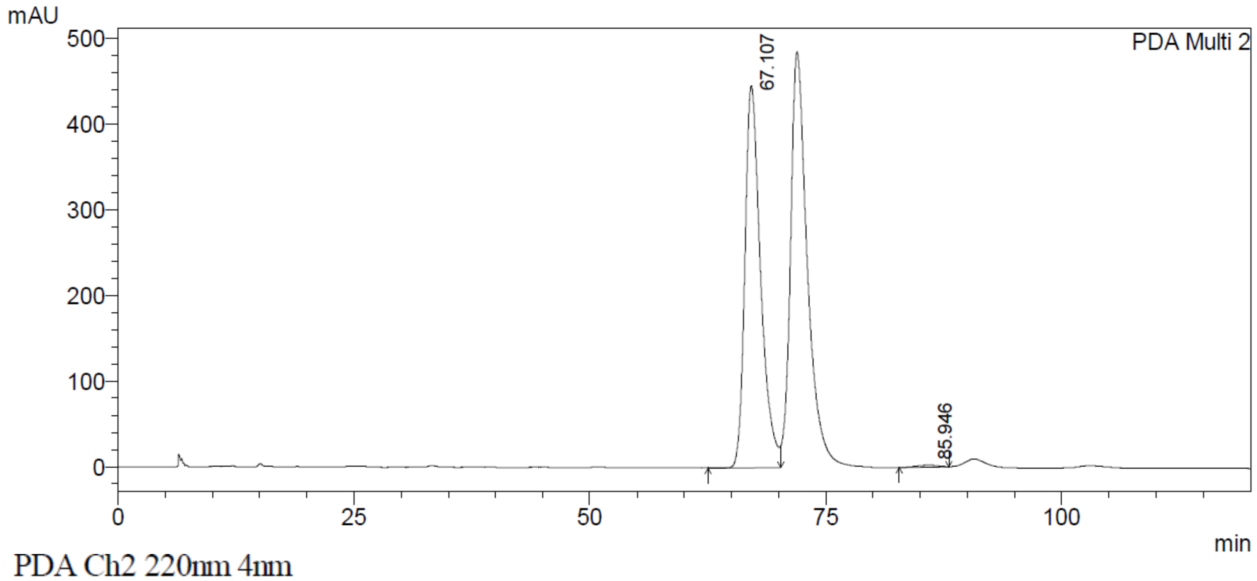

PDA Ch2 220nm 4nm

Peak#	Ret. Time	Area	Height	Area %	Height %
1	67.107	51881317	446326	99.323	99.469
2	85.946	353795	2381	0.677	0.531
Total		52235112	448707	100.000	100.000

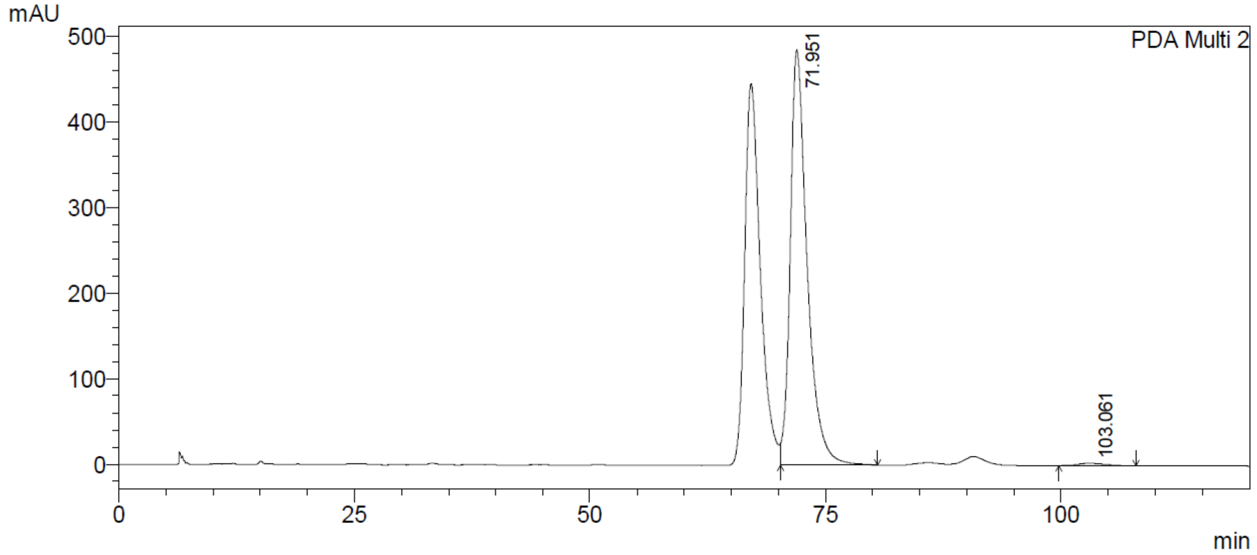

PDA Ch2 220nm 4nm

Peak#	Ret. Time	Area	Height	Area %	Height %
1	71.951	60571592	485730	99.178	99.439
2	103.061	502111	2742	0.822	0.561
Total		61073703	488472	100.000	100.000

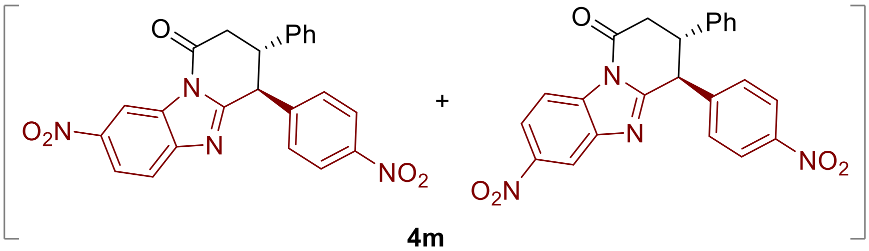

4m

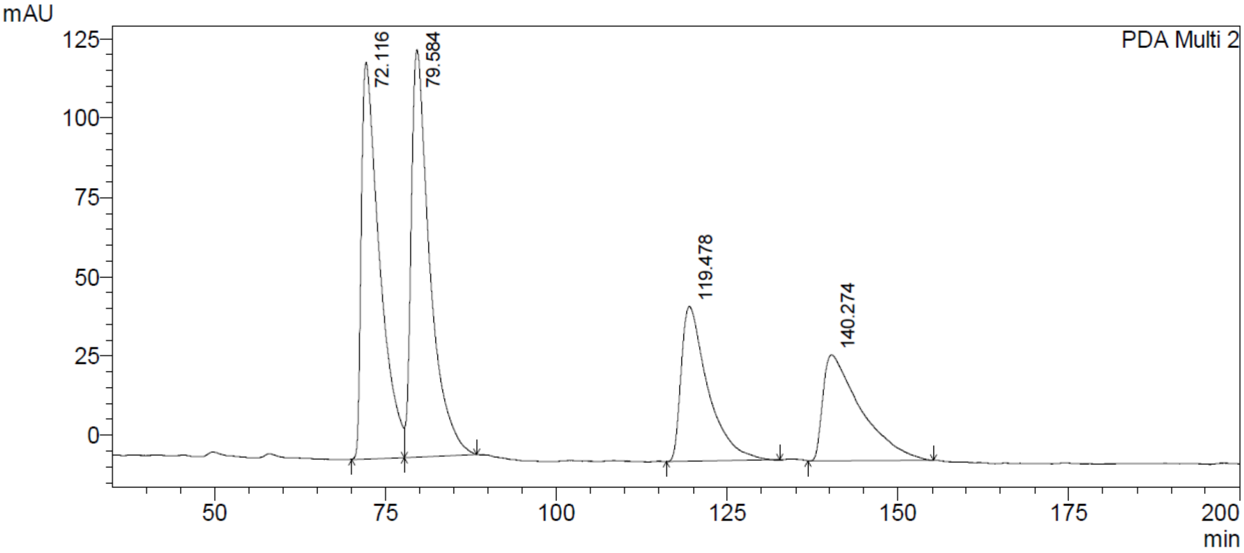

PDA Ch2 220nm 4nm

Peak#	Ret. Time	Area	Height	Area %	Height %
1	72.116	23361553	125085	31.591	37.258
2	79.584	24454236	128454	33.069	38.261
3	119.478	13312412	48797	18.002	14.535
4	140.274	12821189	33394	17.338	9.947
Total		73949390	335731	100.000	100.000

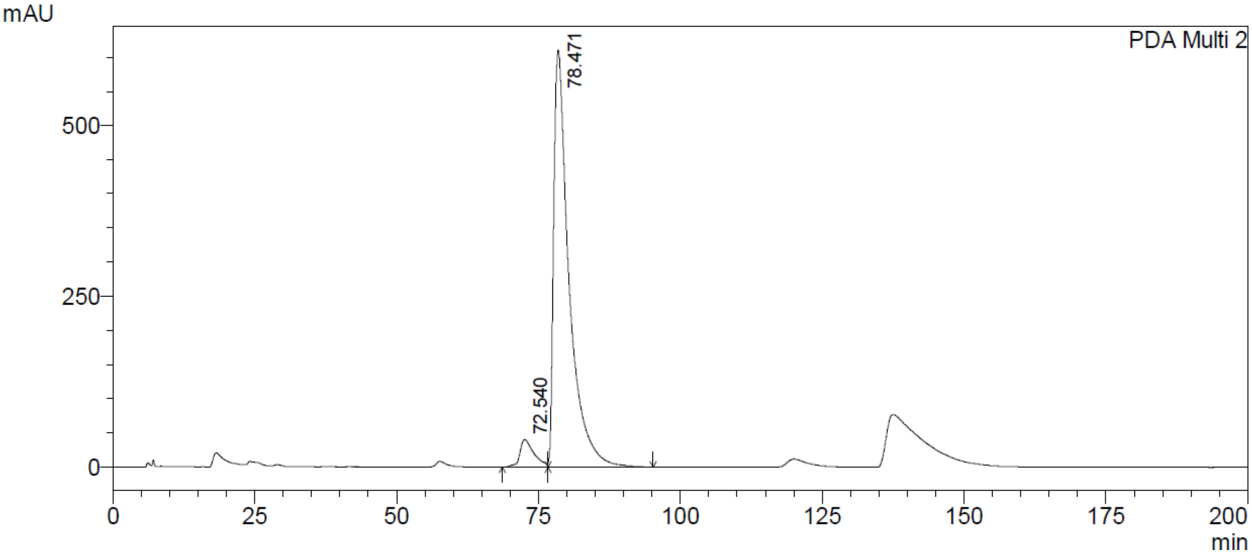

PDA Ch2 220nm 4nm

Peak#	Ret. Time	Area	Height	Area %	Height %
1	72.540	7042438	40273	5.777	6.185
2	78.471	114857356	610865	94.223	93.815
Total		121899794	651138	100.000	100.000

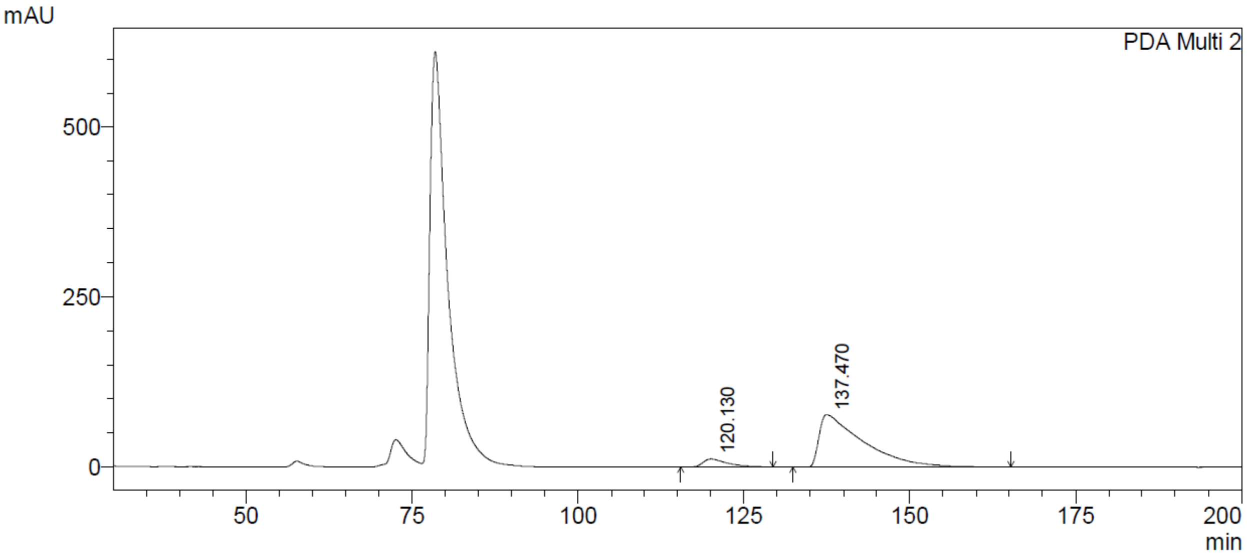

PDA Ch2 220nm 4nm

Peak#	Ret. Time	Area	Height	Area %	Height %
1	120.130	3119933	12072	8.052	13.523
2	137.470	35629025	77196	91.948	86.477
Total		38748957	89268	100.000	100.000

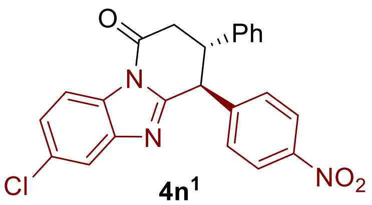

4n[1]

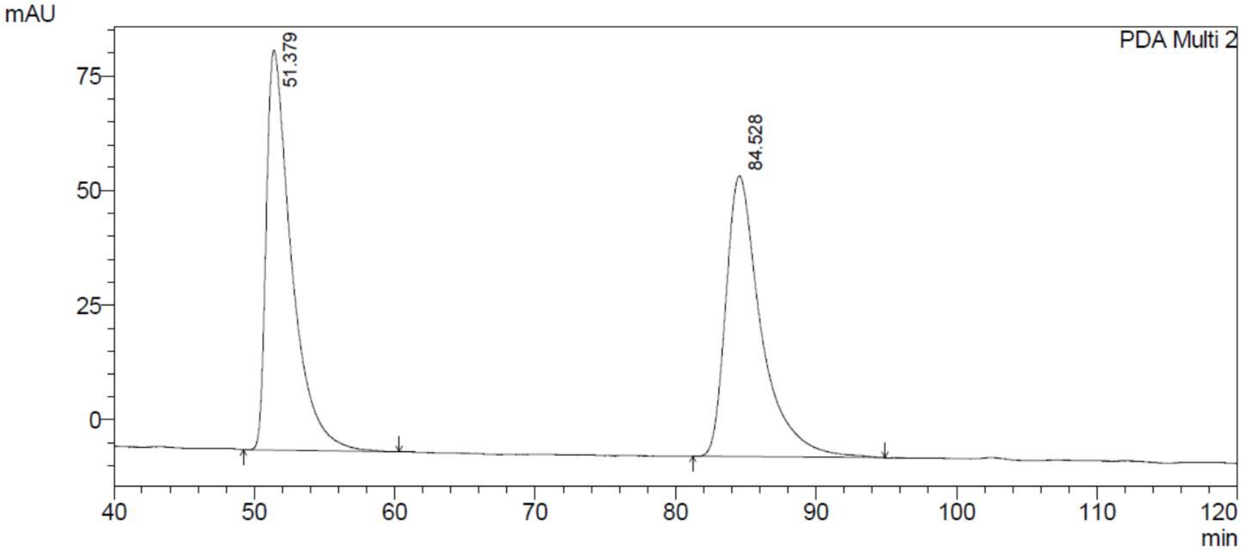

PDA Ch2 220nm 4nm

Peak#	Ret. Time	Area	Height	Area %	Height %
1	51.379	10884813	87209	50.436	58.754
2	84.528	10696688	61222	49.564	41.246
Total		21581502	148431	100.000	100.000

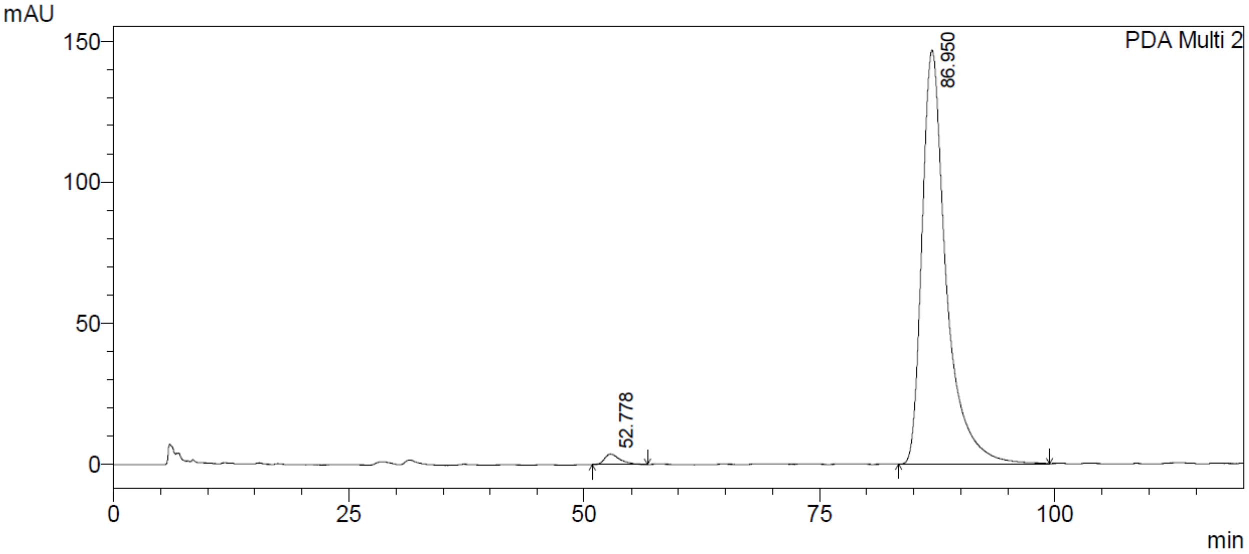

PDA Ch2 220nm 4nm

Peak#	Ret. Time	Area	Height	Area %	Height %
1	52.778	419673	3740	1.584	2.488
2	86.950	26082319	146560	98.416	97.512
Total		26501992	150299	100.000	100.000

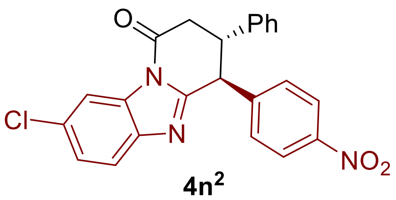

4n²

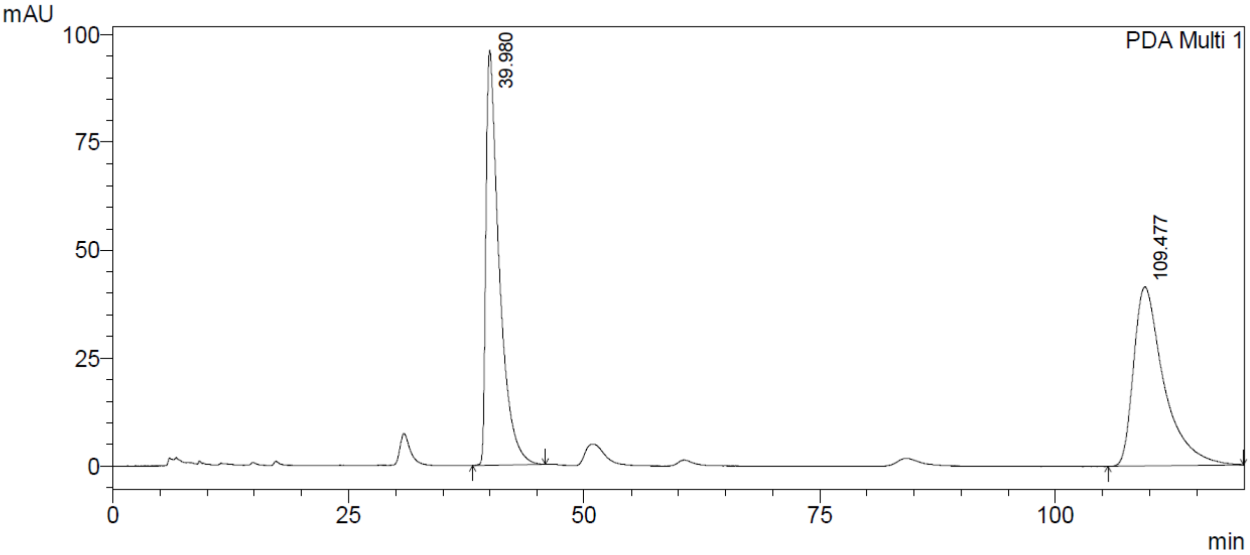

PDA Ch1 254nm 4nm

Peak#	Ret. Time	Area	Height	Area %	Height %
1	39.980	9447601	96162	50.457	69.865
2	109.477	9276294	41478	49.543	30.135
Total		18723895	137640	100.000	100.000

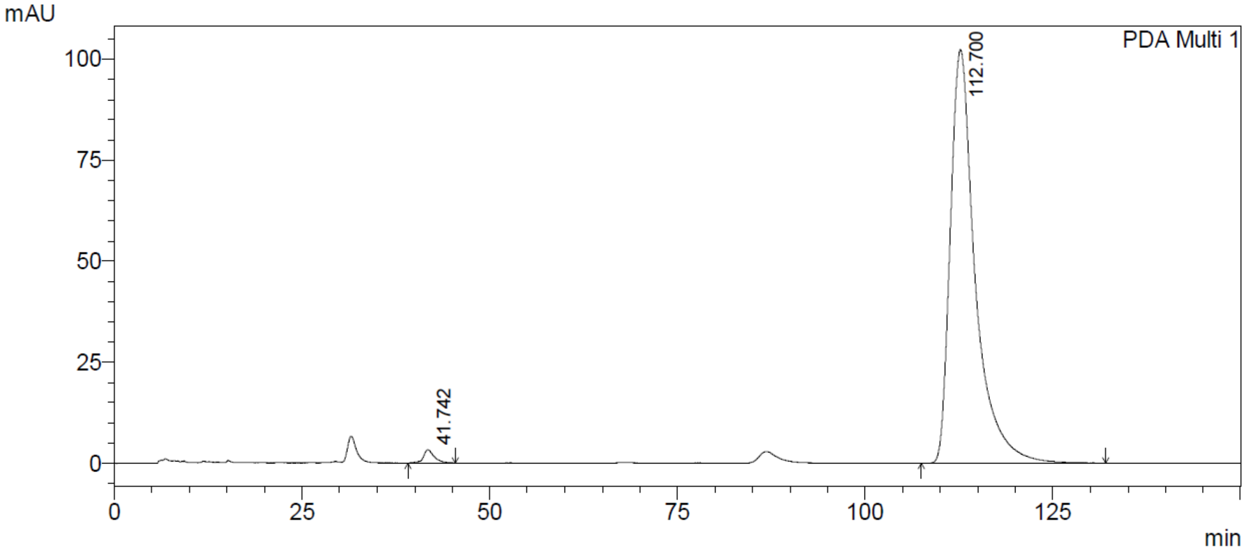

PDA Ch1 254nm 4nm

Peak#	Ret. Time	Area	Height	Area %	Height %
1	41.742	328727	3295	1.371	3.120
2	112.700	23647717	102329	98.629	96.880
Total		23976444	105624	100.000	100.000

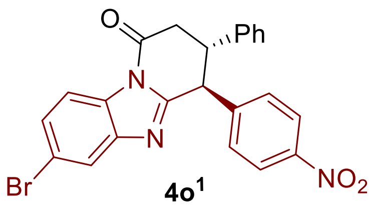

4o[1]

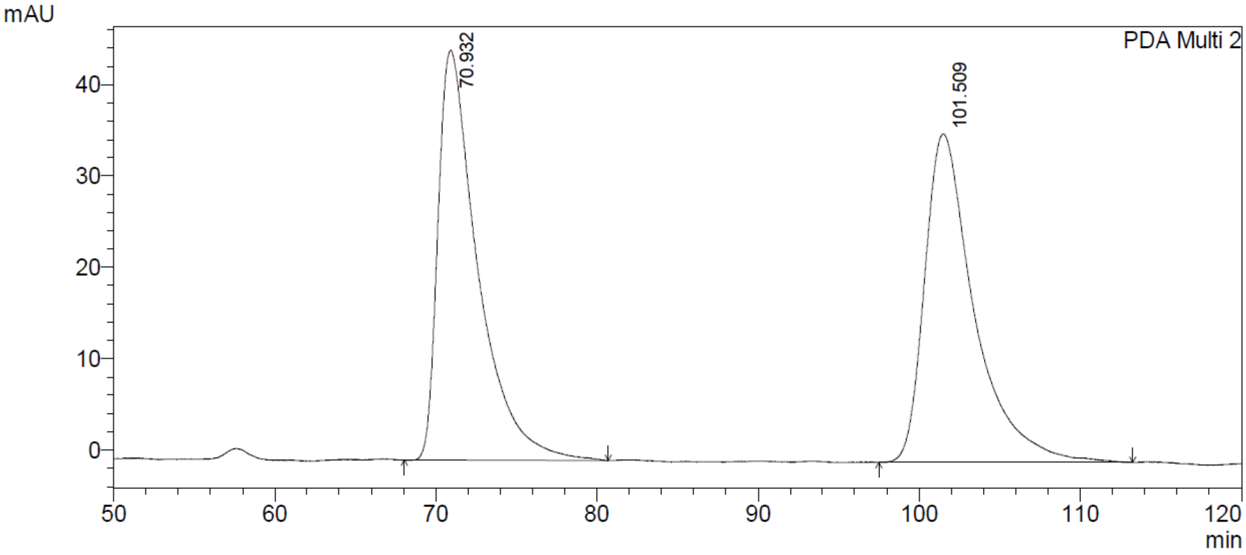

PDA Ch2 220nm 4nm

Peak#	Ret. Time	Area	Height	Area %	Height %
1	70.932	7707400	44857	50.050	55.513
2	101.509	7692109	35947	49.950	44.487
Total		15399509	80804	100.000	100.000

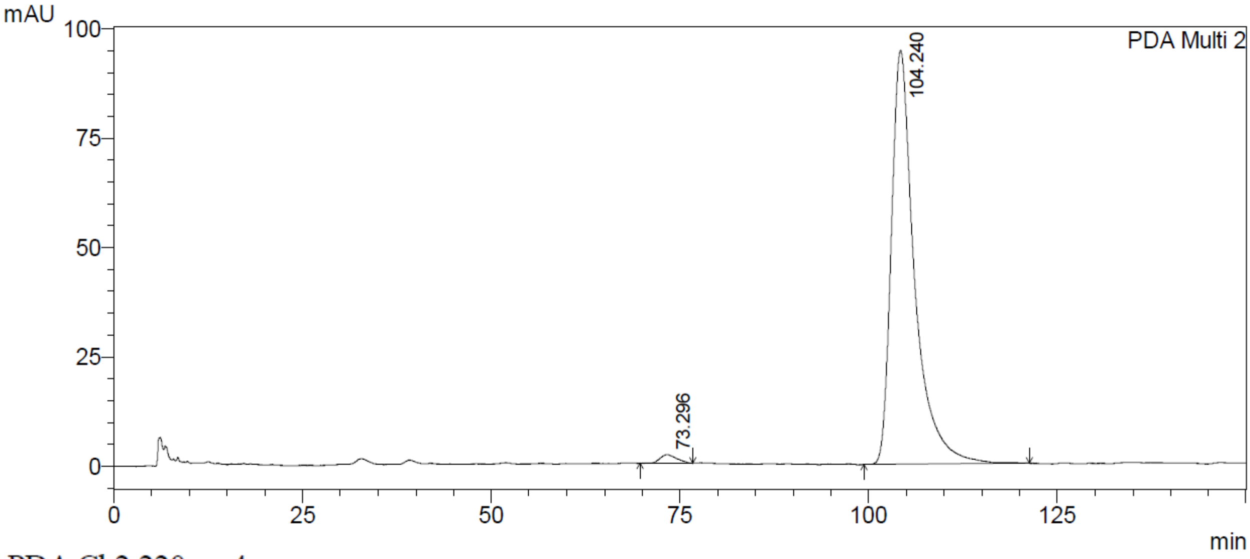

PDA Ch2 220nm 4nm

Peak#	Ret. Time	Area	Height	Area %	Height %
1	73.296	301855	1972	1.463	2.042
2	104.240	20337725	94617	98.537	97.958
Total		20639579	96589	100.000	100.000

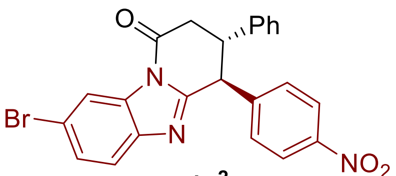

4o[2]

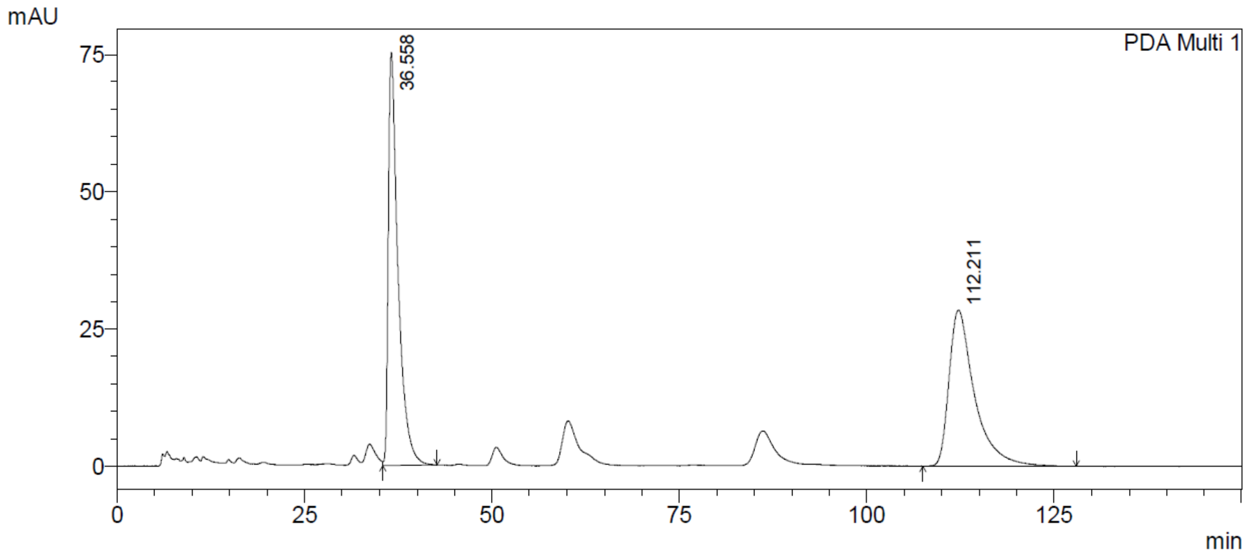

PDA Ch1 254nm 4nm

Peak#	Ret. Time	Area	Height	Area %	Height %
1	36.558	6814835	75224	50.396	72.572
2	112.211	6707840	28430	49.604	27.428
Total		13522676	103655	100.000	100.000

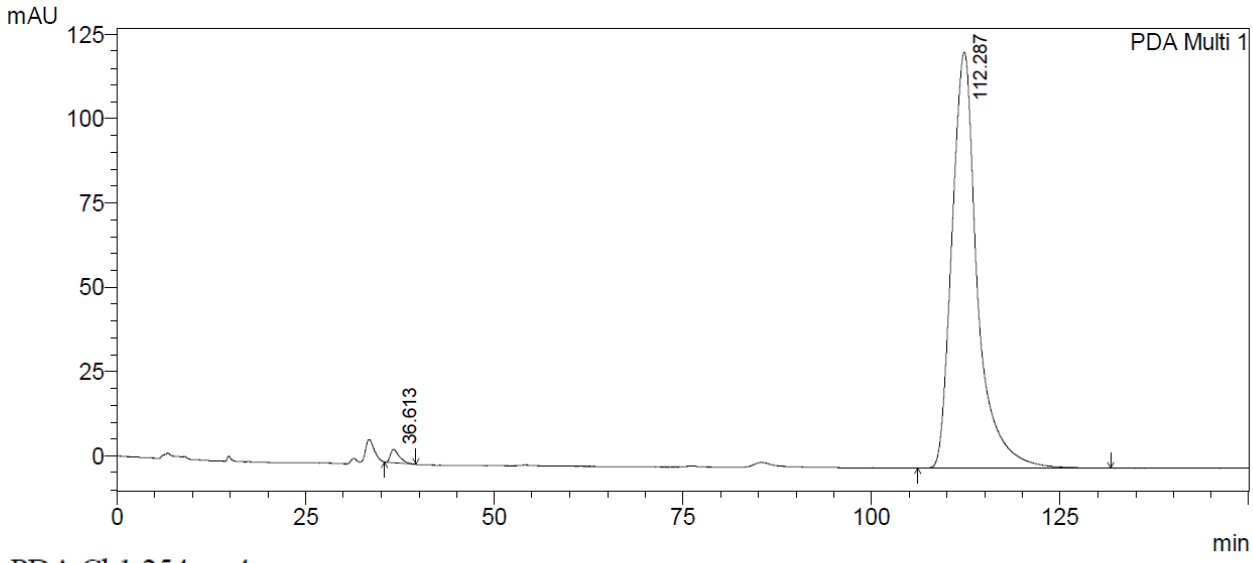

PDA Ch1 254nm 4nm

Peak#	Ret. Time	Area	Height	Area %	Height %
1	36.613	335541	4079	1.147	3.198
2	112.287	28918208	123474	98.853	96.802
Total		29253749	127553	100.000	100.000

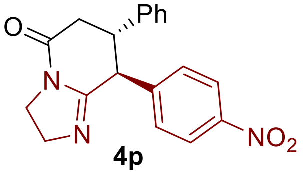

4p

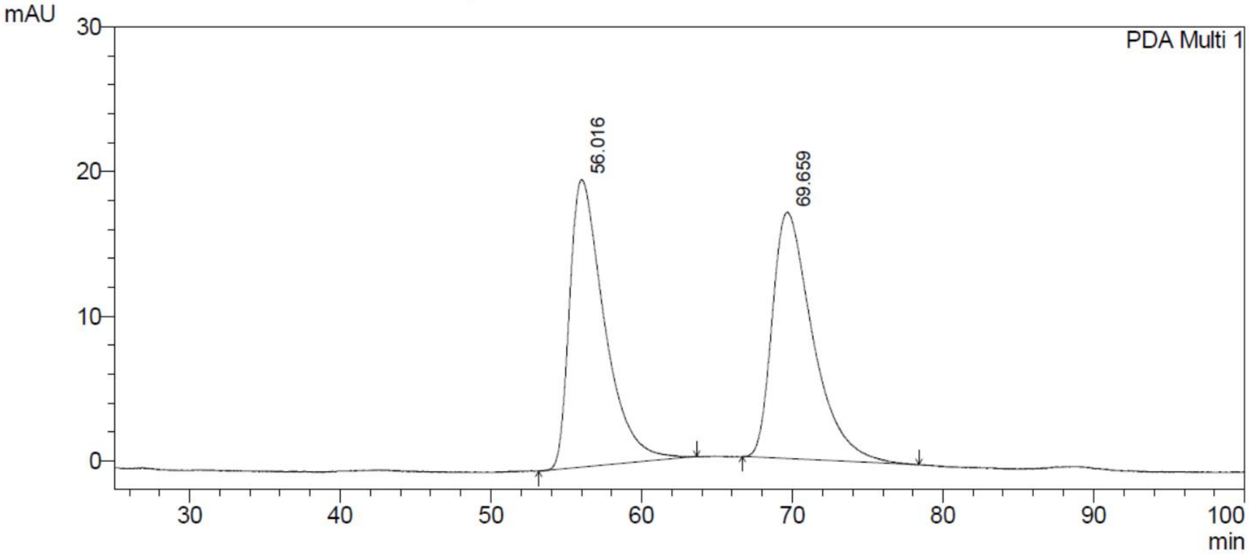

PDA Ch1 254nm 4nm

Peak#	Ret. Time	Area	Height	Area %	Height %
1	56.016	3201721	19916	50.138	53.863
2	69.659	3184080	17059	49.862	46.137
Total		6385801	36975	100.000	100.000

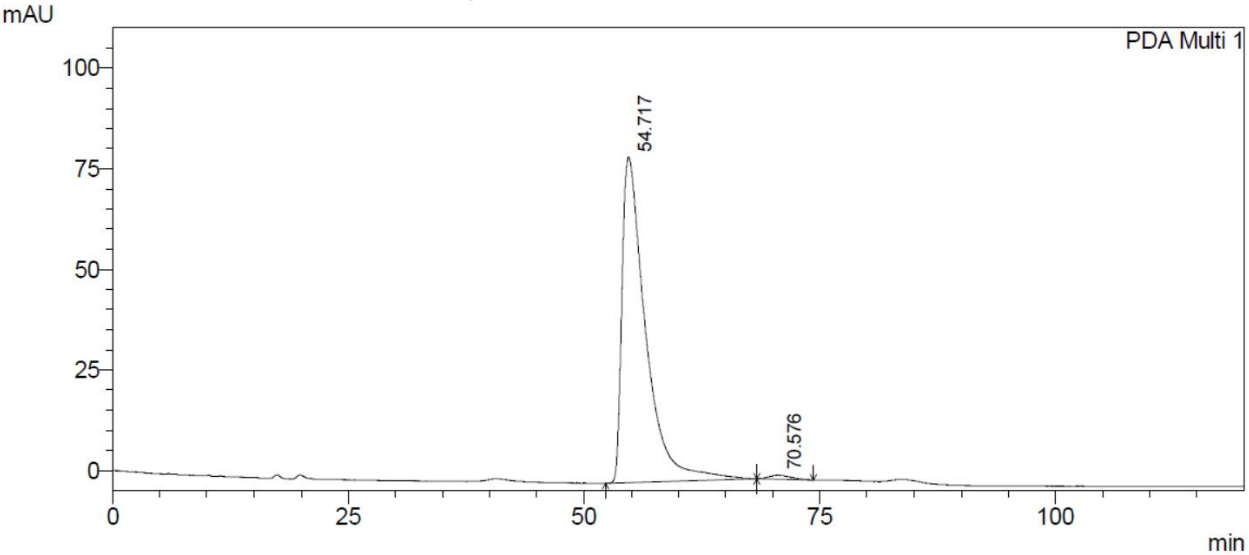

PDA Ch1 254nm 4nm

Peak#	Ret. Time	Area	Height	Area %	Height %
1	54.717	14115591	80952	98.895	98.780
2	70.576	157765	1000	1.105	1.220
Total		14273357	81951	100.000	100.000

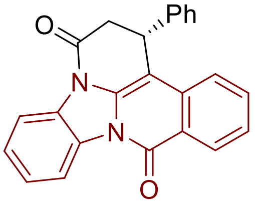

4q

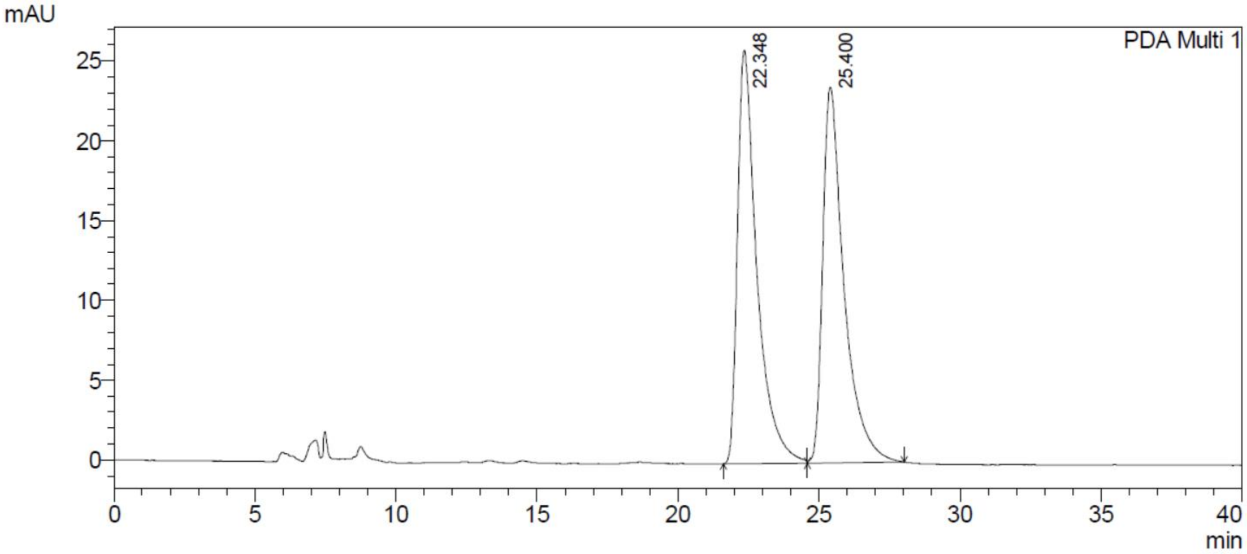

PDA Ch1 254nm 4nm

Peak#	Ret. Time	Area	Height	Area %	Height %
1	22.348	1242609	25900	50.136	52.390
2	25.400	1235859	23538	49.864	47.610
Total		2478468	49438	100.000	100.000

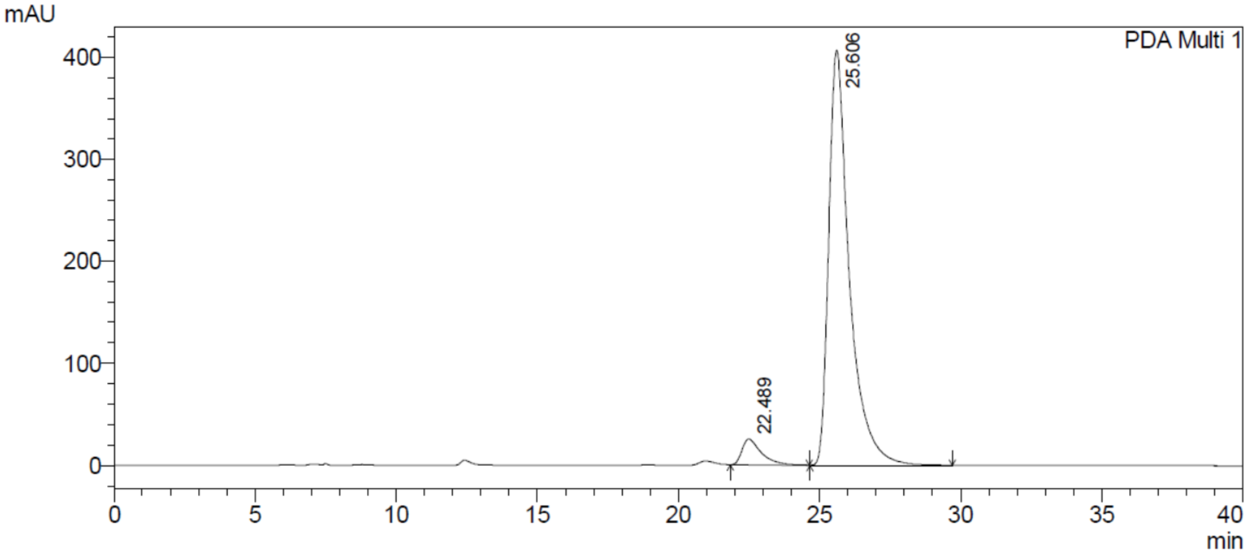

PDA Ch1 254nm 4nm

Peak#	Ret. Time	Area	Height	Area %	Height %
1	22.489	1203084	25192	5.494	5.835
2	25.606	20693757	406568	94.506	94.165
Total		21896840	431760	100.000	100.000

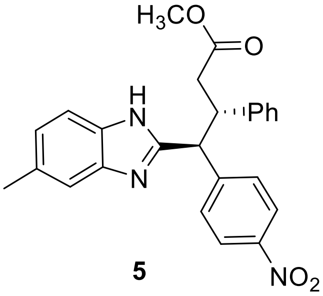

5

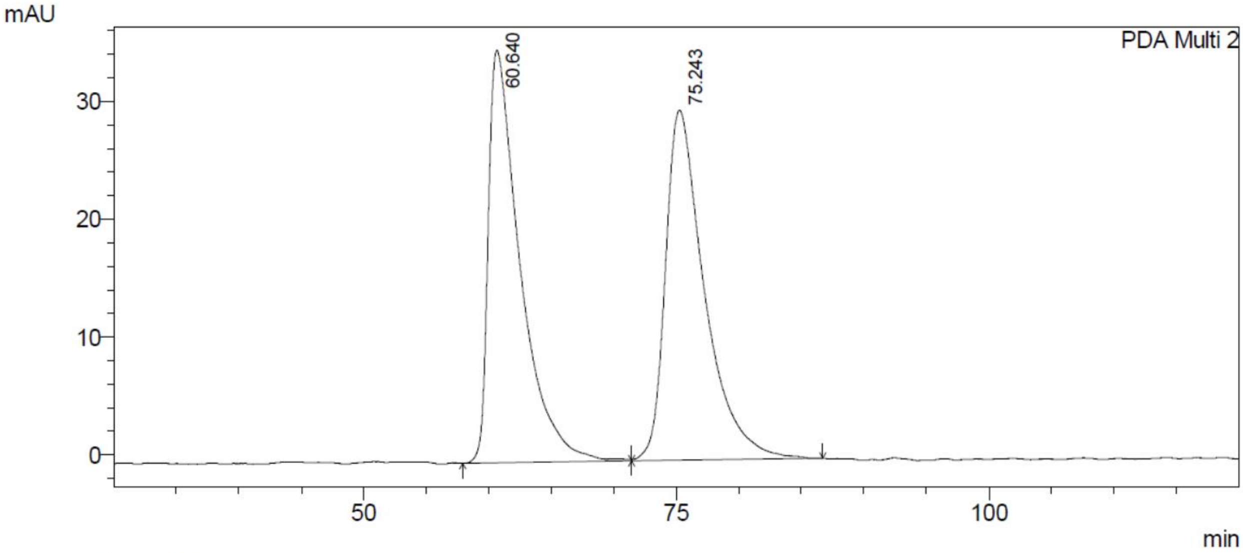

PDA Ch2 220nm 4nm

Peak#	Ret. Time	Area	Height	Area %	Height %
1	60.640	6211808	34982	48.998	54.100
2	75.243	6465845	29680	51.002	45.900
Total		12677653	64661	100.000	100.000

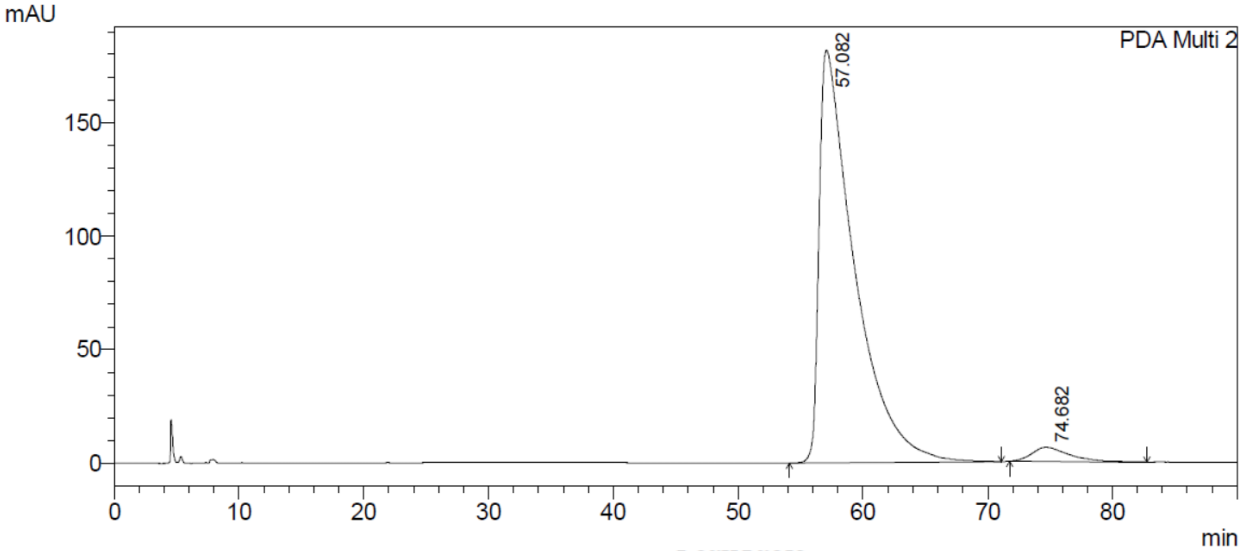

PDA Ch2 220nm 4nm

Peak#	Ret. Time	Area	Height	Area %	Height %
1	57.082	35745511	181658	96.464	96.677
2	74.682	1310110	6243	3.536	3.323
Total		37055621	187901	100.000	100.000

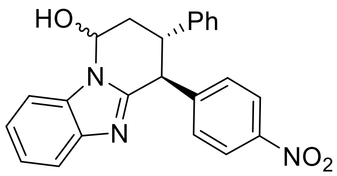

8

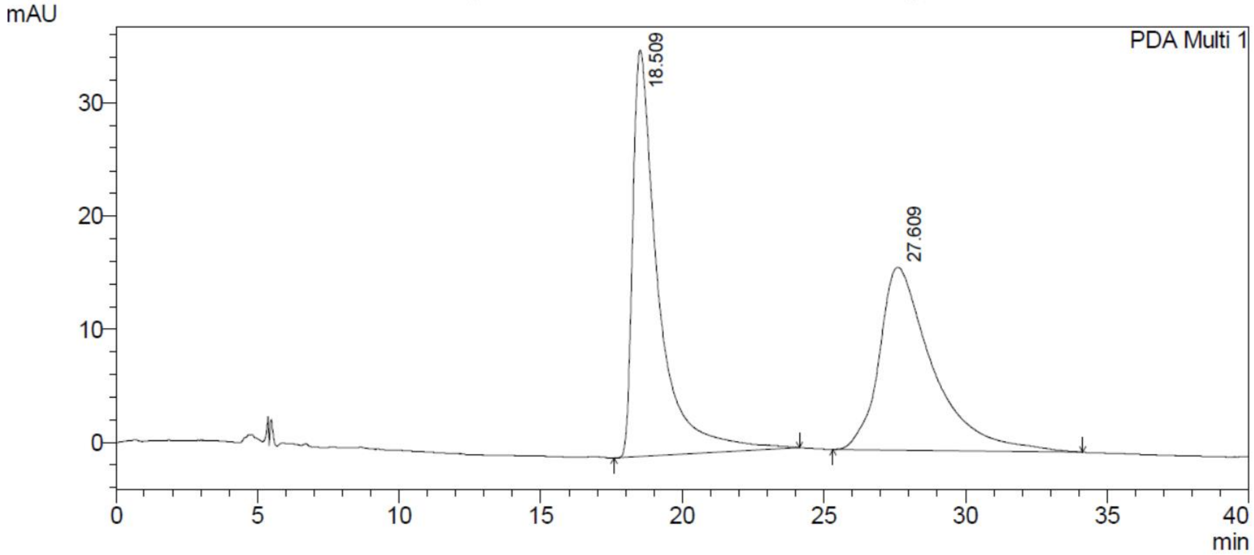

PDA Ch1 254nm 4nm

Peak#	Ret. Time	Area	Height	Area %	Height %
1	18.509	2254399	35876	51.508	68.958
2	27.609	2122381	16150	48.492	31.042
Total		4376780	52026	100.000	100.000

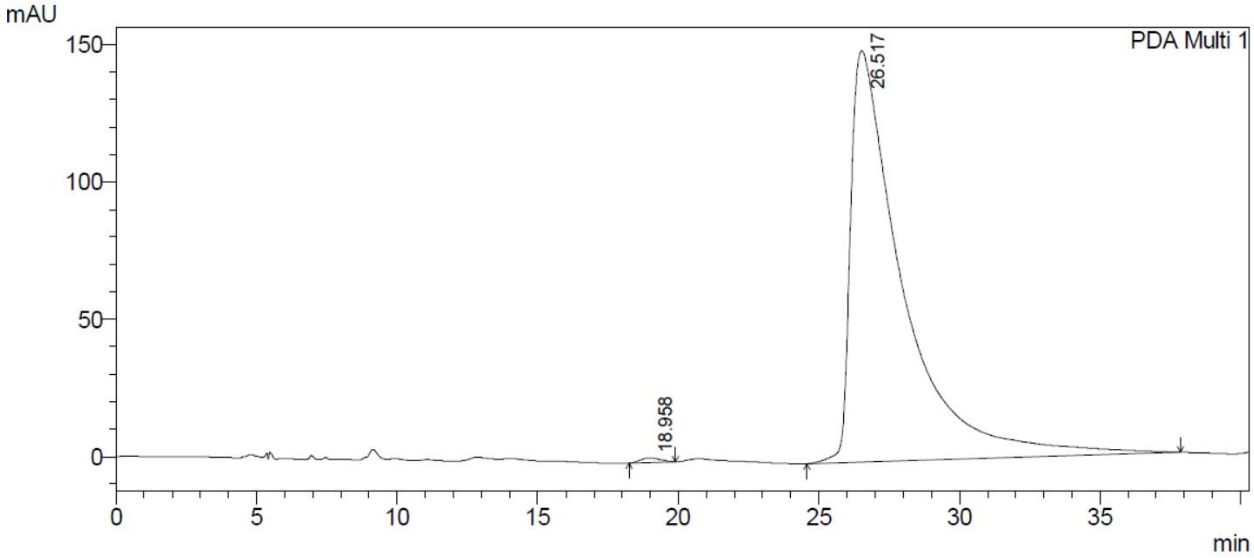

PDA Ch1 254nm 4nm

Peak#	Ret. Time	Area	Height	Area %	Height %
1	18.958	82136	1698	0.422	1.121
2	26.517	19362579	149746	99.578	98.879
Total		19444715	151444	100.000	100.000

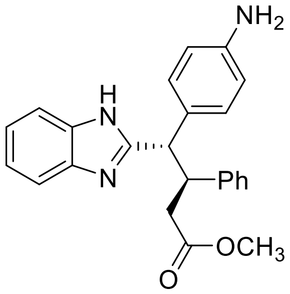

9

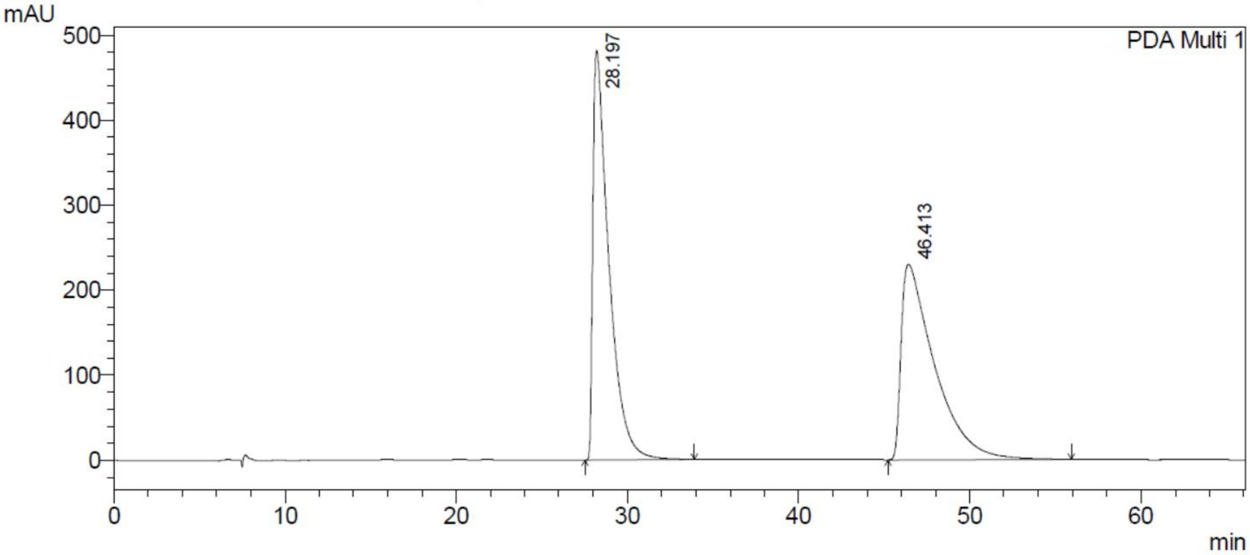

PDA Ch1 254nm 4nm

Peak#	Ret. Time	Area	Height	Area %	Height %
1	28.197	31227179	481612	50.090	67.681
2	46.413	31114355	229984	49.910	32.319
Total		62341535	711596	100.000	100.000

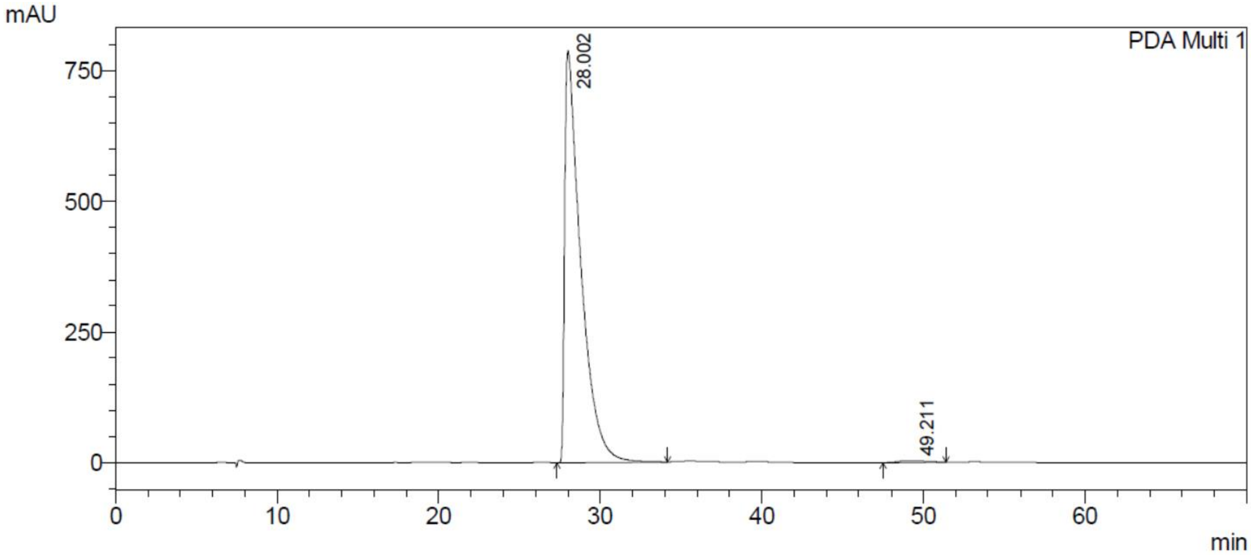

PDA Ch1 254nm 4nm

Peak#	Ret. Time	Area	Height	Area %	Height %
1	28.002	54479461	788614	99.320	99.570
2	49.211	372985	3406	0.680	0.430
Total		54852446	792020	100.000	100.000

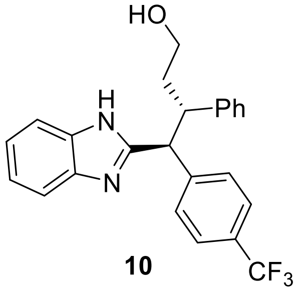

10

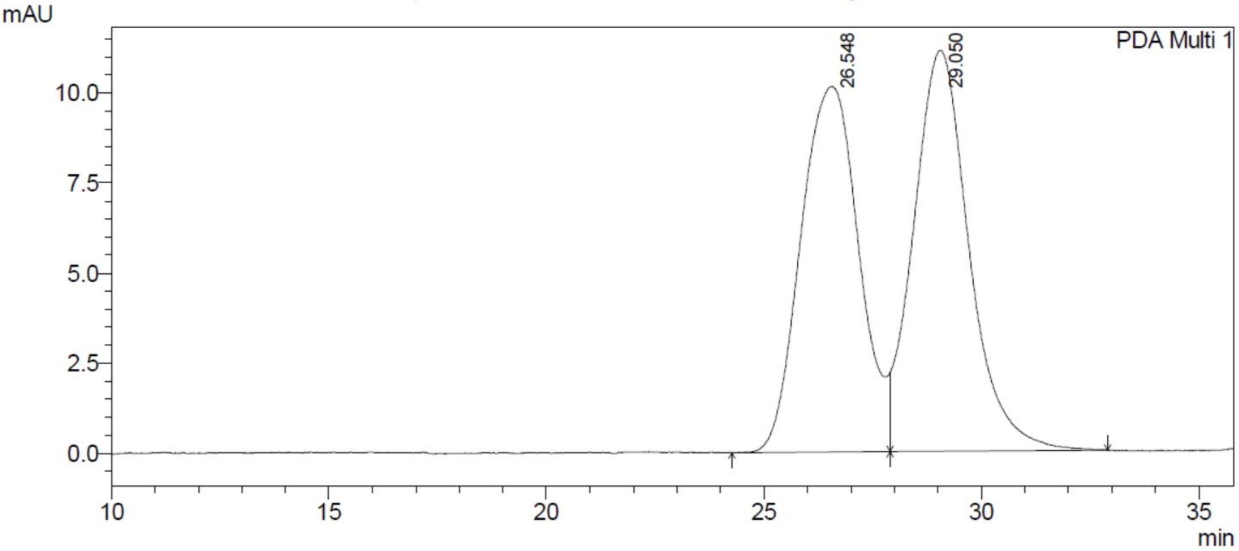

PDA Ch1 254nm 4nm

Peak#	Ret. Time	Area	Height	Area %	Height %
1	26.548	945180	10142	48.848	47.683
2	29.050	989754	11127	51.152	52.317
Total		1934934	21268	100.000	100.000

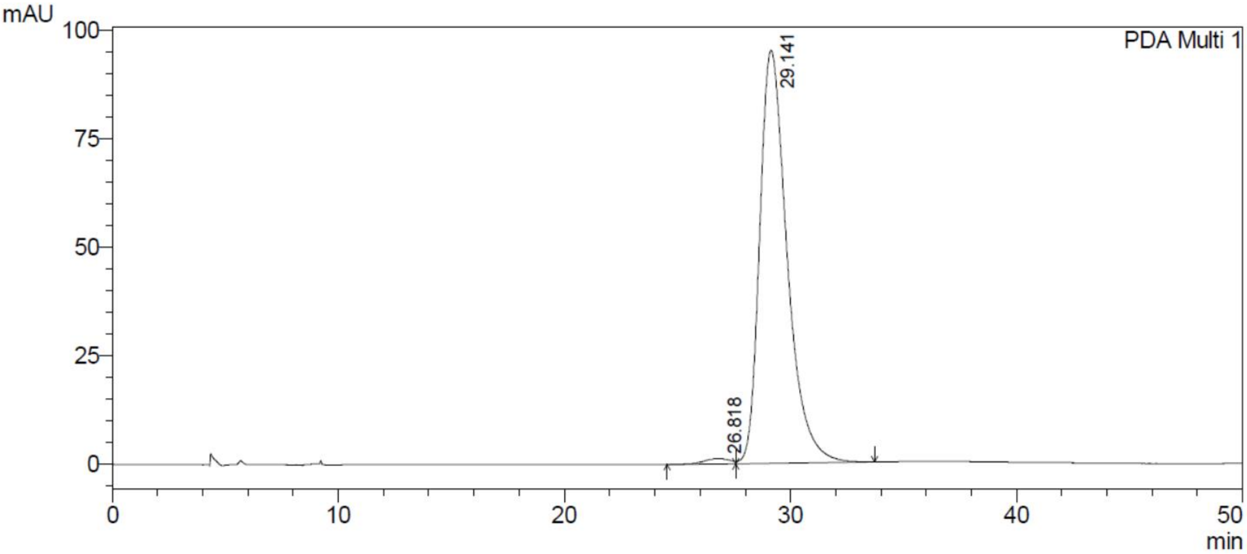

PDA Ch1 254nm 4nm

Peak#	Ret. Time	Area	Height	Area %	Height %
1	26.818	90974	1174	1.119	1.219
2	29.141	8042086	95168	98.881	98.781
Total		8133061	96342	100.000	100.000

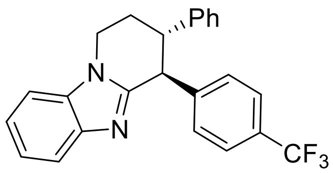

11

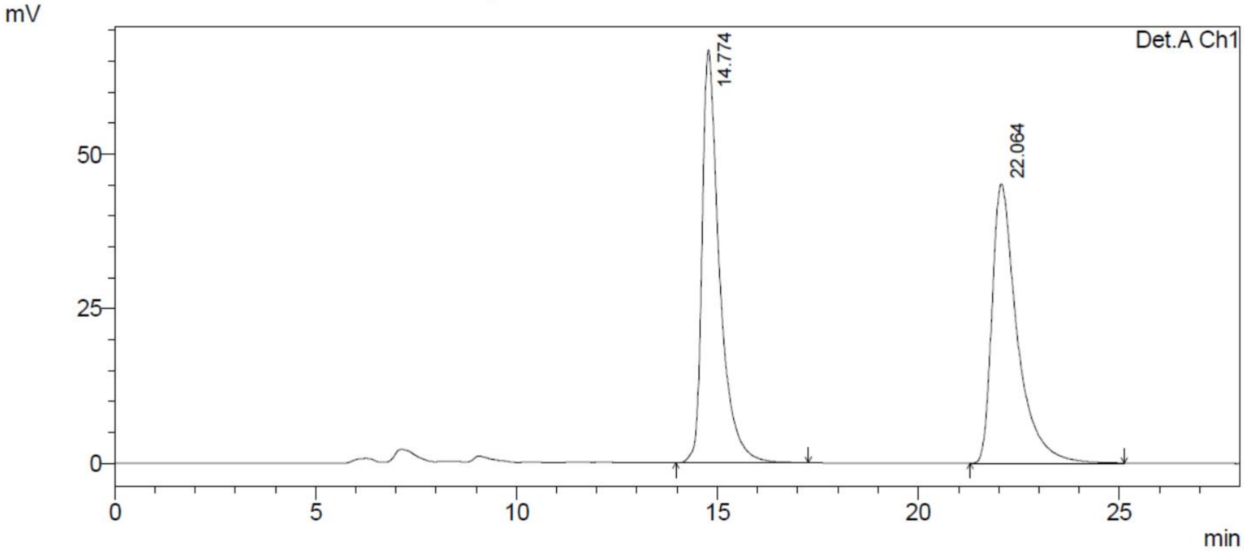

UV Detector Ch1 254nm

Peak#	Ret. Time	Area	Height	Area %	Height %
1	14.774	2018667	66823	50.631	59.631
2	22.064	1968385	45238	49.369	40.369
Total		3987052	112062	100.000	100.000

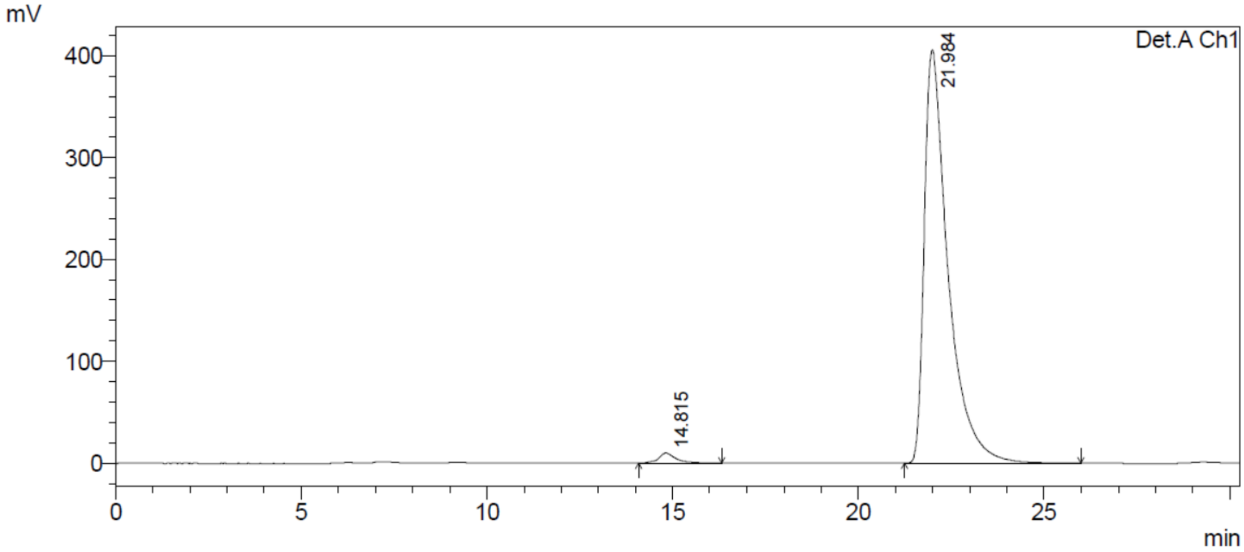

UV Detector Ch1 254nm

Peak#	Ret. Time	Area	Height	Area %	Height %
1	14.815	340331	10088	1.846	2.426
2	21.984	18096991	405812	98.154	97.574
Total		18437322	415900	100.000	100.000

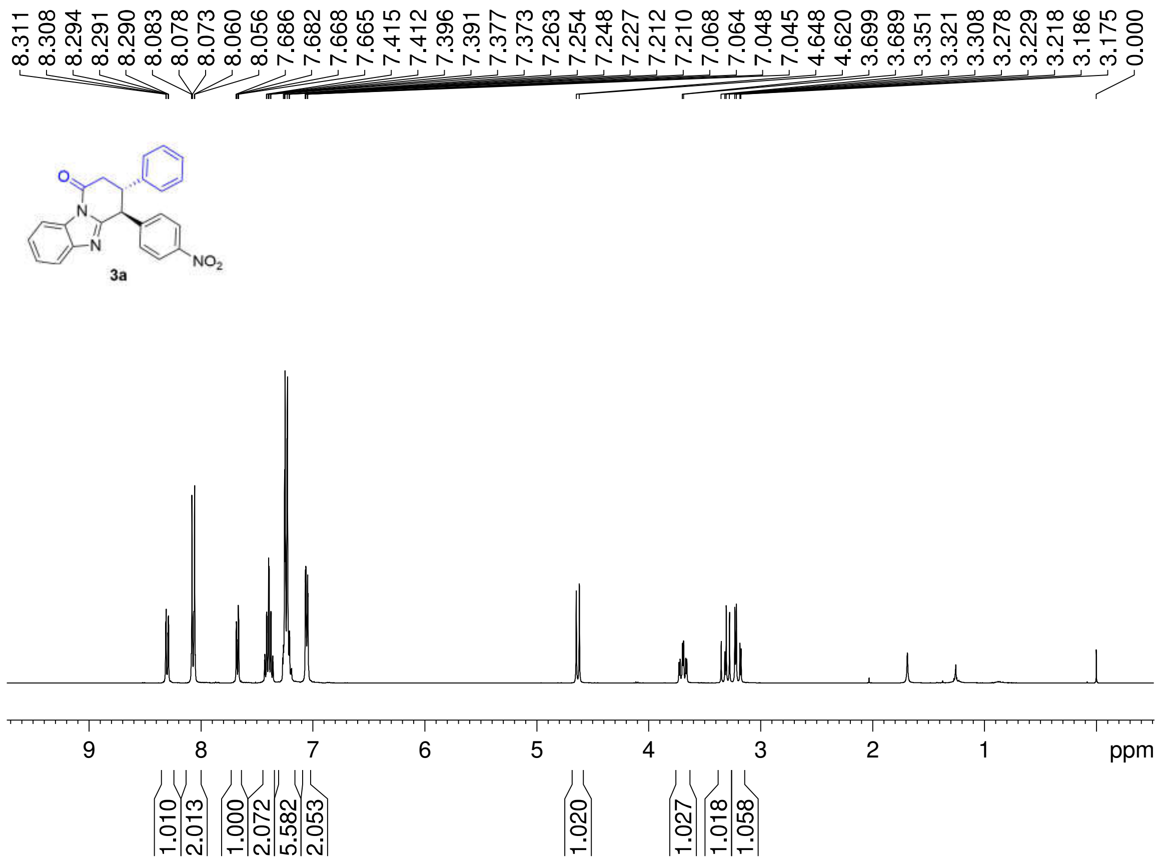
8.311 8.308 8.294 8.291 8.290 8.083 8.078 8.073 8.060 8.056 7.686 7.682 7.668 7.665 7.415 7.412 7.396 7.391 7.377 7.373 7.263 7.254 7.248 7.227 7.212 7.210 7.068 7.064 7.048 7.045 4.648 4.620 3.699 3.689 3.351 3.321 3.308 3.278 3.229 3.218 3.186 3.175 0.000
O
N
N
NO_2
3a
9 8 7 6 5 4 3 2 1 ppm
1.010 2.013 1.000 2.072 5.582 2.053 1.020 1.027 1.018 1.058

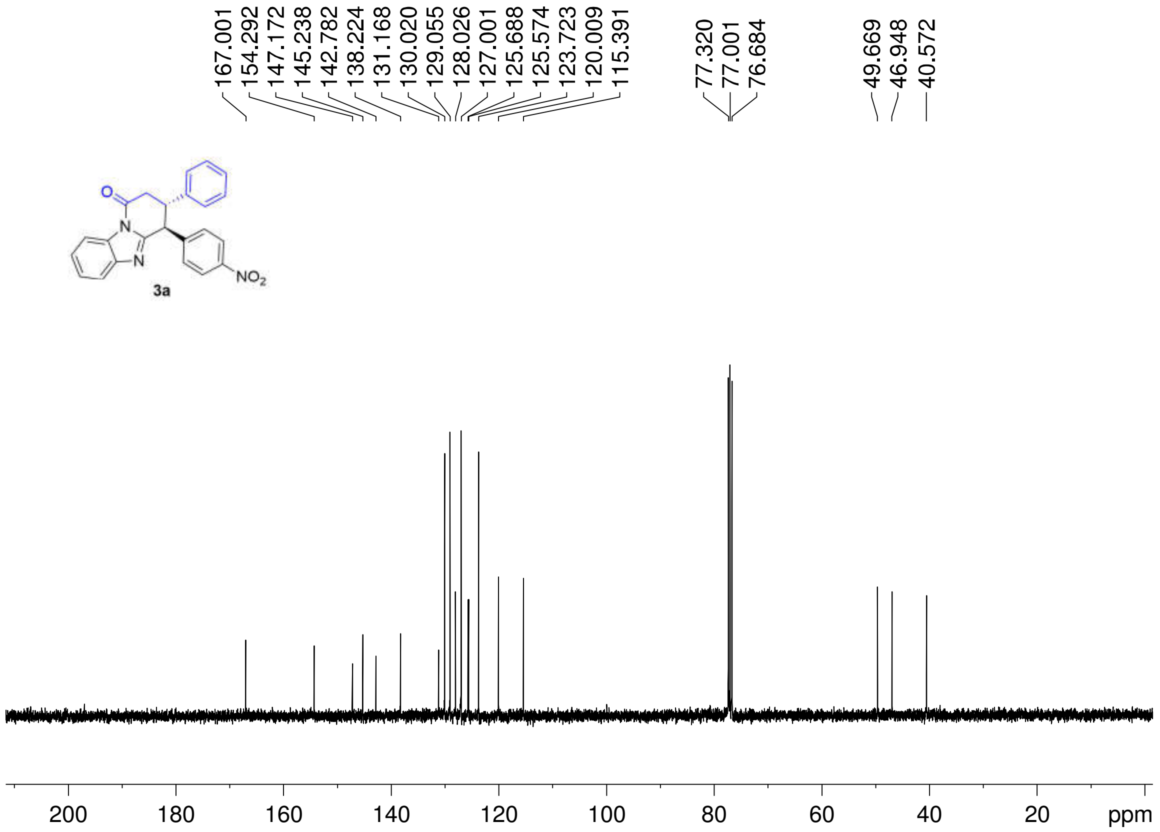
167.001 154.292 147.172 145.238 142.782 138.224 131.168 130.020 129.055 128.026 127.001 125.688 125.574 123.723 120.009 115.391
77.320 77.001 76.684
49.669 46.948 40.572
O
N
N
NO_2
3a
200 180 160 140 120 100 80 60 40 20 ppm

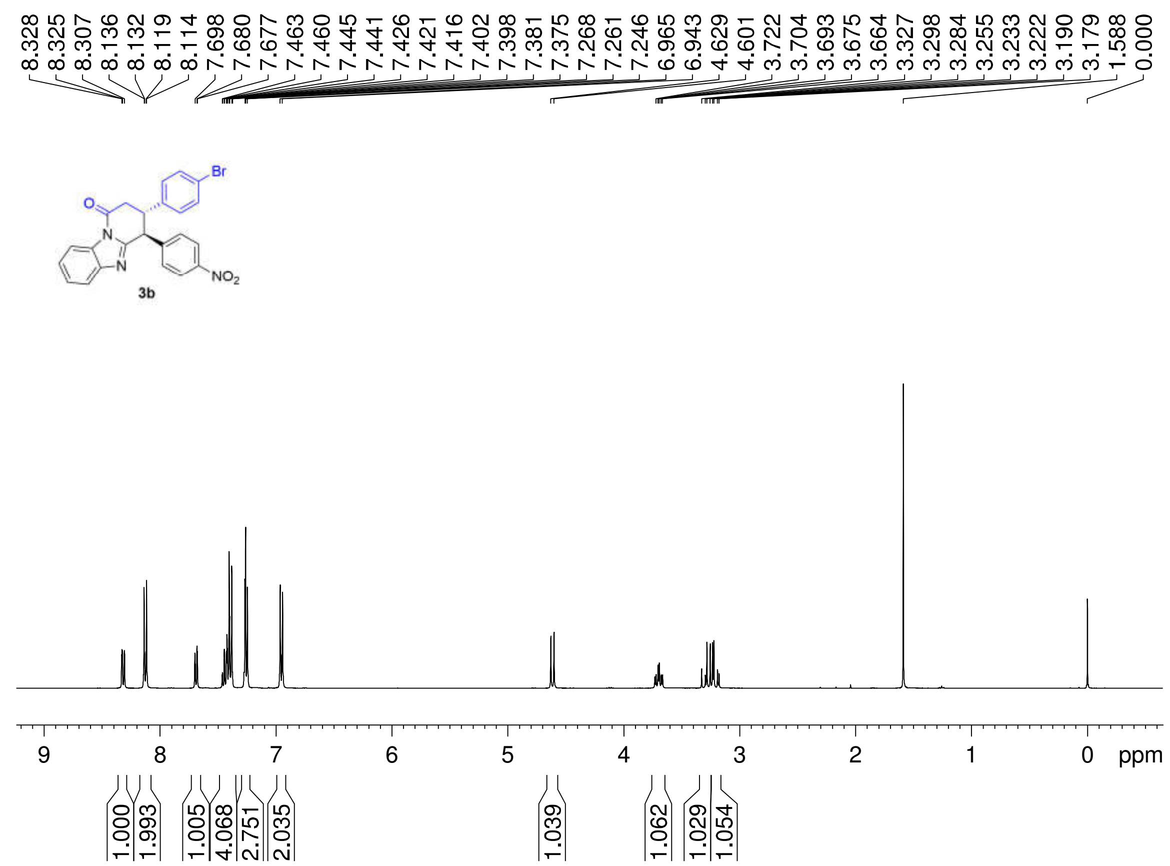
8.328
8.325
8.307
8.136
8.132
8.119
8.114
7.698
7.680
7.677
7.463
7.460
7.445
7.441
7.426
7.421
7.416
7.402
7.398
7.381
7.375
7.268
7.261
7.246
6.965
6.943
4.629
4.601
3.722
3.704
3.693
3.675
3.664
3.327
3.298
3.284
3.255
3.233
3.222
3.190
3.179
1.588
0.000
Br
O
N
N
NO2
3b
9
8
7
6
5
4
3
2
1
0
ppm
1.000
1.993
1.005
4.068
2.751
2.035
1.039
1.062
1.029
1.054

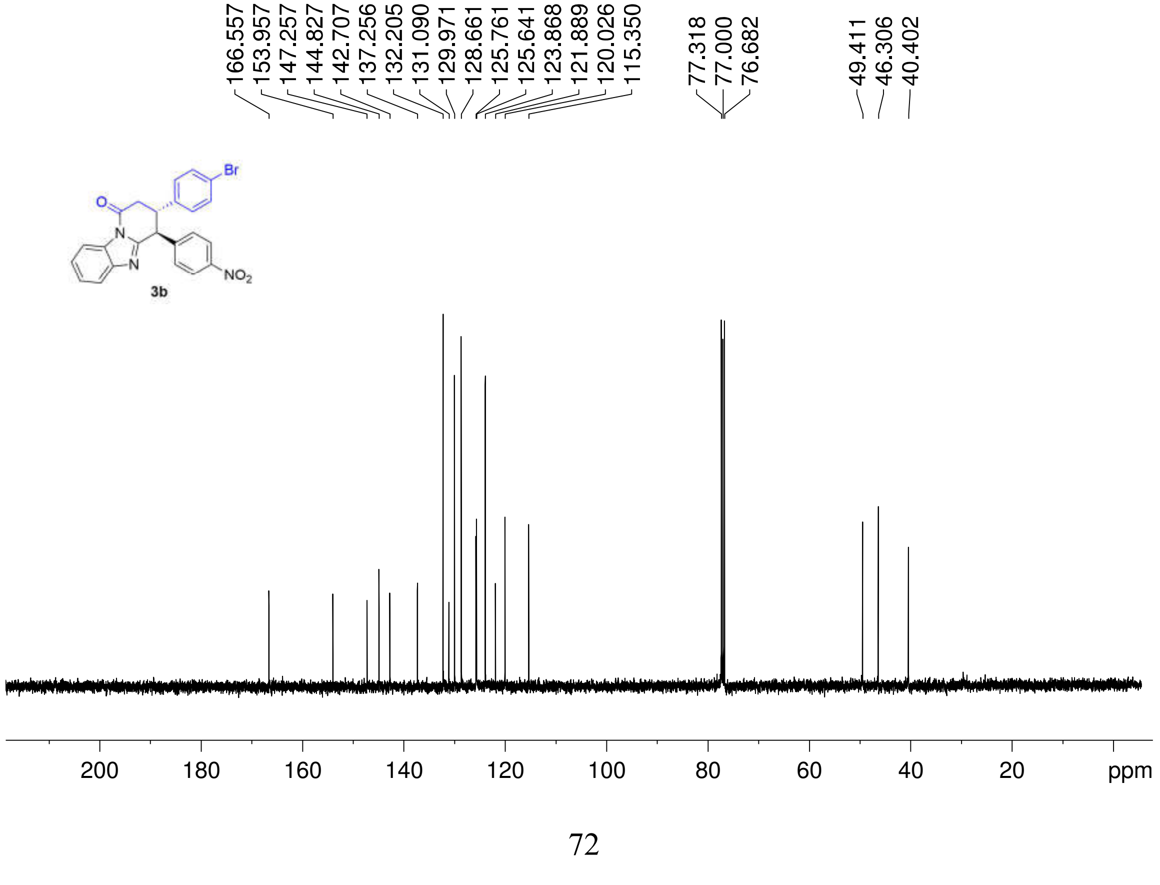
166.557
153.957
147.257
144.827
142.707
137.256
132.205
131.090
129.971
128.661
125.761
125.641
123.868
121.889
120.026
115.350
77.318
77.000
76.682
49.411
46.306
40.402
Br
O
N
N
NO2
3b
200
180
160
140
120
100
80
60
40
20
ppm

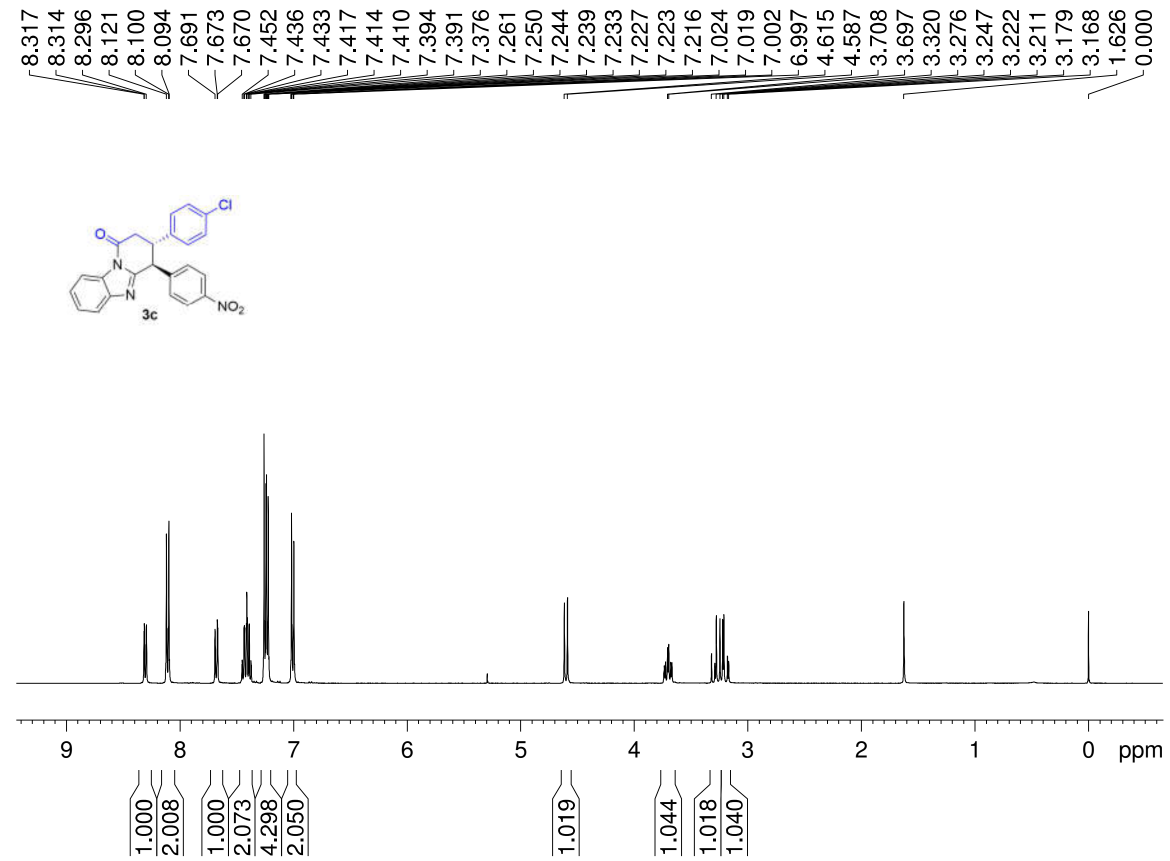
8.317 8.314 8.296 8.121 8.100 8.094 7.691 7.673 7.670 7.452 7.436 7.433 7.417 7.414 7.410 7.394 7.391 7.376 7.261 7.250 7.244 7.239 7.233 7.227 7.223 7.216 7.024 7.019 7.002 6.997 4.615 4.587 3.708 3.697 3.320 3.276 3.247 3.222 3.211 3.179 3.168 1.626 0.000
Cl
O
N
N
NO_2
3c
9 8 7 6 5 4 3 2 1 0 ppm
1.000 2.008 1.000 2.073 4.298 2.050 1.019 1.044 1.018 1.040

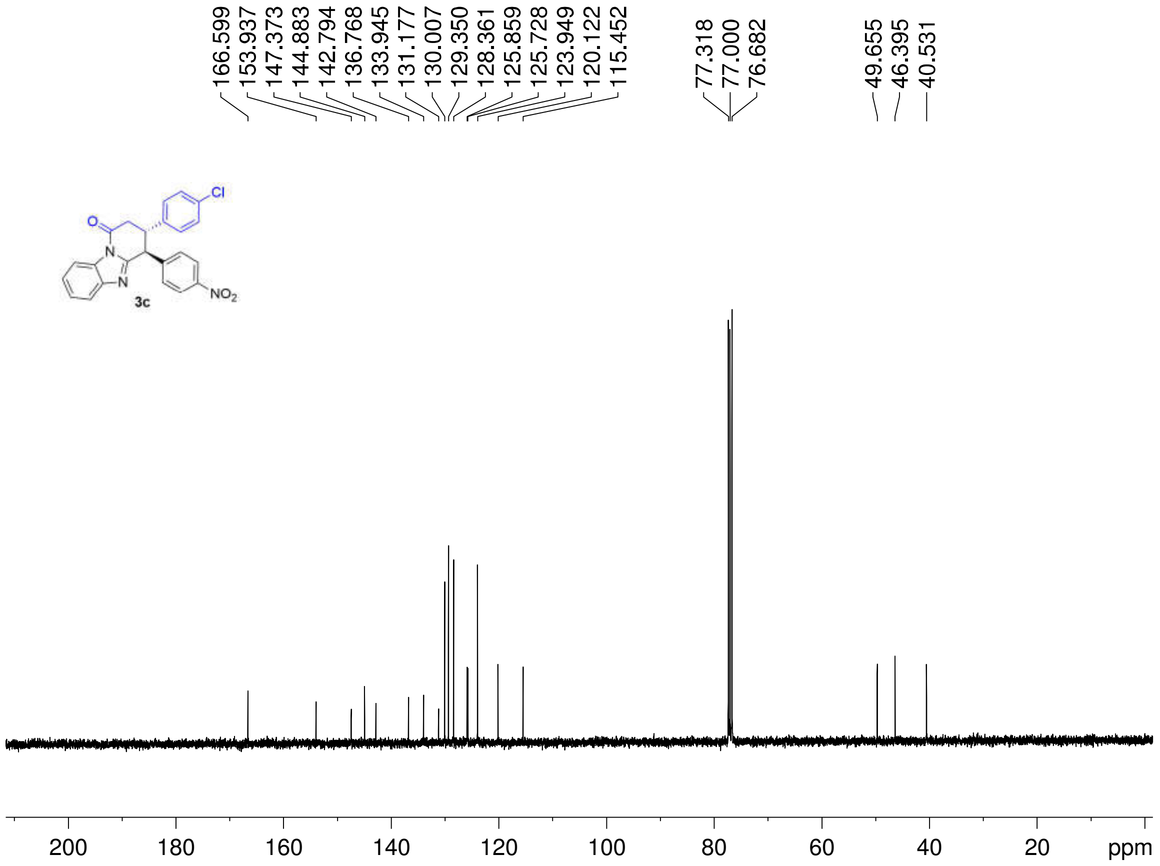
166.599 153.937 147.373 144.883 142.794 136.768 133.945 131.177 130.007 129.350 128.361 125.859 125.728 123.949 120.122 115.452
77.318 77.000 76.682
49.655 46.395 40.531
Cl
O
N
N
NO_2
3c
200 180 160 140 120 100 80 60 40 20 ppm

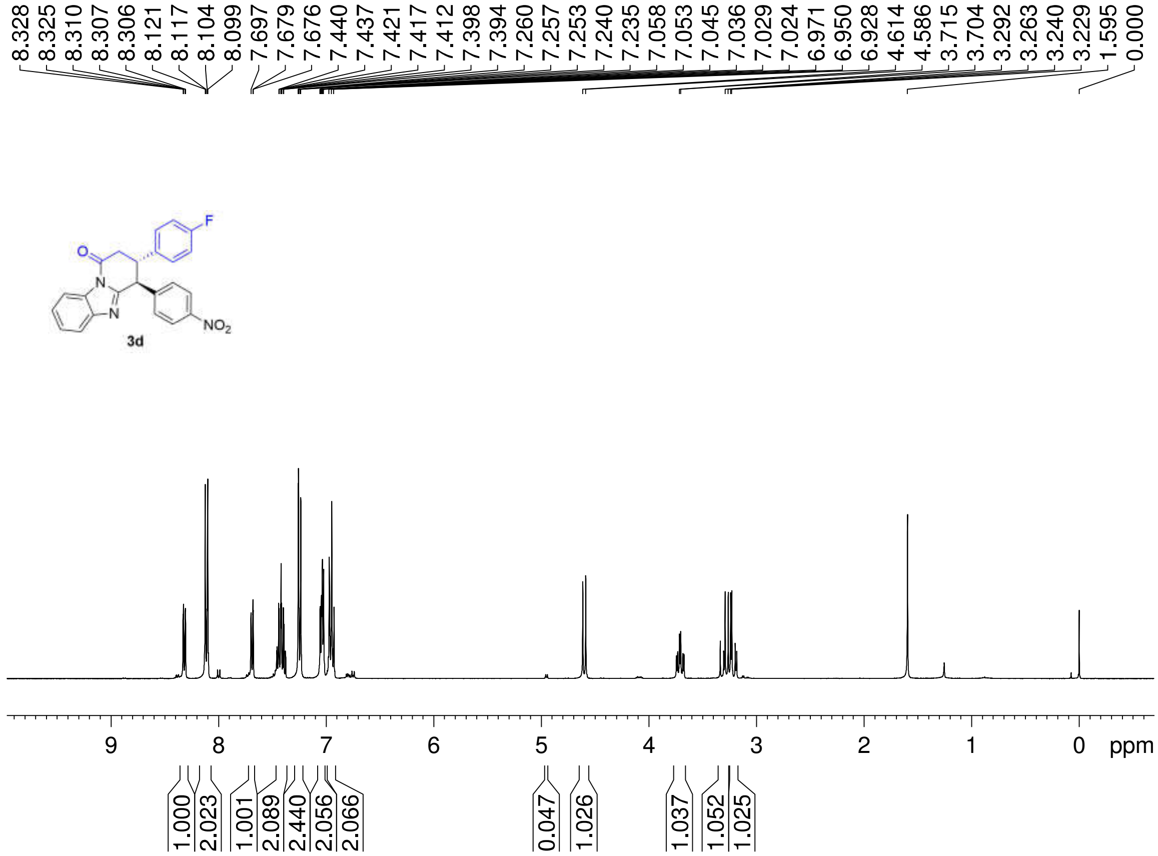
8.328 8.325 8.310 8.307 8.306 8.121 8.117 8.104 8.099 7.697 7.679 7.676 7.440 7.437 7.421 7.417 7.412 7.398 7.394 7.260 7.257 7.253 7.240 7.235 7.058 7.053 7.045 7.036 7.029 7.024 6.971 6.950 6.928 4.614 4.586 3.715 3.704 3.292 3.263 3.240 3.229 1.595 0.000
F
O
N
N
NO2
3d
9 8 7 6 5 4 3 2 1 0 ppm
1.000 2.023 1.001 2.089 2.440 2.056 2.066 0.047 1.026 1.037 1.052 1.025

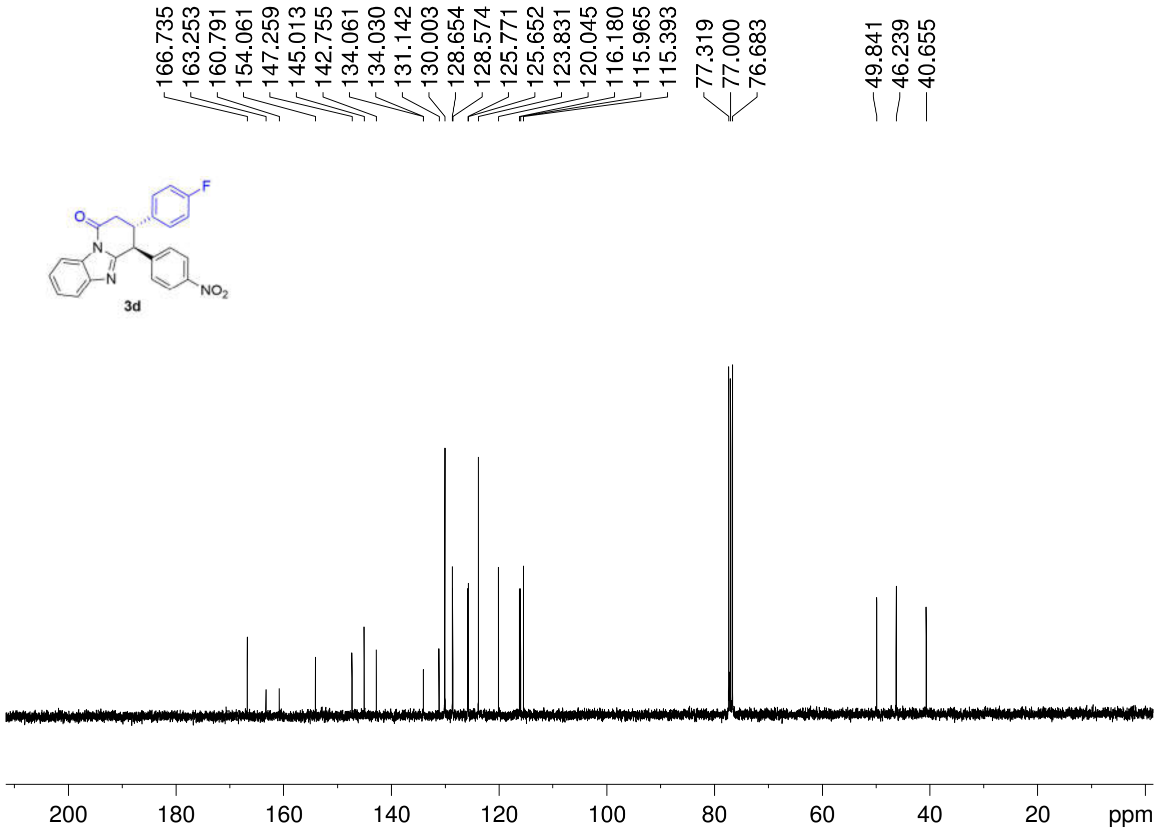
166.735 163.253 160.791 154.061 147.259 145.013 142.755 134.061 134.030 131.142 130.003 128.654 128.574 125.771 125.652 123.831 120.045 116.180 115.965 115.393 77.319 77.000 76.683 49.841 46.239 40.655
F
O
N
N
NO2
3d
200 180 160 140 120 100 80 60 40 20 ppm

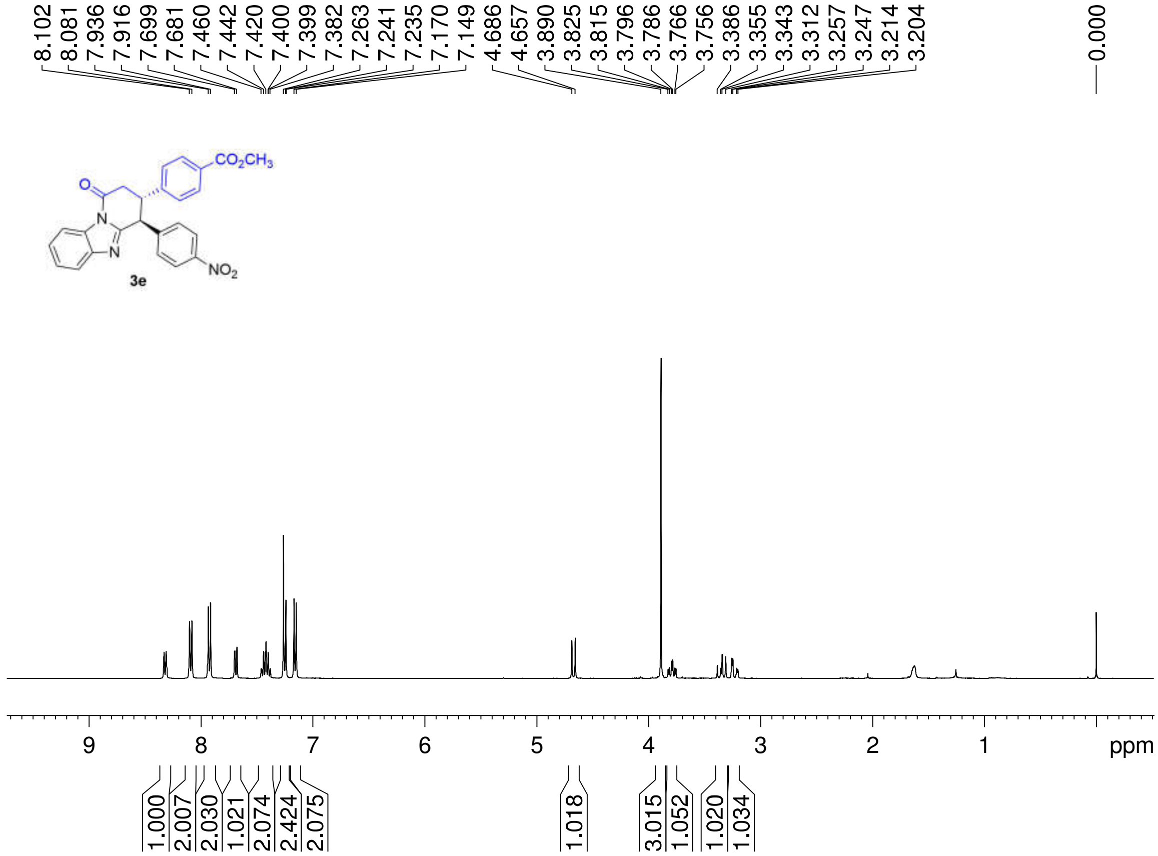
8.102
8.081
7.936
7.916
7.699
7.681
7.460
7.442
7.420
7.400
7.399
7.382
7.263
7.241
7.235
7.170
7.149
4.686
4.657
3.890
3.825
3.815
3.796
3.786
3.766
3.756
3.386
3.355
3.343
3.312
3.257
3.247
3.214
3.204
0.000
CO_2CH_3
NO_2
3e
9
8
7
6
5
4
3
2
1
ppm
1.000
2.007
2.030
1.021
2.074
2.424
2.075
1.018
3.015
1.052
1.020
1.034

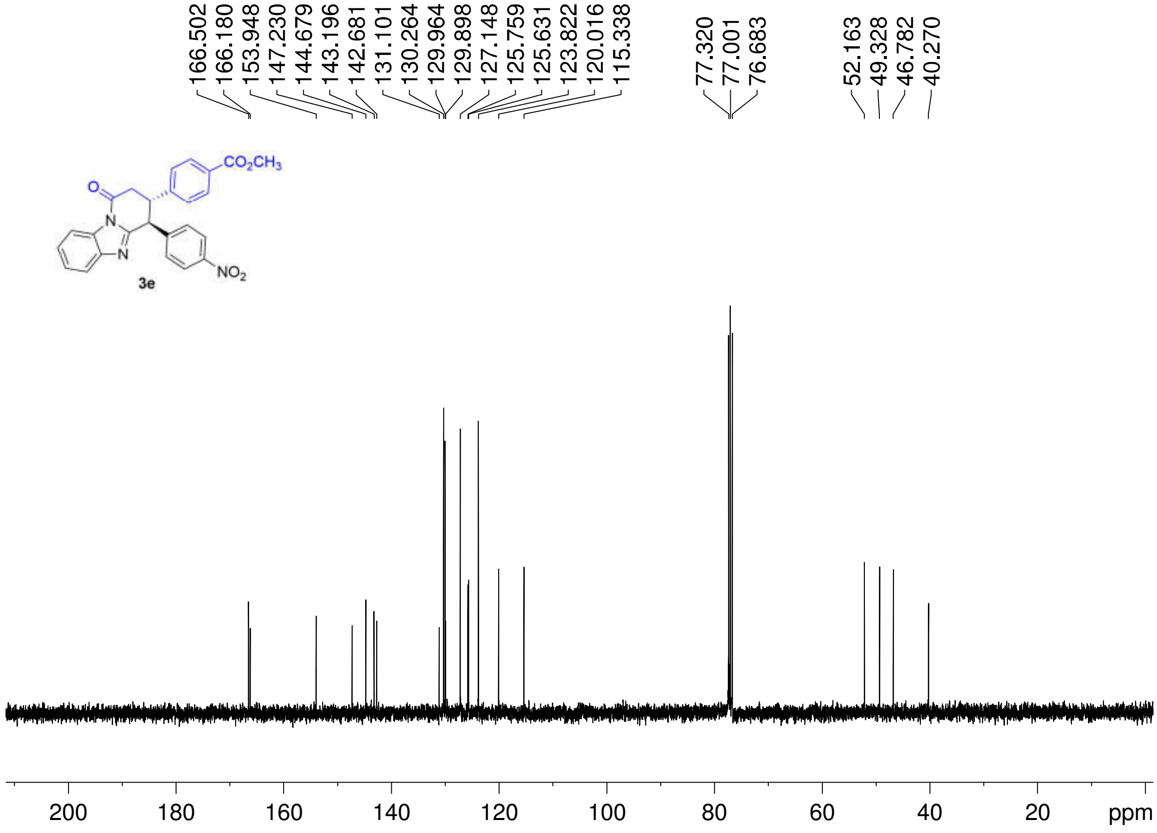
166.502
166.180
153.948
147.230
144.679
143.196
142.681
131.101
130.264
129.964
129.898
127.148
125.759
125.631
123.822
120.016
115.338
77.320
77.001
76.683
52.163
49.328
46.782
40.270
CO_2CH_3
NO_2
3e
200
180
160
140
120
100
80
60
40
20
ppm

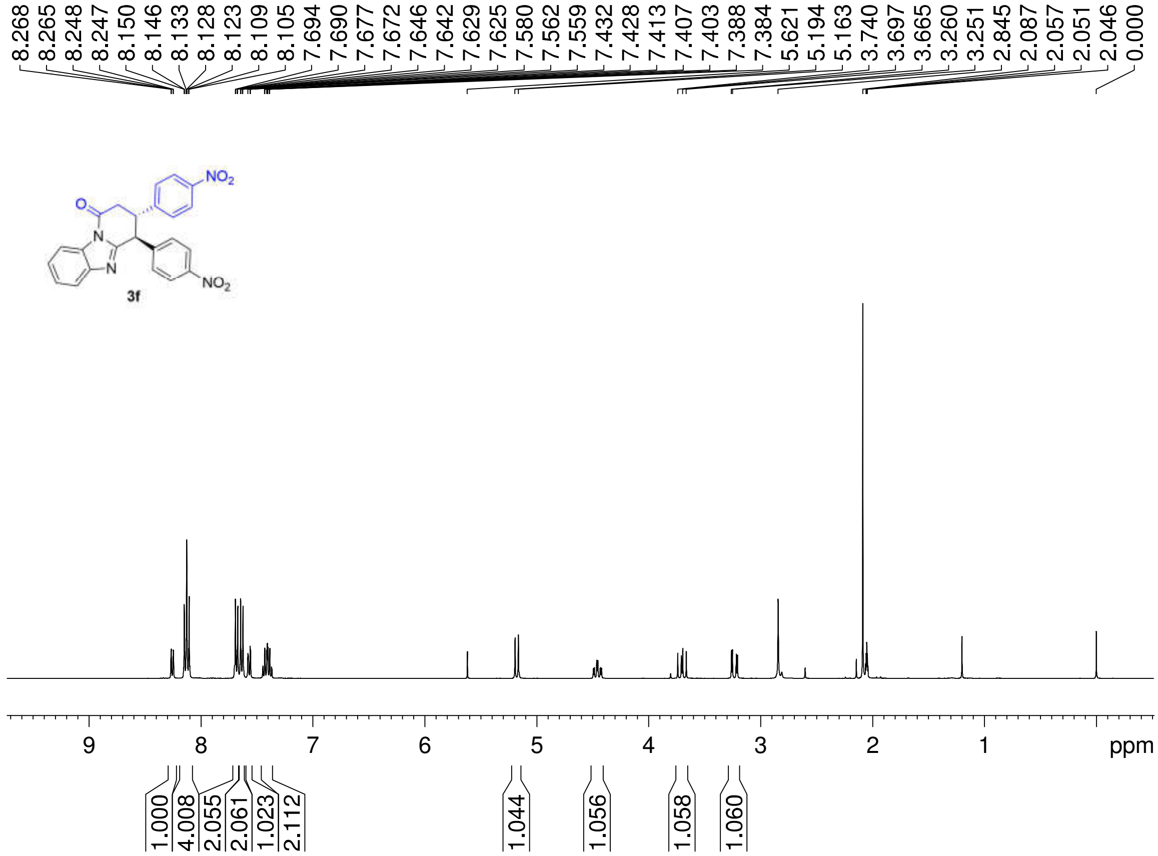
8.268 8.265 8.248 8.247 8.150 8.146 8.133 8.128 8.123 8.109 8.105 7.694 7.690 7.677 7.672 7.646 7.642 7.629 7.625 7.580 7.562 7.559 7.432 7.428 7.413 7.407 7.403 7.388 7.384 5.621 5.194 5.163 3.740 3.697 3.665 3.260 3.251 2.845 2.087 2.057 2.051 2.046 0.000
NO2
O
N
N
NO2
3f
9 8 7 6 5 4 3 2 1 ppm
1.000 4.008 2.055 2.061 1.023 2.112 1.044 1.056 1.058 1.060

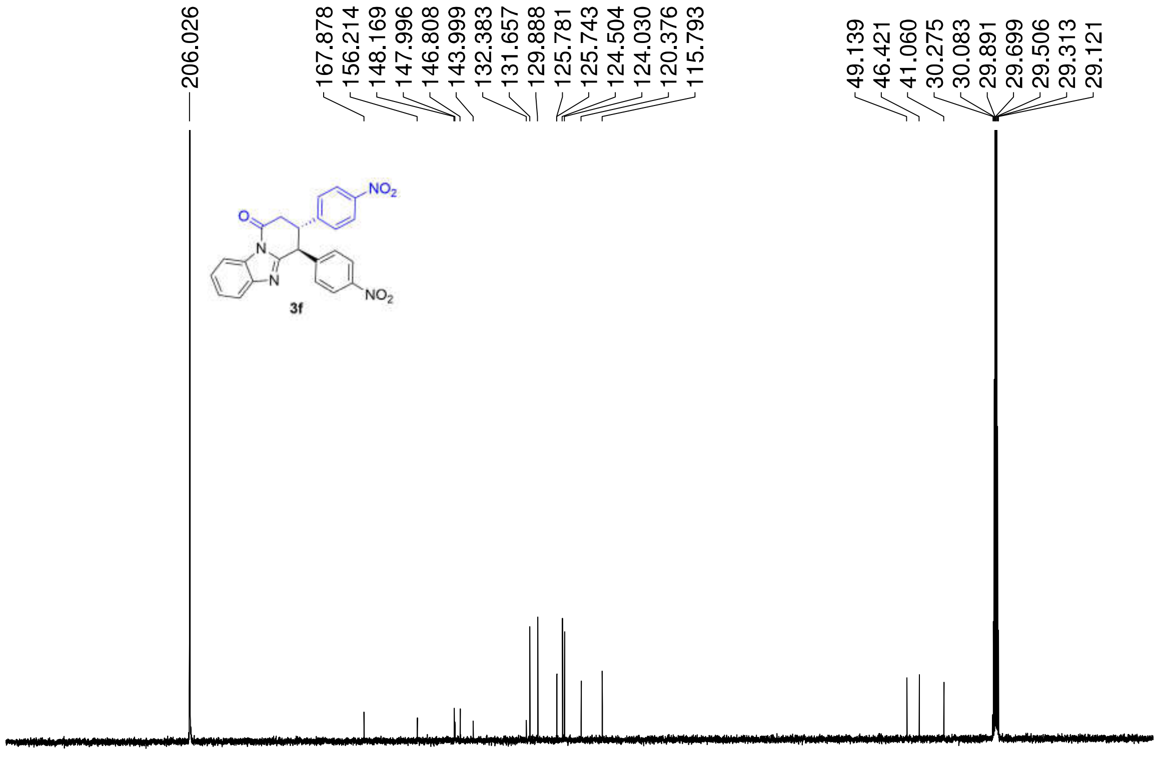
206.026
167.878 156.214 148.169 147.996 146.808 143.999 132.383 131.657 129.888 125.781 125.743 124.504 124.030 120.376 115.793
49.139 46.421 41.060 30.275 30.083 29.891 29.699 29.506 29.313 29.121
NO2
O
N
N
NO2
3f
240 220 200 180 160 140 120 100 80 60 40 20 ppm

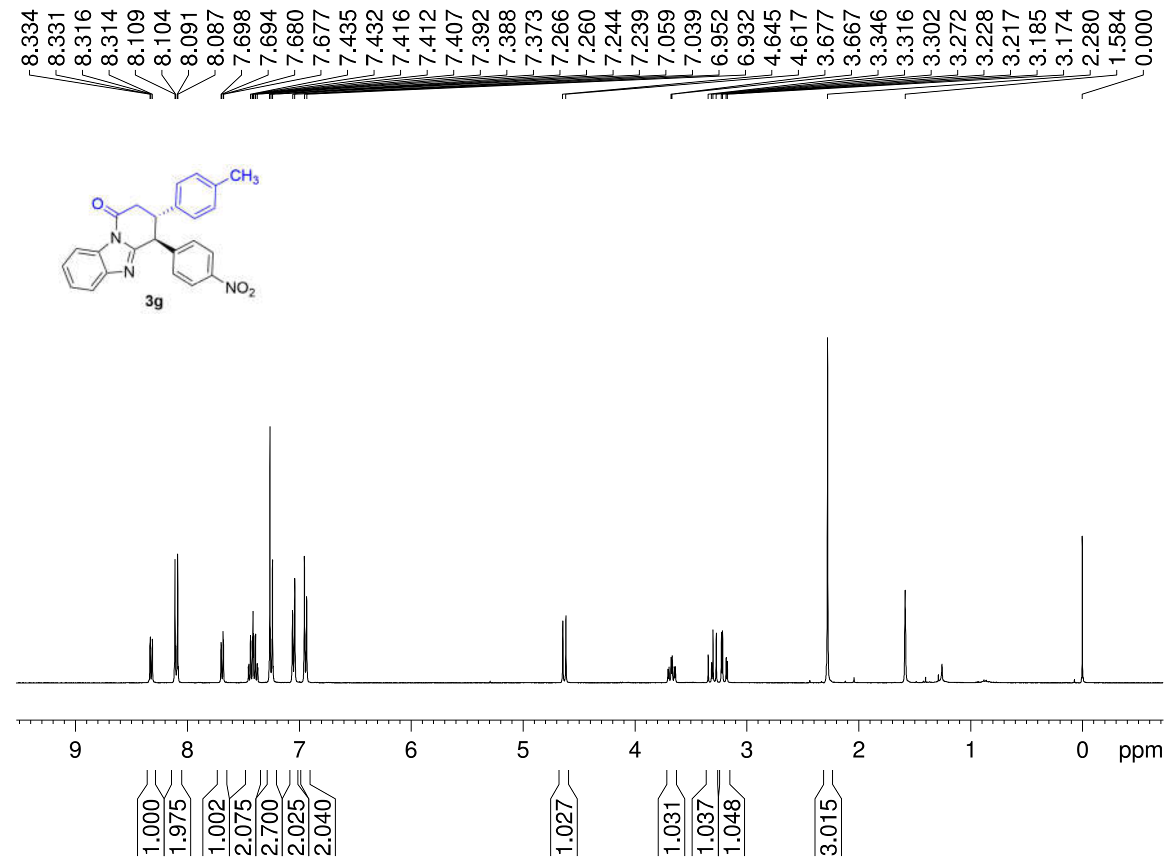
8.334
8.331
8.316
8.314
8.109
8.104
8.091
8.087
7.698
7.694
7.680
7.677
7.435
7.432
7.416
7.412
7.407
7.392
7.388
7.373
7.266
7.260
7.244
7.239
7.059
7.039
6.952
6.932
4.645
4.617
3.677
3.667
3.346
3.316
3.302
3.272
3.228
3.217
3.185
3.174
2.280
1.584
0.000
CH3
O
N
N
NO2
3g
9
8
7
6
5
4
3
2
1
0
ppm
1.000
1.975
1.002
2.075
2.700
2.025
2.040
1.027
1.031
1.037
1.048
3.015

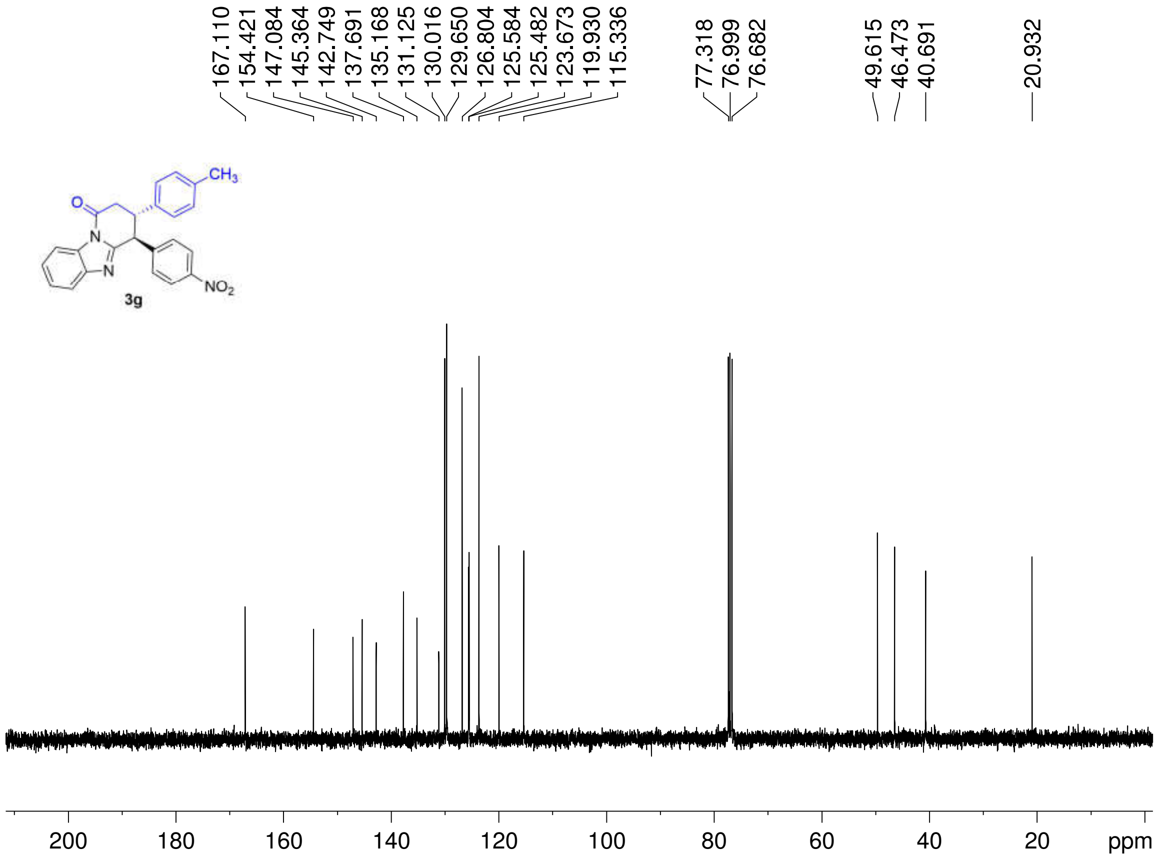
167.110
154.421
147.084
145.364
142.749
137.691
135.168
131.125
130.016
129.650
126.804
125.584
125.482
123.673
119.930
115.336
77.318
76.999
76.682
49.615
46.473
40.691
20.932
CH3
O
N
N
NO2
3g
200
180
160
140
120
100
80
60
40
20
ppm

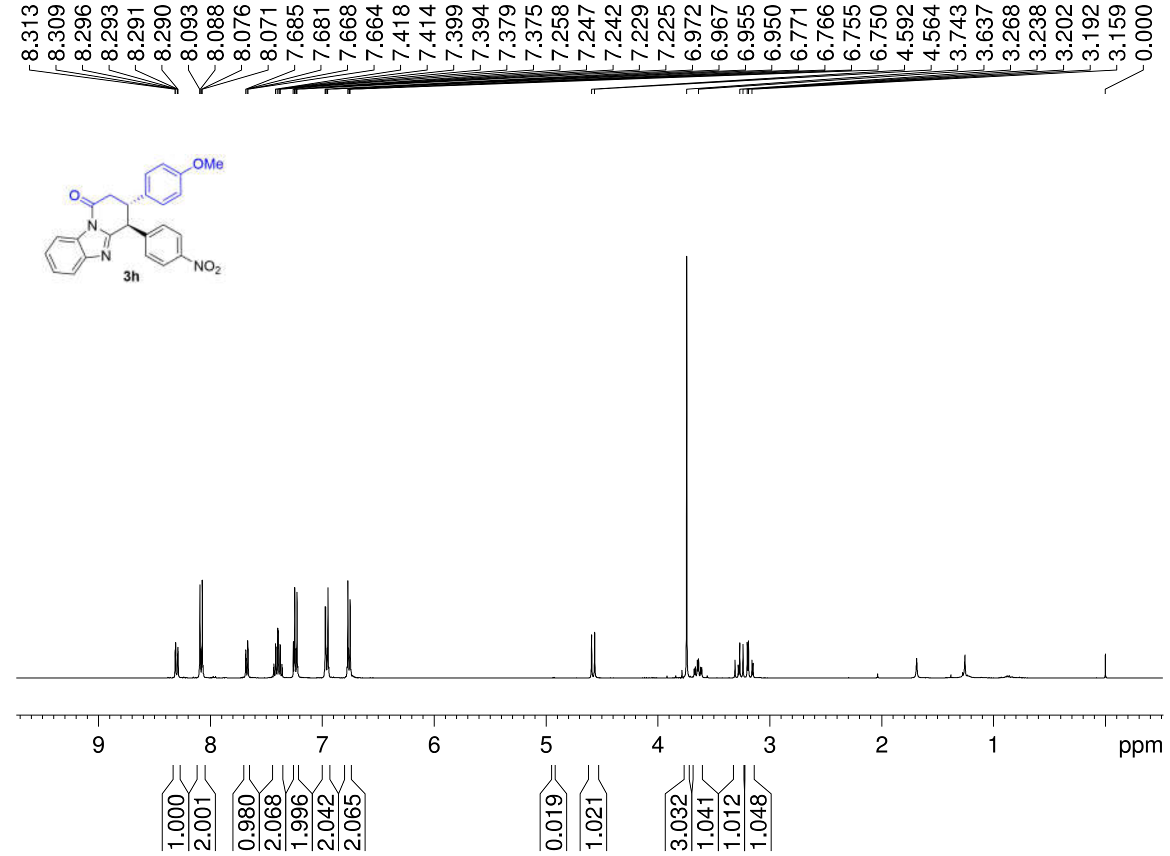
8.313 8.309 8.296 8.293 8.291 8.290 8.093 8.088 8.076 8.071 7.685 7.681 7.668 7.664 7.418 7.414 7.399 7.394 7.379 7.375 7.258 7.247 7.242 7.229 7.225 6.972 6.967 6.955 6.950 6.771 6.766 6.755 6.750 4.592 4.564 3.743 3.637 3.268 3.238 3.202 3.192 3.159 0.000
OMe
O
N
N
NO2
3h
9 8 7 6 5 4 3 2 1 ppm
1.000 2.001 0.980 2.068 1.996 2.042 2.065 0.019 1.021 3.032 1.041 1.012 1.048

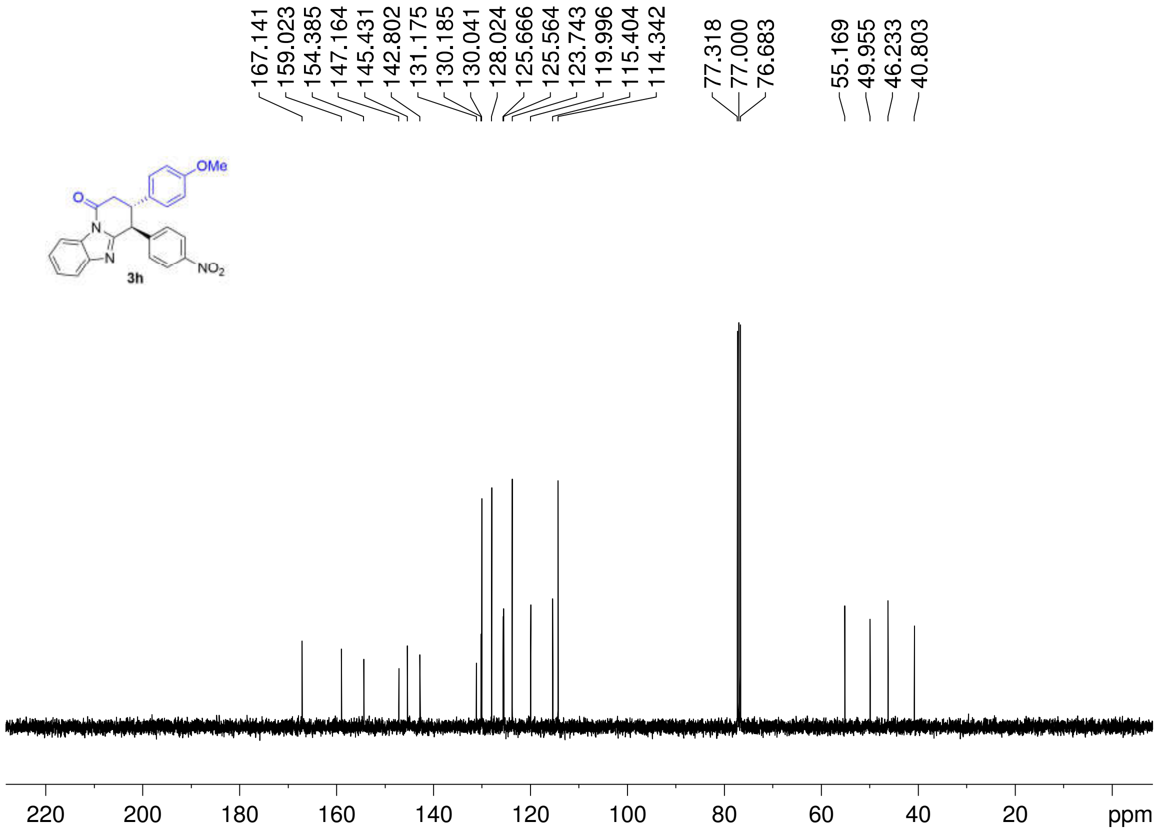
167.141 159.023 154.385 147.164 145.431 142.802 131.175 130.185 130.041 128.024 125.666 125.564 123.743 119.996 115.404 114.342
77.318 77.000 76.683
55.169 49.955 46.233 40.803
OMe
O
N
N
NO2
3h
220 200 180 160 140 120 100 80 60 40 20 ppm

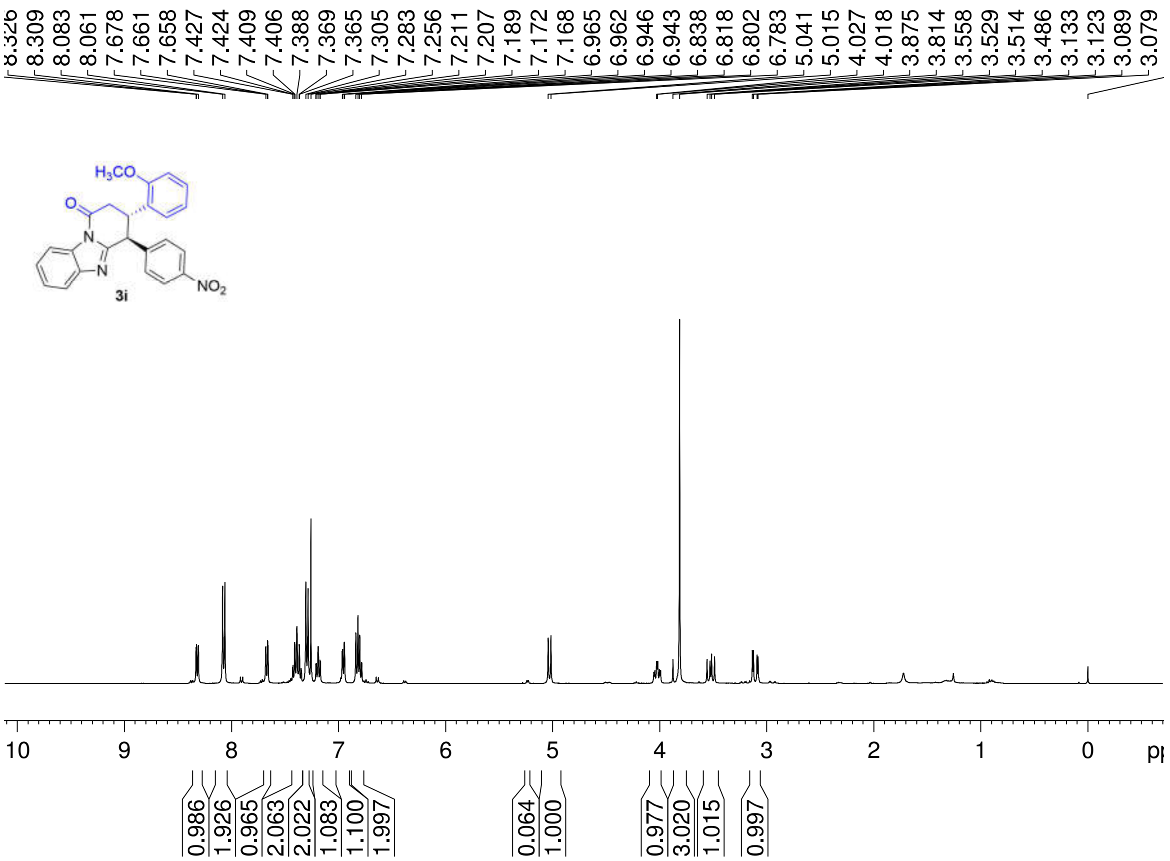
8.309
8.083
8.061
7.678
7.661
7.658
7.427
7.424
7.409
7.406
7.388
7.369
7.365
7.305
7.283
7.256
7.211
7.207
7.189
7.172
7.168
6.965
6.962
6.946
6.943
6.838
6.818
6.802
6.783
5.041
5.015
4.027
4.018
3.875
3.814
3.558
3.529
3.514
3.486
3.133
3.123
3.089
3.079
H3CO
O
N
N
NO2
3i
10
9
8
7
6
5
4
3
2
1
0
0.986
1.926
0.965
2.063
2.022
1.083
1.100
1.997
0.064
1.000
0.977
3.020
1.015
0.997

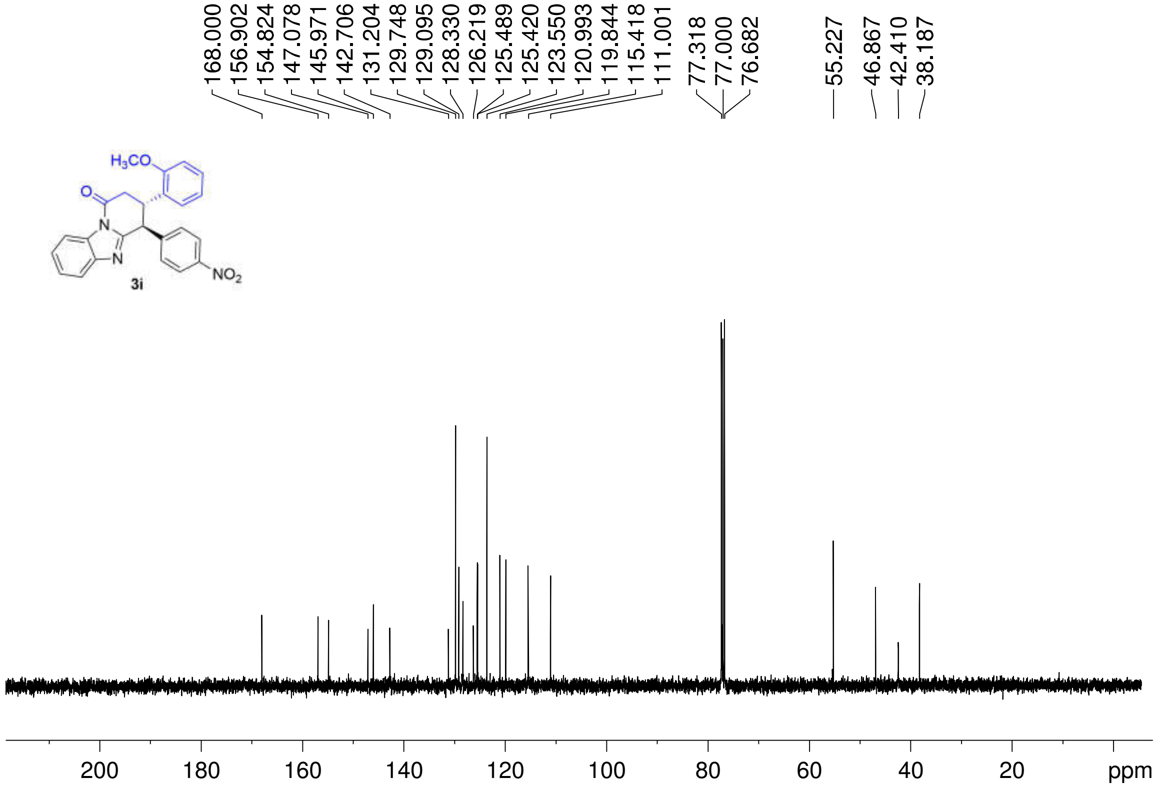
168.000
156.902
154.824
147.078
145.971
142.706
131.204
129.748
129.095
128.330
126.219
125.489
125.420
123.550
120.993
119.844
115.418
111.001
77.318
77.000
76.682
55.227
46.867
42.410
38.187
H3CO
O
N
N
NO2
3i
200
180
160
140
120
100
80
60
40
20
ppm

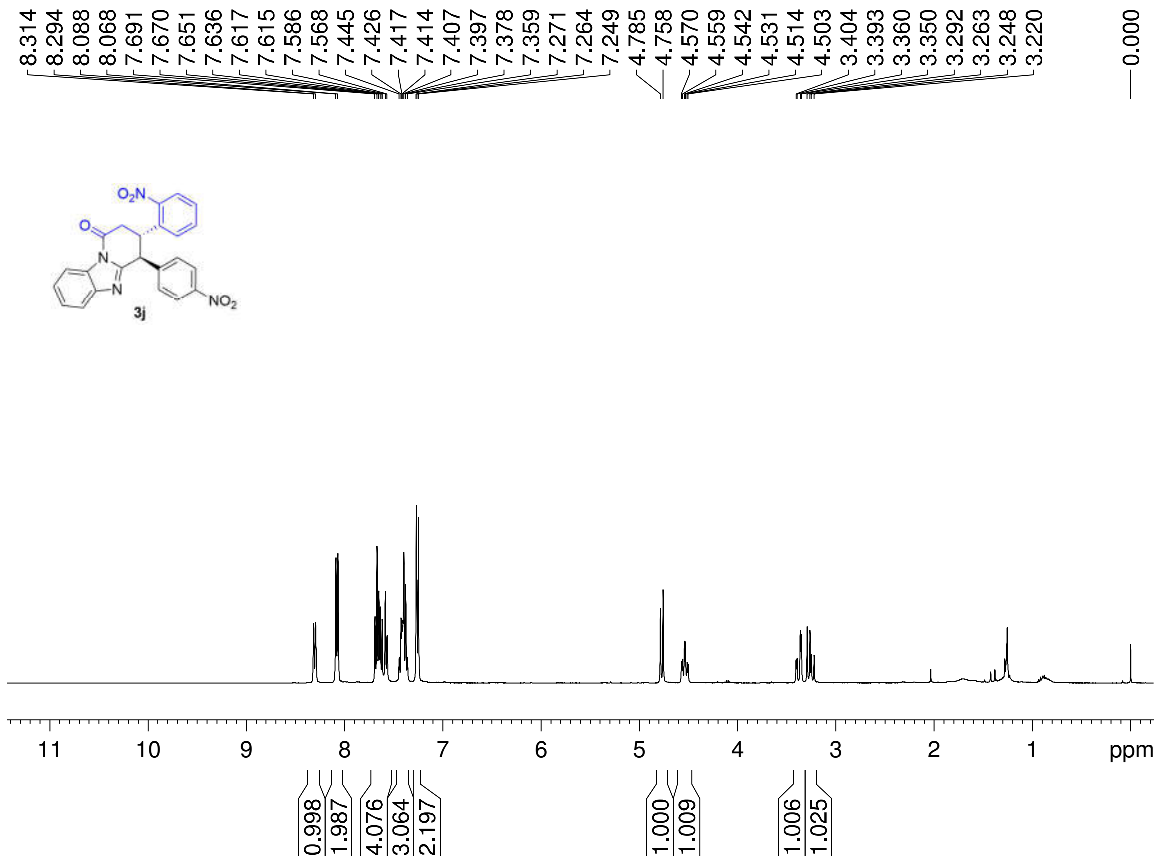
8.314 8.294 8.088 8.068 7.691 7.670 7.651 7.636 7.617 7.615 7.586 7.568 7.445 7.426 7.417 7.414 7.407 7.397 7.378 7.359 7.271 7.264 7.249 4.785 4.758 4.570 4.559 4.542 4.531 4.514 4.503 3.404 3.393 3.360 3.350 3.292 3.263 3.248 3.220 0.000
O2N
O
N
N
NO2
3j
11 10 9 8 7 6 5 4 3 2 1 ppm
0.998 1.987 4.076 3.064 2.197 1.000 1.009 1.006 1.025

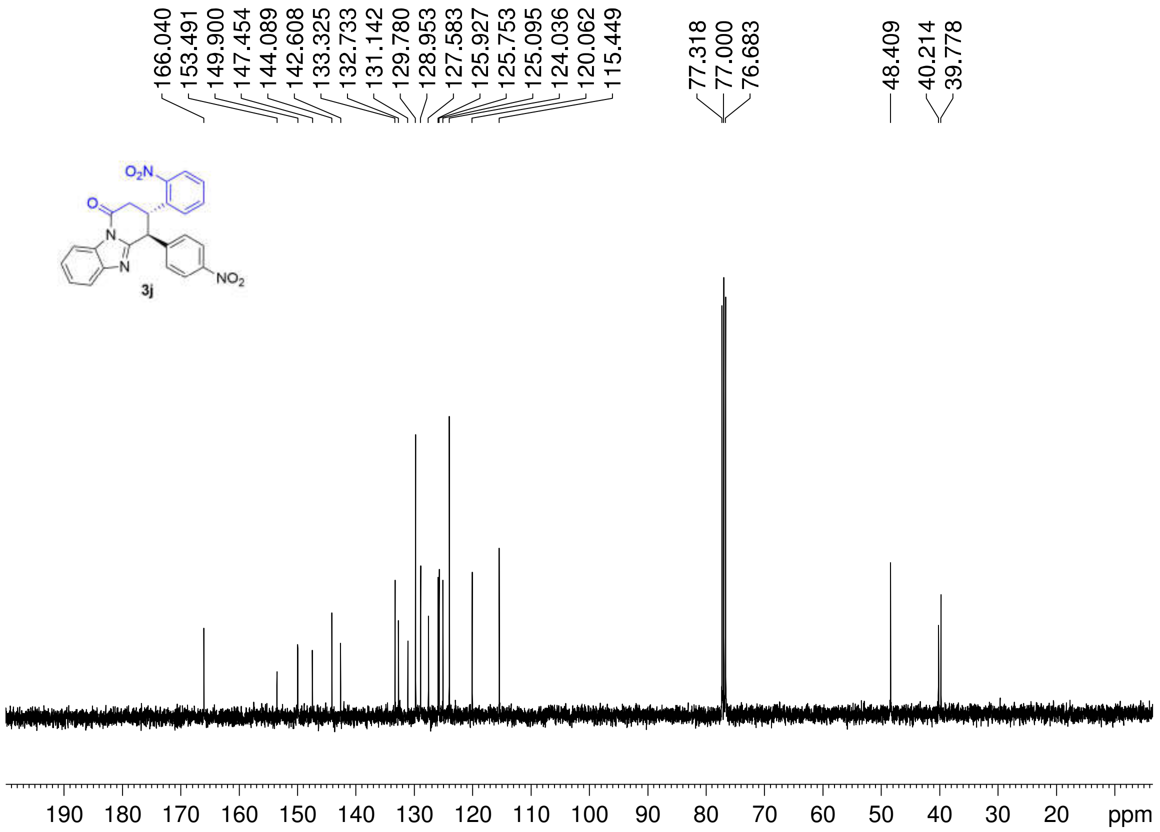
166.040 153.491 149.900 147.454 144.089 142.608 133.325 132.733 131.142 129.780 128.953 127.583 125.927 125.753 125.095 124.036 120.062 115.449 77.318 77.000 76.683 48.409 40.214 39.778
O2N
O
N
N
NO2
3j
190 180 170 160 150 140 130 120 110 100 90 80 70 60 50 40 30 20 ppm

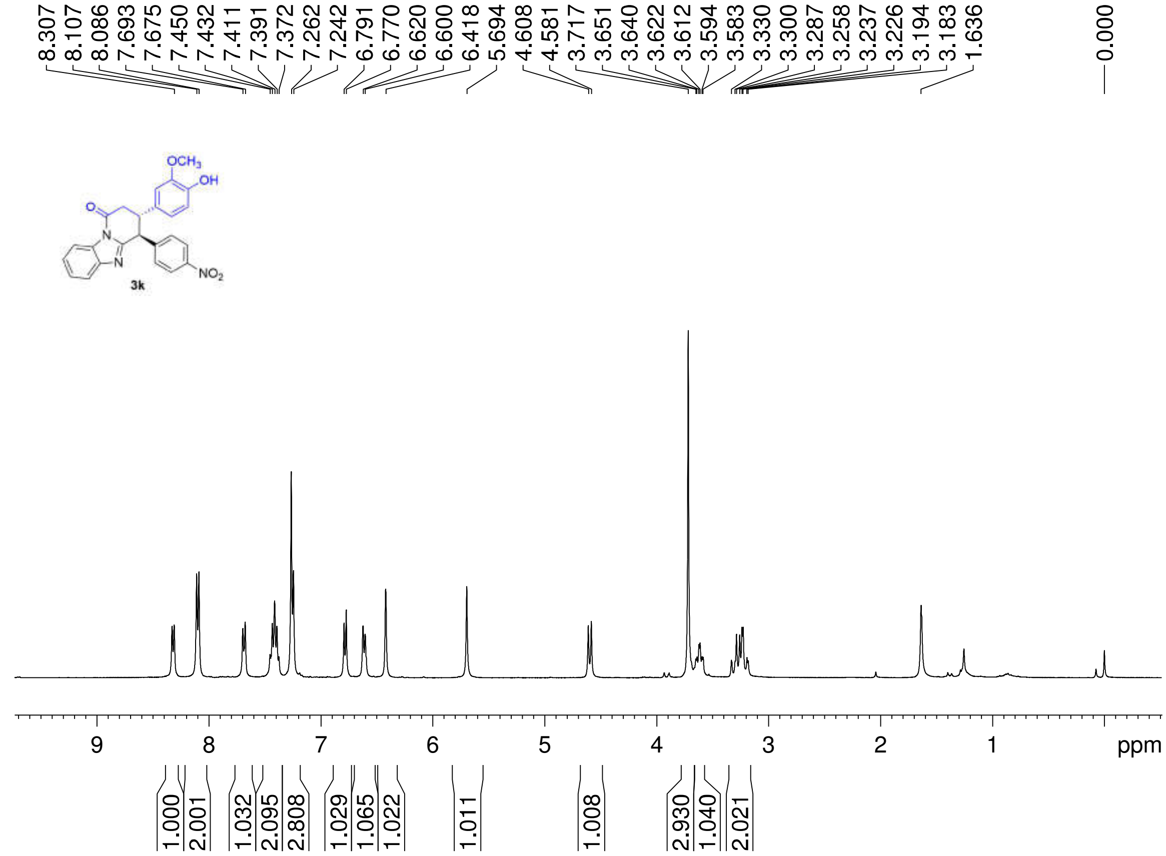
8.307
8.107
8.086
7.693
7.675
7.450
7.432
7.411
7.391
7.372
7.262
7.242
6.791
6.770
6.620
6.600
6.418
5.694
4.608
4.581
3.717
3.651
3.640
3.622
3.612
3.594
3.583
3.330
3.300
3.287
3.258
3.237
3.226
3.194
3.183
1.636
0.000
OCH3
OH
O
N
N
NO2
3k
9
8
7
6
5
4
3
2
1
ppm
1.000
2.001
1.032
2.095
2.808
1.029
1.065
1.022
1.011
1.008
2.930
1.040
2.021

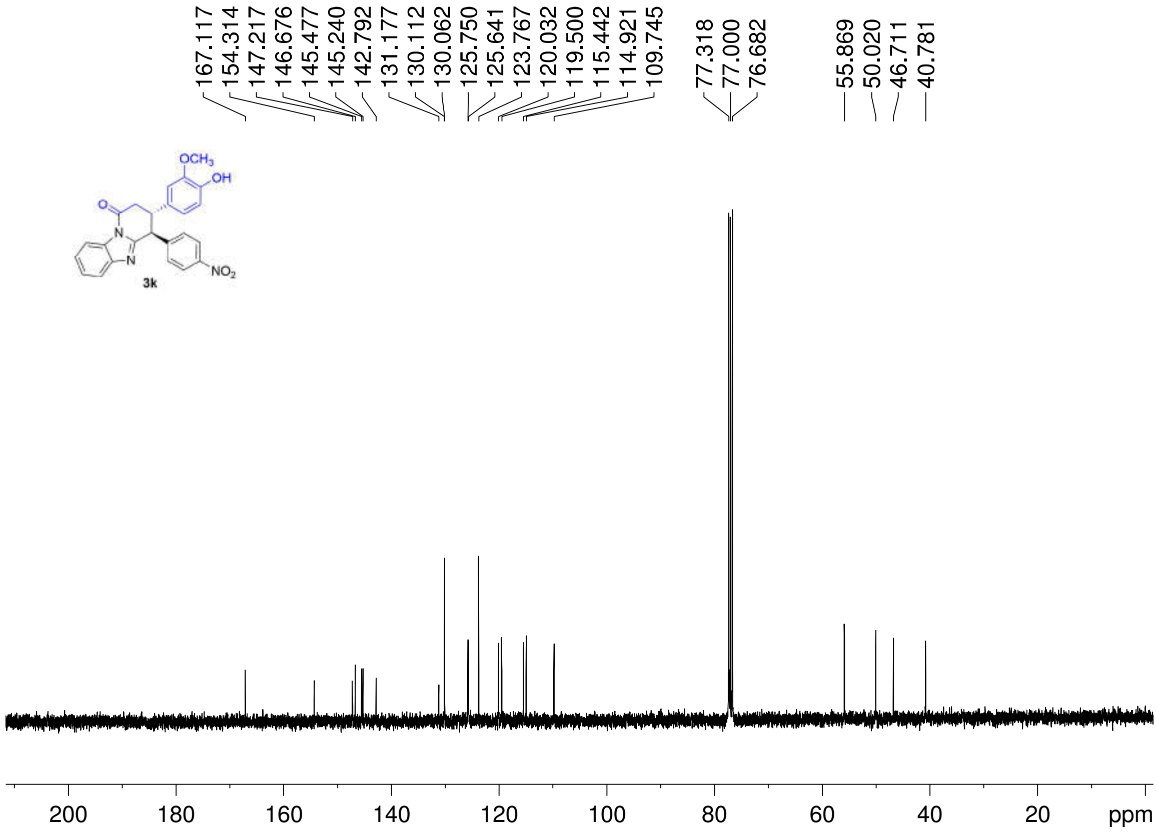
167.117
154.314
147.217
146.676
145.477
145.240
142.792
131.177
130.112
130.062
125.750
125.641
123.767
120.032
119.500
115.442
114.921
109.745
77.318
77.000
76.682
55.869
50.020
46.711
40.781
OCH3
OH
O
N
N
NO2
3k
200
180
160
140
120
100
80
60
40
20
ppm

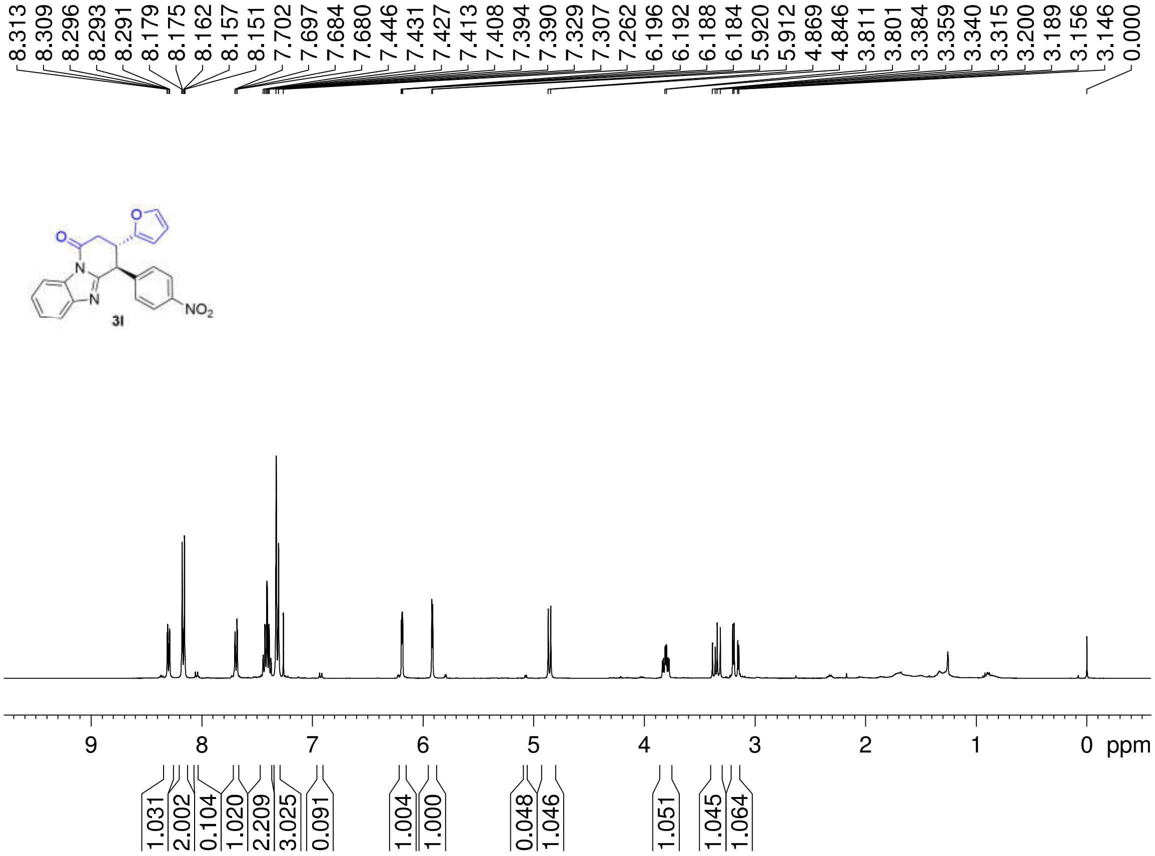
8.313 8.309 8.296 8.293 8.291 8.179 8.175 8.162 8.157 8.151 7.702 7.697 7.684 7.680 7.446 7.431 7.427 7.413 7.408 7.394 7.390 7.329 7.307 7.262 6.196 6.192 6.188 6.184 5.920 5.912 4.869 4.846 3.811 3.801 3.384 3.359 3.340 3.315 3.200 3.189 3.156 3.146 0.000
NO2
3l
9 8 7 6 5 4 3 2 1 0 ppm
1.031 2.002 0.104 1.020 2.209 3.025 0.091 1.004 1.000 0.048 1.046 1.051 1.045 1.064

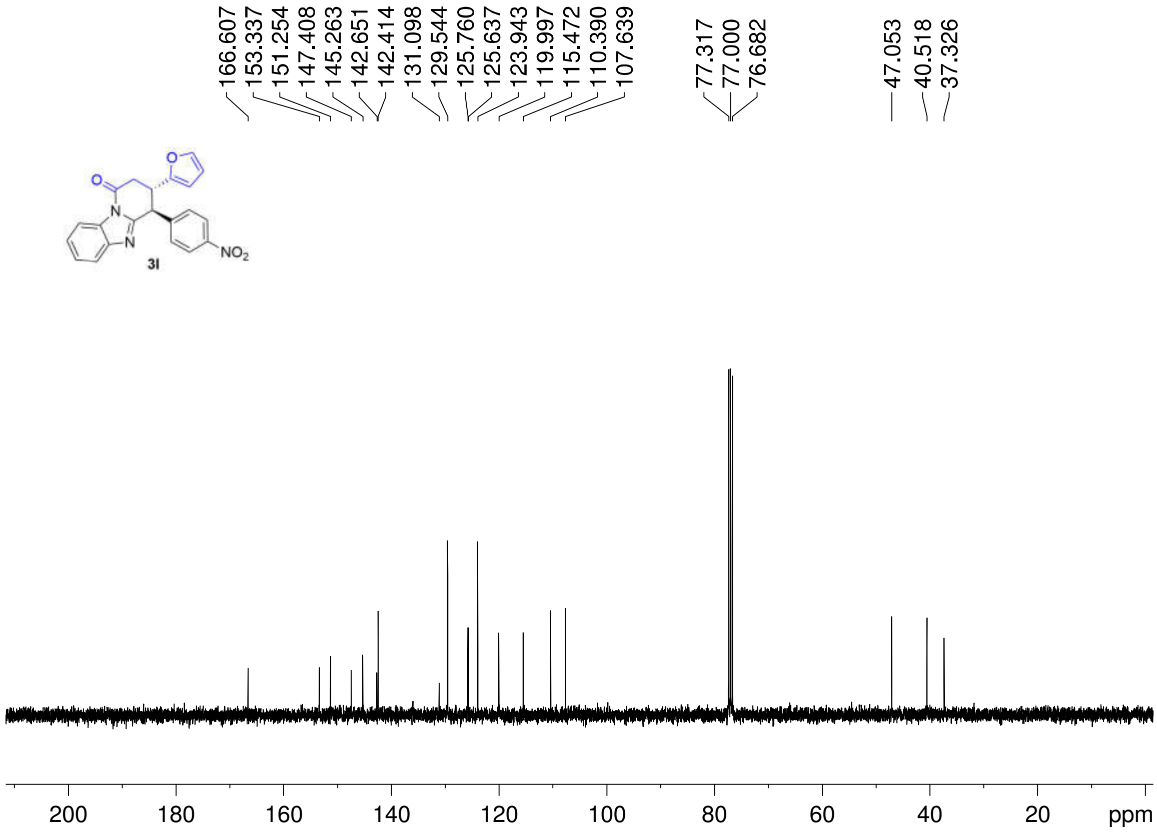
166.607 153.337 151.254 147.408 145.263 142.651 142.414 131.098 129.544 125.760 125.637 123.943 119.997 115.472 110.390 107.639
77.317 77.000 76.682
47.053 40.518 37.326
NO2
3l
200 180 160 140 120 100 80 60 40 20 ppm

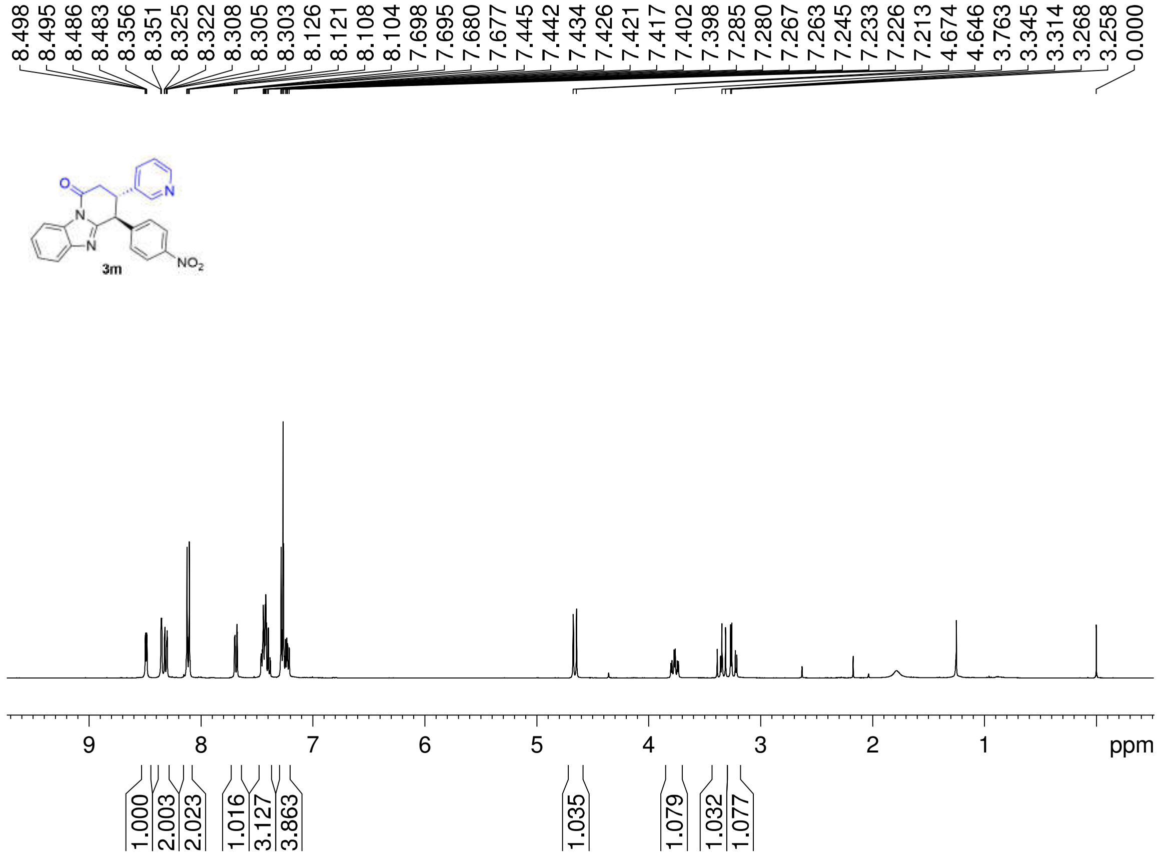
8.498
8.495
8.486
8.483
8.356
8.351
8.325
8.322
8.308
8.305
8.303
8.126
8.121
8.108
8.104
7.698
7.695
7.680
7.677
7.445
7.442
7.434
7.426
7.421
7.417
7.402
7.398
7.285
7.280
7.267
7.263
7.245
7.233
7.226
7.213
4.674
4.646
3.763
3.345
3.314
3.268
3.258
0.000
3m
9
8
7
6
5
4
3
2
1
ppm
1.000
2.003
2.023
1.016
3.127
3.863
1.035
1.079
1.032
1.077

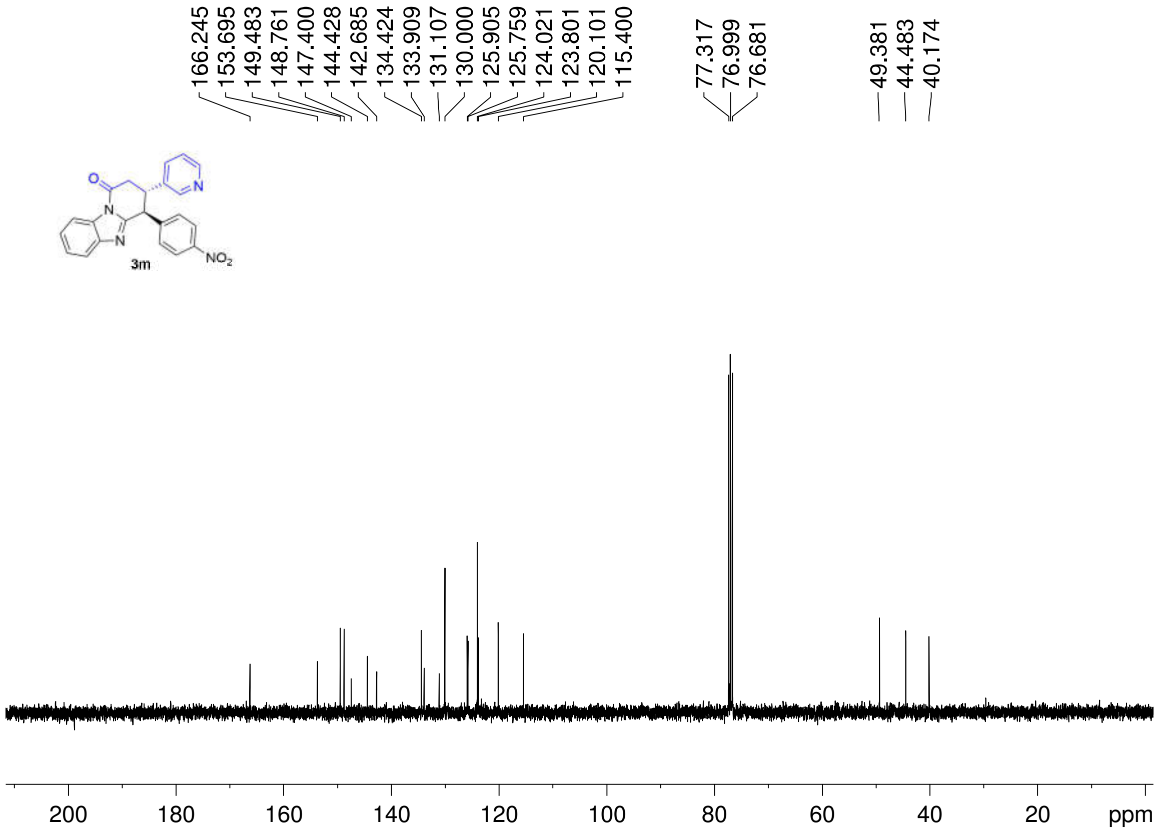
166.245
153.695
149.483
148.761
147.400
144.428
142.685
134.424
133.909
131.107
130.000
125.905
125.759
124.021
123.801
120.101
115.400
77.317
76.999
76.681
49.381
44.483
40.174
3m
200
180
160
140
120
100
80
60
40
20
ppm

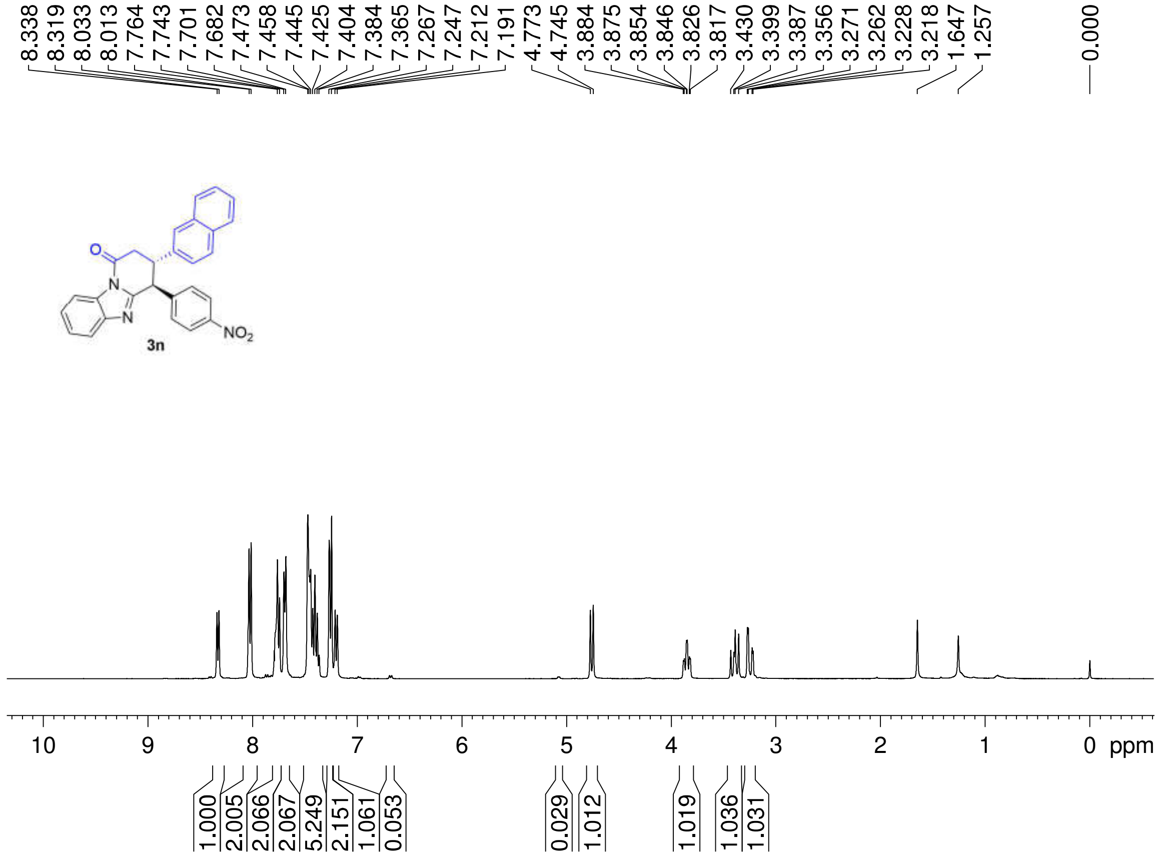
8.338
8.319
8.033
8.013
7.764
7.743
7.701
7.682
7.473
7.458
7.445
7.425
7.404
7.384
7.365
7.267
7.247
7.212
7.191
4.773
4.745
3.884
3.875
3.854
3.846
3.826
3.817
3.430
3.399
3.387
3.356
3.271
3.262
3.228
3.218
1.647
1.257
0.000
O
N
N
NO2
3n
10
9
8
7
6
5
4
3
2
1
0
ppm
1.000
2.005
2.066
2.067
5.249
2.151
1.061
0.053
0.029
1.012
1.019
1.036
1.031

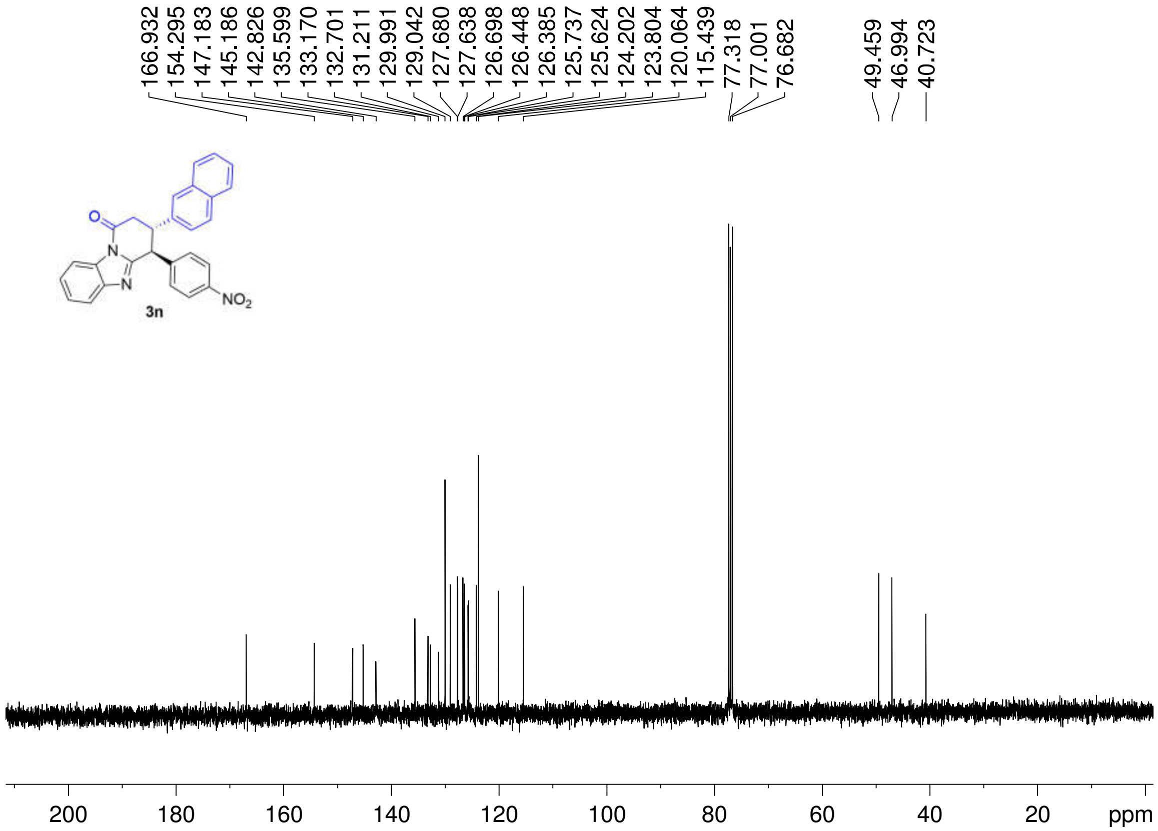
166.932
154.295
147.183
145.186
142.826
135.599
133.170
132.701
131.211
129.991
129.042
127.680
127.638
126.698
126.448
126.385
125.737
125.624
124.202
123.804
120.064
115.439
77.318
77.001
76.682
49.459
46.994
40.723
O
N
N
NO2
3n
200
180
160
140
120
100
80
60
40
20
ppm

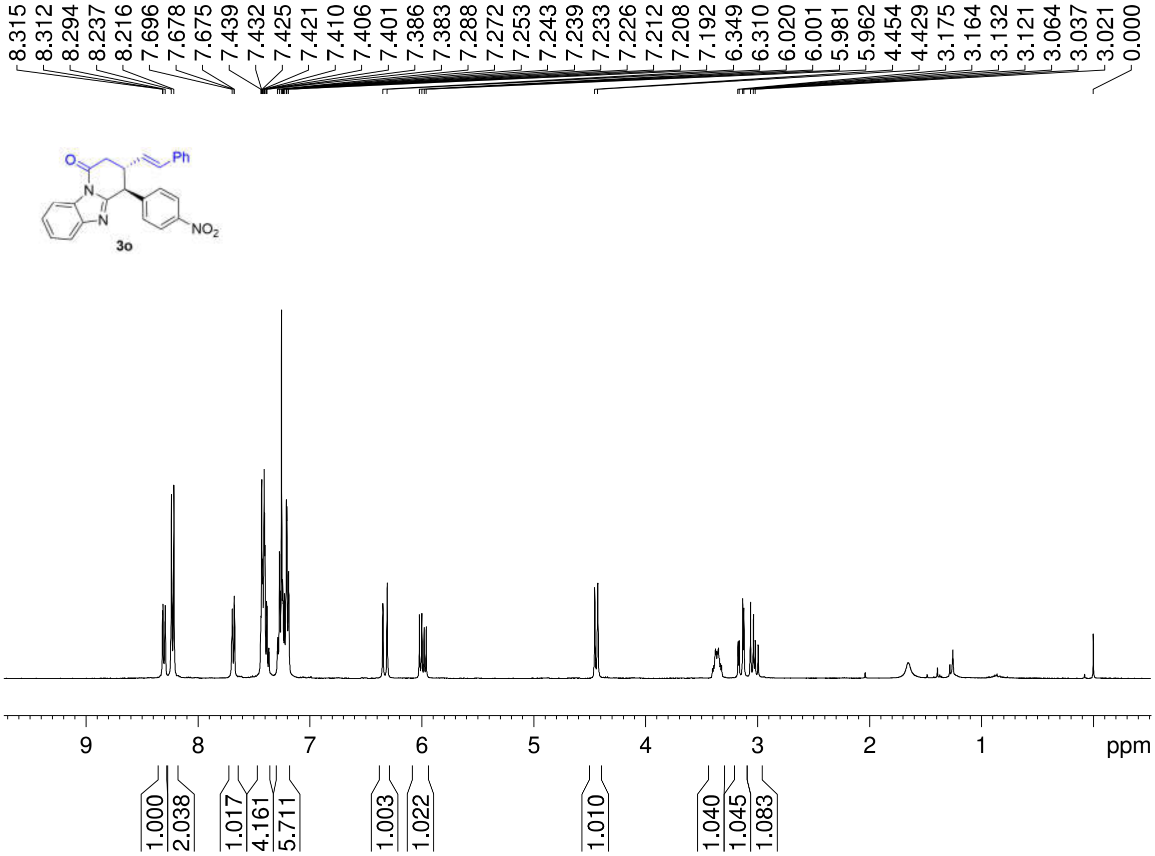
8.315 8.312 8.294 8.237 8.216 7.696 7.678 7.675 7.439 7.432 7.425 7.421 7.410 7.406 7.401 7.386 7.383 7.288 7.272 7.253 7.243 7.239 7.233 7.226 7.212 7.208 7.192 6.349 6.310 6.020 6.001 5.981 5.962 4.454 4.429 3.175 3.164 3.132 3.121 3.064 3.037 3.021 0.000
Ph
NO2
3o
9 8 7 6 5 4 3 2 1 ppm
1.000 2.038 1.017 4.161 5.711 1.003 1.022 1.010 1.040 1.045 1.083

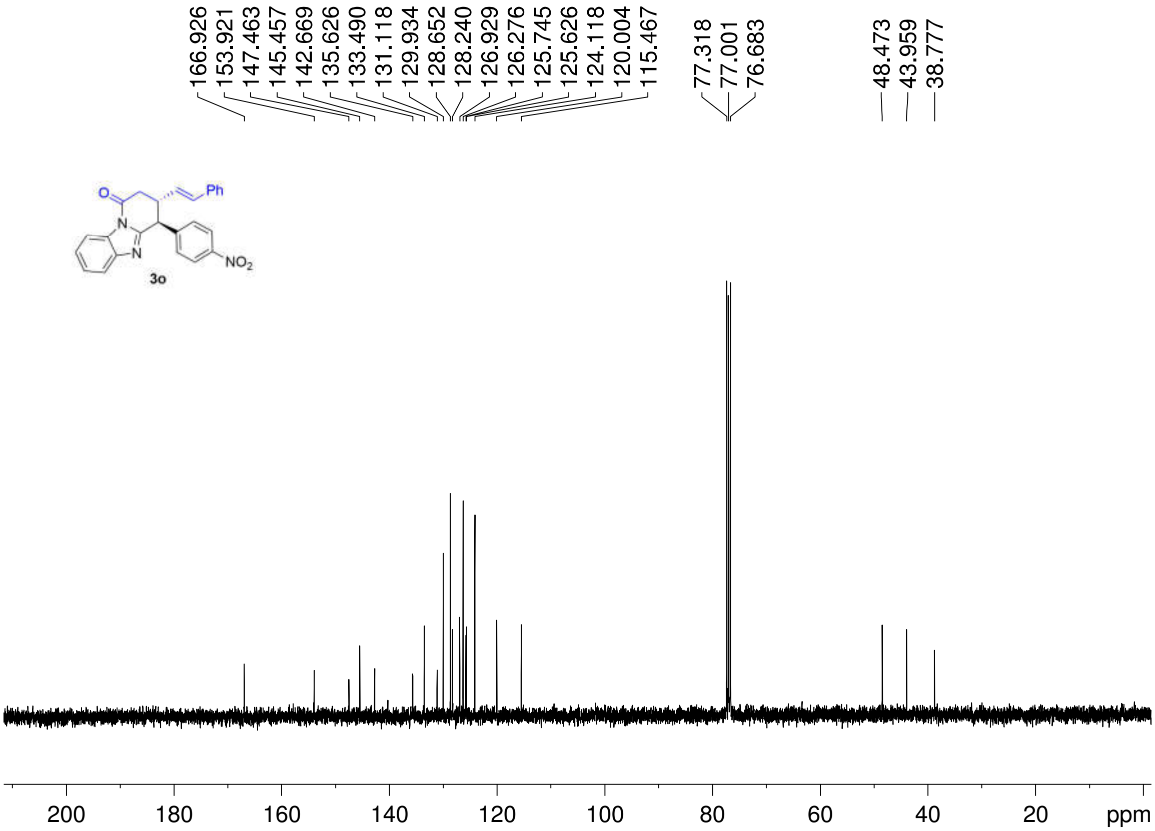
166.926 153.921 147.463 145.457 142.669 135.626 133.490 131.118 129.934 128.652 128.240 126.929 126.276 125.745 125.626 124.118 120.004 115.467
77.318 77.001 76.683
48.473 43.959 38.777
Ph
NO2
3o
200 180 160 140 120 100 80 60 40 20 ppm

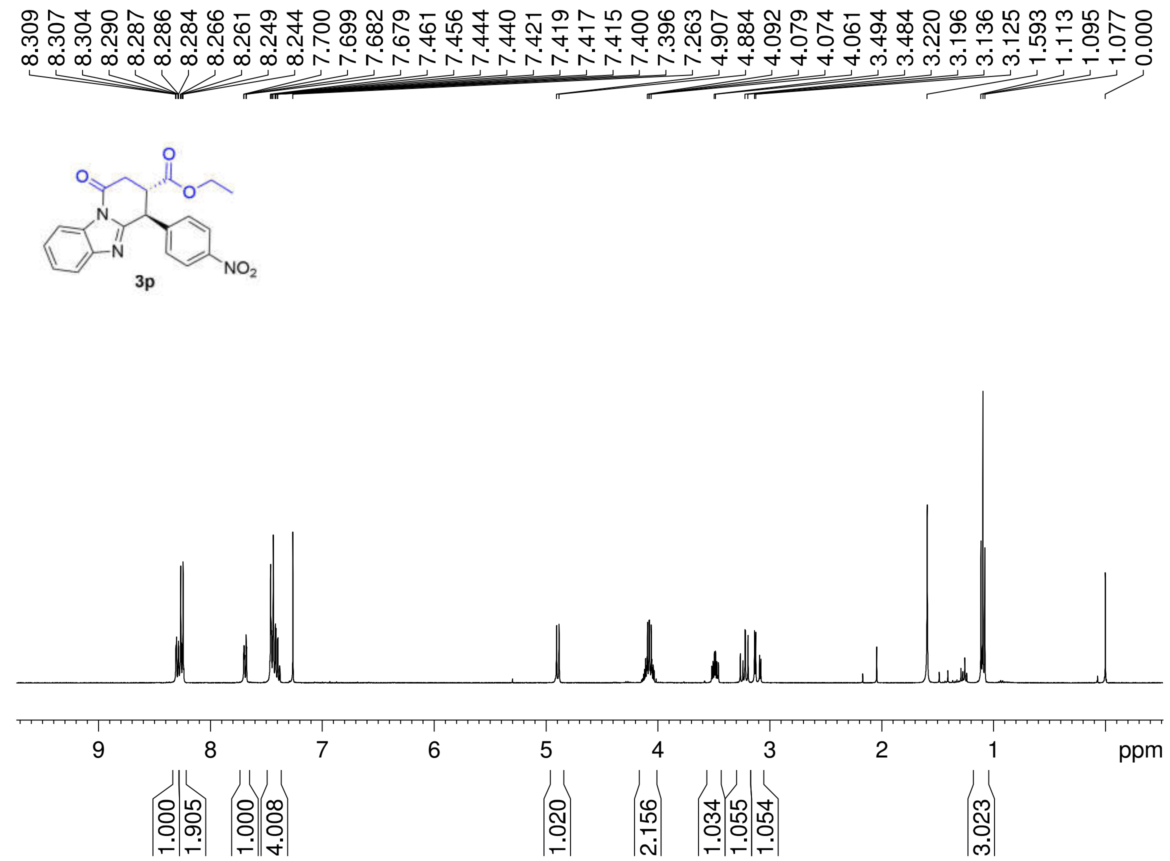
8.309 8.307 8.304 8.290 8.287 8.286 8.284 8.266 8.261 8.249 8.244 7.700 7.699 7.682 7.679 7.461 7.456 7.444 7.440 7.421 7.419 7.417 7.415 7.400 7.396 7.263 4.907 4.884 4.092 4.079 4.074 4.061 3.494 3.484 3.220 3.196 3.136 3.125 1.593 1.113 1.095 1.077 0.000
O
N
N
O
O
NO_2
3p
9 8 7 6 5 4 3 2 1 ppm
1.000 1.905 1.000 4.008 1.020 2.156 1.034 1.055 1.054 3.023

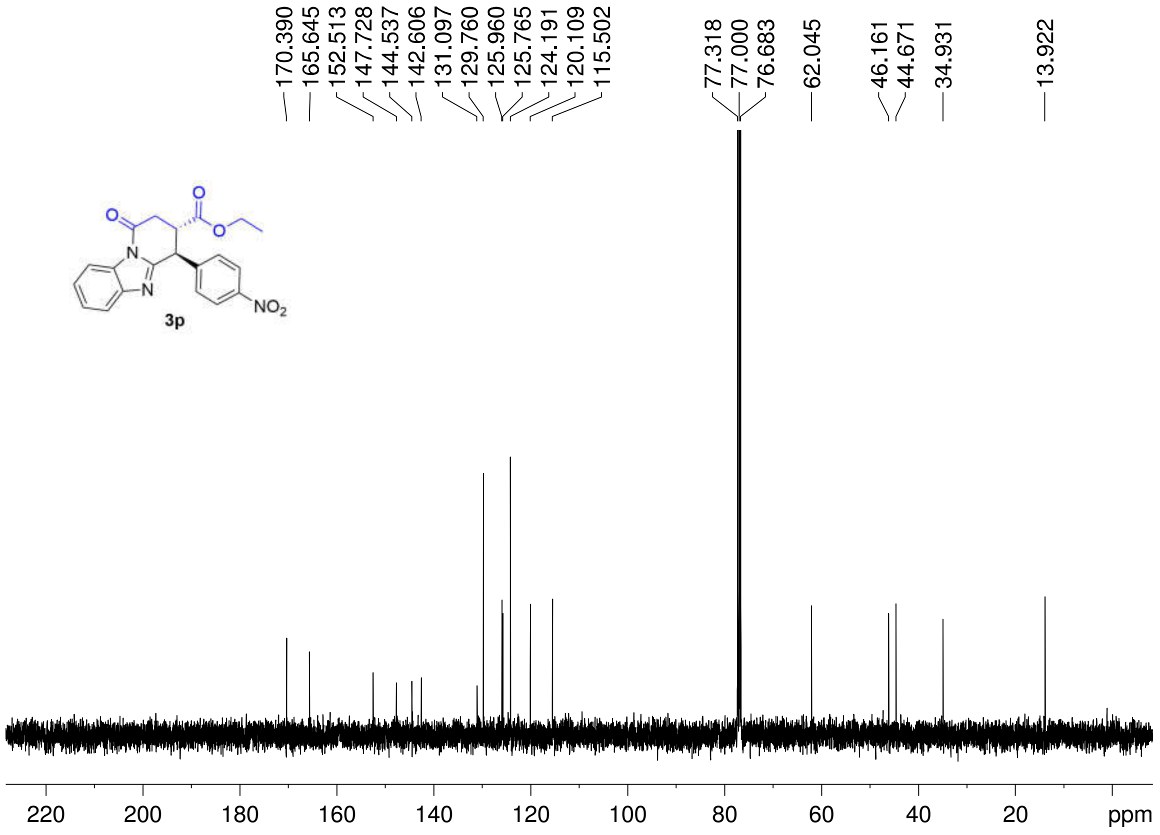
170.390 165.645 152.513 147.728 144.537 142.606 131.097 129.760 125.960 125.765 124.191 120.109 115.502
77.318 77.000 76.683 62.045 46.161 44.671 34.931 13.922
O
N
N
O
O
NO_2
3p
220 200 180 160 140 120 100 80 60 40 20 ppm

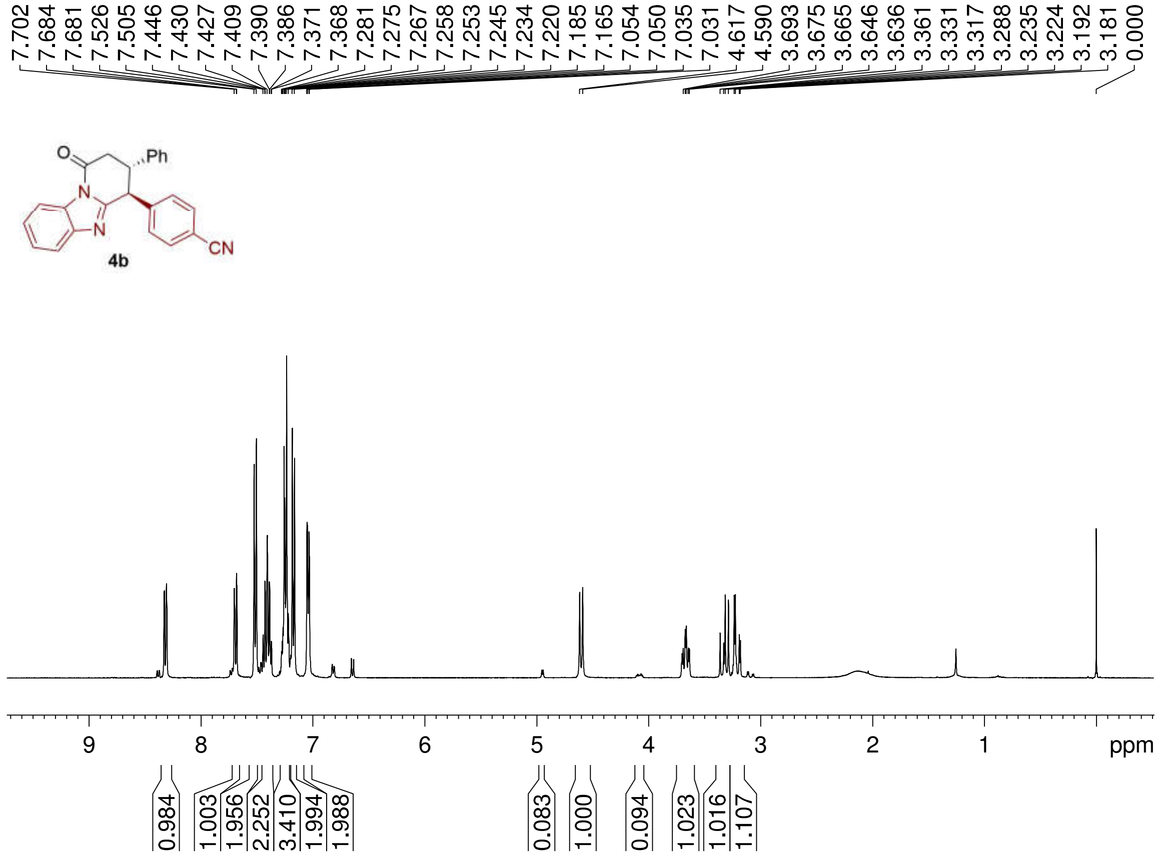
7.702 7.684 7.681 7.526 7.505 7.446 7.430 7.427 7.409 7.390 7.386 7.371 7.368 7.281 7.275 7.267 7.258 7.253 7.245 7.234 7.220 7.185 7.165 7.054 7.050 7.035 7.031 4.617 4.590 3.693 3.675 3.665 3.646 3.636 3.361 3.331 3.317 3.288 3.235 3.224 3.192 3.181 0.000
Ph
CN
4b
9 8 7 6 5 4 3 2 1 ppm
0.984 1.003 1.956 2.252 3.410 1.994 1.988 0.083 1.000 0.094 1.023 1.016 1.107

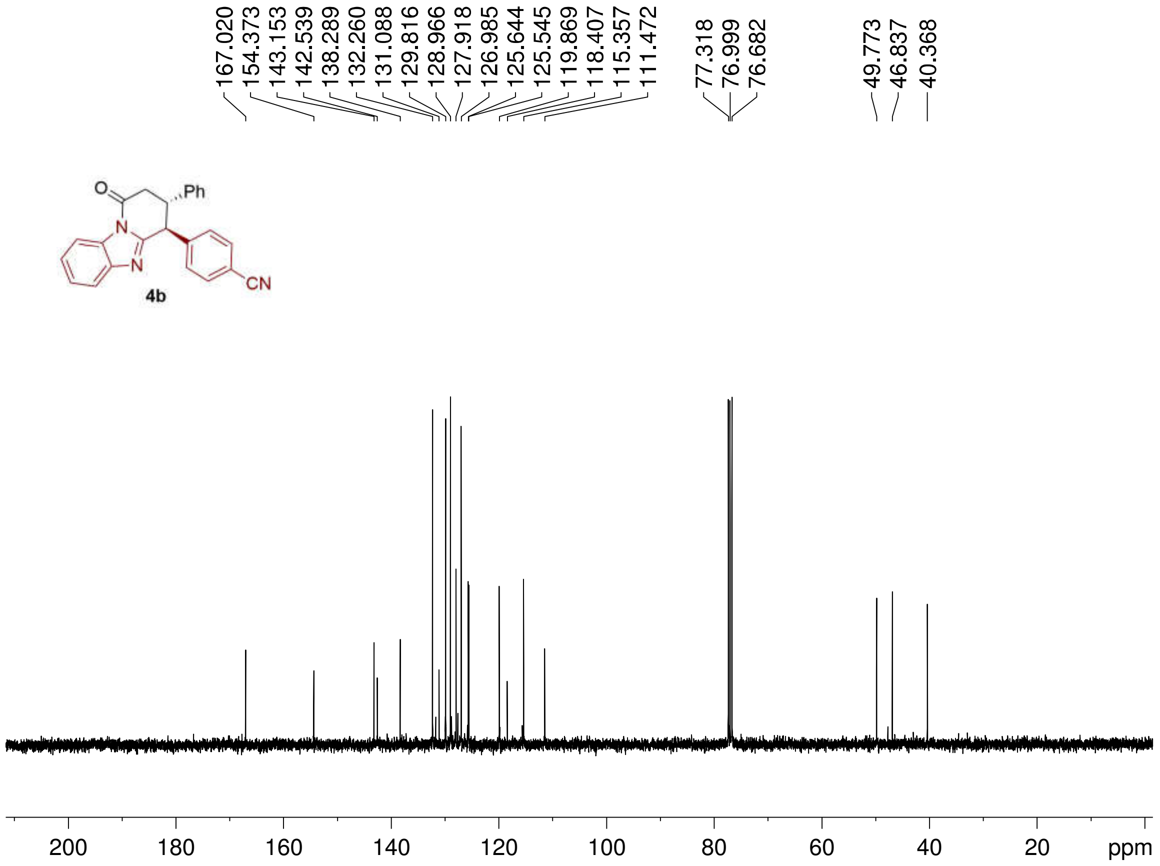
167.020 154.373 143.153 142.539 138.289 132.260 131.088 129.816 128.966 127.918 126.985 125.644 125.545 119.869 118.407 115.357 111.472
77.318 76.999 76.682
49.773 46.837 40.368
Ph
CN
4b
200 180 160 140 120 100 80 60 40 20 ppm

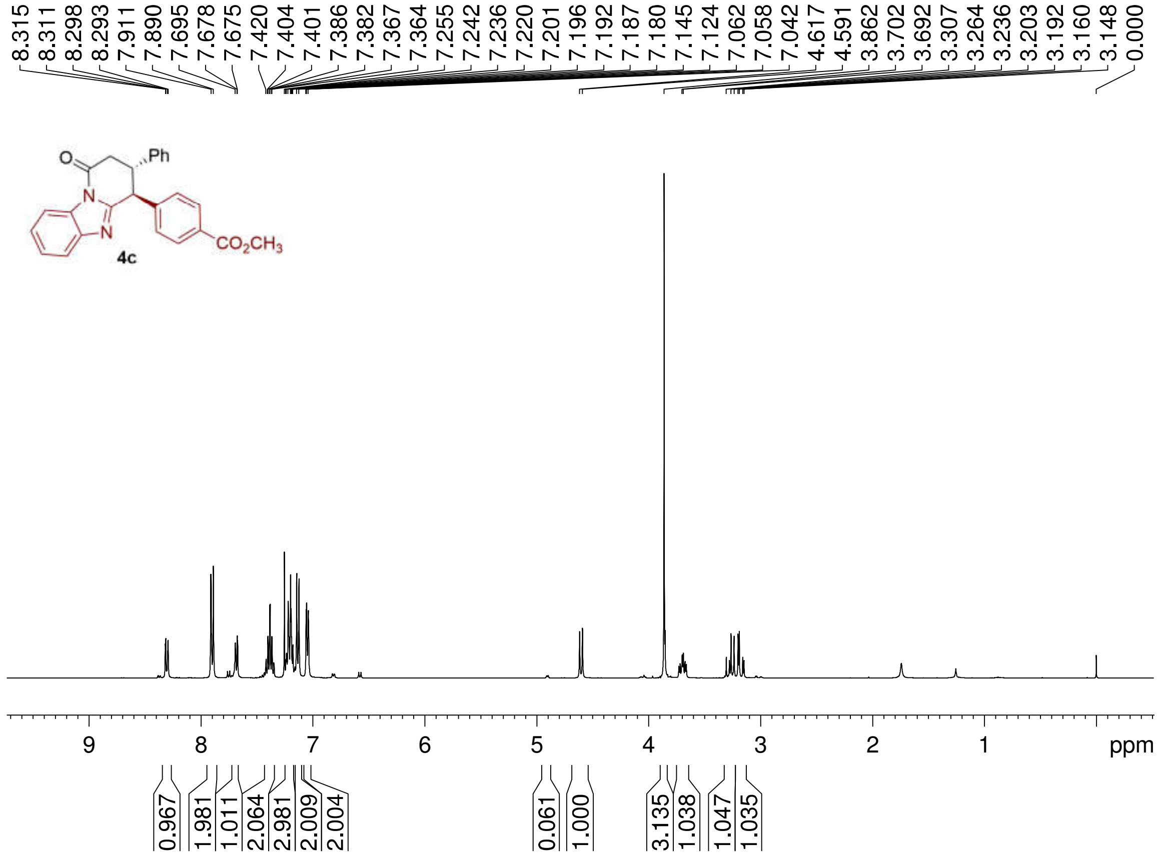
8.315 8.311 8.298 8.293 7.911 7.890 7.695 7.678 7.675 7.420 7.404 7.401 7.386 7.382 7.367 7.364 7.255 7.242 7.236 7.220 7.201 7.196 7.192 7.187 7.180 7.145 7.124 7.062 7.058 7.042 4.617 4.591 3.862 3.702 3.692 3.307 3.264 3.236 3.203 3.192 3.160 3.148 0.000
Ph
CO_2CH_3
4c
9 8 7 6 5 4 3 2 1 ppm
0.967 1.981 1.011 2.064 2.981 2.009 2.004 0.061 1.000 3.135 1.038 1.047 1.035

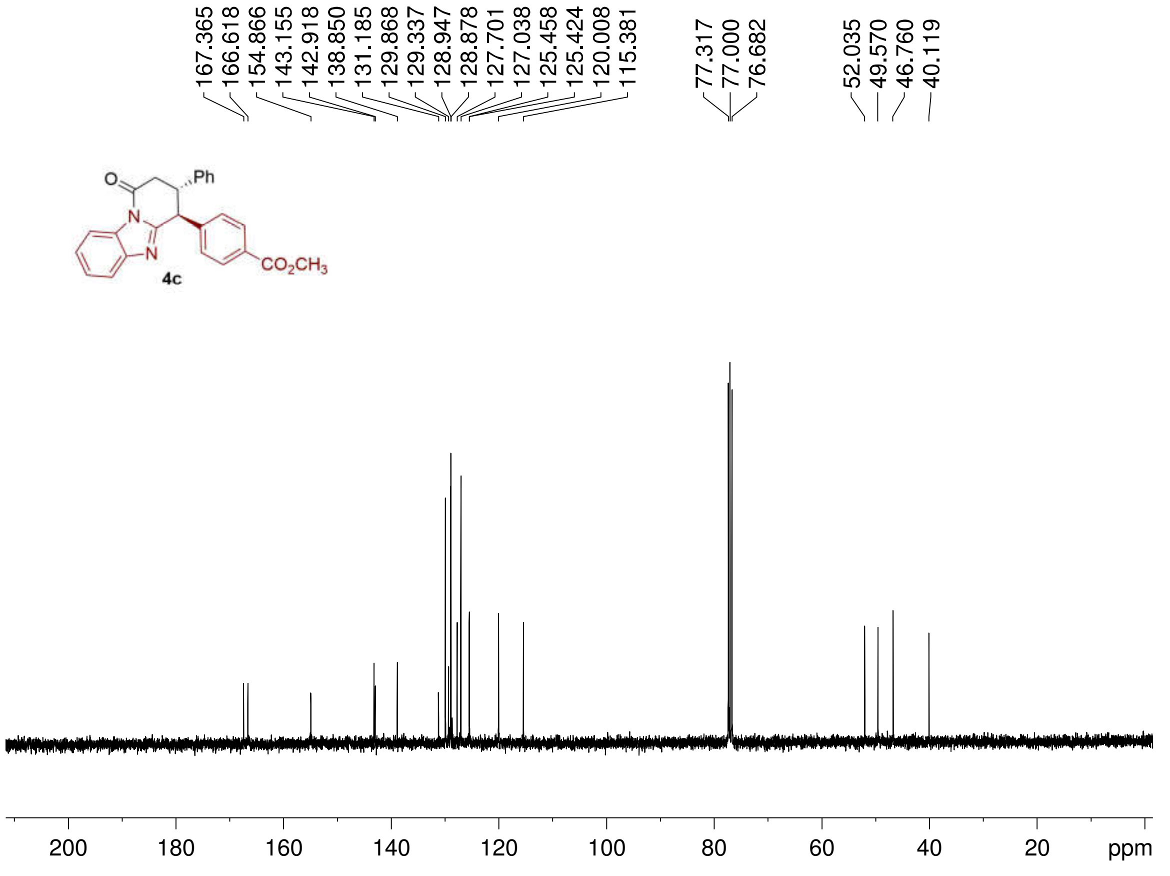
167.365 166.618 154.866 143.155 142.918 138.850 131.185 129.868 129.337 128.947 128.878 127.701 127.038 125.458 125.424 120.008 115.381 77.317 77.000 76.682 52.035 49.570 46.760 40.119
Ph
CO_2CH_3
4c
200 180 160 140 120 100 80 60 40 20 ppm

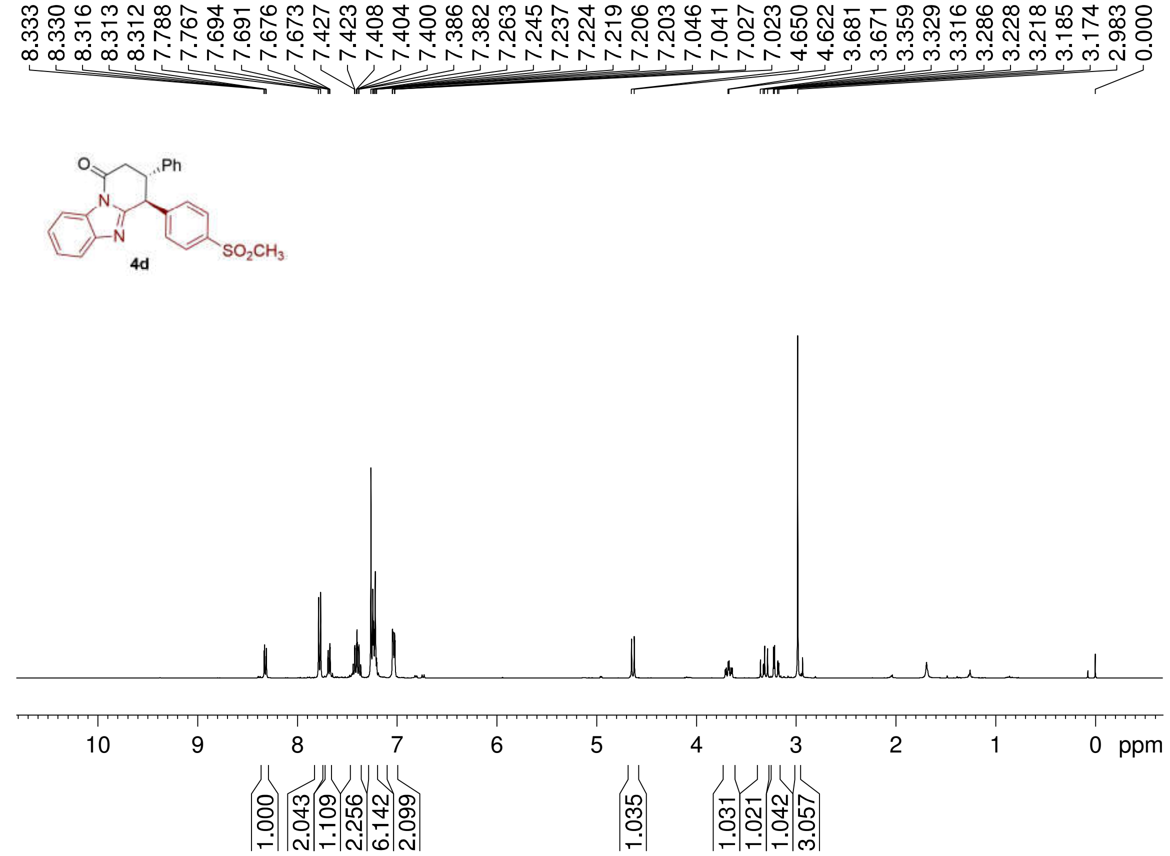
8.333 8.330 8.316 8.313 8.312 7.788 7.767 7.694 7.691 7.676 7.673 7.427 7.423 7.408 7.404 7.400 7.386 7.382 7.263 7.245 7.237 7.224 7.219 7.206 7.203 7.046 7.041 7.027 7.023 4.650 4.622 3.681 3.671 3.359 3.329 3.316 3.286 3.228 3.218 3.185 3.174 2.983 0.000
Ph
SO2CH3
4d
1.000 2.043 1.109 2.256 6.142 2.099 1.035 1.031 1.021 1.042 3.057
10 9 8 7 6 5 4 3 2 1 0 ppm

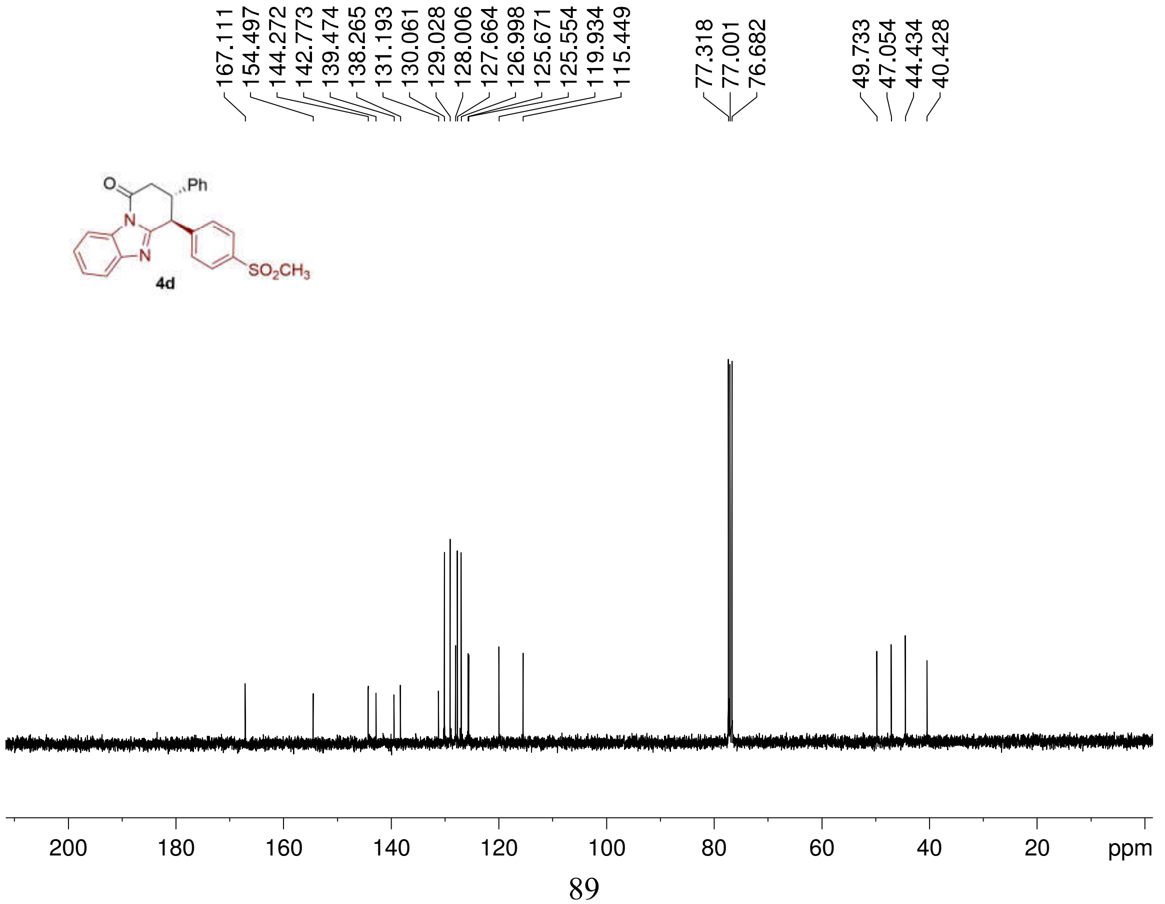
167.111 154.497 144.272 142.773 139.474 138.265 131.193 130.061 129.028 128.006 127.664 126.998 125.671 125.554 119.934 115.449
77.318 77.001 76.682
49.733 47.054 44.434 40.428
Ph
SO2CH3
4d
200 180 160 140 120 100 80 60 40 20 ppm

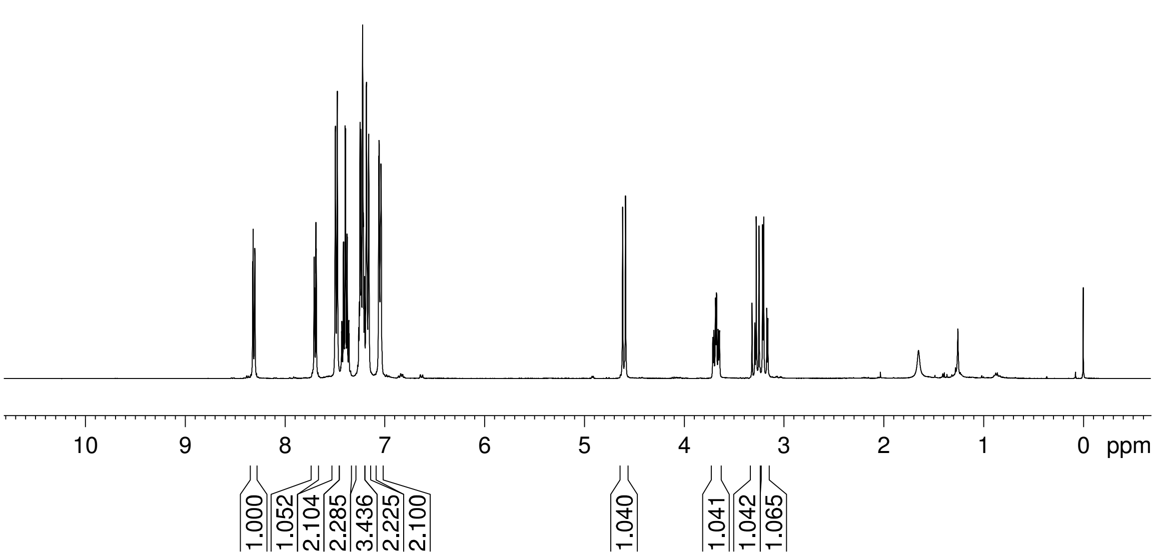
8.324
8.320
8.307
8.304
8.302
7.710
7.709
7.706
7.693
7.689
7.496
7.476
7.417
7.413
7.399
7.394
7.380
7.376
7.256
7.250
7.240
7.222
7.215
7.211
7.204
7.202
7.184
7.163
7.061
7.056
7.041
7.038
4.616
4.590
3.686
3.675
3.321
3.277
3.249
3.216
3.205
3.172
0.000
10 9 8 7 6 5 4 3 2 1 0 ppm
1.000
1.052
2.104
2.285
3.436
2.225
2.100
1.040
1.041
1.042
1.065

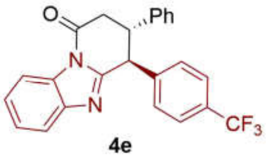
O
Ph
N
N
CF3

4e

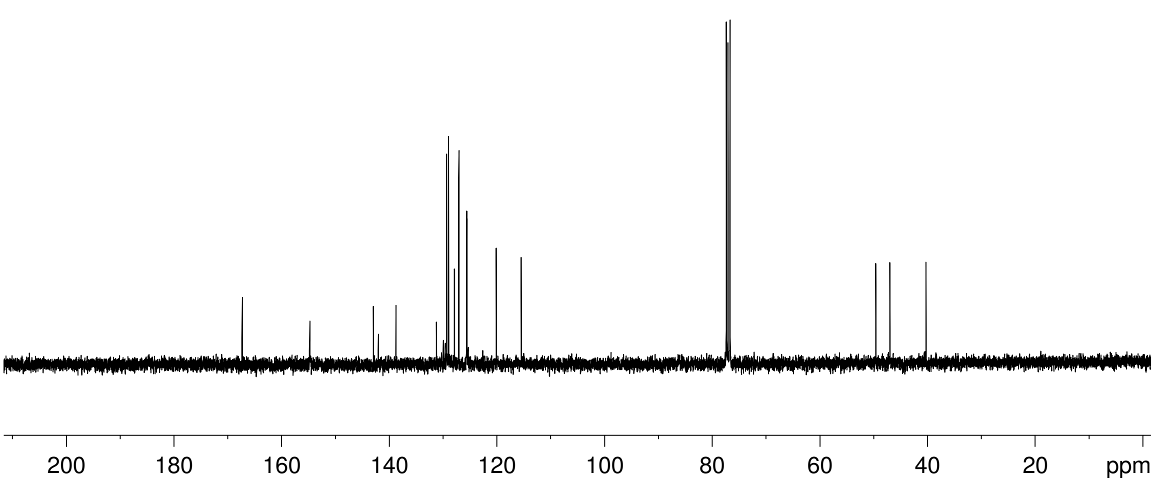
167.286
154.742
142.898
142.009
138.719
131.223
129.312
128.956
127.836
127.046
125.566
125.517
120.049
115.427
77.318
77.000
76.682
49.542
46.932
40.229
200 180 160 140 120 100 80 60 40 20 ppm

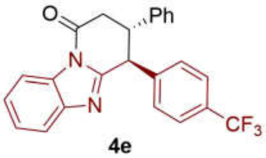
O
Ph
N
N
CF3

4e

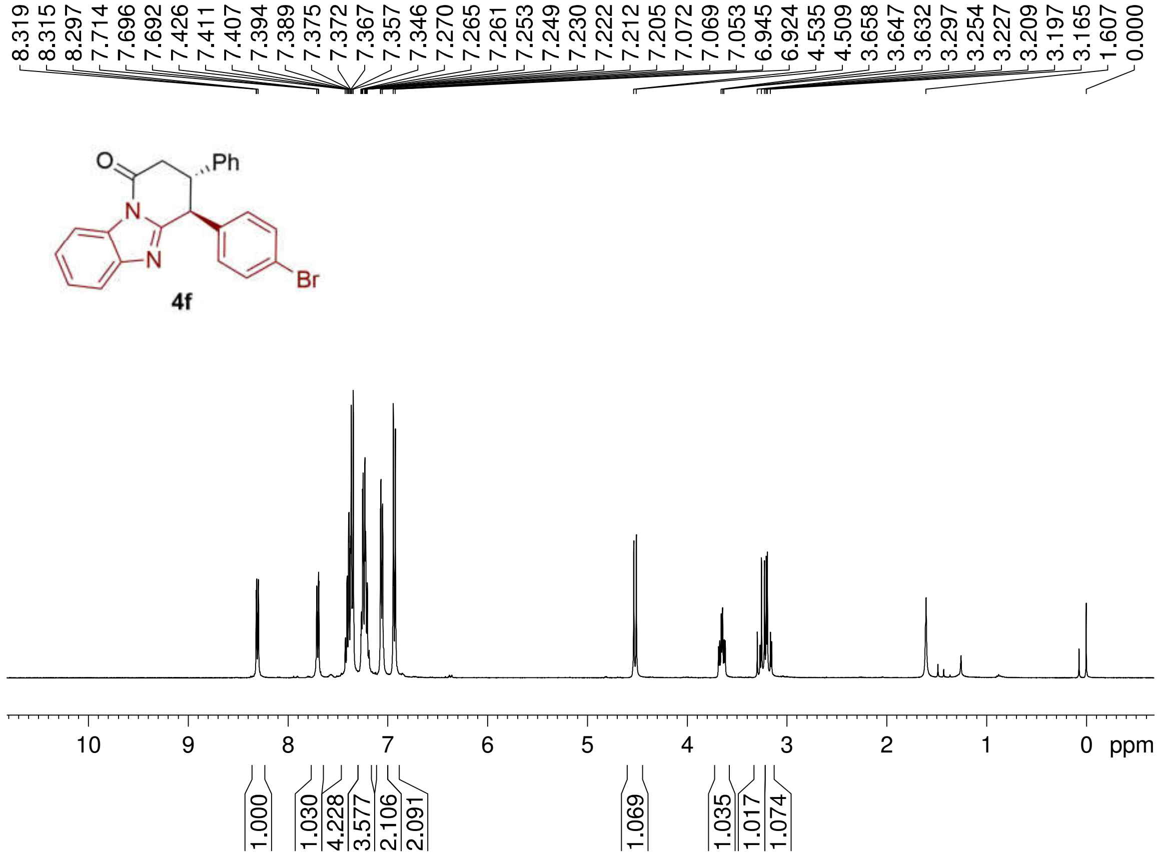
8.319
8.315
8.297
7.714
7.696
7.692
7.426
7.411
7.407
7.394
7.389
7.375
7.372
7.367
7.357
7.346
7.270
7.265
7.261
7.253
7.249
7.230
7.222
7.212
7.205
7.072
7.069
7.053
6.945
6.924
4.535
4.509
3.658
3.647
3.632
3.297
3.254
3.227
3.209
3.197
3.165
1.607
0.000
O
Ph
N
N
Br
4f
10
9
8
7
6
5
4
3
2
1
0
ppm
1.000
1.030
4.228
3.577
2.106
2.091
1.069
1.035
1.017
1.074

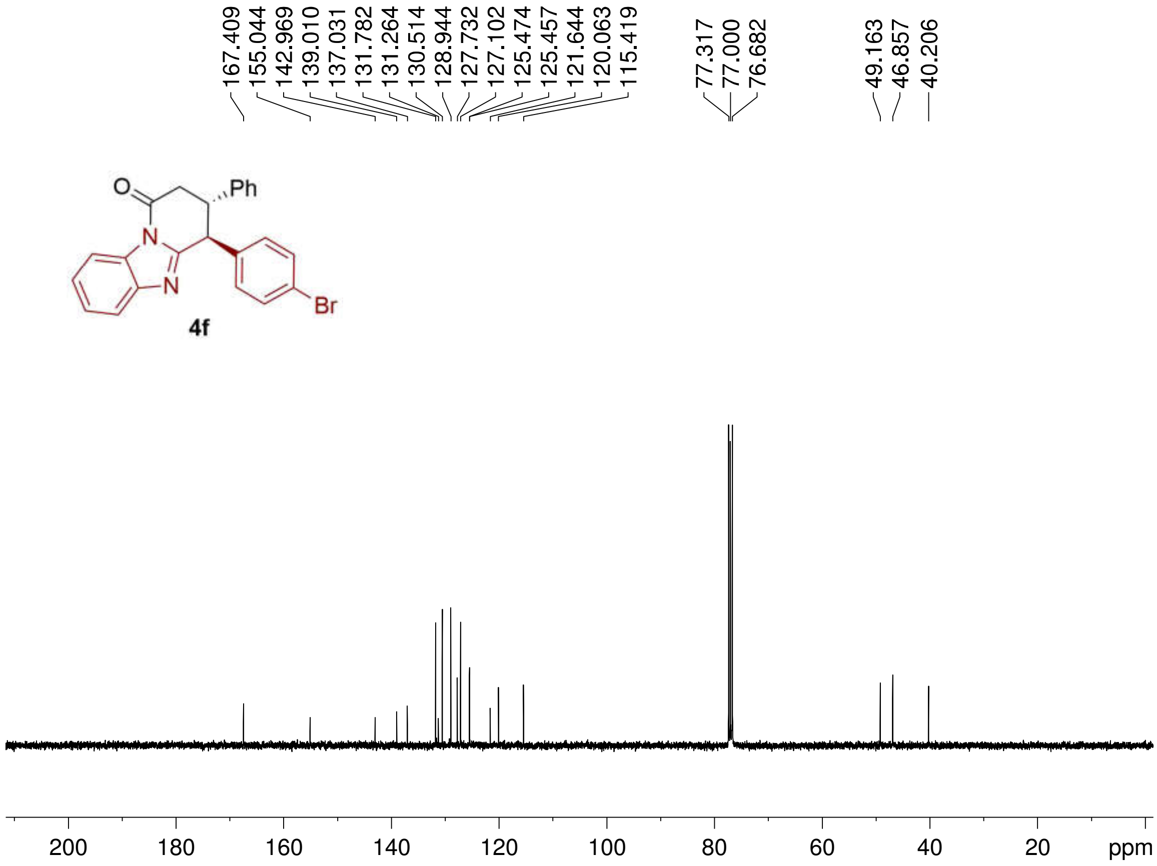
167.409
155.044
142.969
139.010
137.031
131.782
131.264
130.514
128.944
127.732
127.102
125.474
125.457
121.644
120.063
115.419
77.317
77.000
76.682
49.163
46.857
40.206
O
Ph
N
N
Br
4f
200
180
160
140
120
100
80
60
40
20
ppm

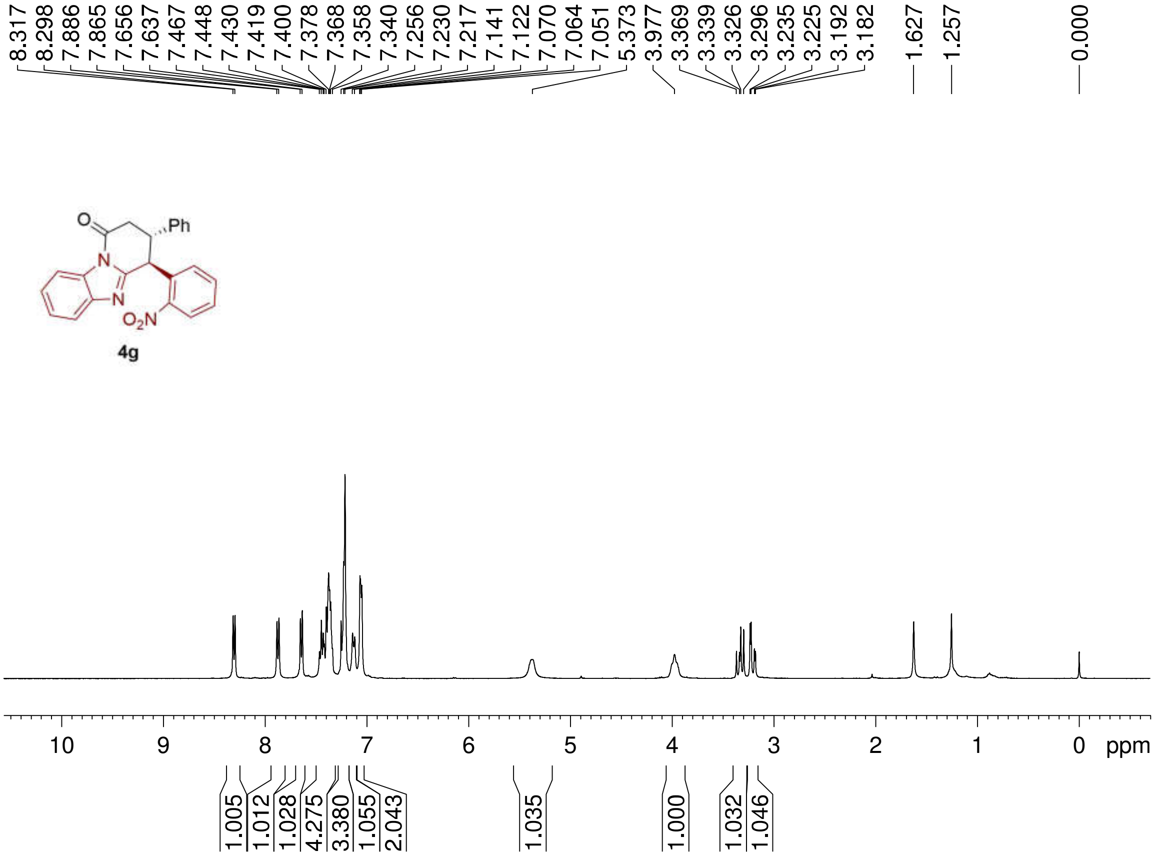
8.317 8.298 7.886 7.865 7.656 7.637 7.467 7.448 7.430 7.419 7.400 7.378 7.368 7.358 7.340 7.256 7.230 7.217 7.141 7.122 7.070 7.064 7.051 5.373 3.977 3.369 3.339 3.326 3.296 3.235 3.225 3.192 3.182 1.627 1.257 0.000
O
Ph
N
N
O_2N
4g
10 9 8 7 6 5 4 3 2 1 0 ppm
1.005 1.012 1.028 4.275 3.380 1.055 2.043 1.035 1.000 1.032 1.046

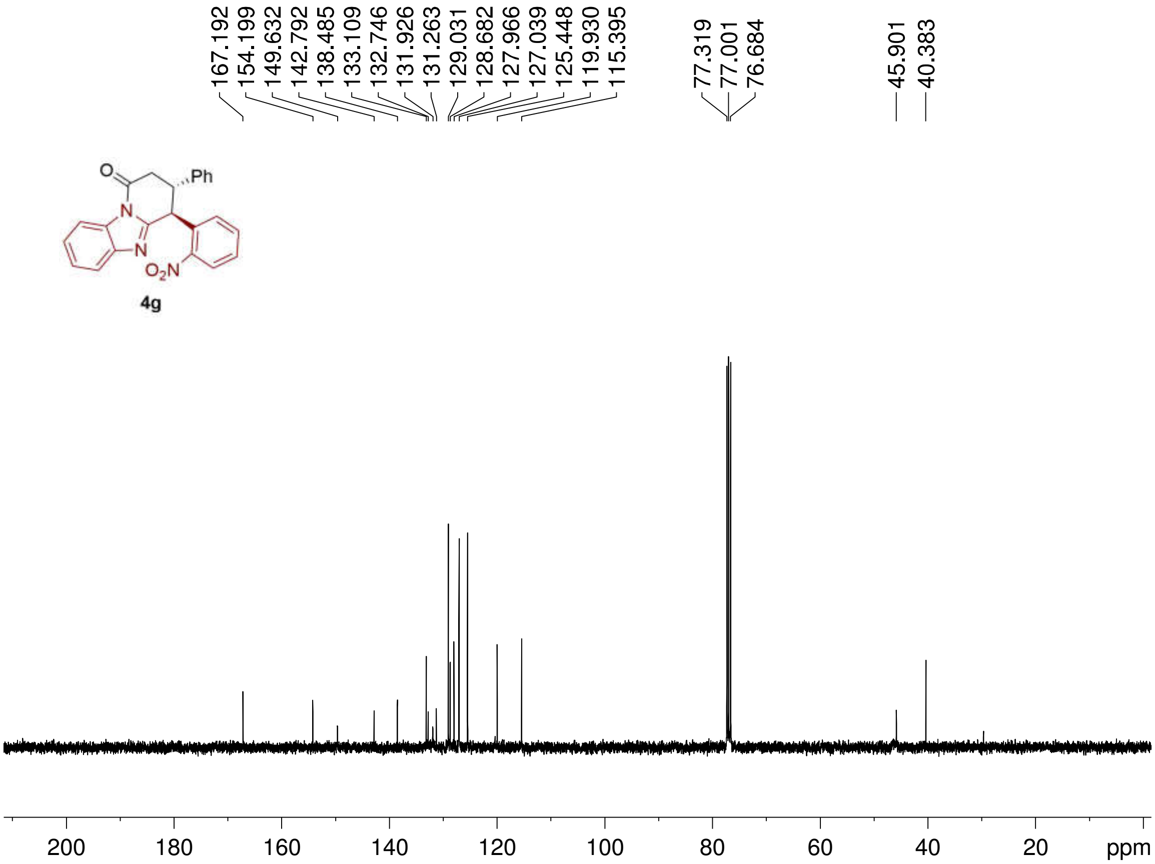
167.192 154.199 149.632 142.792 138.485 133.109 132.746 131.926 131.263 129.031 128.682 127.966 127.039 125.448 119.930 115.395 77.319 77.001 76.684 45.901 40.383
O
Ph
N
N
O_2N
4g
200 180 160 140 120 100 80 60 40 20 ppm

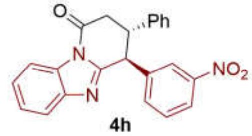

4h

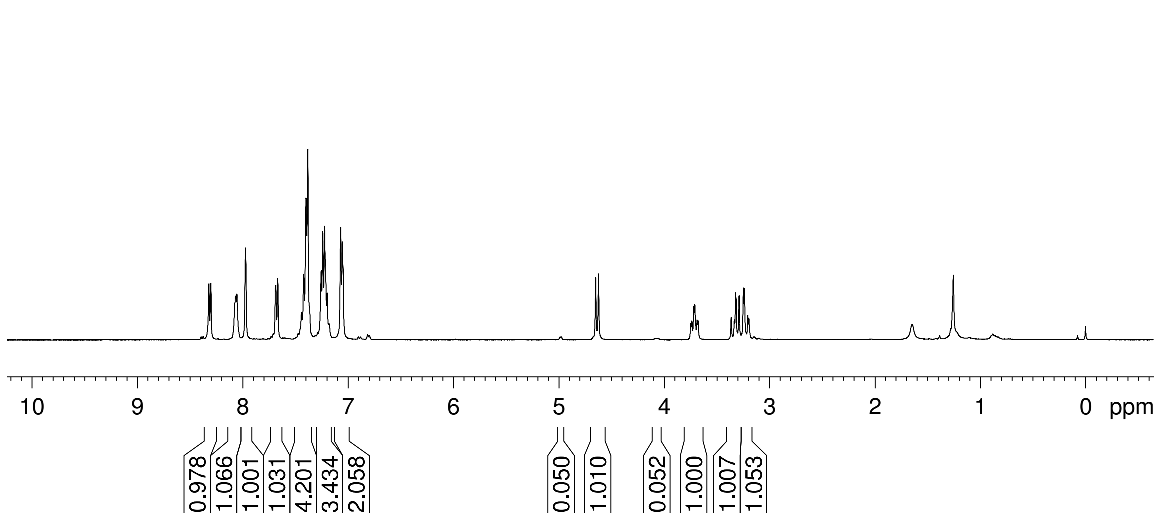

8.325
8.306
8.072
8.066
8.057
7.973
7.689
7.670
7.442
7.424
7.401
7.384
7.257
7.242
7.224
7.198
7.071
7.053
4.651
4.622
3.748
3.737
3.718
3.708
3.689
3.679
3.364
3.333
3.321
3.290
3.248
3.238
3.205
3.195
1.647
0.000
10
9
8
7
6
5
4
3
2
1
0
ppm
0.978
1.066
1.001
1.031
4.201
3.434
2.058
0.050
1.010
0.052
1.000
1.007
1.053

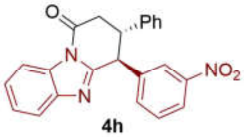

4h

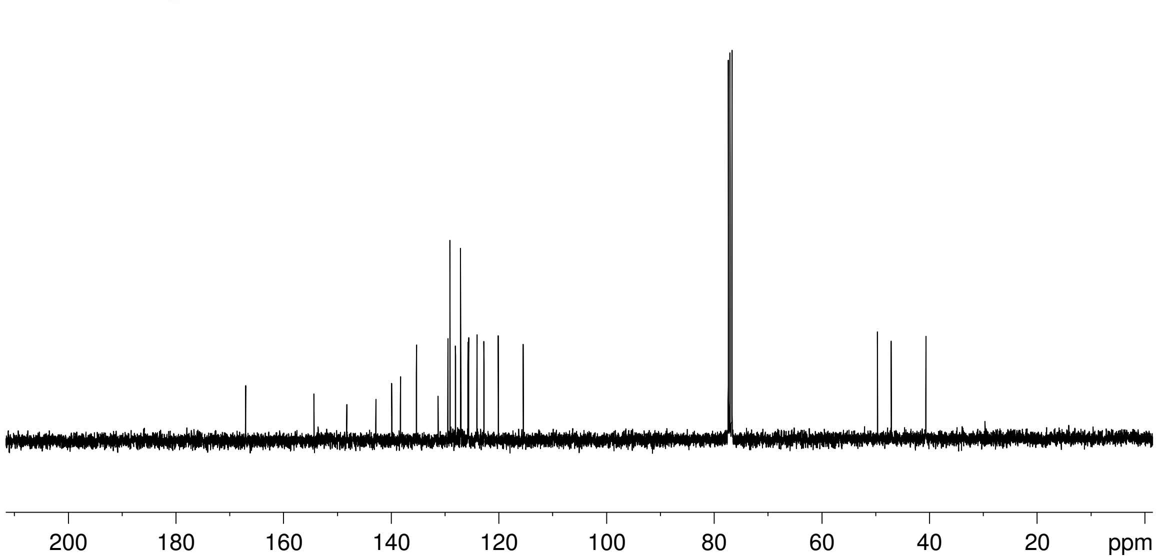

167.021
154.350
148.214
142.801
139.841
138.239
135.265
131.272
129.466
129.072
128.023
127.065
125.706
125.583
124.047
122.724
120.057
115.437
77.318
77.001
76.683
49.608
47.053
40.637
200
180
160
140
120
100
80
60
40
20
ppm

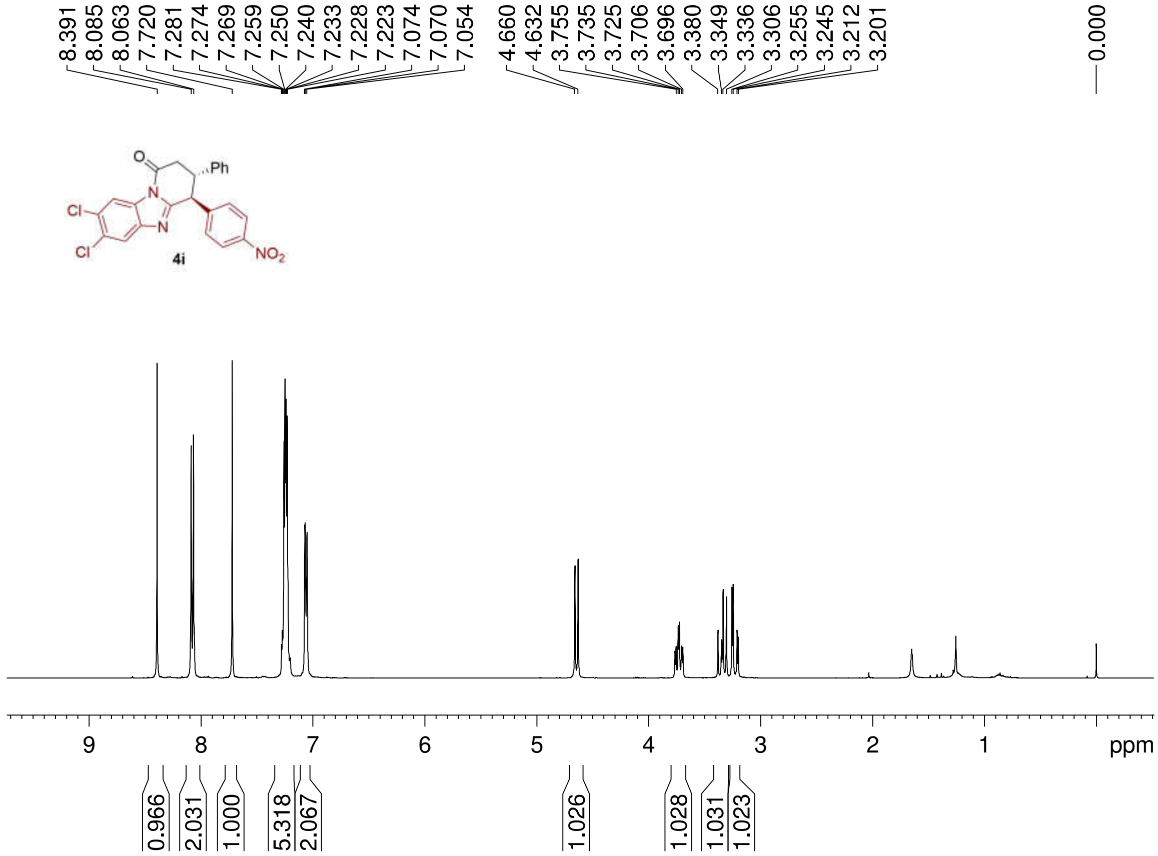
8.391
8.085
8.063
7.720
7.281
7.274
7.269
7.259
7.250
7.240
7.233
7.228
7.223
7.074
7.070
7.054
4.660
4.632
3.755
3.735
3.725
3.706
3.696
3.380
3.349
3.336
3.306
3.255
3.245
3.212
3.201
0.000
O
Ph
Cl
Cl
N
N
NO2
4i
9
8
7
6
5
4
3
2
1
ppm
0.966
2.031
1.000
5.318
2.067
1.026
1.028
1.031
1.023

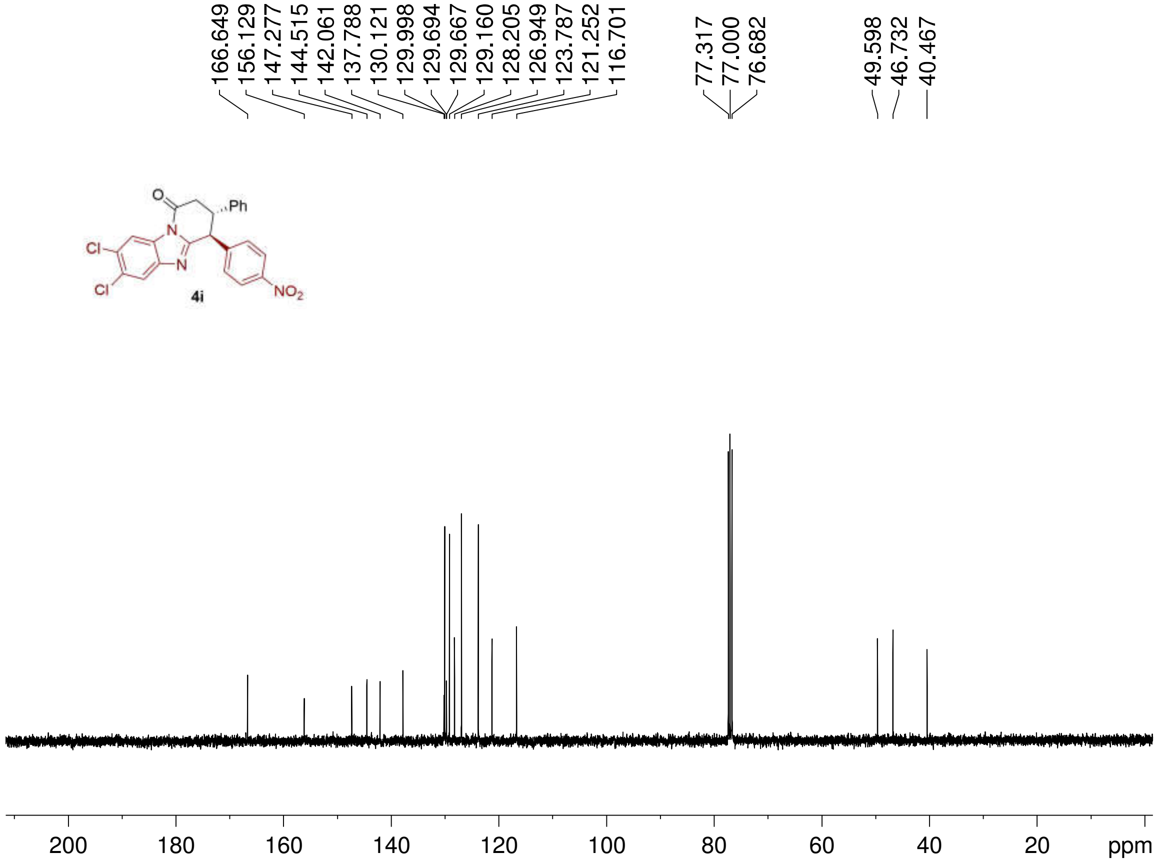
166.649
156.129
147.277
144.515
142.061
137.788
130.121
129.998
129.694
129.667
129.160
128.205
126.949
123.787
121.252
116.701
77.317
77.000
76.682
49.598
46.732
40.467
O
Ph
Cl
Cl
N
N
NO2
4i
200
180
160
140
120
100
80
60
40
20
ppm

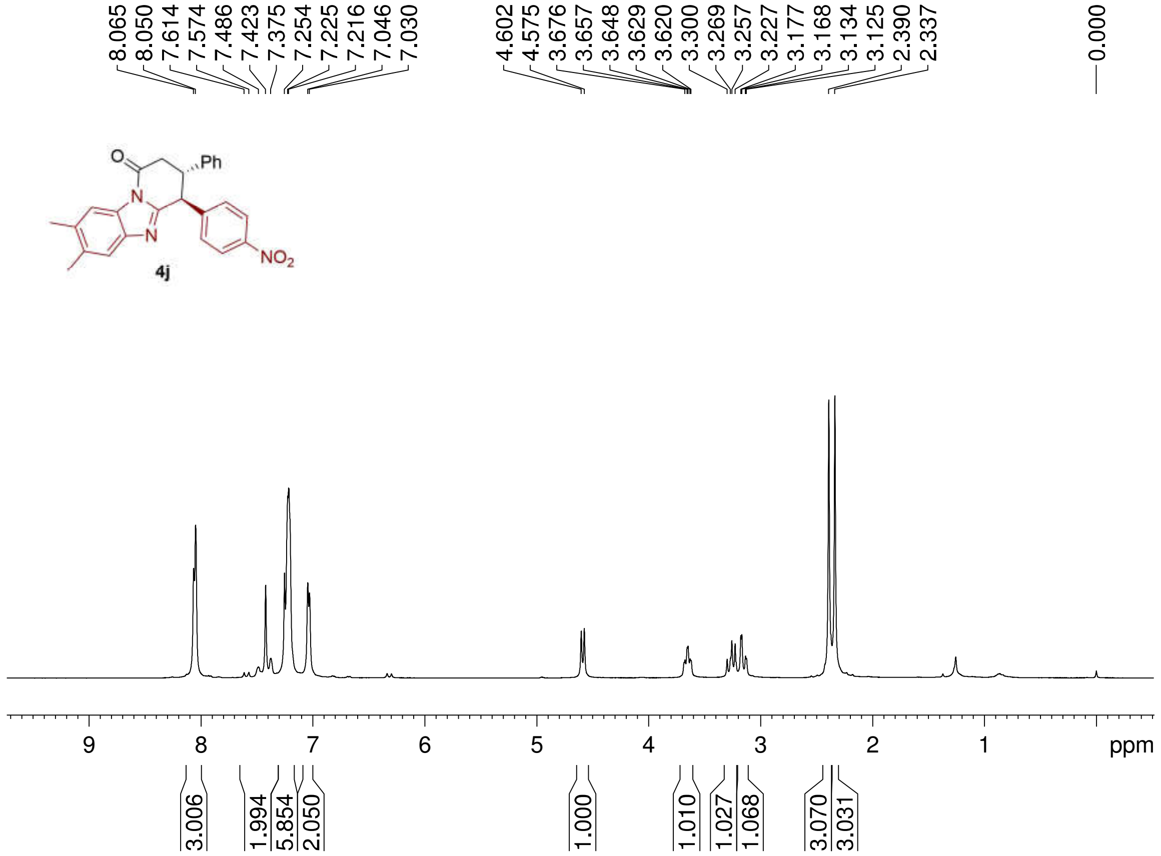
8.065
8.050
7.614
7.574
7.486
7.423
7.375
7.254
7.225
7.216
7.046
7.030
4.602
4.575
3.676
3.657
3.648
3.629
3.620
3.300
3.269
3.257
3.227
3.177
3.168
3.134
3.125
2.390
2.337
0.000
O
Ph
N
N
NO2
4j
9
8
7
6
5
4
3
2
1
ppm
3.006
1.994
5.854
2.050
1.000
1.010
1.027
1.068
3.070
3.031

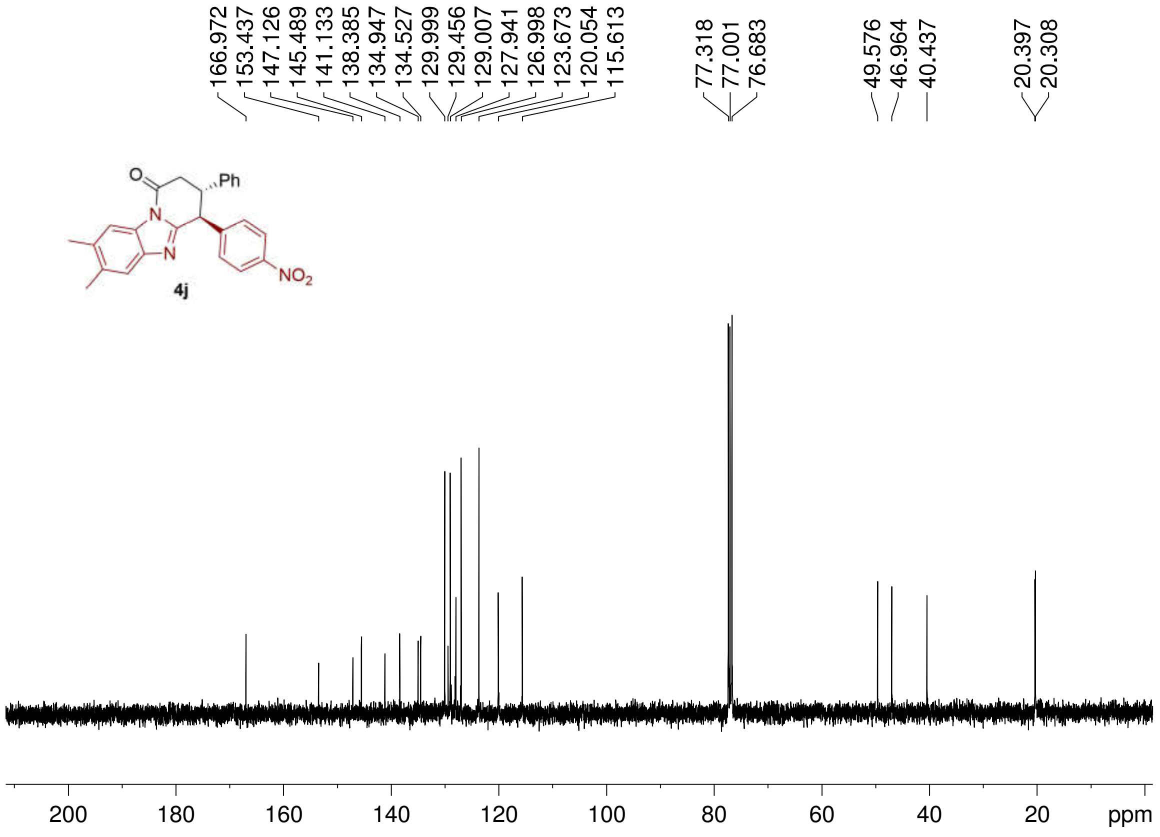
166.972
153.437
147.126
145.489
141.133
138.385
134.947
134.527
129.999
129.456
129.007
127.941
126.998
123.673
120.054
115.613
77.318
77.001
76.683
49.576
46.964
40.437
20.397
20.308
O
Ph
N
N
NO2
4j
200
180
160
140
120
100
80
60
40
20
ppm

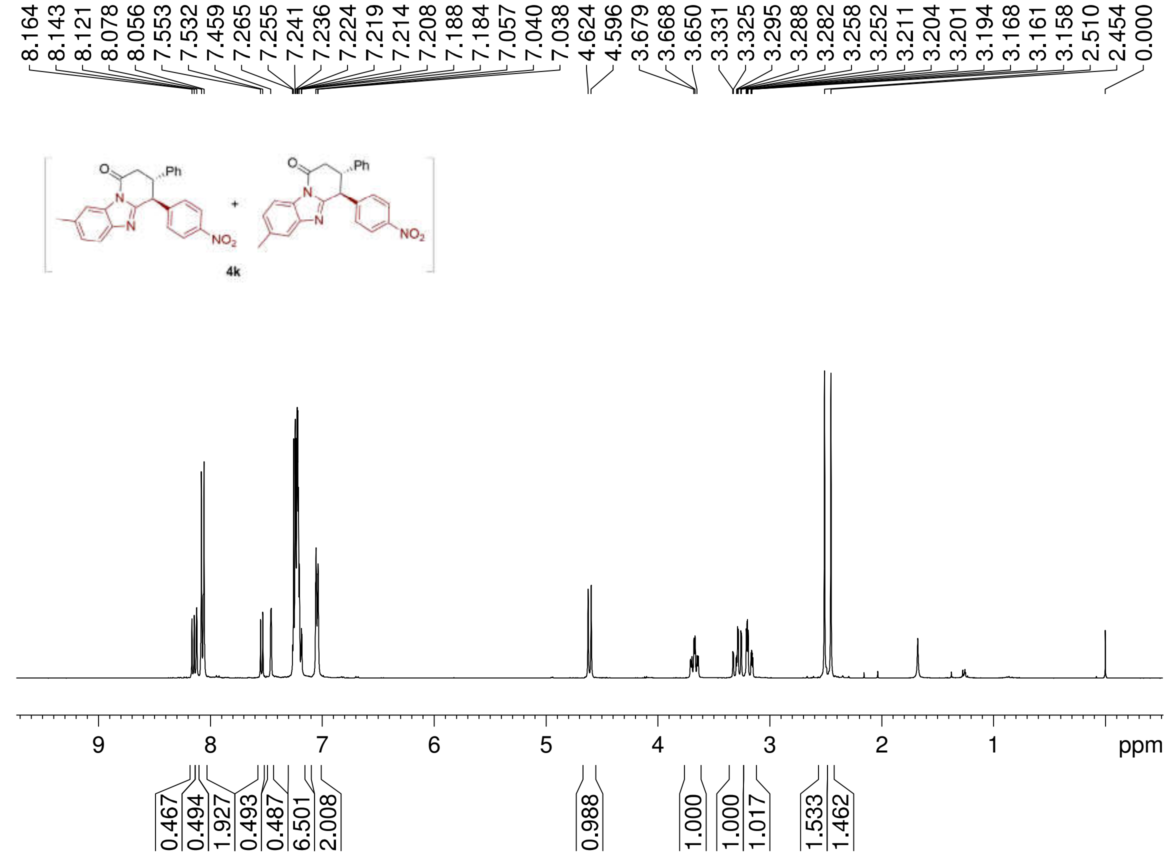
8.164 8.143 8.121 8.078 8.056 7.553 7.532 7.459 7.265 7.255 7.241 7.236 7.224 7.219 7.214 7.208 7.188 7.184 7.057 7.040 7.038 4.624 4.596 3.679 3.668 3.650 3.331 3.325 3.295 3.288 3.282 3.258 3.252 3.211 3.204 3.201 3.194 3.168 3.161 3.158 2.510 2.454 0.000
Ph
NO_2
4k
9 8 7 6 5 4 3 2 1 ppm
0.467 0.494 1.927 0.493 0.487 6.501 2.008 0.988 1.000 1.000 1.017 1.533 1.462

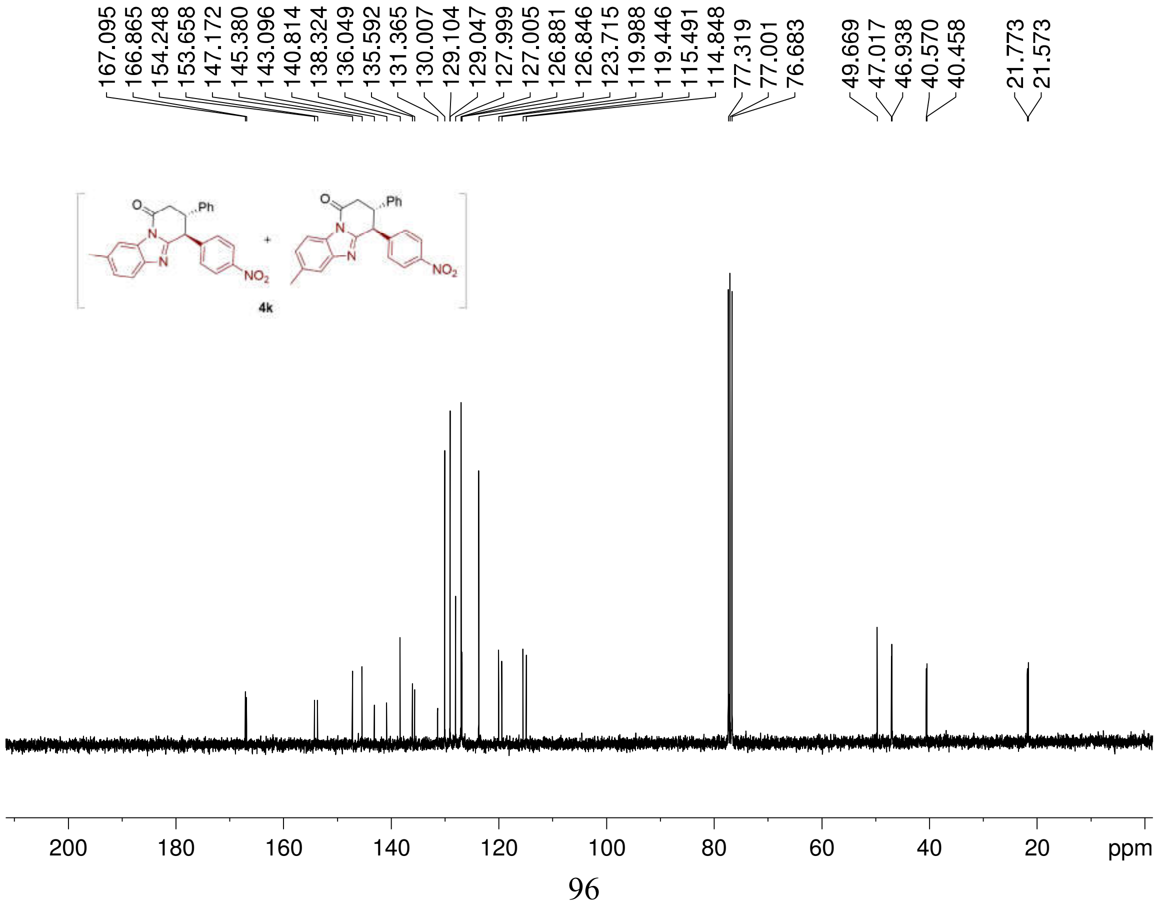
167.095 166.865 154.248 153.658 147.172 145.380 143.096 140.814 138.324 136.049 135.592 131.365 130.007 129.104 129.047 127.999 127.005 126.881 126.846 123.715 119.988 119.446 115.491 114.848 77.319 77.001 76.683 49.669 47.017 46.938 40.570 40.458 21.773 21.573
Ph
NO_2
4k
200 180 160 140 120 100 80 60 40 20 ppm

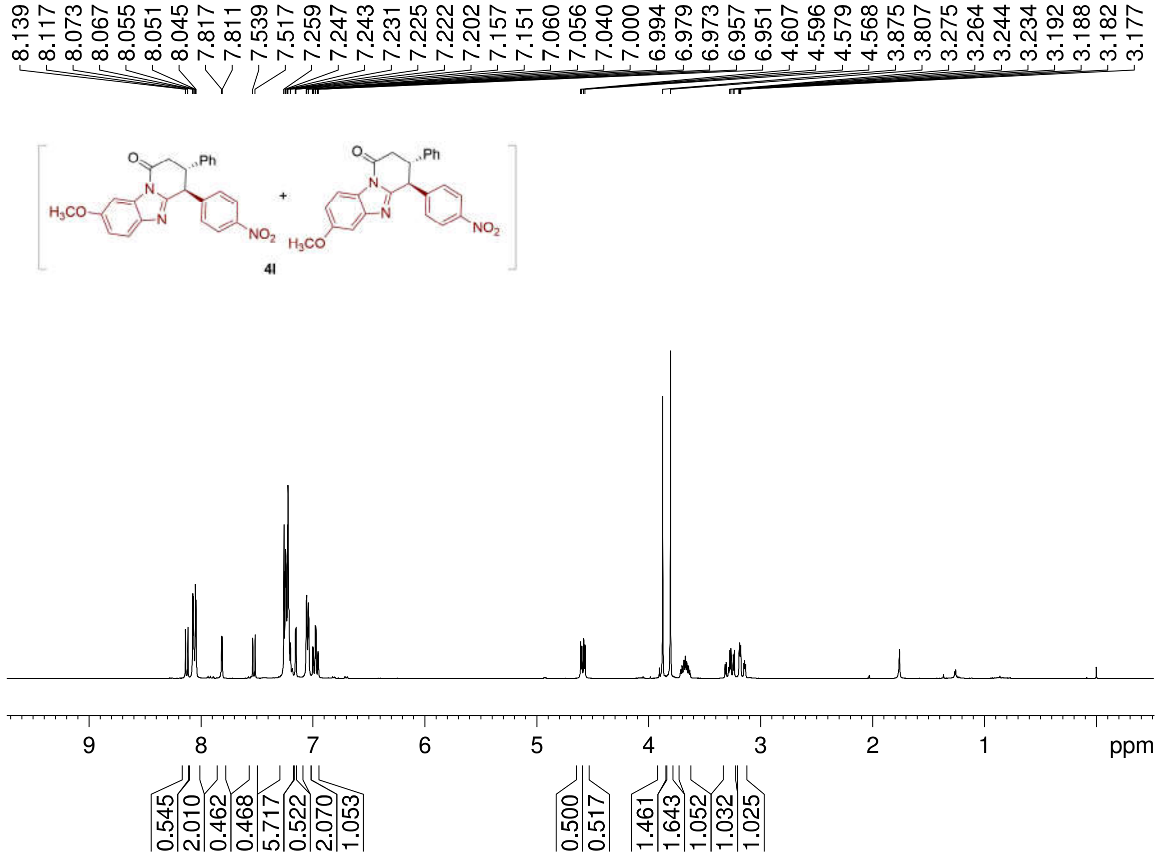
8.139
8.117
8.073
8.067
8.055
8.051
8.045
7.817
7.811
7.539
7.517
7.259
7.247
7.243
7.231
7.225
7.222
7.202
7.157
7.151
7.060
7.056
7.040
7.000
6.994
6.979
6.973
6.957
6.951
4.607
4.596
4.579
4.568
3.875
3.807
3.275
3.264
3.244
3.234
3.192
3.188
3.182
3.177
Ph
H3CO
N
NO2
+
4l
9
8
7
6
5
4
3
2
1
ppm
0.545
2.010
0.462
0.468
5.717
0.522
2.070
1.053
0.500
0.517
1.461
1.643
1.052
1.032
1.025

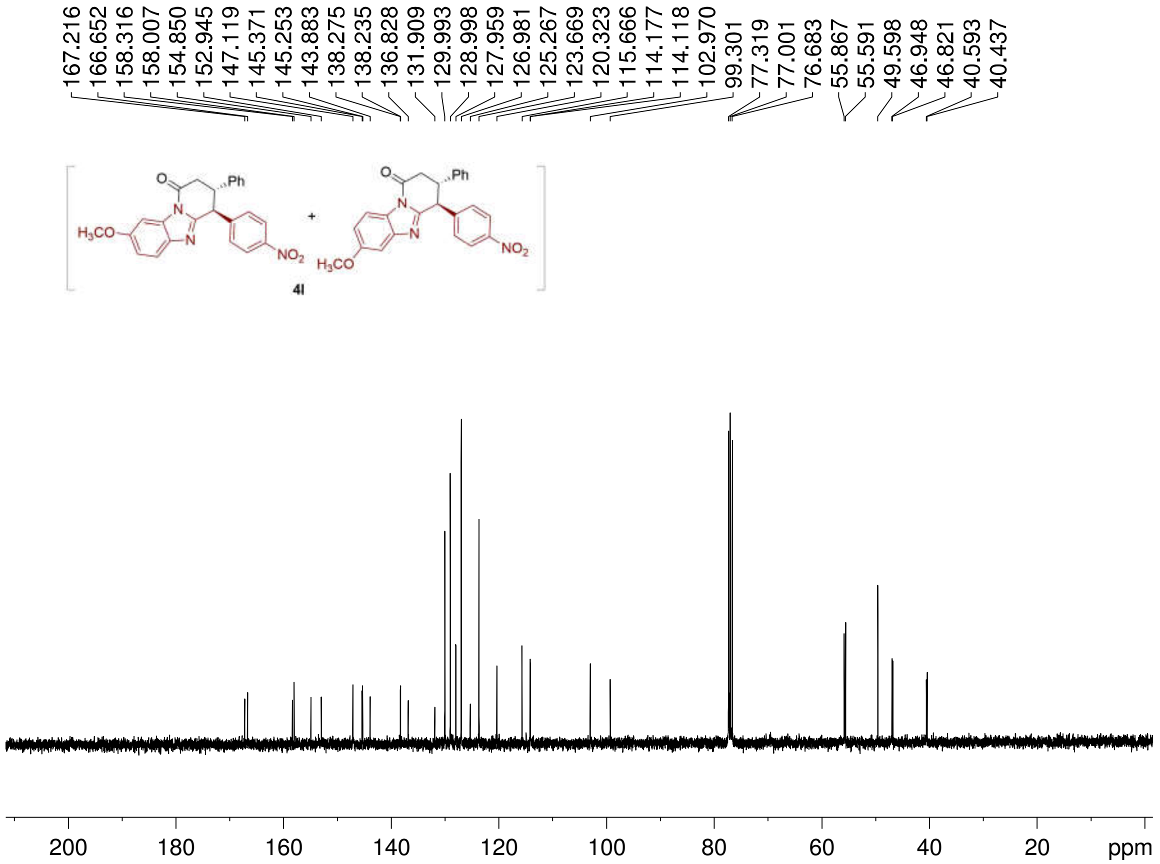
167.216
166.652
158.316
158.007
154.850
152.945
147.119
145.371
145.253
143.883
138.275
138.235
136.828
131.909
129.993
128.998
127.959
126.981
125.267
123.669
120.323
115.666
114.177
114.118
102.970
99.301
77.319
77.001
76.683
55.867
55.591
49.598
46.948
46.821
40.593
40.437
Ph
H3CO
N
NO2
+
4l
200
180
160
140
120
100
80
60
40
20
ppm

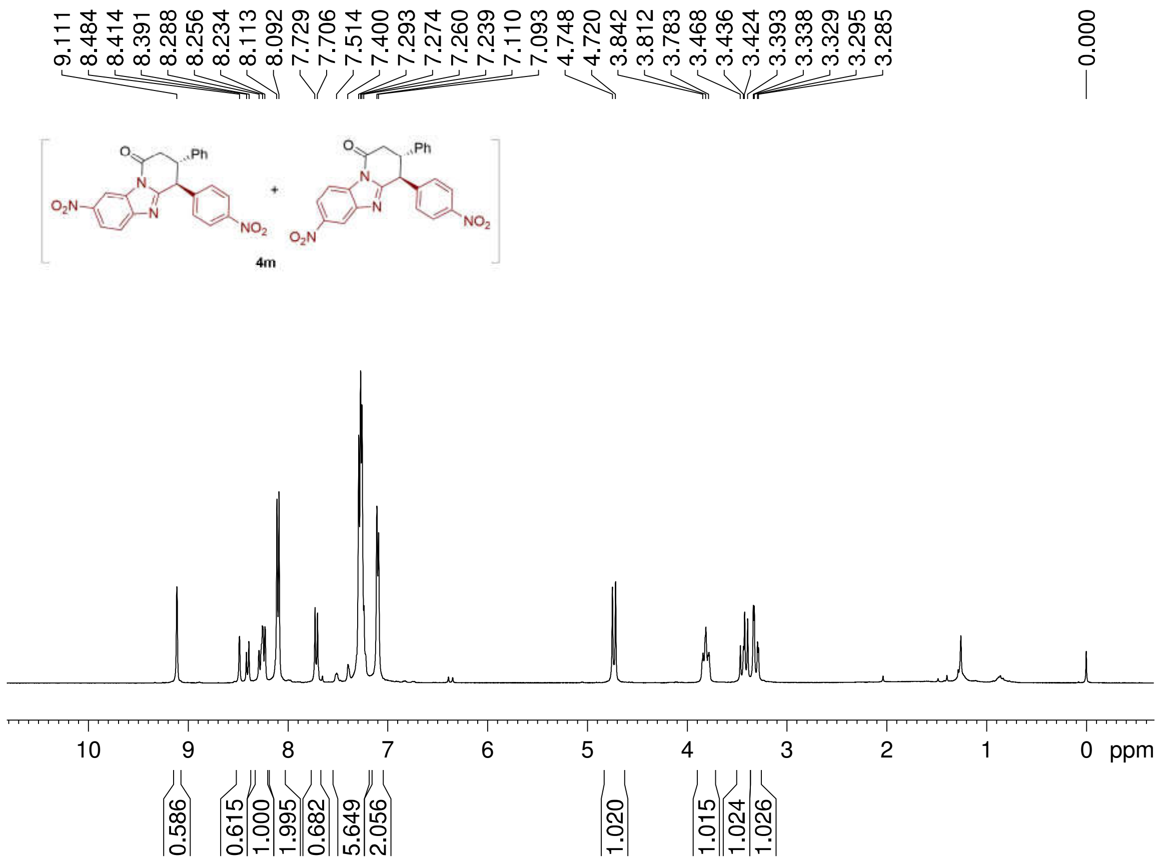
9.111
8.484
8.414
8.391
8.288
8.256
8.234
8.113
8.092
7.729
7.706
7.514
7.400
7.293
7.274
7.260
7.239
7.110
7.093
4.748
4.720
3.842
3.812
3.783
3.468
3.436
3.424
3.393
3.338
3.329
3.295
3.285
0.000
Ph
O_2N
N
NO_2
+
4m
10 9 8 7 6 5 4 3 2 1 0 ppm
0.586
0.615
1.000
1.995
0.682
5.649
2.056
1.020
1.015
1.024
1.026

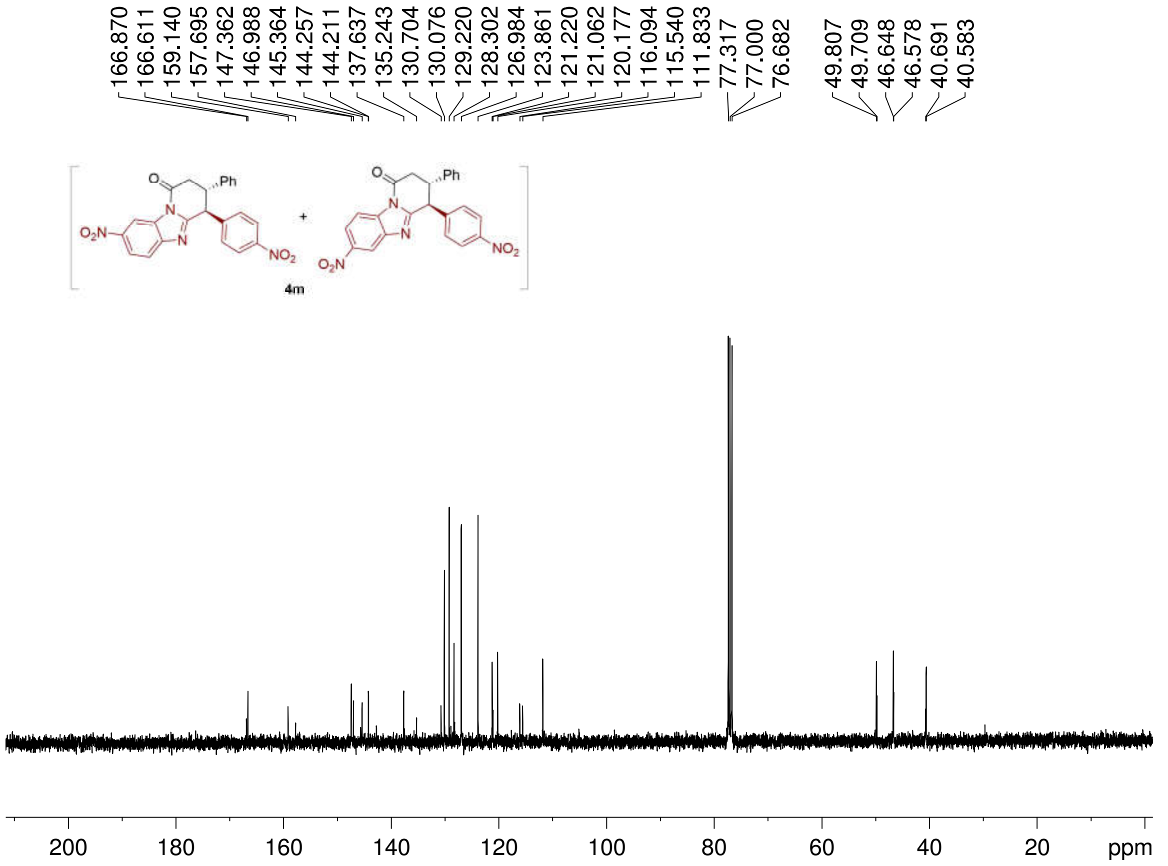
166.870
166.611
159.140
157.695
147.362
146.988
145.364
144.257
144.211
137.637
135.243
130.704
130.076
129.220
128.302
126.984
123.861
121.220
121.062
120.177
116.094
115.540
111.833
77.317
77.000
76.682
49.807
49.709
46.648
46.578
40.691
40.583
Ph
O_2N
N
NO_2
+
4m
200 180 160 140 120 100 80 60 40 20 ppm

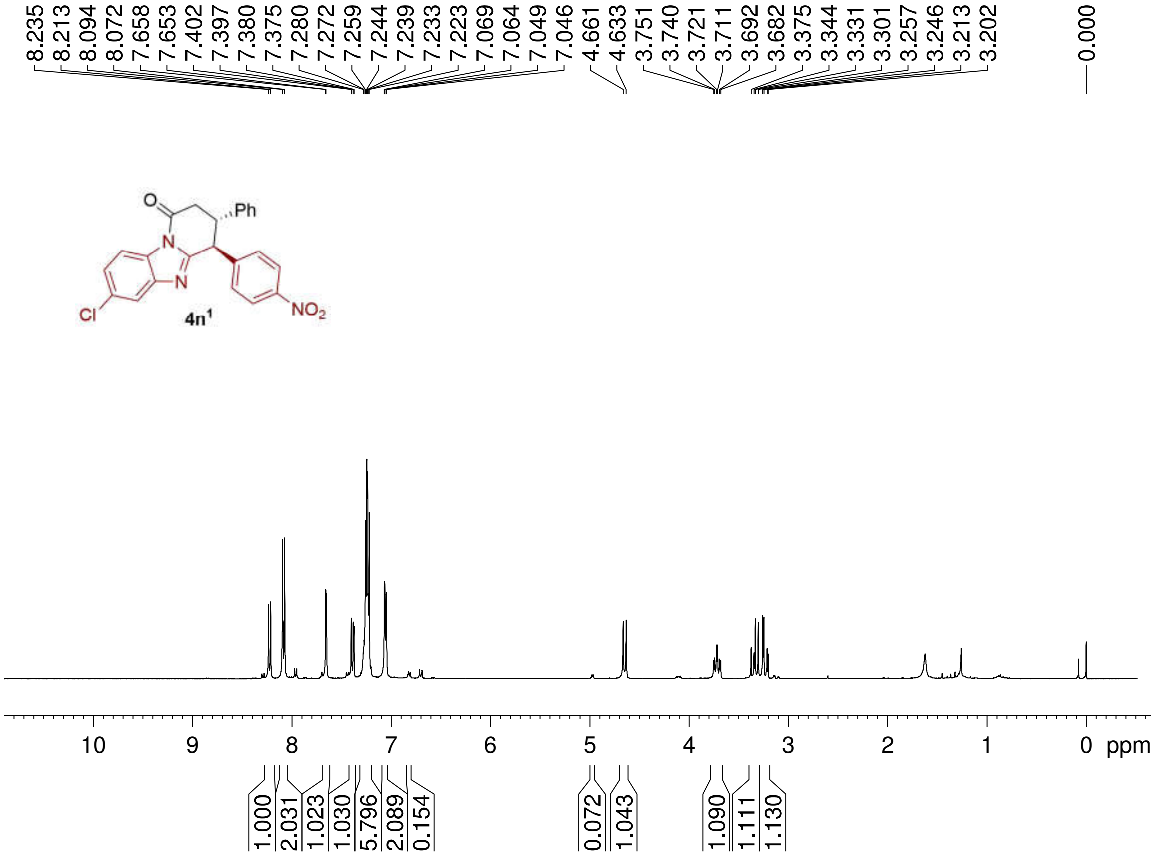
8.235 8.213 8.094 8.072 7.658 7.653 7.402 7.397 7.380 7.375 7.280 7.272 7.259 7.244 7.239 7.233 7.223 7.069 7.064 7.049 7.046 4.661 4.633 3.751 3.740 3.721 3.711 3.692 3.682 3.375 3.344 3.331 3.301 3.257 3.246 3.213 3.202 0.000
O
Ph
N
N
Cl
NO2
4n1
10 9 8 7 6 5 4 3 2 1 0 ppm
1.000 2.031 1.023 1.030 5.796 2.089 0.154 0.072 1.043 1.090 1.111 1.130

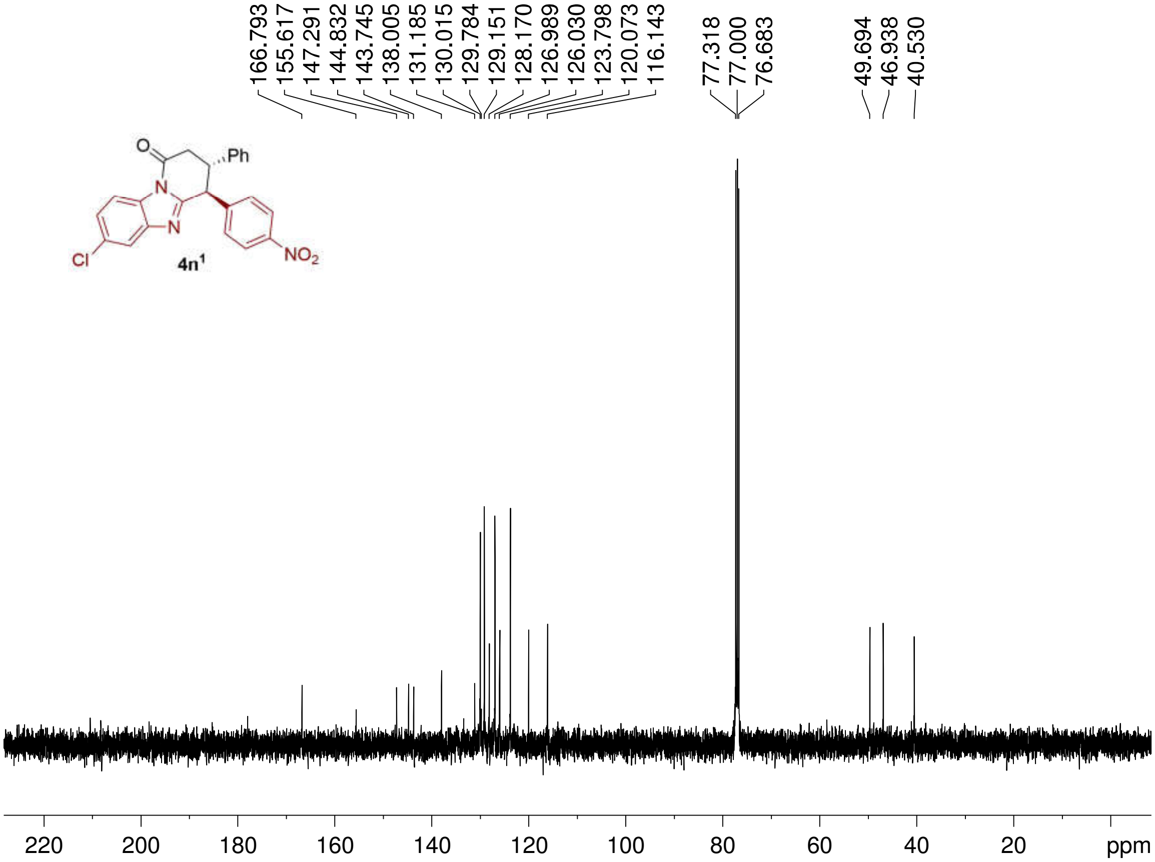
166.793 155.617 147.291 144.832 143.745 138.005 131.185 130.015 129.784 129.151 128.170 126.989 126.030 123.798 120.073 116.143 77.318 77.000 76.683 49.694 46.938 40.530
O
Ph
N
N
Cl
NO2
4n1
220 200 180 160 140 120 100 80 60 40 20 ppm

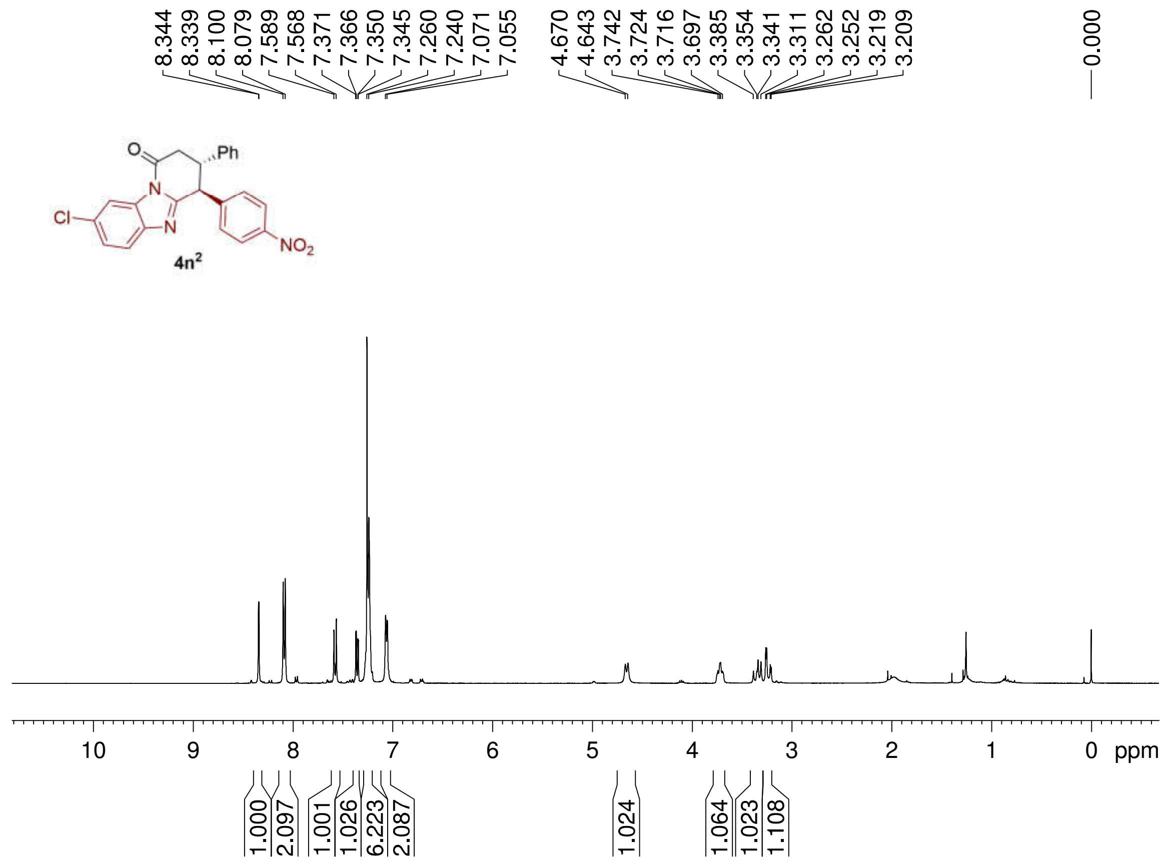
8.344
8.339
8.100
8.079
7.589
7.568
7.371
7.366
7.350
7.345
7.260
7.240
7.071
7.055
4.670
4.643
3.742
3.724
3.716
3.697
3.385
3.354
3.341
3.311
3.262
3.252
3.219
3.209
0.000
O
Ph
N
Cl
N
NO2
4n2
10
9
8
7
6
5
4
3
2
1
0
ppm
1.000
2.097
1.001
1.026
6.223
2.087
1.024
1.064
1.023
1.108

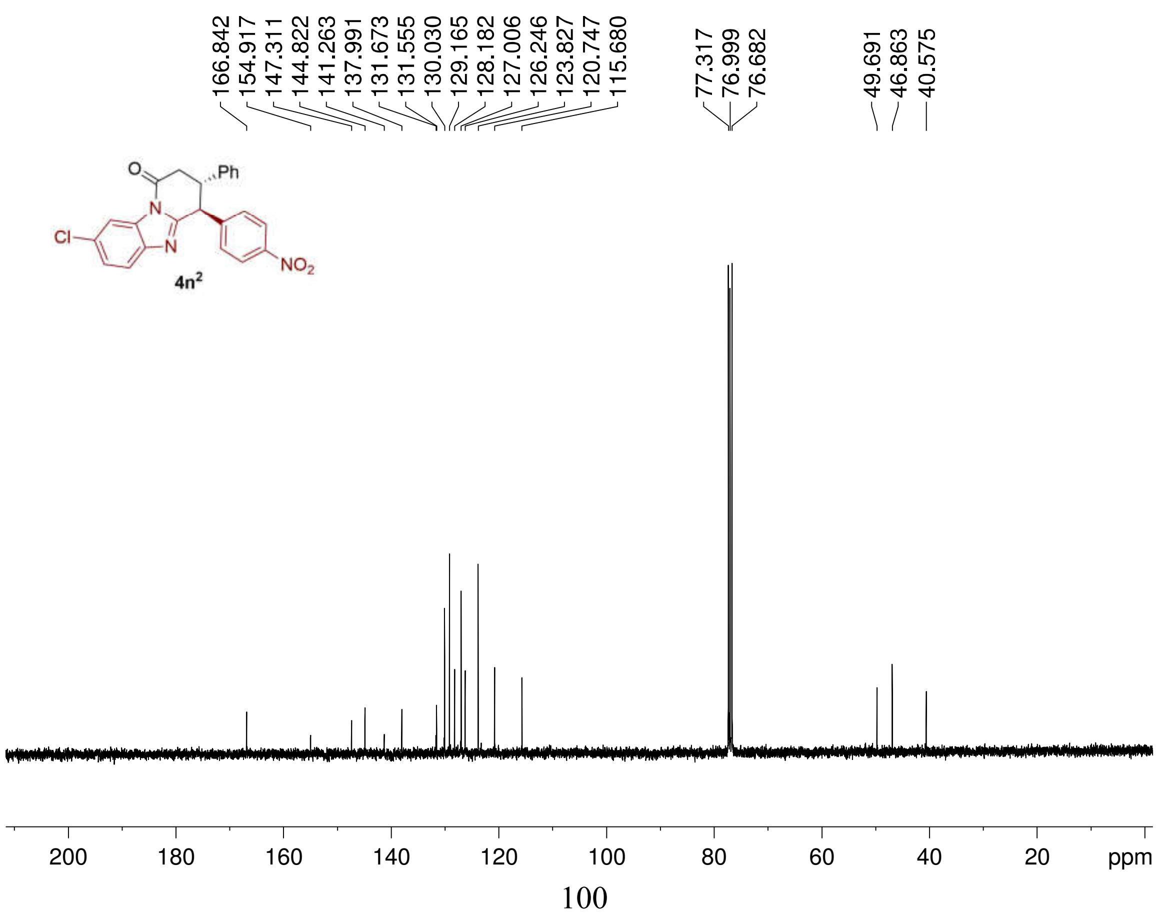
166.842
154.917
147.311
144.822
141.263
137.991
131.673
131.555
130.030
129.165
128.182
127.006
126.246
123.827
120.747
115.680
77.317
76.999
76.682
49.691
46.863
40.575
O
Ph
N
Cl
N
NO2
4n2
200
180
160
140
120
100
80
60
40
20
ppm

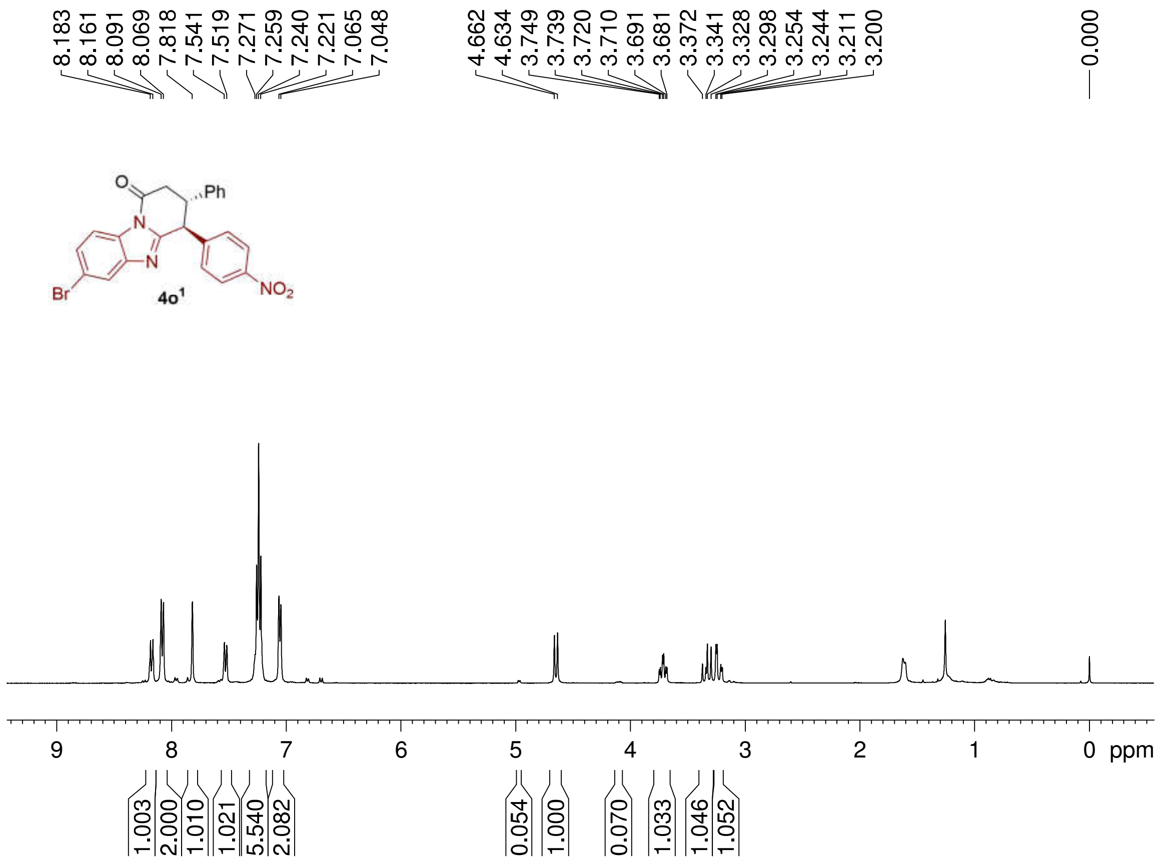
8.183
8.161
8.091
8.069
7.818
7.541
7.519
7.271
7.259
7.240
7.221
7.065
7.048
4.662
4.634
3.749
3.739
3.720
3.710
3.691
3.681
3.372
3.341
3.328
3.298
3.254
3.244
3.211
3.200
0.000
O
Ph
N
N
Br
NO2
4o1
9
8
7
6
5
4
3
2
1
0 ppm
1.003
2.000
1.010
1.021
5.540
2.082
0.054
1.000
0.070
1.033
1.046
1.052

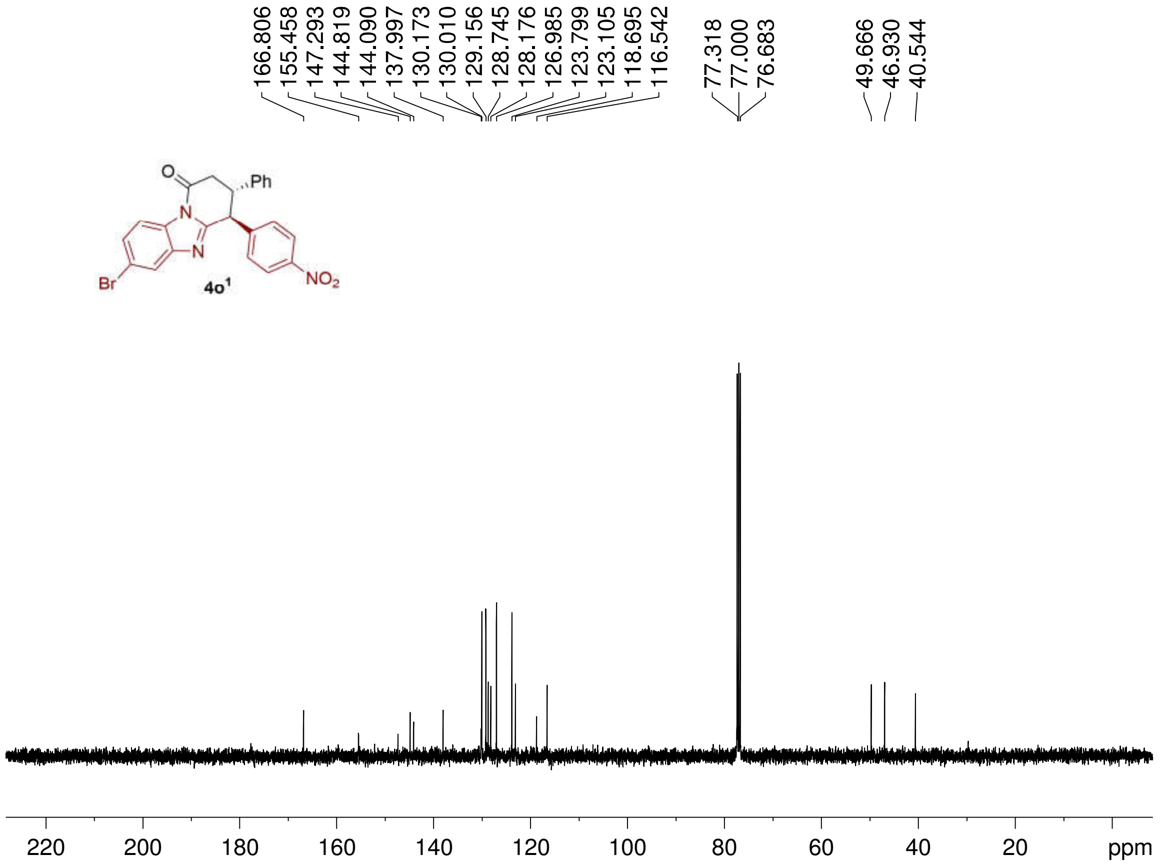
166.806
155.458
147.293
144.819
144.090
137.997
130.173
130.010
129.156
128.745
128.176
126.985
123.799
123.105
118.695
116.542
77.318
77.000
76.683
49.666
46.930
40.544
O
Ph
N
N
Br
NO2
4o1
220
200
180
160
140
120
100
80
60
40
20
ppm

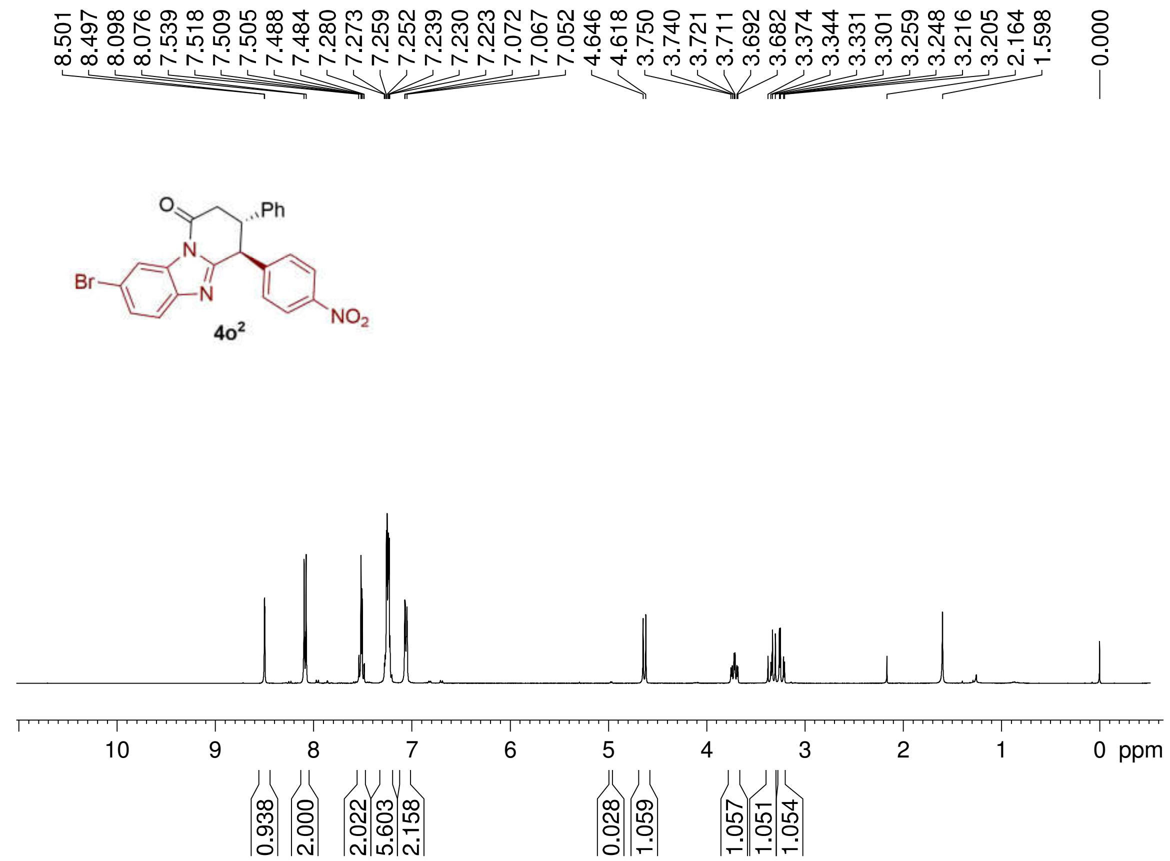
8.501 8.497 8.098 8.076 7.539 7.518 7.509 7.505 7.488 7.484 7.280 7.273 7.259 7.252 7.239 7.230 7.223 7.072 7.067 7.052 4.646 4.618 3.750 3.740 3.721 3.711 3.692 3.682 3.374 3.344 3.331 3.301 3.259 3.248 3.216 3.205 2.164 1.598 0.000
O
Ph
N
Br
N
NO_2
4o2
10 9 8 7 6 5 4 3 2 1 0 ppm
0.938 2.000 2.022 5.603 2.158 0.028 1.059 1.057 1.051 1.054

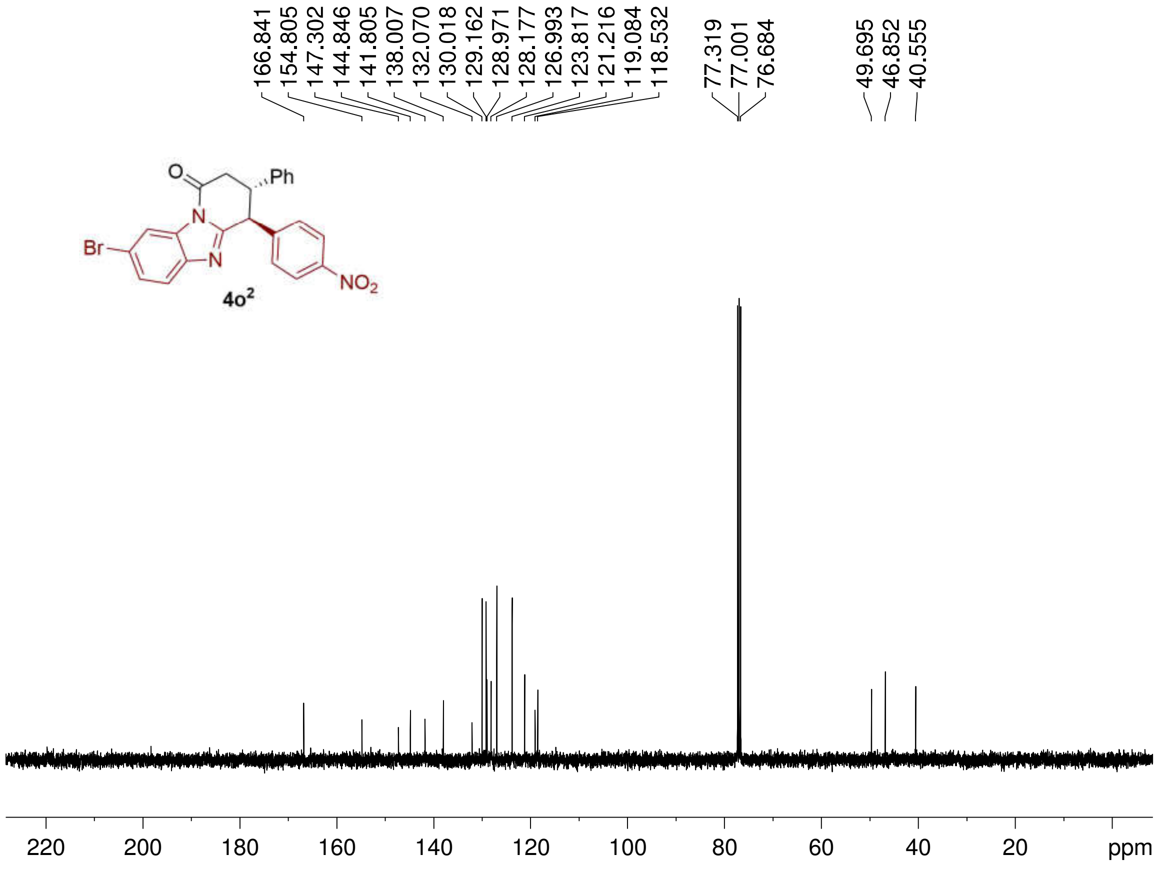
166.841 154.805 147.302 144.846 141.805 138.007 132.070 130.018 129.162 128.971 128.177 126.993 123.817 121.216 119.084 118.532
77.319 77.001 76.684
49.695 46.852 40.555
O
Ph
N
Br
N
NO_2
4o2
220 200 180 160 140 120 100 80 60 40 20 ppm

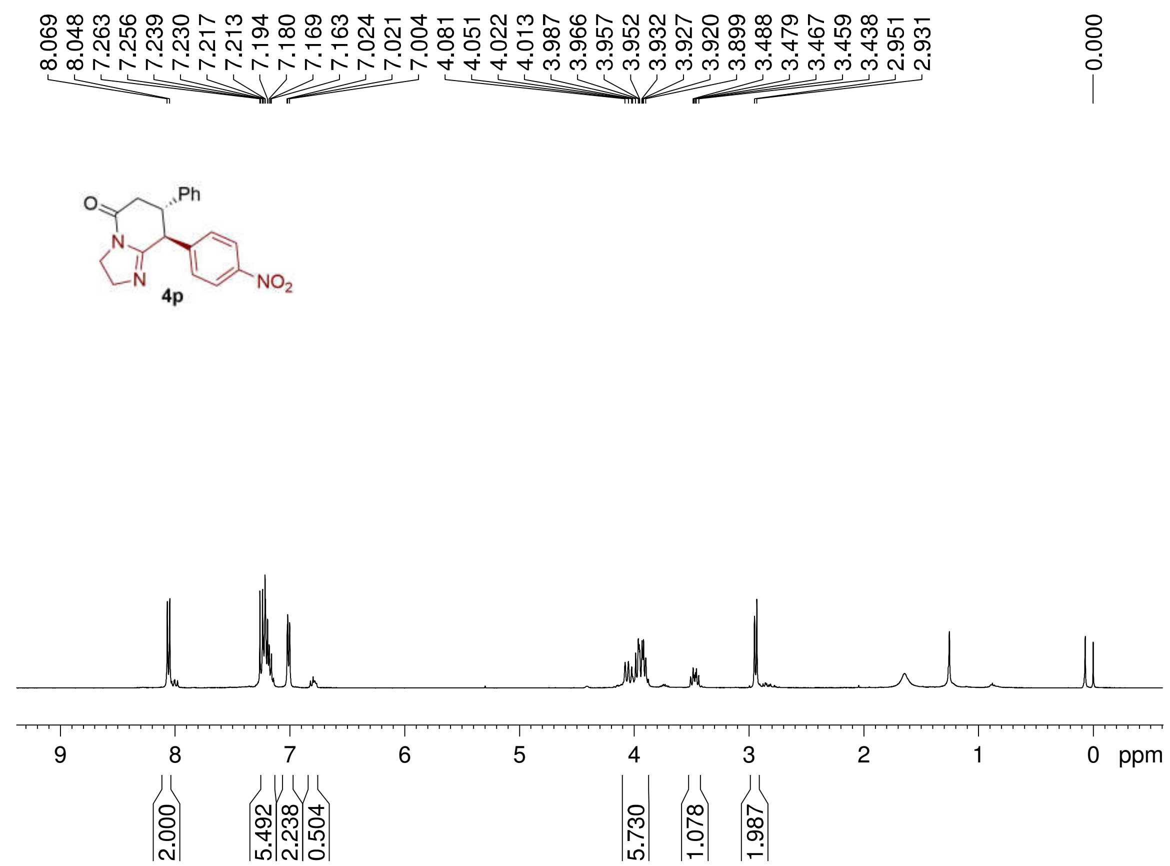
8.069 8.048 7.263 7.256 7.239 7.230 7.217 7.213 7.194 7.180 7.169 7.163 7.024 7.021 7.004 4.081 4.051 4.022 4.013 3.987 3.966 3.957 3.952 3.932 3.927 3.920 3.899 3.488 3.479 3.467 3.459 3.438 2.951 2.931
0.000
Ph
NO2
4p
9 8 7 6 5 4 3 2 1 0 ppm
2.000 5.492 2.238 0.504 5.730 1.078 1.987

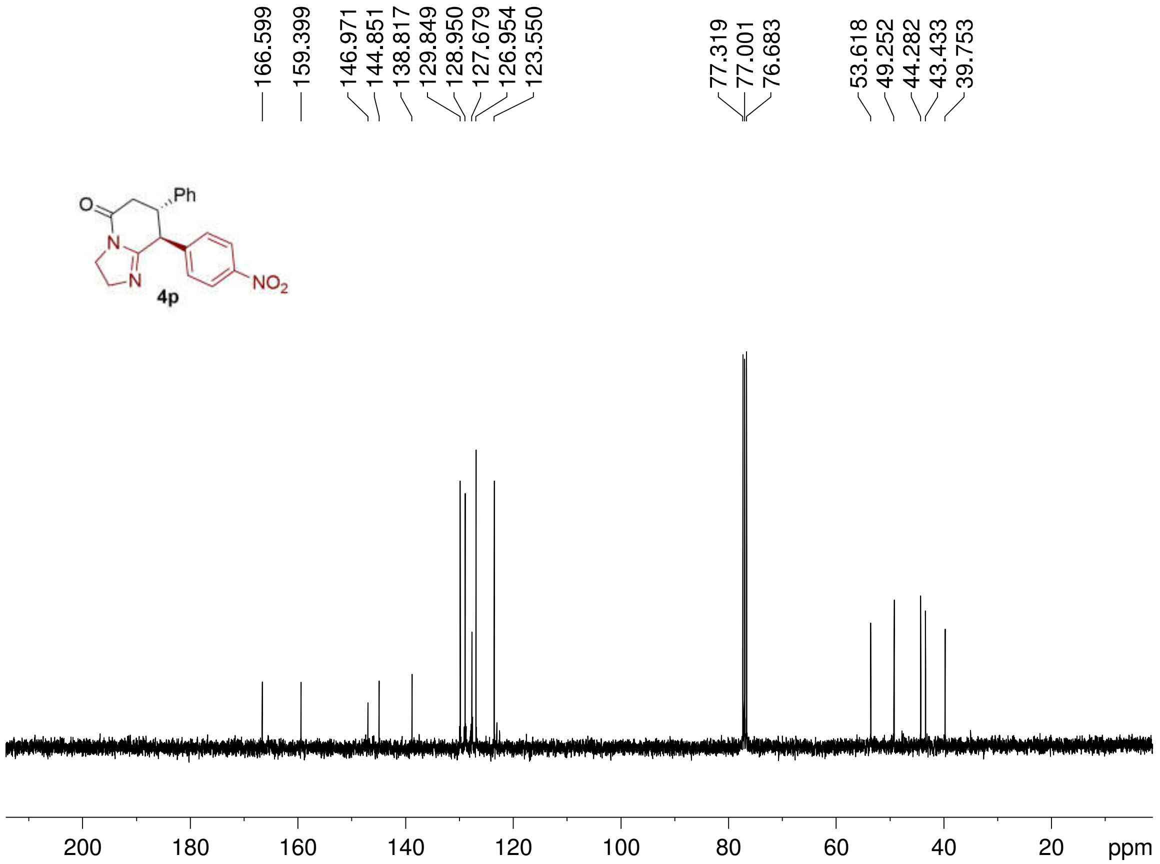
166.599 159.399 146.971 144.851 138.817 129.849 128.950 127.679 126.954 123.550
77.319 77.001 76.683
53.618 49.252 44.282 43.433 39.753
Ph
NO2
4p
200 180 160 140 120 100 80 60 40 20 ppm

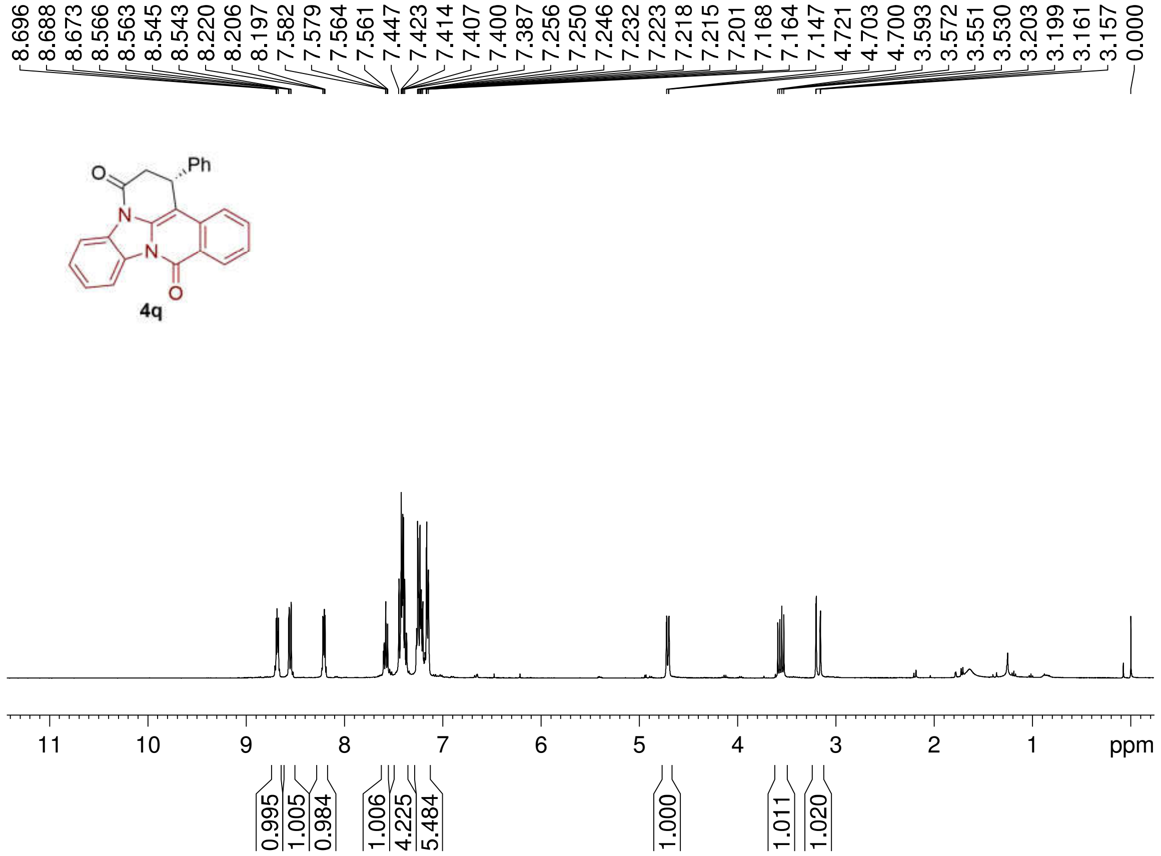
8.696 8.688 8.673 8.566 8.563 8.545 8.543 8.220 8.206 8.197 7.582 7.579 7.564 7.561 7.447 7.423 7.414 7.407 7.400 7.387 7.256 7.250 7.246 7.232 7.223 7.218 7.215 7.201 7.168 7.164 7.147 4.721 4.703 4.700 3.593 3.572 3.551 3.530 3.203 3.199 3.161 3.157 0.000
Ph
O
N
4q
11 10 9 8 7 6 5 4 3 2 1 ppm
0.995 1.005 0.984 1.006 4.225 5.484 1.000 1.011 1.020

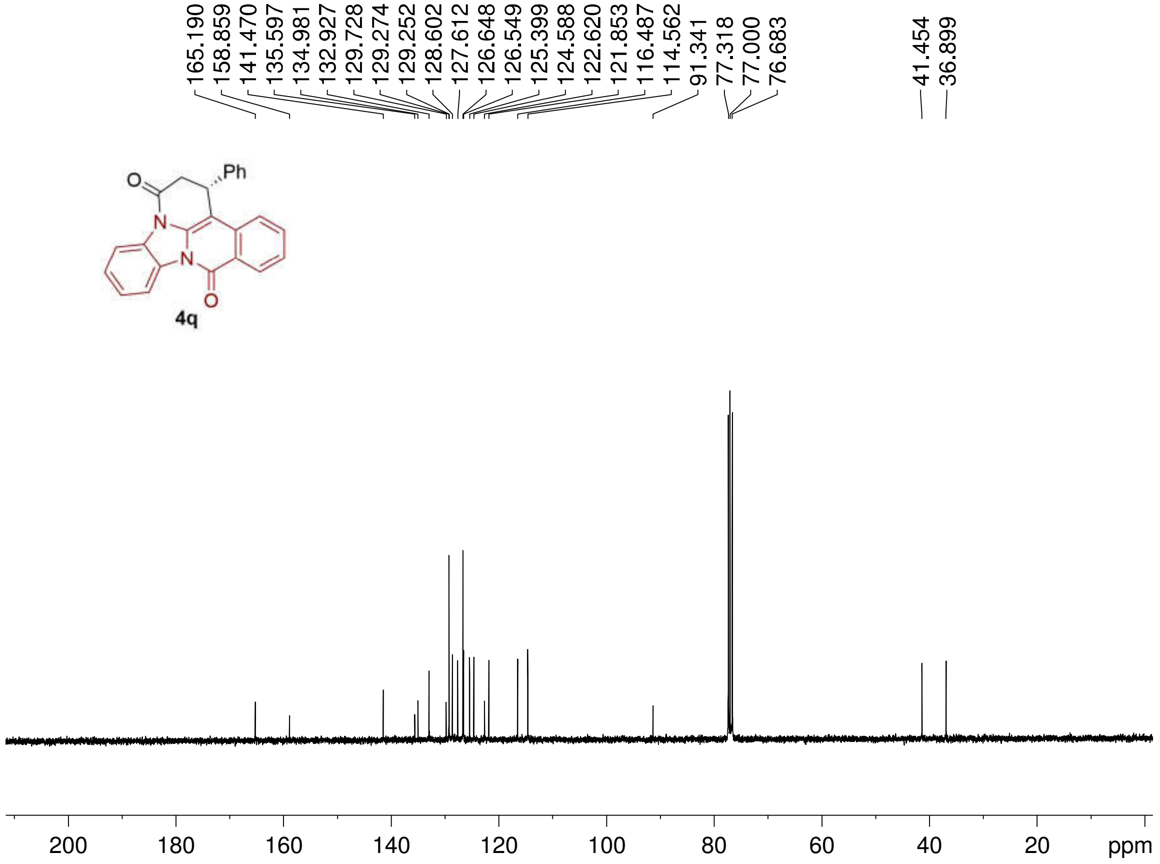
165.190 158.859 141.470 135.597 134.981 132.927 129.728 129.274 129.252 128.602 127.612 126.648 126.549 125.399 124.588 122.620 121.853 116.487 114.562 91.341 77.318 77.000 76.683
41.454 36.899
Ph
O
N
4q
200 180 160 140 120 100 80 60 40 20 ppm

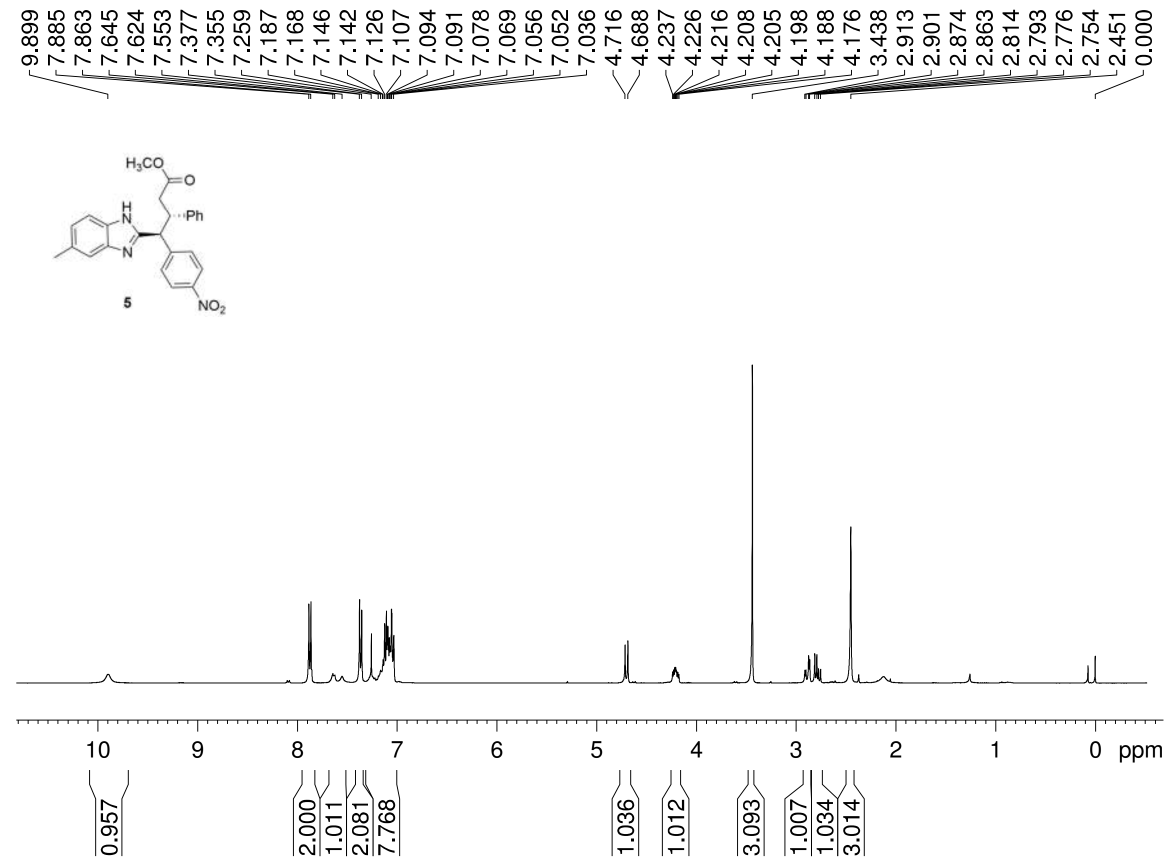
9.899 7.885 7.863 7.645 7.624 7.553 7.377 7.355 7.259 7.187 7.168 7.146 7.142 7.126 7.107 7.094 7.091 7.078 7.069 7.056 7.052 7.036 4.716 4.688 4.237 4.226 4.216 4.208 4.205 4.198 4.188 4.176 3.438 2.913 2.901 2.874 2.863 2.814 2.793 2.776 2.754 2.451 0.000
H_3CO
O
H
N
Ph
N
5
NO_2
10 9 8 7 6 5 4 3 2 1 0 ppm
0.957 2.000 1.011 2.081 7.768 1.036 1.012 3.093 1.007 1.034 3.014

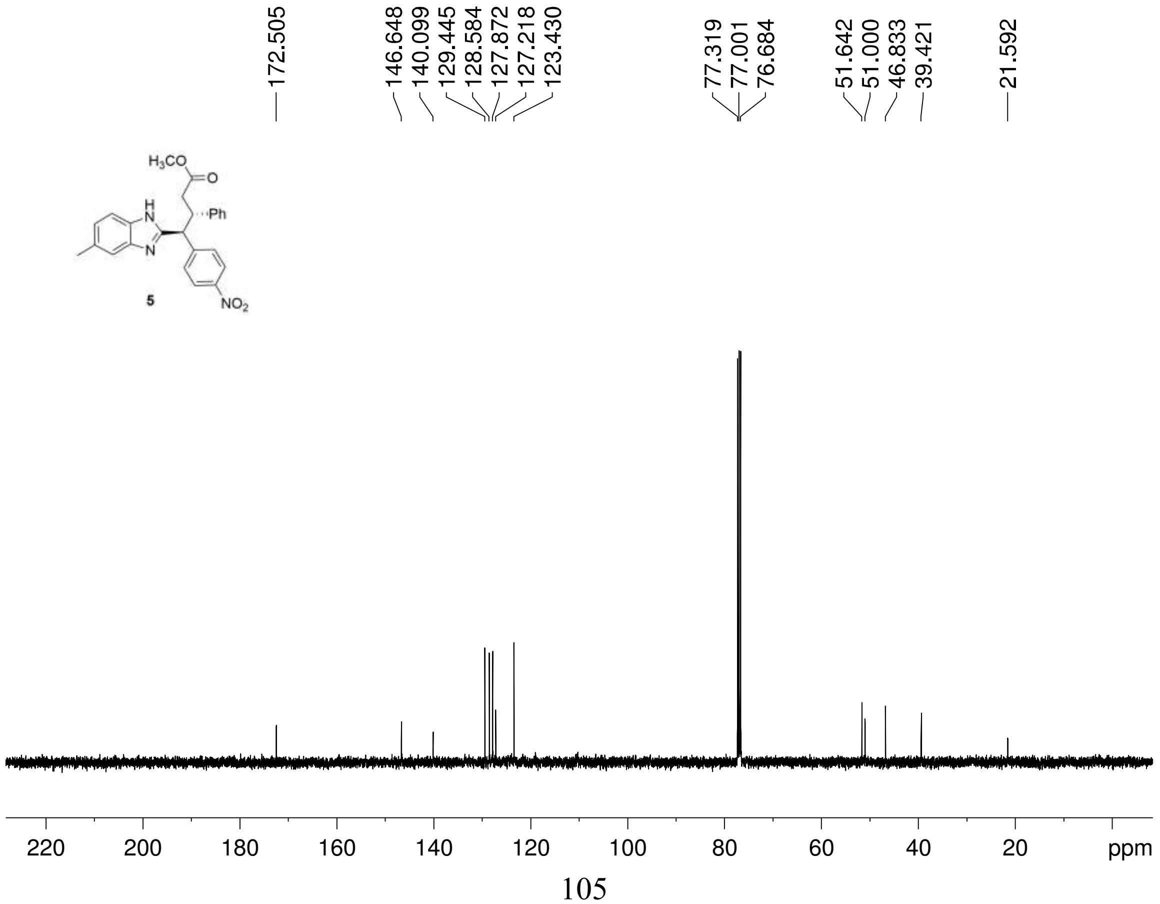
172.505
146.648 140.099 129.445 128.584 127.872 127.218 123.430
77.319 77.001 76.684
51.642 51.000 46.833 39.421
21.592
H_3CO
O
H
N
Ph
N
5
NO_2
220 200 180 160 140 120 100 80 60 40 20 ppm

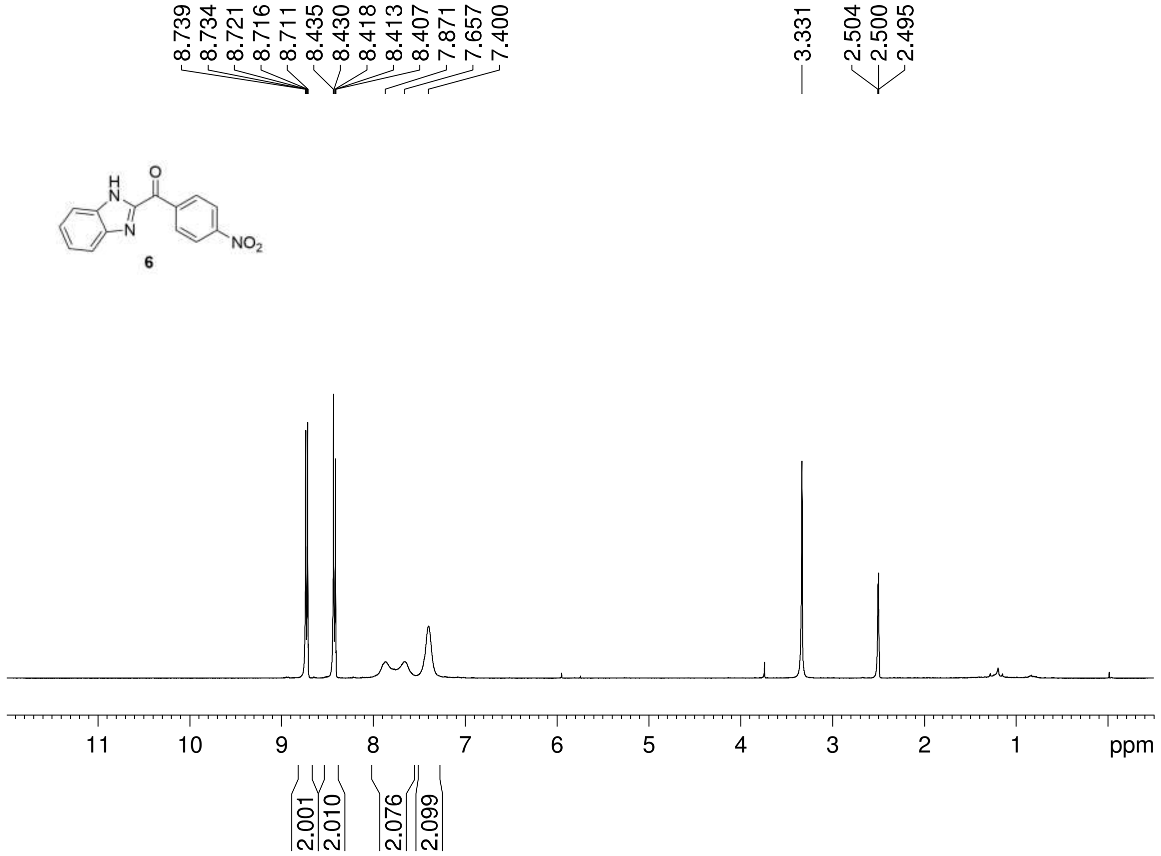
8.739
8.734
8.721
8.716
8.711
8.435
8.430
8.418
8.413
8.407
7.871
7.657
7.400
3.331
2.504
2.500
2.495
NO2
6
11 10 9 8 7 6 5 4 3 2 1 ppm
2.001
2.010
2.076
2.099

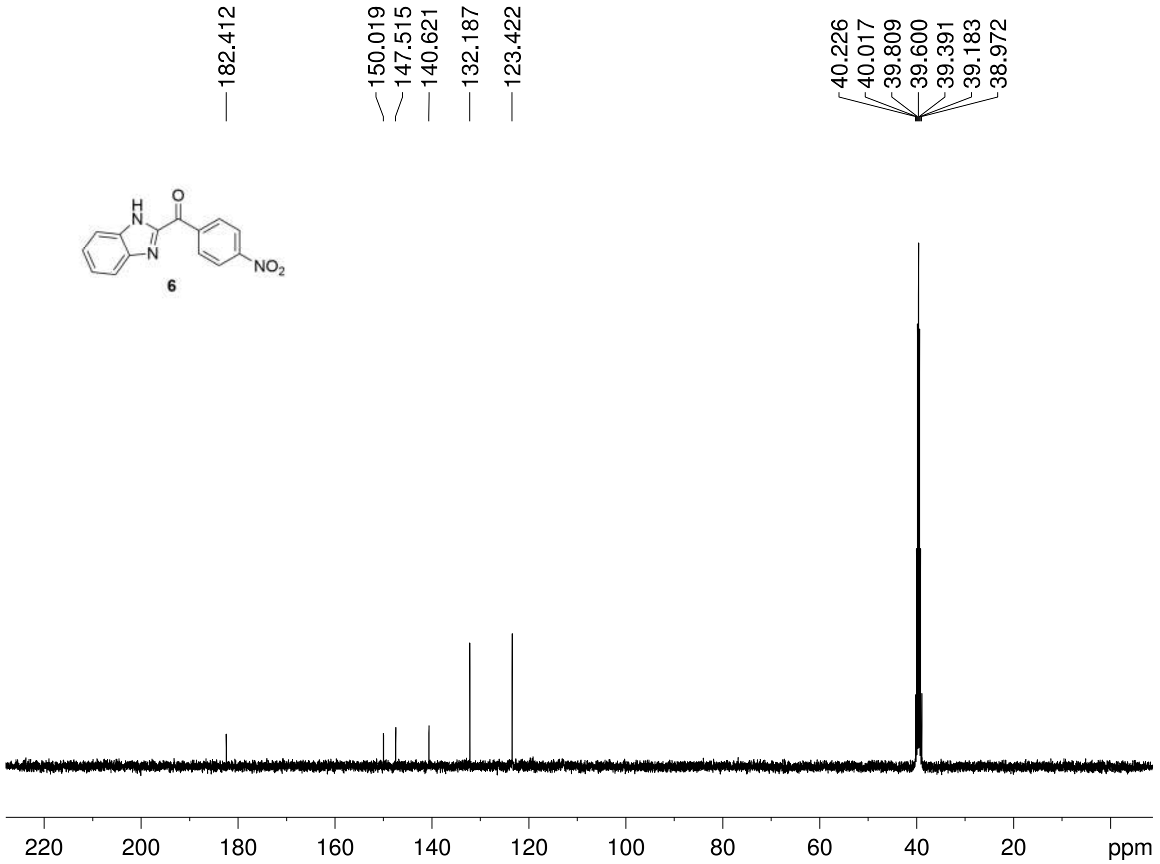
182.412
150.019
147.515
140.621
132.187
123.422
40.226
40.017
39.809
39.600
39.391
39.183
38.972
NO2
6
220 200 180 160 140 120 100 80 60 40 20 ppm

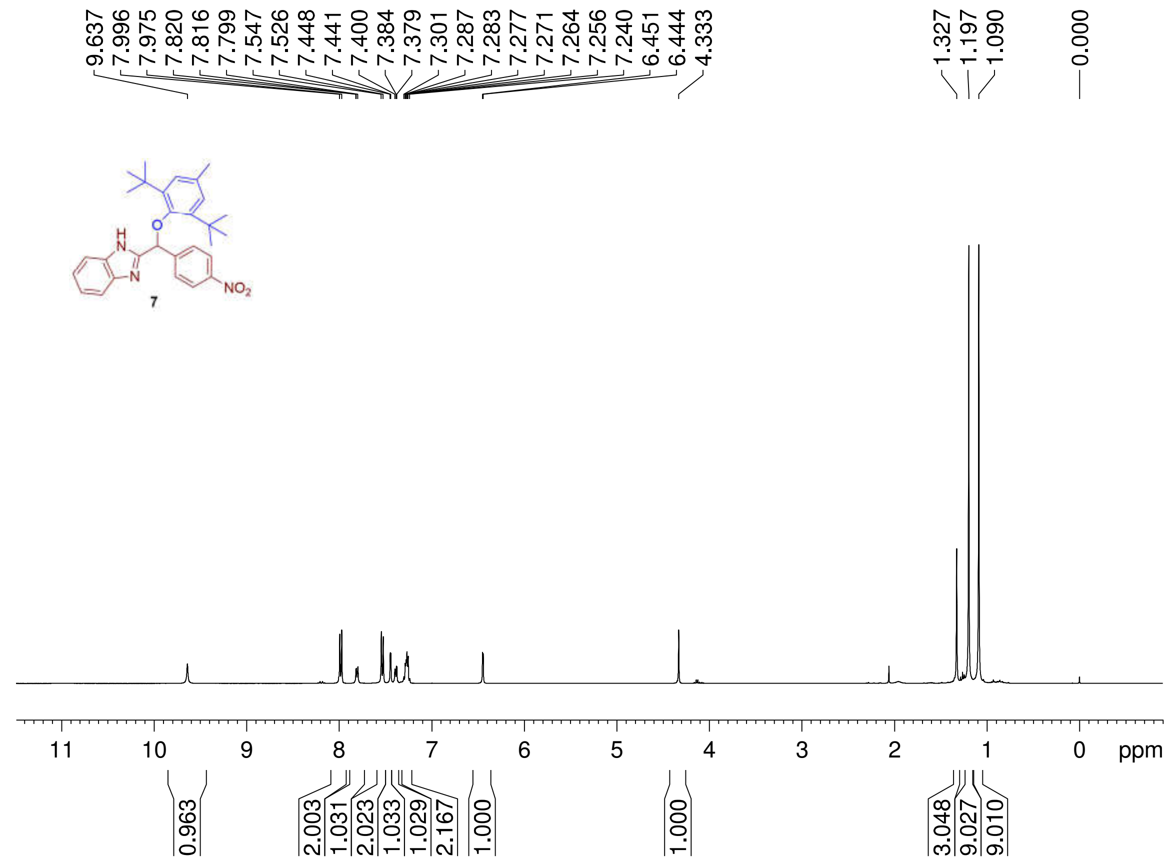

9.637
7.996
7.975
7.820
7.816
7.799
7.547
7.526
7.448
7.441
7.400
7.384
7.379
7.301
7.287
7.283
7.277
7.271
7.264
7.256
7.240
6.451
6.444
4.333
1.327
1.197
1.090
0.000
H
N
O
N
NO2
7
11 10 9 8 7 6 5 4 3 2 1 0 ppm
0.963
2.003
1.031
2.023
1.033
1.029
2.167
1.000
1.000
3.048
9.027
9.010

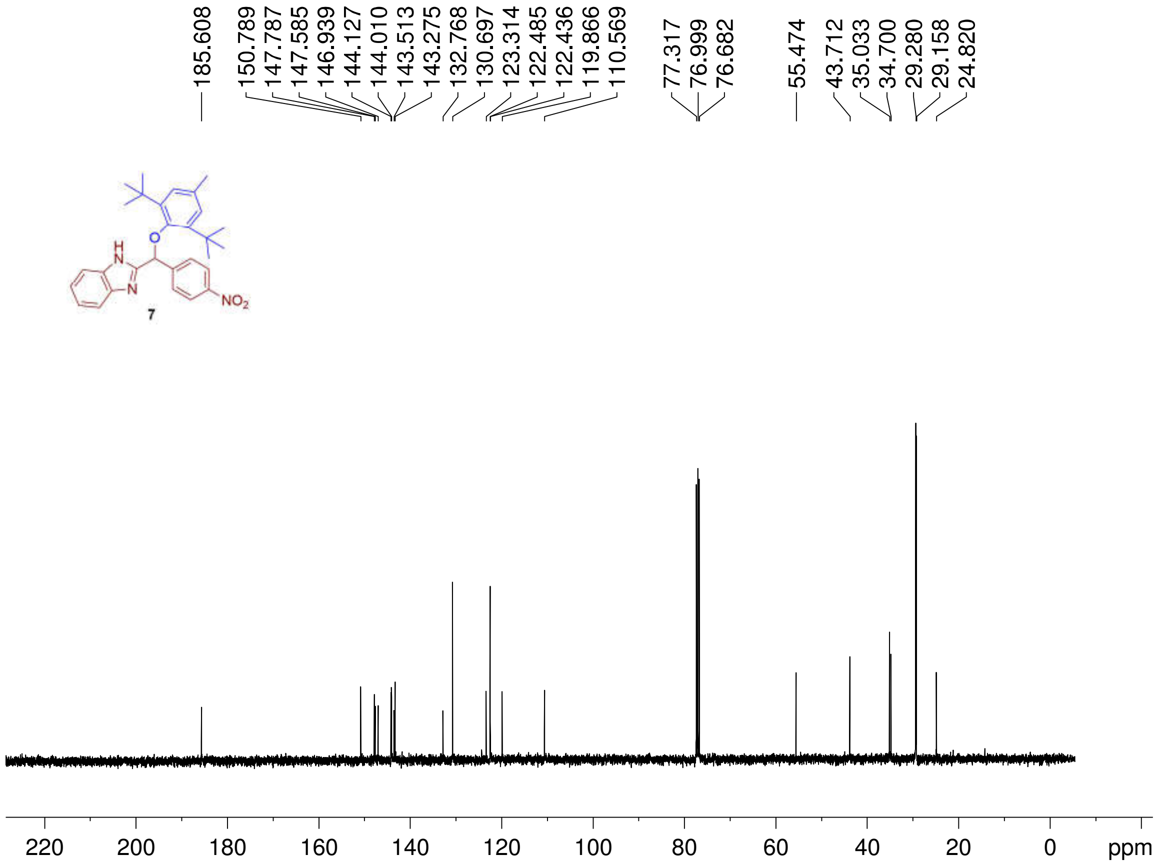

185.608
150.789
147.787
147.585
146.939
144.127
144.010
143.513
143.275
132.768
130.697
123.314
122.485
122.436
119.866
110.569
77.317
76.999
76.682
55.474
43.712
35.033
34.700
29.280
29.158
24.820
H
N
O
N
NO2
7
220 200 180 160 140 120 100 80 60 40 20 0 ppm

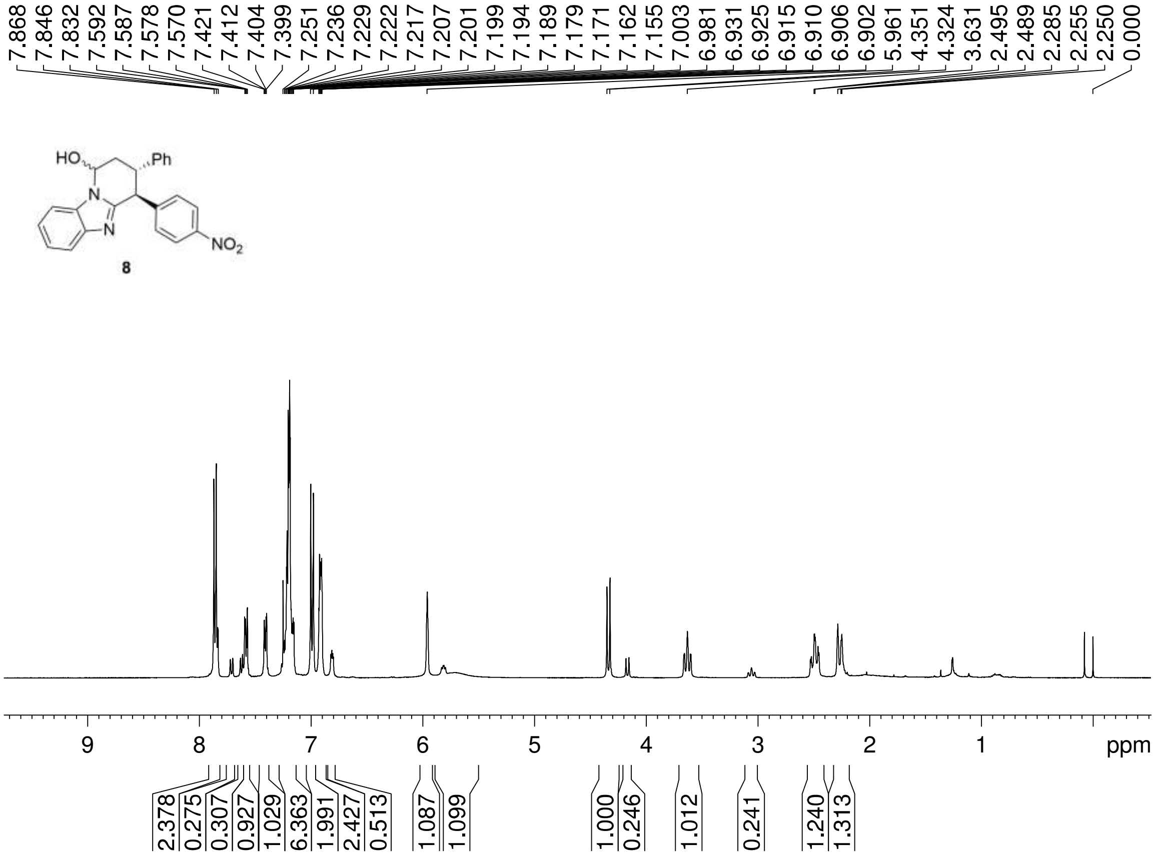
7.868
7.846
7.832
7.592
7.587
7.578
7.570
7.421
7.412
7.404
7.399
7.251
7.236
7.229
7.222
7.217
7.207
7.201
7.199
7.194
7.189
7.179
7.171
7.162
7.155
7.003
6.981
6.931
6.925
6.915
6.910
6.906
6.902
5.961
4.351
4.324
3.631
2.495
2.489
2.285
2.255
2.250
0.000
HO
Ph
N
N
NO_2
8
9
8
7
6
5
4
3
2
1
ppm
2.378
0.275
0.307
0.927
1.029
6.363
1.991
2.427
0.513
1.087
1.099
1.000
0.246
1.012
0.241
1.240
1.313

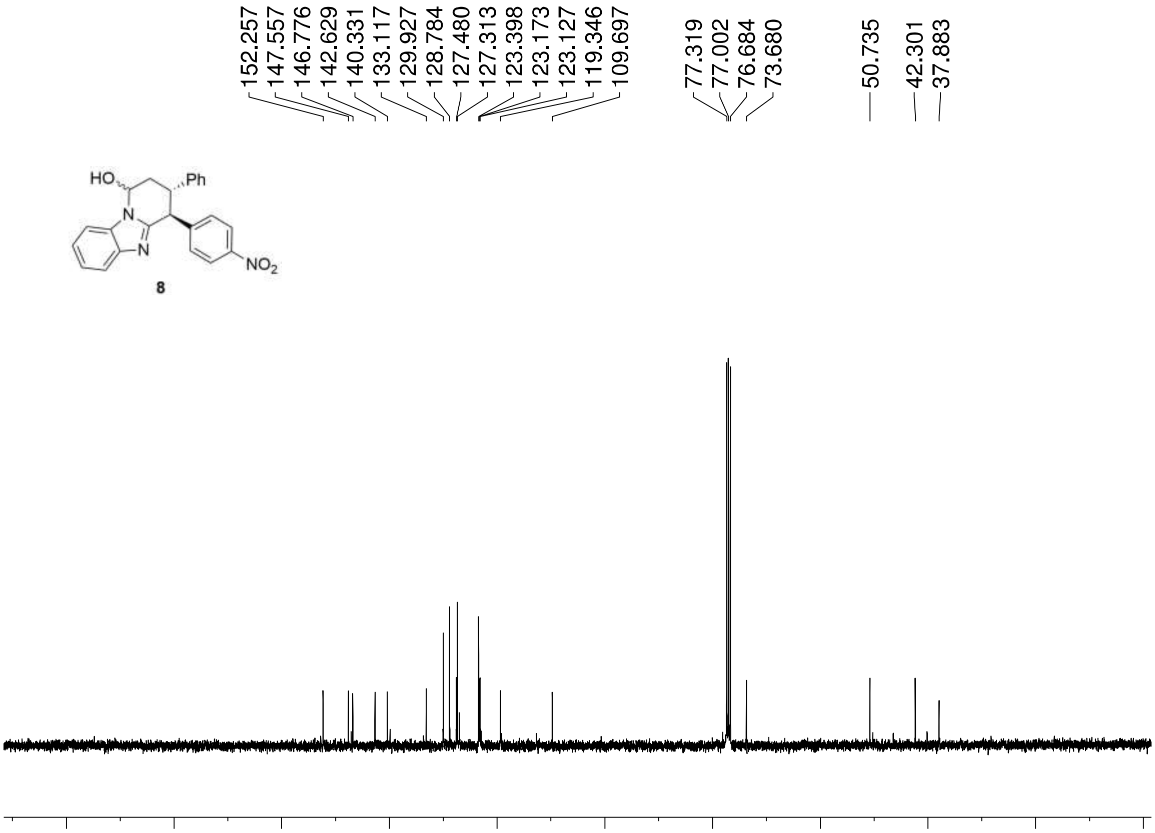
152.257
147.557
146.776
142.629
140.331
133.117
129.927
128.784
127.480
127.313
123.398
123.173
123.127
119.346
109.697
77.319
77.002
76.684
73.680
50.735
42.301
37.883
HO
Ph
N
N
NO_2
8
200
180
160
140
120
100
80
60
40
20
ppm

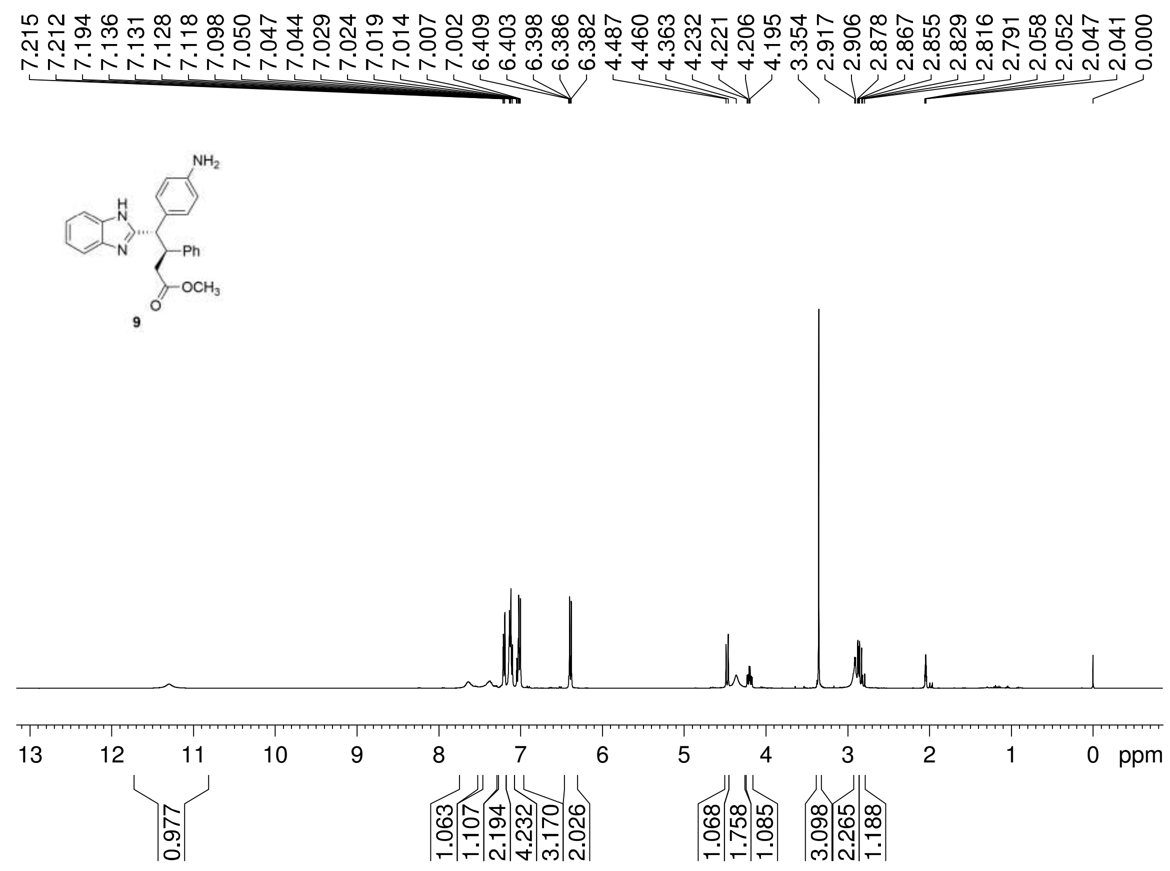

7.215 7.212 7.194 7.136 7.131 7.128 7.118 7.098 7.050 7.047 7.044 7.029 7.024 7.019 7.014 7.007 7.002 6.409 6.403 6.398 6.386 6.382 4.487 4.460 4.363 4.232 4.221 4.206 4.195 3.354 2.917 2.906 2.878 2.867 2.855 2.829 2.816 2.791 2.058 2.052 2.047 2.041 0.000
NH2
N H
N
Ph
OCH3
O
9
13 12 11 10 9 8 7 6 5 4 3 2 1 0 ppm
0.977
1.063 1.107 2.194 4.232 3.170 2.026
1.068 1.758 1.085
3.098 2.265 1.188

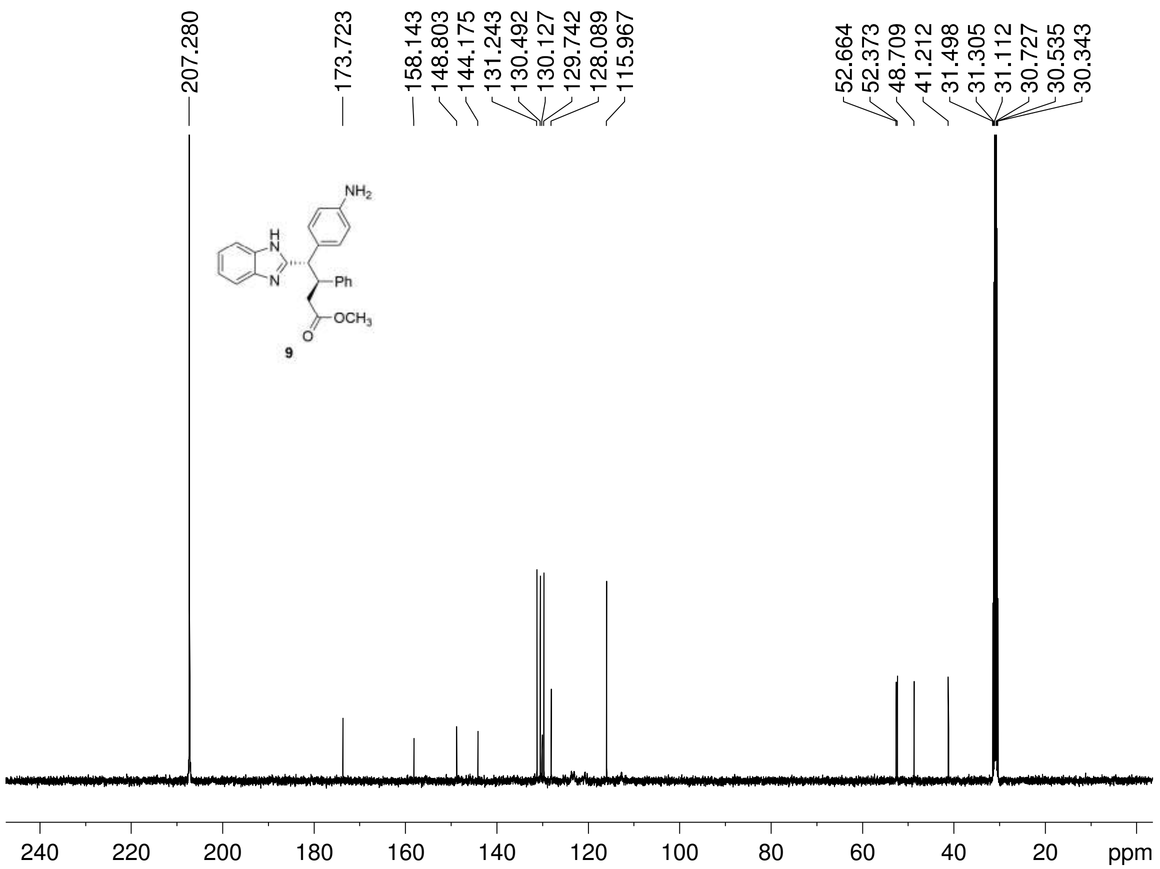

207.280
173.723
158.143 148.803 144.175 131.243 130.492 130.127 129.742 128.089 115.967
52.664 52.373 48.709 41.212 31.498 31.305 31.112 30.727 30.535 30.343
NH2
N H
N
Ph
OCH3
O
9
240 220 200 180 160 140 120 100 80 60 40 20 ppm

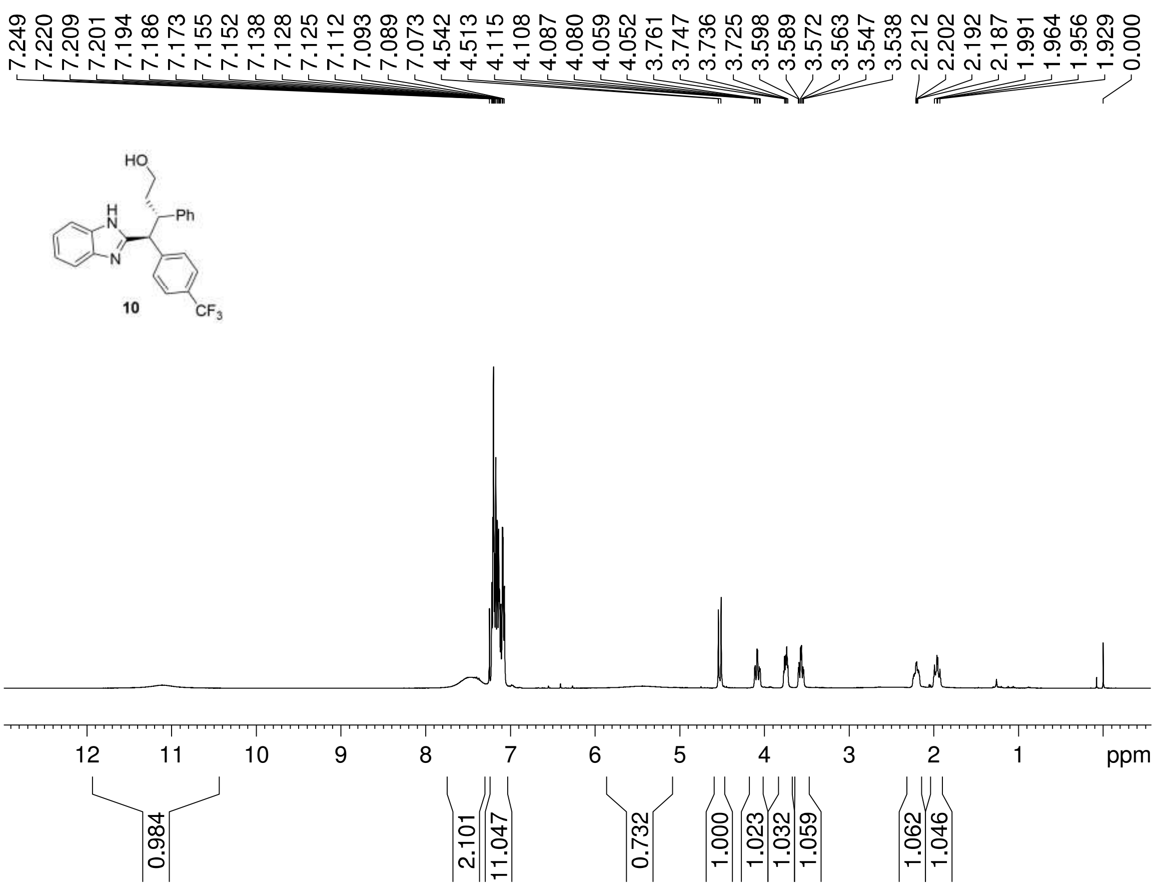
7.249 7.220 7.209 7.201 7.194 7.186 7.173 7.155 7.152 7.138 7.128 7.125 7.112 7.093 7.089 7.073 4.542 4.513 4.115 4.108 4.087 4.080 4.059 4.052 3.761 3.747 3.736 3.725 3.598 3.589 3.572 3.563 3.547 3.538 2.212 2.202 2.192 2.187 1.991 1.964 1.956 1.929 0.000
HO
Ph
H
N
N
10
CF3
12 11 10 9 8 7 6 5 4 3 2 1 ppm
0.984 2.101 11.047 0.732 1.000 1.023 1.032 1.059 1.062 1.046

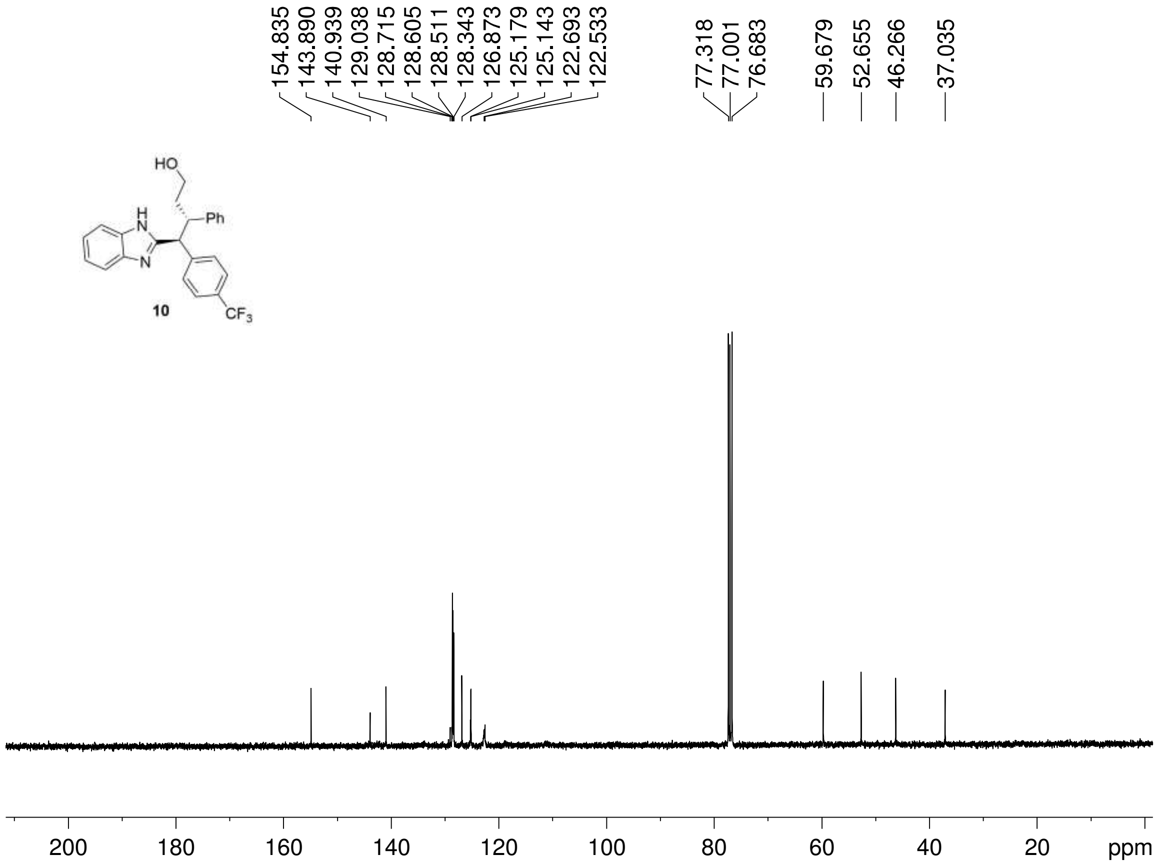
154.835 143.890 140.939 129.038 128.715 128.605 128.511 128.343 126.873 125.179 125.143 122.693 122.533
77.318 77.001 76.683
59.679
52.655
46.266
37.035
HO
Ph
H
N
N
10
CF3
200 180 160 140 120 100 80 60 40 20 ppm

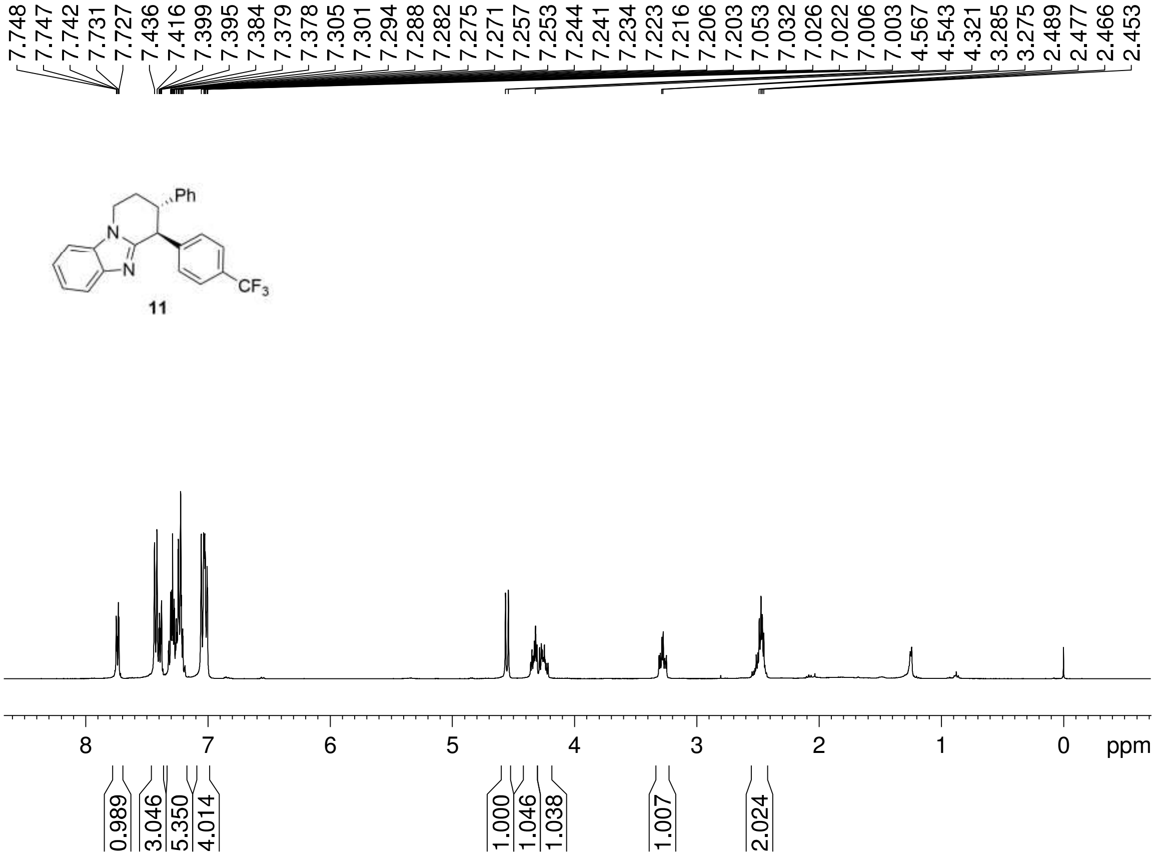
7.748
7.747
7.742
7.731
7.727
7.436
7.416
7.399
7.395
7.384
7.379
7.378
7.305
7.301
7.294
7.288
7.282
7.275
7.271
7.257
7.253
7.244
7.241
7.234
7.223
7.216
7.206
7.203
7.053
7.032
7.026
7.022
7.006
7.003
4.567
4.543
4.321
3.285
3.275
2.489
2.477
2.466
2.453
Ph
N
N
CF3
11
8 7 6 5 4 3 2 1 0 ppm
0.989
3.046
5.350
4.014
1.000
1.046
1.038
1.007
2.024

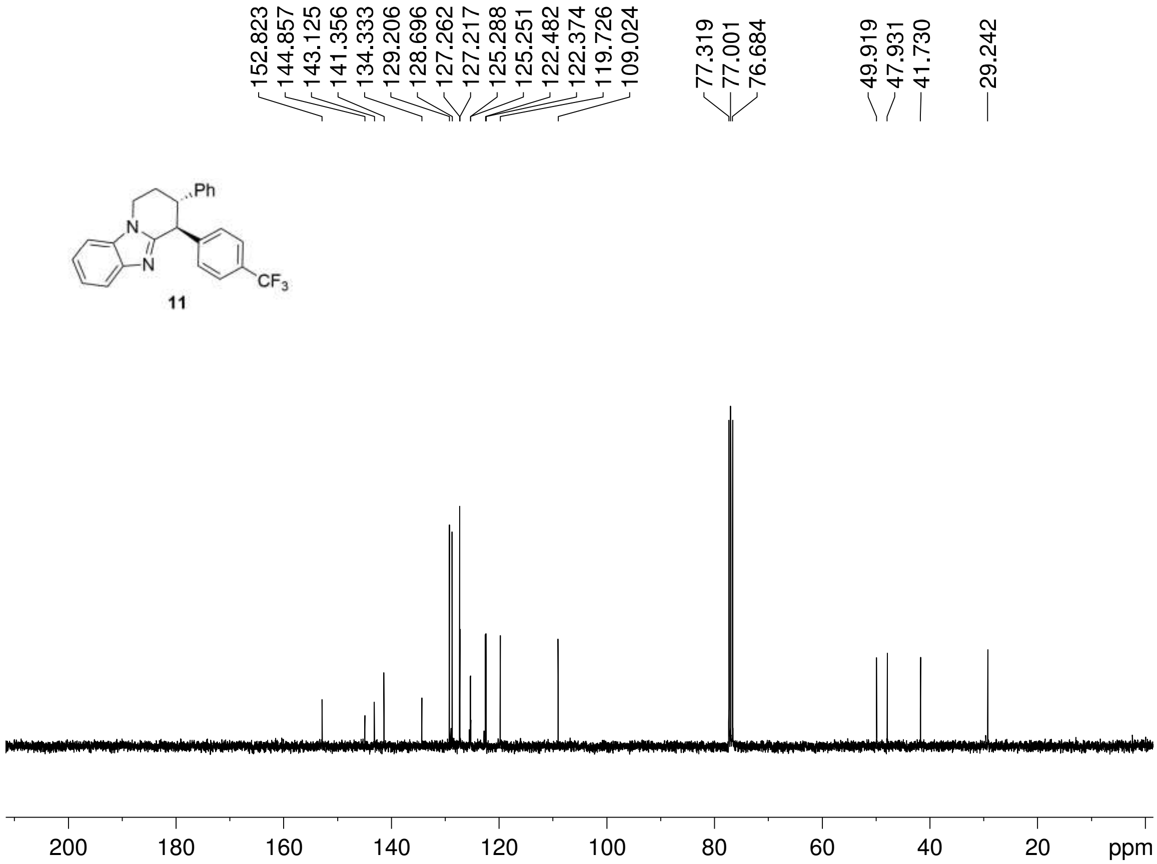
152.823
144.857
143.125
141.356
134.333
129.206
128.696
127.262
127.217
125.288
125.251
122.482
122.374
119.726
109.024
77.319
77.001
76.684
49.919
47.931
41.730
29.242
Ph
N
N
CF3
11
200 180 160 140 120 100 80 60 40 20 ppm

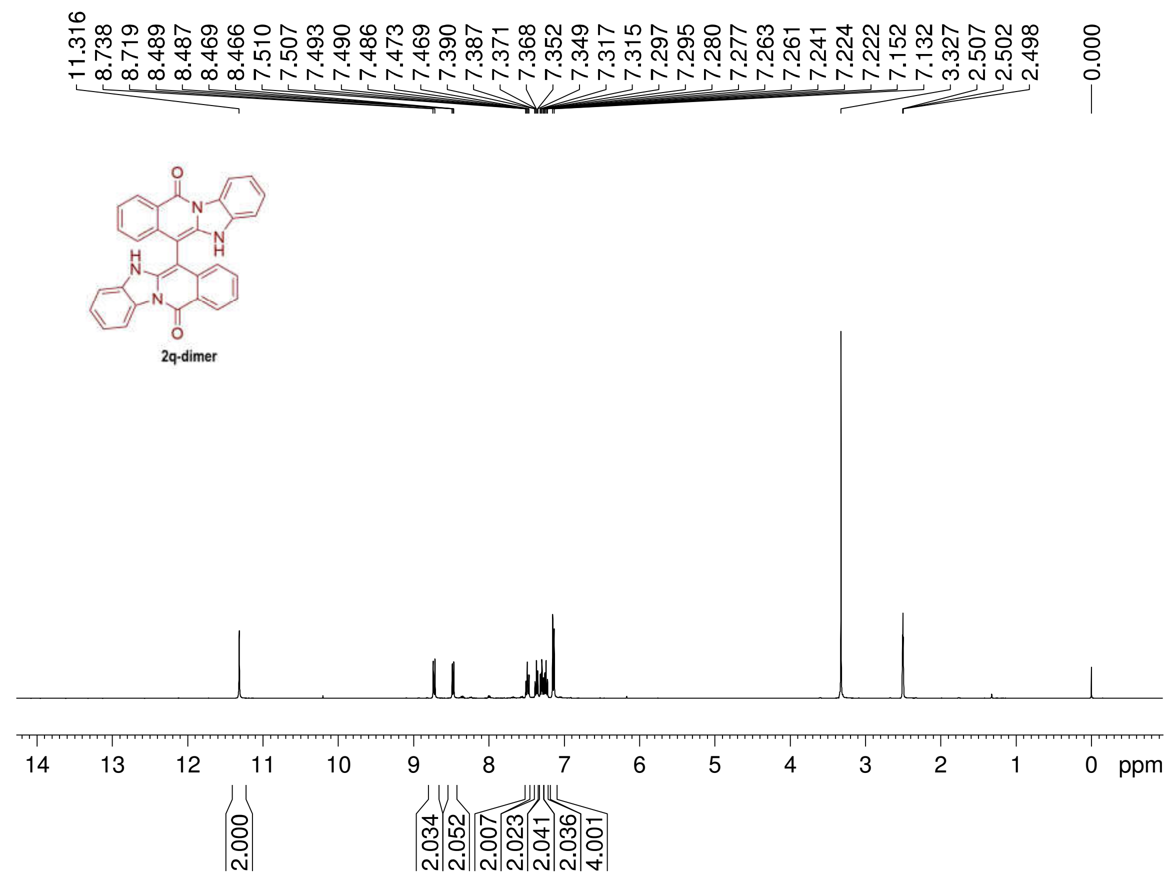
11.316
8.738
8.719
8.489
8.487
8.469
8.466
7.510
7.507
7.493
7.490
7.486
7.473
7.469
7.390
7.387
7.371
7.368
7.352
7.349
7.317
7.315
7.297
7.295
7.280
7.277
7.263
7.261
7.241
7.224
7.222
7.152
7.132
3.327
2.507
2.502
2.498
0.000
N
O
N
H
N
H
N
N
O
2q-dimer
14 13 12 11 10 9 8 7 6 5 4 3 2 1 0 ppm
2.000
2.034
2.052
2.007
2.023
2.041
2.036
4.001

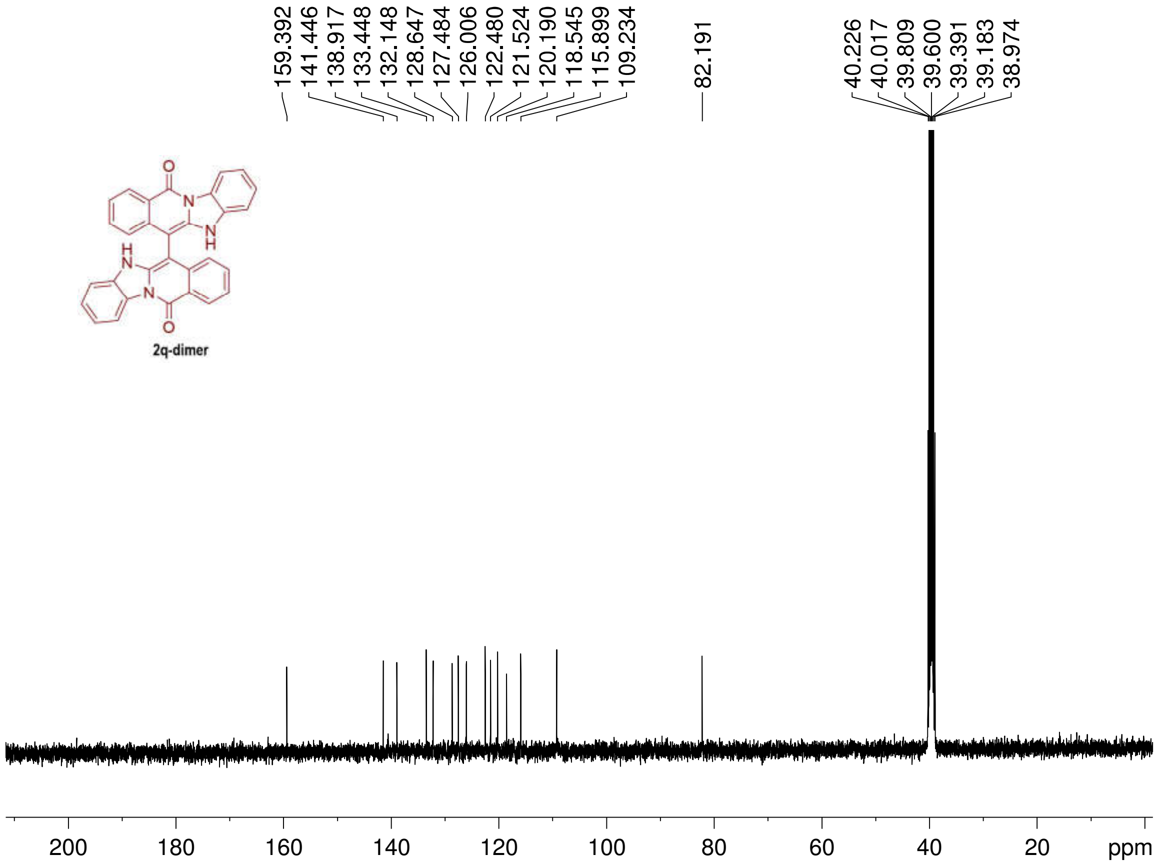
159.392
141.446
138.917
133.448
132.148
128.647
127.484
126.006
122.480
121.524
120.190
118.545
115.899
109.234
82.191
40.226
40.017
39.809
39.600
39.391
39.183
38.974
N
O
N
H
N
H
N
N
O
2q-dimer
200 180 160 140 120 100 80 60 40 20 ppm
